# Paired Immunoglobulin-like Type 2 Receptor Alpha G78R variant alters ligand binding and confers protection to Alzheimer's disease

**DOI:** 10.1371/journal.pgen.1007427

**Published:** 2018-11-02

**Authors:** Nisha Rathore, Sree Ranjani Ramani, Homer Pantua, Jian Payandeh, Tushar Bhangale, Arthur Wuster, Manav Kapoor, Yonglian Sun, Sharookh B. Kapadia, Lino Gonzalez, Ali A. Zarrin, Alison Goate, David V. Hansen, Timothy W. Behrens, Robert R. Graham

**Affiliations:** 1 Department of OMNI Human Genetics, Genentech Inc., South San Francisco, California, United States of America; 2 Department of Microchemistry, Proteomics & Lipidomics, Genentech Inc., South San Francisco, California, United States of America; 3 Department of Immunology and Infectious Diseases, Genentech Inc., South San Francisco, California, United States of America; 4 Department of Structural Biology, Genentech Inc., South San Francisco, California, United States of America; 5 Department of Bioinformatics and Computational Biology, Genentech Inc., South San Francisco, California, United States of America; 6 Department of Neuroscience, Ronald M. Loeb Center for Alzheimer’s disease, Icahn School of Medicine at Mount Sinai, New York, United States of America; 7 Department of Immunology, Genentech Inc., South San Francisco, California, United States of America; 8 Department of Proteomics & Biological Resources, Genentech Inc., South San Francisco, California, United States of America; 9 Department of Neuroscience, Genentech Inc., South San Francisco, California, United States of America; Yale School of Medicine, UNITED STATES

## Abstract

Paired Immunoglobulin-like Type 2 Receptor Alpha (PILRA) is a cell surface inhibitory receptor that recognizes specific *O*-glycosylated proteins and is expressed on various innate immune cell types including microglia. We show here that a common missense variant (G78R, rs1859788) of PILRA is the likely causal allele for the confirmed Alzheimer’s disease risk locus at 7q21 (rs1476679). The G78R variant alters the interaction of residues essential for sialic acid engagement, resulting in >50% reduced binding for several PILRA ligands including a novel ligand, complement component 4A, and herpes simplex virus 1 (HSV-1) glycoprotein B. PILRA is an entry receptor for HSV-1 via glycoprotein B, and macrophages derived from R78 homozygous donors showed significantly decreased levels of HSV-1 infection at several multiplicities of infection compared to homozygous G78 macrophages. We propose that PILRA G78R protects individuals from Alzheimer’s disease risk via reduced inhibitory signaling in microglia and reduced microglial infection during HSV-1 recurrence.

## Author summary

Alzheimer’s disease (AD) is a devastating neurodegenerative disorder resulting from a complex interaction of environmental and genetic risk factors. Despite considerable progress in defining the genetic component of AD risk, understanding the biology of common variant associations is a challenge. We find that PILRA G78R (rs1859788) is the likely AD risk variant from the 7q21 locus (rs1476679) and PILRA G78R reduces PILRA endogenous and exogenous ligand binding. Our study highlights a new immune signaling axis in AD and suggests a role for exogenous ligands (HSV-1). Further, we have identified that reduced function of a negative regulator of microglia and neutrophils is protective from AD risk, providing a new candidate therapeutic target.

## Introduction

Alzheimer’s disease (AD) results from a complex interaction of environmental and genetic risk factors [1]. Proposed environmental risk factors include a history of head trauma [2–4] and infection [5–7]. In recent years, large-scale genome-wide association studies (GWAS) and family-based studies have made considerable progress in defining the genetic component of AD risk, and >30 AD risk loci have been identified [8,9,18–20,10–17].

A key role for microglial/monocyte biology in modulating risk of AD has emerged from analysis of the loci associated with AD risk. Rare variants of TREM2, a microglial activating receptor that signals through DAP12, greatly increase AD risk [11,14]. Beyond TREM2, a number of the putative causal genes mapping to AD risk loci encode microglial/monocyte receptors (complement receptor 1, CD33), myeloid lineage transcription factors (SPI1), and other proteins highly expressed in microglia (including ABI3, PLGC2, INPP5D, and PICALM).

## Results

### PILRA G78R is associated with protection from AD

The index variant for the Alzheimer’s disease risk locus at 7q21 is rs1476679 (meta P value = 5.6 x 10^−10^, odds ratio = 0.91)[15]. In addition to reduced disease risk, the C allele of rs1476679 has been associated with age of onset [21] and lower odds of pathologic AD (plaques and tangles) in the ROSMAP study [22]. In the 1000 Genomes project CEU population (phase 3 data), there were 6 variants in strong linkage disequilibrium (r^2^>0.9) with rs1476679 (S1 Table). None of the 6 variants were predicted to alter regulatory motifs that might influence gene expression (Regulome DBscore ≤ 4), but one variant (rs1859788) encoded a missense allele (G78R, ggg to agg transition) in Paired Immunoglobulin-like Type 2 Receptor Alpha (PILRA) protein. Using a cohort of 1,357 samples of European ancestry whole genome-sequenced to 30X average read-depth (Illumina), we confirmed the strong linkage between rs1476679 (in *ZCWPW1* intron) and rs1859788 (G78R PILRA variant) (S1 Table).

**Fig 1:**
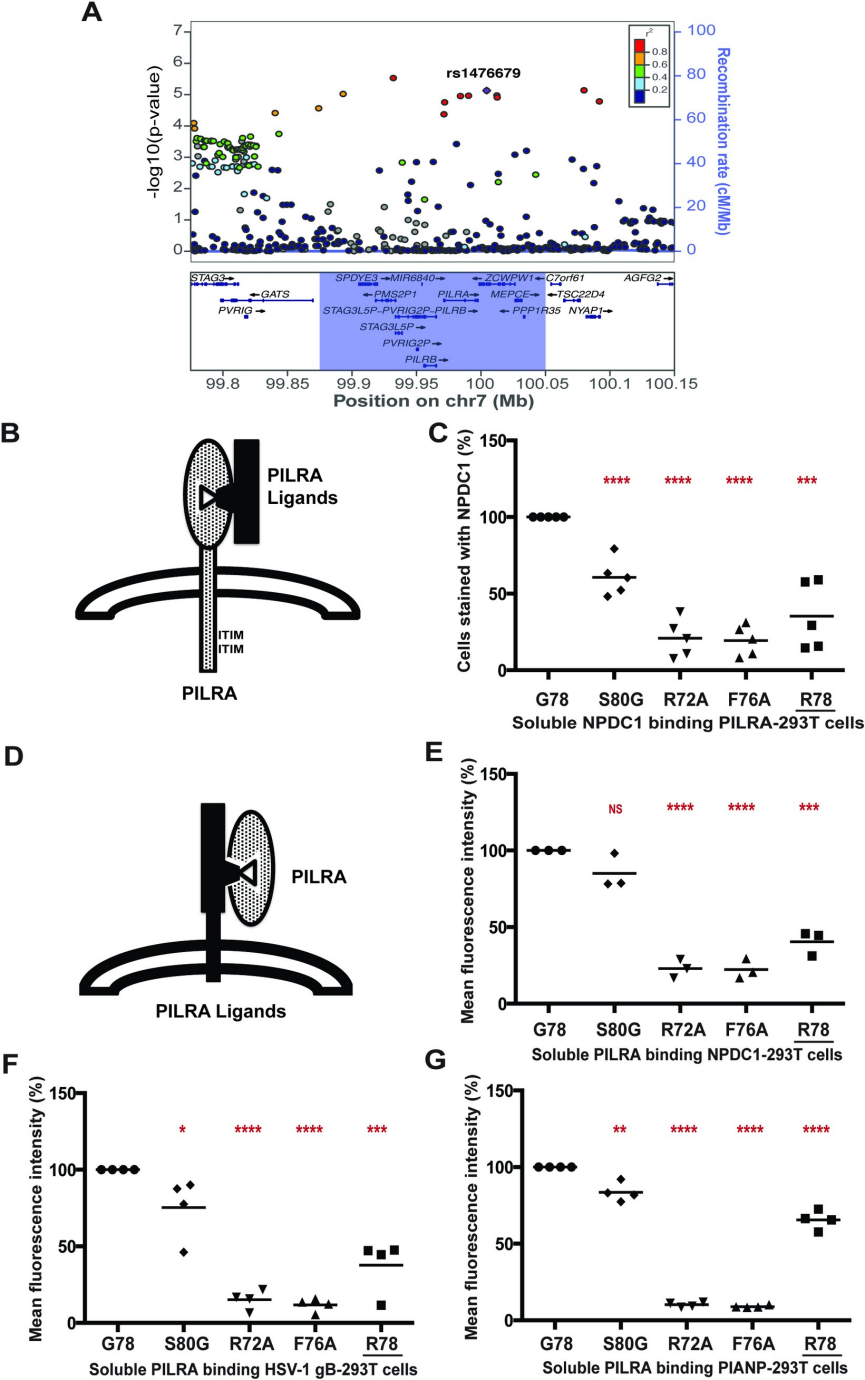
PILRA G78R reduces ligand binding. A) Association of variants in the 7q21 locus with AD risk in the IGAP phase 1 dataset [15]. B,C) 293T cells were transfected with G78 (AD risk) PILRA and several point mutants of PILRA. Binding of NPDC1-mFC to PILRA variant-transfected cells was measured by flow cytometry. The percent of cells expressing PILRA and positive for NPDC1 is indicated in each *panel* considering G78 (AD risk) PILRA binding as 100% for each experiment. D,E,F,G) In the inverse experiment, 293T cells were transfected with different known ligands of PILRA (NPDC1, HSV-1 gB and myc-PIANP). Binding of different PILRA variants to ligand-transfected cells was analyzed by flow cytometry. Results are the percentage of MFI of PILRA-mFC binding on ligand-transfected cells considering G78 (AD risk) PILRA binding as 100% for each experiment. Statistical analysis is two-tailed unpaired t-test (p values <0.05 = *, <0.005 = **, <0.0005 = ***, <0.0001 = ****) performed on 3–5 independent experiments.

We hypothesized that PILRA G78R was the functional variant that accounts for the observed protection from AD risk. As expected from the strong linkage disequilibrium (LD) between PILRA G78R and rs1476679 (Fig 1A), conditional analysis demonstrated that the 2 variants were indistinguishable for AD risk in individuals of European ancestry. In a cohort of 8060 European ancestry samples (a subset of samples described in *19*), individuals homozygous for R78 (OR = 0.72) and heterozygous (OR = 0.89) for R78 were protected from AD risk relative to G78 homozygotes. We note that the allele frequency of PILRA G78R varies considerably in world populations. Indeed *PILRA R78* is the minor allele in populations of African (10%) and European descent (38%) but is the major allele (65%) in East Asian populations [23].

The index variant in the 7q21 locus (rs1476679) has been associated with expression levels of multiple genes in the region, including *PILRB* [24,25]. However, the strongest cis-eQTL in the region is a haplotype tagged by rs6955367 which has a low coefficient of determination to rs1476679 (r^2^ = 0.085, D’ = 0.982) in Europeans and is more strongly associated with expression in whole blood of multiple genes in the region (PILRB, STAG3L5, PMS2P1, MEPCE) compared to rs1476679 [26]. Since the PILRB eQTL P value for rs1476679 is not significant (P = 0.31) after conditioning rs6955367 (S2 Table) in whole blood, we conclude that rs1476679 and rs1859788 are not significant causal eQTLs in the 7q21 region and the observed relationship of these SNPs with *PILRB* expression is due to the weakly correlated variant rs6955367 (S1 Fig). Of interest, the G allele of rs6955367 (increased expression of PILRB) is linked to rs7803454 (r^2^ = 0.83), a variant associated with increased risk of age-related macular degeneration and suggests the presence of independent effects in the PILRA/PILRB region [27].

### PILRA G78R reduces ligand binding

Paired activating/inhibitory receptors are common in the immune system, with the activating receptor typically having weaker affinity than the inhibitory receptor toward the ligands. PILRA and PILRB are type I transmembrane proteins with highly similar extracellular domains that bind certain O-glycosylated proteins [28–31], but they differ in their intracellular signaling domains [32–34]. PILRA contains an immunoreceptor tyrosine-based inhibitory motif (ITIM), while PILRB signals through interaction with DAP12, which contains an immunoreceptor tyrosine-based activation motif (ITAM). Analysis of PILRA knockout mice suggests that PILRA is a negative regulator of inflammation in myeloid cells [35–37], with knockout macrophages showing increased production of cytokines (IL6, IL-1b, KC, MCP-1) in addition to increased infiltration of monocytes and neutrophil via altered integrin signaling.

PILRA is known to bind both endogenous (including COLEC12, NPDC1, CLEC4G, and PIANP) and exogenous ligands (HSV-1 glycoprotein B (gB)) [30,31,36,38]. Because the G78R (R78 (AD protective)) variant resides close to the sialic acid-binding pocket of PILRA, we tested whether the glycine (uncharged, short amino acid) to arginine (basic, long side chain amino acid) substitution might interfere with PILRA ligand-binding activity. All non-human *PILRA* sequences, as well as all *PILRB* sequences, encode glycine at this position. We also generated amino acid point variants in and around the sialic acid-binding pocket of PILRA. A residue conserved among PILR proteins and related SIGLEC receptors, R126 in PILRA, is well known to be essential for sialic acid interaction [29,31,38] and so was not further studied here. Based on their location in the crystal structure, evolutionary conservation [31], and involvement in binding HSV-1 gB [38], amino acids R72 and F76 were predicted to be important for ligand binding and were substituted to alanine as positive controls for loss-of-function [31]. In addition, S80, a residue outside of the sialic acid-binding pocket was substituted to glycine. The R72A, F76A, and S80G mutations have not been detected in human populations (dbSNP v147).

To study receptor-ligand binding, 293T cells were transfected with G78 (AD risk) PILRA or variants, and then incubated with purified NPDC1-mIgG2a protein (Fig 1B), followed by flow cytometry to detect PILRA and the NPDC1 fusion protein. Among known PILRA ligands, NPDC1 is expressed in the central nervous system and binds with high affinity to PILRA [31]. Expression of the PILRA variants on the transfected 293T cells was comparable to or greater than G78 (AD risk) PILRA (S2 Fig). G78 (AD risk) PILRA binding to NPDC1 was considered 100%. Both R72A and F76A mutations severely impaired NPDC1 binding (~20% of G78, p-value < 0.0001). The R78 (AD protective) variant also showed significantly reduced ligand binding (~35% of G78, p < 0.0005), while the G80 mutant was the least affected (~60% of G78, p < 0.0001) (Fig 1C and S3A and S3B Fig).

To further test the hypothesis that the AD protective PILRA R78 variant impacts ligand binding, NPDC1 or alternative PILRA ligands HSV-1 gB and PIANP were expressed on the cell surface of 293T cells, and the binding of purified PILRA protein variants was measured by flow cytometry. PILRA R78 showed reduced binding to the various ligands in these assays as compared to G78 (Fig 1D to 1G and S4A to S4G Fig). These data confirmed that the R78 variant impairs ligand-binding activity of PILRA.

### Identification of C4A as PILRA ligand

**Fig 2:**
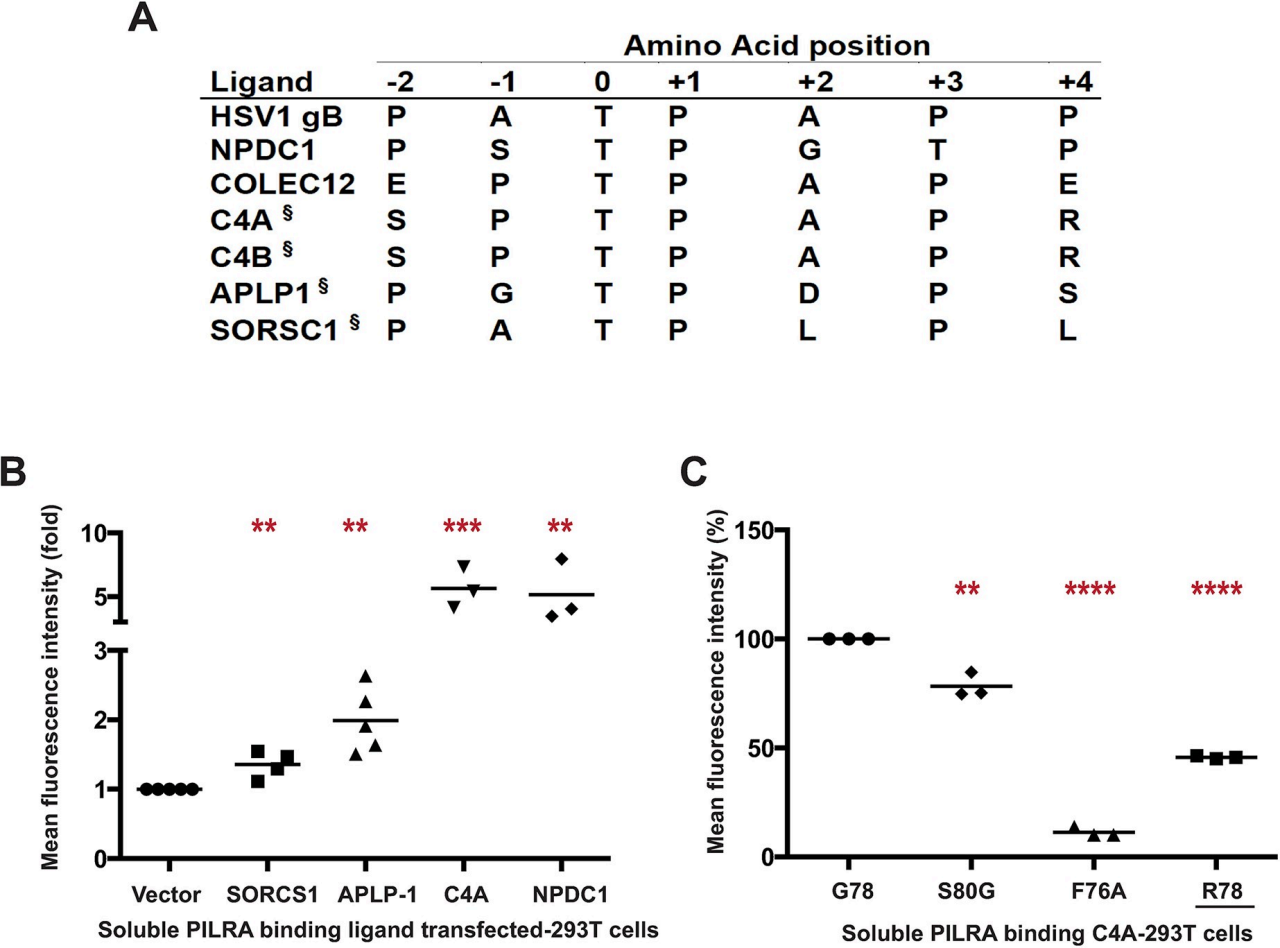
C4A is a novel ligand for PILRA. A) Comparison of the peptide sequence around the O-glycosylated Thr (position 0) of known and putative (§) PILRA ligands. B) 293T cells were transfected with putative ligands of PILRA (SORCS1 ECD, APLP1 ECD. or full length C4A) fused with C-terminal glycoprotein D (gD) tag and GPI anchor, or full length NPDC1 as positive control. Binding of G78 (AD risk) PILRA to ligand-transfected cells was analyzed by flow cytometry. Results are fold-increase in binding to each putative ligand compared to vector control for each experiment. C) 293T cells were transfected with full length C4A fused with C-terminal gD tag and GPI anchor. Binding of different PILRA variants to C4A-transfected cells was analyzed by flow cytometry. Results are the percentage of MFI of PILRA-mFc binding on ligand-transfected cells considering G78 (AD risk) PILRA binding as 100% for each experiment. Statistical analysis is two-tailed unpaired t-test (p values <0.05 = *, <0.005 = **, <0.0005 = ***, <0.0001 = ****) performed on 3–5 independent experiments.

A peptide motif for PILRA interaction has been established (Fig 2A) that includes an O-glycosylated threonine, an invariant proline at the +1 position, and additional prolines at the -1 or -2 and +3 or +4 positions [31,38]. Of note, PILRA is capable of binding murine CD99 and human NPCD1 (both contain the consensus motif), but not human CD99 or murine NPCD1 (both lack the consensus motif), suggesting divergence between human and mouse in the range of endogenous ligands bound by PILRA [31].

We sought to identify novel endogenous PILRA ligands by searching for human proteins with either the PTPXP, PTPXXP, PXTPXP or PXTPXXP motif. A total of 1540 human proteins carry at least 1 of these putative PILRA-binding motifs (S3 Table). Narrowing the search, we considered proteins with the motif that have previously been shown to be O-glycosylated in human cerebral spinal fluid [39], and measured the binding of these proteins to PILRA variants. By flow cytometry, complement component 4A (C4A) bound to G78 (AD risk) PILRA in a manner comparable to NPDC1, while APLP1 and SORCS1 showed relatively little interaction with PILRA (Fig 2B and S5A and S5B Fig). We further demonstrated that the PILRA R78 (AD protective) variant has reduced binding for C4A (Fig 2C and S5C Fig). We did not test C4B, but its putative PILRA-binding motif is identical to that of C4A.

### G78R stabilizes the ligand-free state of PILRA

**Fig 3:**
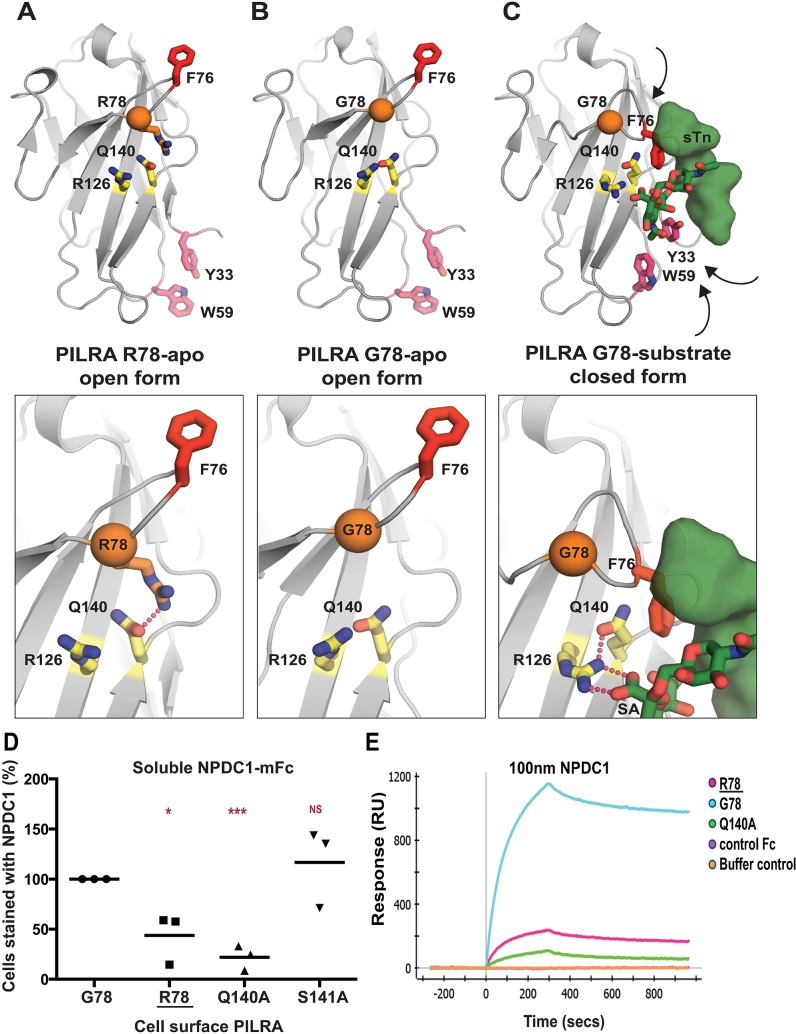
Structural determinants of PILRA in apo and ligand-bound conformations. A) The unliganded crystal structure of R78 (AD protective) PILRA (PDB 4NFB) displays an “open” conformation with an unformed sialic acid (SA) binding site. B) The apo crystal structure of G78 (AD risk) PILRA (PDB 3WUZ) reveals that the Q140-R126 interaction network (productive for SA-coordination) is pre-formed and the “downward” movement of F76 is not impeded in the R78 side chain mediated interactions. C) The sialylated *O*-linked sugar T antigen (sTn)-bound PILRA structure (PDB 3WV0) reveals the ligand-induced conformational changes across the receptor that lead to engagement of the SA-motif by direct coordination to R126 and the critical involvement of F76 in peptide (sTn ligand) recognition. Aromatic residues including Y33 (fuchsia) and W59 (fuchsia) also undergo significant ligand-induced conformational changes. D) 293T cells were transfected with G78 (AD risk) PILRA and several point mutants of PILRA. Binding of NPDC1-mFC to PILRA variant-transfected cells was measured by flow cytometry. The percent of cells expressing PILRA and positive for NPDC1 is indicated in each *panel* considering G78 PILRA binding as 100% for each experiment. Statistical analysis is two-tailed unpaired t-test. E) Binding of NPDC1.mFC to PILRA variants by surface plasmon resonance (SPR).

To understand the conformational changes that might occur in the PILRA sialic acid-binding pocket during receptor-ligand interactions in the presence of G78 (AD risk) or R78 (AD-protective) variants, we evaluated available experimental crystal structures (Fig 3A to 3C) [38,40]. Structures of G78 (AD risk) PILRA reveal a monomeric extracellular domain with a single V-set Ig-like β-sandwich fold that binds O-glycan ligands (Fig 3B and 3C) [38]. By analogy to a molecular clamp, the sialic acid-binding site in PILRA undergoes a large structural rearrangement from an “open” to a “closed” conformation upon binding its peptide and sugar ligands simultaneously (Fig 3A to 3C). The essential R126 side-chain engages the carboxyl group of sialic acid (SA) directly in a strong salt bridge (Fig 3C). The CC’ loop which contains F76 and G78 undergoes a large conformational change where F76 translates ~15 Å to participate in key interactions with the peptide of the ligand and abut the Q140 side-chain of PILRA (Fig 3B and 3C). In this ligand-bound “closed” conformation of PILRA, Q140 helps to position R126 precisely for its interaction with SA (Fig 3C).

Notably, in the structure of R78 (AD protective) PILRA crystallized in the absence of any ligand [40], the long side-chain of R78 is observed to hydrogen bond with Q140 directly (Fig 3A). This unique R78-Q140 interaction has three important consequences: 1) it sterically hinders F76 from obtaining a ligand-bound “closed” conformation, 2) it affects the ability of R126 to interact with the carboxyl group of SA by altering the R126-Q140 interactions observed in G78 (AD risk) PILRA and, 3) it likely alters CC’ loop dynamics, (Fig 3B to 3C). Overall, the structure of the R78 (AD protective) variant shows that this single side-chain alteration appears to stabilize the “open” apo form of PILRA and likely alters the conformational sampling of the molecular clamp required to obtain its “closed” form to engage its ligands.

We therefore propose that in G78 PILRA (AD-risk associated), the engagement of SA by R126 and peptide by F76 is facilitated by G78 (Fig 3C). However, in the AD-protective PILRA variant R78, the R78 side-chain competes with the central R126-Q140 interaction and alters the positioning of F76 (Fig 3A), which leads to an overall decrease in PILRA ligand binding. This structure-based hypothesis is consistent with the reduced functional cellular binding observed for the R78 variant (Fig 1).

To further test this model, we generated two additional alanine mutants of PILRA at amino acids predicted to be essential (Q140) or non-essential (S141) for conformational changes associated with ligand interaction. 293T cells were transfected with G78 (AD risk), R78 (AD protective), Q140A and S141A variants of PILRA, and receptor-ligand interaction was measured after incubating cells with soluble NPDC1-mIgG2a. PILRA expression was comparable among variants, matching or exceeding G78 (AD risk) expression (S2 Fig). R78 (44% of G78, p = 0.02) and Q140A (22% of G78, p = 0.0004) variants showed significantly decreased binding to NPDC1, while S141A (117% of G78, p = 0.5) had no significant effect (Fig 3D and S6A and S6B Fig). These data are consistent with the experimental structural models that show the interaction of Q140 with R126 is important for productive sialic acid binding (Fig 3A to 3C). Consistently, the Q140A mutation has a strong effect because the Q140-R126 interaction network is completely abolished. By contrast, the AD-protective R78 variant likely has an intermediate effect since it only modulates the Q140 interaction with R126, which is expected to only alter the frequency or strength of relevant PILRA-ligand interactions.

### PILRA G78R reduces the on-rate of ligand binding

We next investigated the interaction of PILRA variant and ligands *in vitro* using surface plasmon resonance (SPR). Human PILRA–Fc variants (G78, R78, or Q140A) were immobilized on a ProteOn GLC sensor chip and binding of NPDC1-mFc or a control mFc-tagged protein was measured. Qualitatively, NPDC1-Fc bound to the R78 (AD-protective) and Q140A (essential for R126 conformation) variants to a much lesser extent than to G78 (AD risk) PILRA, while control Fc-tagged protein showed no binding (Fig 3E).

To further probe the mechanistic basis of R78 (AD protective) function and phenotype, a more complete SPR characterization of NPDC1-His binding to PILRA variants was performed (S6C Fig). The affinity of NPDC1 toward R78 (AD-protective) PILRA (76.5 nM) was 4.5-fold weaker than the affinity toward G78 PILRA (16.8 nM). The on-rate constant *k*_on_ for NPDC1-His binding to R78 (AD protective) (6.8×10^+3^ M^-1^s^-1^) was ~3-fold lower than binding to G78 (AD risk) PILRA (2.2×10^+4^ M^-1^s^-1^), while the *k*_off_ rate constants were comparable (S6C Fig). These results are consistent with the idea that, once engaged, the affinity and disassociation rate of R78-ligand complexes are similar to G78 PILRA, but the frequency with which PILRA can productively engage with ligands is reduced in the R78 (AD protective) variant by R78 side chain interactions favoring the apo-state (Fig 3). Taken together, these data support a structural model in which R78 impairs PILRA-ligand interactions by altering the accessibility of a productive sialic acid-binding conformation in PILRA.

### PILRA G78R reduces entry of HSV-1 into hMDMs

Given that PILRA is a known entry receptor for HSV-1 [41] and the R78 (AD protective) variant showed reduced binding to HSV-1 gB (Fig 1F), we next determined whether there were differences in HSV-1 infectivity based on PILRA genotype. We isolated and differentiated human monocyte-derived macrophages (hMDMs) from five pairs of healthy volunteers homozygous for either the G78 (AD risk) or R78 (AD protective) PILRA variants (matched for age, gender and ethnicity). hMDMs were infected with HSV-1 at different multiplicities of infection (MOI) (0.01, 0.1, 1 and 10), and infectivity was measured morphologically by light microscopy, by using an LDH cytotoxicity assay, by measuring intracellular viral DNA and in a viral plaque assay.

**Fig 4:**
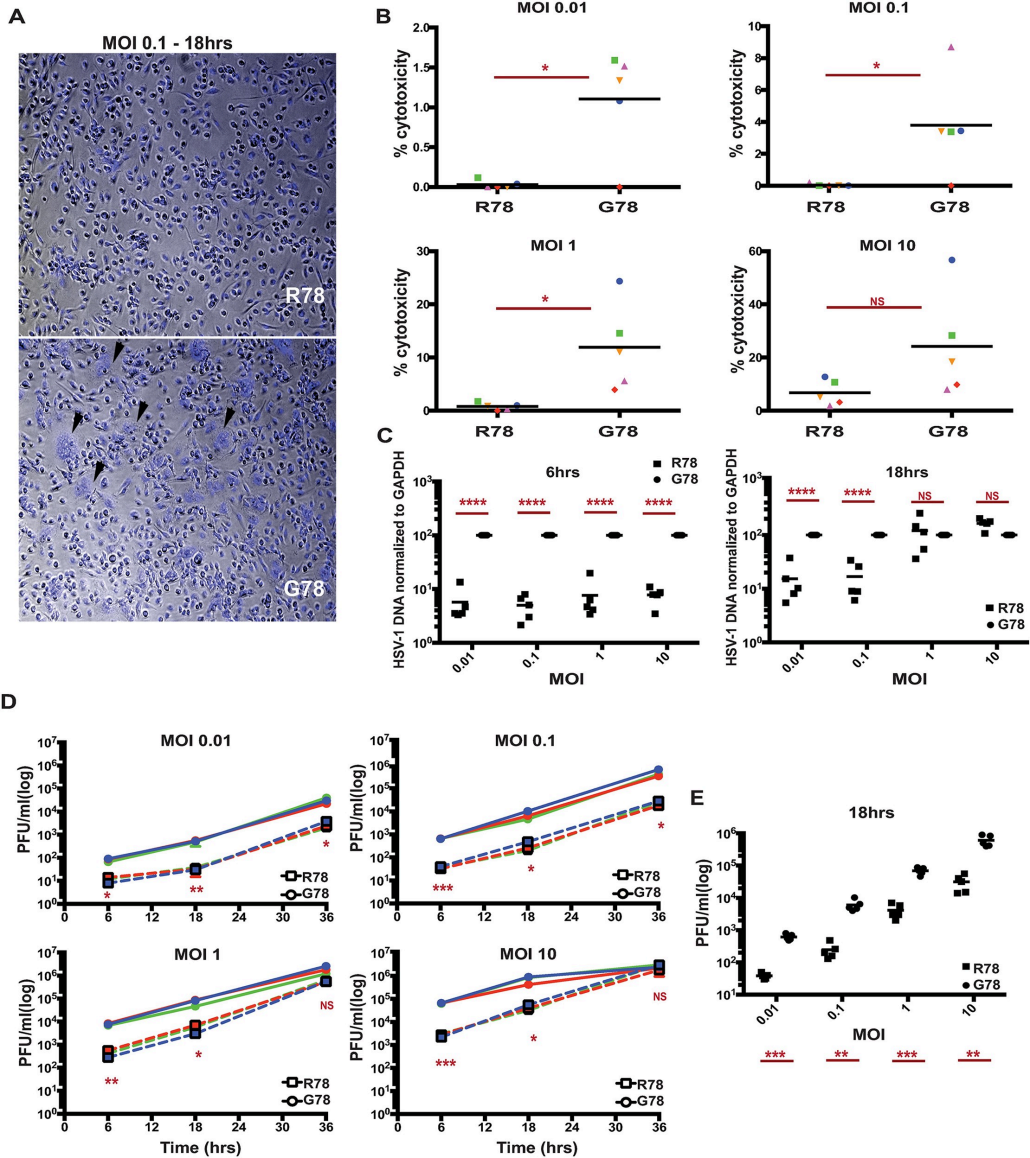
PILRA G78R reduces entry of HSV-1 into hMDMs. A) Representative images of hMDM, infected with HSV-1 for 18 hrs at MOI 0.1, were fixed and stained with DAPI. B) LDH cytotoxicity assay was performed on supernatants harvested from HSV-1-infected hMDMs after 18 hrs. Results are % cytotoxicity—amount of LDH in supernatant after infection compared to LDH released from cells completely lysed by lysis buffer, with completely lysed cells (maximum LDH release) considered as 100% for each donor. Each shape represents one donor pair. C) HSV-1 DNA was quantitated on DNA extracted from HSV-1-infected hMDMs after 6 and 18 hrs by qPCR. Results are % HSV-1 DNA normalized to GAPDH considering G78 donor as 100% for each donor pair. D,E) Viral titers in the supernatant of HSV-1-infected hMDMs were determined by plaque assay on Vero cells. Results are number of plaque forming units (pfu) per ml of supernatant collected from HSV-1-infected hMDMs. (D) 6, 18 and 36 hrs of infection (G78, solid lines; R78, dashed lines)(experiment 1) (E) 18 hrs of infection (data from two individual experiments). Statistical analysis is two-tailed paired (B,D,E) or unpaired (C) t-test performed on 3–5 genotyped individual donor pairs (p values <0.05 = *, <0.005 = **, <0.0005 = ***, <0.0001 = ****).

No notable cytopathic effects were observed in the first 6 h of infection, however at 18 hours post infection, extensive cytopathy was detected in G78/G78 PILRA-expressing hMDMs, including loss of cell shape, increased cell volume, birefringence, and formation of both cell aggregates and multinucleated giant cells (syncytia) (Fig 4A and S7 Fig). Cytopathic changes were less pronounced in R78/R78 (Alzheimer’s protective) homozygous hMDMs (Fig 4A and S7 Fig).

hMDMs from R78/R78 PILRA donors showed significantly less HSV-1-induced cytotoxicity at 18 hrs post infection in the LDH assay at 0.01, 0.1, or 1 MOI (Fig 4B and S4 Table). The difference was no longer significant at 10 MOI or if the infection was allowed to proceed for 36 hrs, except at the lowest MOI of 0.01 (Fig 4B, S8A Fig and S4 and S5 Tables).

hMDMs from R78/R78 donors showed 5–10 fold decreased amounts of HSV-1 DNA at 6 hrs at all MOIs (0.01, 0.1, 1 and 10), and at 18 hrs at lower MOIs (0.01 and 0.1), compared to those from G78/G78 donors (Fig 4C and S8C and S8D Fig). No significant differences in HSV-1 DNA were observed between the two genotypes at 18 hrs at higher doses (1 and 10 MOI) (Fig 4C and S8D Fig), or at 36 hrs for any dose of virus (S8B and S8E Fig).

Finally, we measured the amount of infectious HSV-1 virus by harvesting supernatants from HSV-1-infected hMDMs and measuring viral titer by plaque assays on Vero cells. Viral plaque forming units (PFUs) were significantly lower after 6 and 18 hrs of infection for all MOIs tested, and at 36 hrs for lower MOIs (Fig 4D and 4E, and S9 Fig). Taken together, these data indicate that R78/R78 macrophages were less susceptible to HSV-1 infection than G78/G78 macrophages.

## Discussion

We show here that PILRA G78R is a likely causal variant conferring protection from AD risk at the 7q21 locus. G78R alters the access to SA-binding pocket in PILRA, where R78 PILRA shows reduced binding to several of its endogenous cellular ligands and with HSV-1 gB. Reduced interaction with one or more of PILRA’s endogenous ligands (including PIANP and NPDC1) could impact microglial migration or activation [35–37]. In fact, microglia up-regulate the expression of the *PIANP* gene in the PS2APP, 5xFAD, and APP/PS1 mouse models of AD [42–44]. The identification of C4 as a novel PILRA-interacting protein is also intriguing, given the increased expression of C4 in mouse AD models [42], the increase in amyloid deposition observed when complement activation is inhibited [45], and the genetic association of complement receptor 1 with AD [46]. Finally, we note that both TREM2 and PILRB function as activating receptors and signal through DAP12 [32,34,47]. A reduction of PILRA inhibitory signals in R78 carriers could allow more microglial activation via PILRB/DAP12 signaling and reinforce the cellular mechanisms by which TREM2 is believed to protect from AD incidence [48]. The relevant ligands for PILRA/PILRB *in vivo* and the mechanism by which reducing PILRA-ligand interaction confers protection from Alzheimer’s disease remain to be elucidated.

A role for infection in accelerating AD has been proposed, but remains controversial [49]. HSV-1 is a neurotropic virus that infects a large fraction of the adult population and has frequent reactivation events. HSV-1 has been implicated in AD pathogenesis by several lines of evidence, including the presence of HSV-1 viral DNA in human brain tissue [50,51], increased HSV-1 seropositivity in AD cases [52–55], the correlation of high avidity HSV-1 antibodies with protection from cognitive decline [55], the binding of HSV-1 gB to APOE-containing lipoproteins [56], HSV-1-induced amyloidogenic processing of amyloid precursor protein (APP) [57–59], and preferential targeting of AD-affected regions in HSV-1 acute encephalitis [60]. In addition, HSV-1 gD receptors and gB receptor PILRA increase with age in multiple brain regions, including the hippocampus [61]. Additional AD risk loci have been proposed to play a role in the life cycle of HSV-1 [62], including CR1, which is capable of binding HSV-1 [63]. The reduced infectivity of HSV-1 in R78/R78 macrophages suggests that brain microglia from R78/G78 and R78/R78 individuals are less susceptible to HSV-1 infection and more competent for immune defense during HSV-1 recurrence.

These data provide additional evidence for a key role of microglia in AD pathogenesis and provide a mechanism by which HSV-1 may contribute to AD risk. Inhibiting the interaction of PILRA with its ligands could therefore represent a novel therapeutic mechanism to prevent or slow AD progression.

## Materials and methods

### Ethics statement

Blood samples and genotypes from healthy human volunteers from the Genentech Genotype and Phenotype program (gGAP) were used in this project. Written consent was obtained from all participants in the gGAP program. The study was reviewed and approved by the Western Regional Institutional Board (Study Number: 1096262, IRB Tracking Number: 20080040).

### Alzheimer’s disease case/control datasets

The conditional analysis between rs1476679 and rs1859788 was performed using the Genome-wide Complex Trait Analysis (GCTA) program’s Conditional & joint (COJO) analysis option. This program takes summary statistics as input. We used the summary statistics for rs1859788, rs1476679 from IGAP stage1 GWAS [15]. The COJO program also needs a reference population to calculate the LD and to perform the conditional analysis. For reference population analysis we used the raw genotype data from ADGC cohort. There were 22,255 individuals in this cohort that had the non-missing genotype for the rs1859788. The ADGC dataset was also used for the minor allele frequency calculations that are provided in the text.

### PILRA variants and PILRA ligands expression and purification

The coding sequences (CDS) of full length PILRA (AJ400841), human herpesvirus 1 strain KOSc glycoprotein B (HSV-1 gB) (EF157316), and neural proliferation, differentiation and control 1 (NPDC1) (NM_015392.3) were cloned in the pRK neo expression vector. Several PILRA point mutations were generated, including R72A, F76A, G78R, S80G, Q140A and S141A. The PILRA variants were incorporated into a full-length G78 (AD risk) PILRA construct by site-directed mutagenesis as per the manufacturer’s recommendation (Agilent Cat. No. 200523) and sequences were verified. A full length myc-DDK tagged PIANP construct was purchased from Origene (Cat. No. RC207868). Full length complement component 4A (Rodgers blood group) C4A (NM_007293.2), extra cellular domain (ECD) of amyloid beta precursor like protein 1 (APLP1) (NM_005166) (1–580 aa) and ECD of sortilin-related VPS10 domain-containing receptor 1 (SORCS1) (NM_052918) (1–1102 aa) were fused with C-terminal gD tag (US6/gD, partial [Human alphaherpesvirus 1) (AAP32019.1) and GPI anchor in pRK vector. The ECD of all PILRA variants (1–196 aa) and NPDC1 (1–190 aa) were PCR amplified and cloned with C-terminal murine IgG2a Fc tag in a pRK expression vector.

ECDs of PILRA variants (G78 (AD risk), R72A, F76A, G78R, S80G, Q140A and S141A) and NPDC1 fused to the Fc region of murine IgG2a were expressed in a CHO cell expression system, supernatants collected, protein A/G affinity-purified and verified by SDS-PAGE and mass spectroscopy.

### Relative PILRA-ligand binding to PILRA variant transfected cells

293T cells were transfected with lipofectamine LTX reagent (ThermoFisher) with various full-length constructs of PILRA variants (G78 (AD risk), R72A, F76A, G78R, S80G, Q140A and S141A). After 48 hours, the transfected cells were harvested and incubated with soluble mIgG2a-tagged ligand, NPDC1-mFc at 50 μg/ml (as described above) for 30 minutes on ice. Cells were then washed and stained with 1 μg/ml chimeric anti-PILRA antibody (mouse Fc region is substituted to human IgG1 backbone on anti-PILRA antibodies [31]) on ice for 30 min followed by APC-conjugated mouse anti-human IgG (BD Pharmingen Cat.No. 550931) and FITC anti-mouse IgG2a (BD Pharmingen Cat. No. 553390) secondary antibodies according to manufacturer’s instruction. PILRA-transfected 293T cells were examined by flow cytometry for binding of NPDC1 by measuring the frequency of APC and FITC double-positive cells. Double positive cells were gated on the WT sample and than the gates were overlaid on subsequent samples to maintain the same cell population throughout the experiment. For each PILRA variant, the mean percentage of the number of cells binding to NPDC1-mFC relative to the wild type PILRA binding for each experiment was calculated.

### Relative PILRA variant binding to PILRA ligand transfected cells

In the inverse experiment, 293T cells were transfected with lipofectamine LTX reagent [ThermoFisher] with known full-length PILRA ligand (NPDC1, HSV-1gB and PIANP) and predicted ligand constructs (SORCS1, APLP1 and C4A) (described above). After 48 hours, the transfected cells were harvested and incubated with soluble mIgG2a-tagged variants of PILRA (G78 (AD risk), R72A, F76A, G78R, S80G) (described above) 50 μg/ml for 30 min on ice. Cells were then washed and stained with FITC anti-mouse IgG2a (BD Pharmingen Cat. No. 553390) secondary antibody according to manufacturer’s instruction. PILRA ligand-transfected 293T cells were examined by flow cytometry for binding to PILRA variants by measuring the frequency of FITC-positive cells. The percentage of mean fluorescence intensity (MFI) of PILRA-mFC binding on ligand-transfected cells relative to the wild type PILRA binding for each experiment was calculated.

### PILRA variant ligand binding Surface Plasmon Resonance (SPR)

Binding of human NPDC1.Fc to PILRa-Fc variants was measured by SPR using a ProteOn XPR36 (Bio-Rad). PILRA-Fc WT and variants (G78R and Q140A) were immobilized on a ProteOn GLC sensor chip (Bio-Rad) by EDC/NHS amine coupling (2000–2400 RU’s) and the chip surface was deactivated by ethanolamine after immobilization. NPDC1-Fc diluted in PBST or a control Fc-tagged protein was injected at a concentration of 100 nM over the immobilized PILRA proteins at room temperature[31].

### Isolation and differentiation of monocytes

Healthy human volunteers from the Genentech Genotype and Phenotype program (gGAP) were genotyped for rs1859788 (PILRA G78R) using custom design ABI SNP genotyping assay with the following primers; Forward primer seq: GCGGCCTTGTGCTGTAGAA, Reverse primer seq: GCTCCCGACGTGAGAATATCC, Reporter 1 sequence: VIC- ACTTCCACGGGCAGTC-NFQ, Reporter 2 sequence: FAM- ACTTCCACAGGCAGTC-NFQ. To control for a possible effect of the eQTL for PILRB, all volunteers selected were homozygous AA (lower PILRB expression) for rs6955367 (http://biorxiv.org/content/early/2016/09/09/074450). Genotype for rs6955367 was determined using an InfiniumOmni2.5Exome-8v1-2_A.bpm. Peripheral Blood Mononuclear Cells (PBMC’s) were obtained by Ficol gradient from five pairs of homozygous donors for rs1859788 (one with each genotype AA/GG). The pairs of samples were matched for age [± 5 years], gender and self-reported ethnicity. Monocytes were purified from PBMC’s by negative selection using the EasySep Human Monocyte Enrichment Kit without CD16 Depletion (19058), as recommended by the manufacturer. Isolated monocytes were differentiated into macrophages in DMEM + 10%FBS + 1X glutaMax and 100 ng/ml MCSF media for 7–10 days. The gGAP program was reviewed and approved by the Western Regional Institutional Board.

### HSV-1 infection of macrophages

Macrophages differentiated from healthy human monocytes were incubated with 10, 1, 0.1 and 0.01 multiplicity of infection (MOI) of HSV-1 virus at 37°C for 1 hour with gentle swirling to allow virus adsorption. Cells were washed after 1 hr of adsorption and infection was continued for 6, 18 and 36 hrs. Supernatant was harvested at 6, 18 and 36 hrs of infection and cell debris were removed by centrifugation at 3000 rpm for 5 min at 4°C. DNA was isolated from infected cells using the QIAamp DNA mini-kit (Qiagen Cat. No. 51304). Additional cells were fixed with 4% paraformaldehyde after infection and stained with DAPI for microscopy.

### Lactate Dehydrogenase (LDH) cytotoxicity assay

The CytoTox 96 Non-Radioactive Cytotoxicity Assay (Promega Cat. No. E1780) was performed on supernatant harvested from HSV-1-infected human macrophages as per manufacturer’s recommendations to measure cell toxicity after HSV-1 infection. For each sample, the percent cytotoxicity was calculated as the ratio of LDH released in culture supernatant after infection to completely lysed cells (maximum LDH release).

### Quantitative polymerase chain reaction

HSV-1 DNA was quantitated using a custom design ABI TaqMan gene expression assay, with the following primers: Forward primer seq: 5'-GGCCTGGCTATCCGGAGA-3', Reverse primer seq: 5'-GCGCAGAGACATCGCGA-3', HSV-1 probe: 5'-FAM-CAGCACACGACTTGGCGTTCTGTGT-MGB-3'. GAPDH DNA was quantitated using ABI endogenous control (Applied Biosystem Cat. No. 4352934E). Amplification reactions were carried out with 5 μl of extracted DNA from infected cells in a final volume of 25 μl with TaqMan Universal PCR Master Mix (Applied Biosystems Cat. No. 4304437) as per manufacturer’s recommendations. HSV-1 DNA (Ct values) was normalized to cell GAPDH (Ct values) to account for cell number.

### HSV-1 plaque assay

Virus titers from HSV-1-infected cells were determined following a standard plaque assay protocol [64]. In brief, the plaque assay was performed using Vero cells (African Green Monkey Cells) seeded at 1x10^5^ cells per well in 48-well plates. After overnight incubation at 37°C, the monolayer was ~90–100% confluent. Supernatants harvested from HSV-1-infected human macrophages were clarified from cells and debris by centrifugation at 3000 rpm for 5 minutes at 4°C. Virus-containing supernatants were then diluted from 10^−1^ to 10^−8^ in DMEM (1 ml total volume). Growth media was removed from Vero cells and 250 μl of supernatant dilution was transferred onto the cells, followed by incubation at 37°C for 2 hrs with gentle swirling every 30 min to allow virus adsorption, after which the virus-containing media was aspirated. The cells were then overlaid with 2% methylcellulose containing 2X DMEM and 5% FBS and incubated at 37°C. 48 hrs post-infection, plaques were enumerated from each dilution. Virus titers were calculated in pfu/ml.

## Supporting information

S1 Fig **A) 7q21 AD risk locus**. The genomic location and linkage relationship (r^2^) of the index variant for the AD risk (rs1476679), PILRA missense allele (G78R) and the PILRB eQTL (rs6955367).
**B) The AD risk variant does not co-localize with the PILRB eQTL**. The association (-logP) of variants in the 7q21 region with mRNA levels in whole blood (GTEX data) is displayed along the y-axis, and association with AD risk (IGAP Phase 1 data) is shown along the x-axis.
(TIF)

S2 Fig Expression of PILRA variants in 293T cells. 293T cells were transfected with various constructs of PILRA (G78 (AD risk), R72A, F76A, R78 (AD protective), S80G, Q140A and S141A) and stained with anti-PILRA antibody followed by APC-conjugated anti-human IgG1 secondary to check the expression of PILRA by flow cytometry.
(TIF)

S3 Fig G78R impairs PILRA ligand binding. A,B) 293T cells were transfected with various constructs of PILRA (G78 (AD risk), S80G, R72A, F76A and R78 (AD protective)). 48 hrs. after the transfection cells were harvested and incubated with soluble mIgG2a tagged ligand (NPDC1-mFc, 50 μg/ml) for 30 min on ice for receptor-ligand interactions. Cells were than stained with anti-PILRA (APC) and anti-mIgG2a (FITC).
(TIF)

S4 Fig A) 293T cells were transfected with the expression vector or various PILRA ligand constructs (NPDC1, HSV1gB and PIANP). 48 hrs. after the transfection cells were harvested and incubated with 50 μg/ml soluble mIgG2a-tagged G78 PILRA for 30 min on ice for receptor-ligand interactions. Cells were than stained with anti-mIgG2a (FITC). Binding of G78 PILRA to vector or ligand-transfected cells was analyzed by flow cytometry. Results are MFI of PILRA-mFC binding on ligand-transfected cells. Each shape is an independent experiment.
B, D, F) 293T cells were transfected with the expression construct of NPDC1 (B), HSV-1 gB (D), or myc-PIANP (F). 48 hrs. after the transfection cells were harvested and incubated with 50 μg/ml soluble mIgG2a-tagged variants of PILRA (G78 (AD risk), S80G, R72A, F76A and R78 (AD protective)) for 30 min on ice for receptor-ligand interactions. Cells were than stained with anti-mIgG2a (FITC). Binding of different PILRA variants to ligand-transfected cells was analyzed by flow cytometry. Results are MFI of PILRA-mFC binding on NPDC1-transfected cells. Each shape is an independent experiment.
C, E, G) Transfected cells were also stained with anti-hNPDC1 (C), anti-HSV-1 gB (E), or anti-myc (G) antibodies, followed by appropriate APC-conjugated secondary antibodies, to check the expression of PILRA ligands by flow cytometry.
(TIF)

S5 Fig C4A is a high affinity ligand for PILRA. A,B) 293T cells were transfected with putative ligands of PILRA (SORCS ECD, APLP1 ECD or full length C4A, fused with C-terminal gD tag and GPI anchor) and full length NPDC1 as positive control. 48 hrs post transfection, cells were harvested and incubated with soluble mIgG2a-tagged G78 PILRA (50 μg/ml) for 30 min on ice for receptor-ligand interactions. Cells were then stained with anti-mIgG2a (FITC). Binding of G78 PILRA to ligand-transfected cells was analyzed by flow cytometry. (A) representative images shown. (B) Results are fold increase in binding of each putative ligand compared to vector control for each experiment. Each shape is an independent experiment.
C) 293T cells were transfected with full length C4A fused with C-terminal gD tag and GPI anchor. 48 hrs post transfection, cells were harvested and incubated with soluble mIgG2a-tagged variants of PILRA (50 μg/ml) for 30 min on ice for receptor-ligand interactions. Cells were then stained with anti-mIgG2a (FITC). Binding of different PILRA variants to ligand-transfected cells was analyzed by flow cytometry. Results are the percentage of MFI of PILRA-mFc binding on ligand-transfected cells considering the G78 (AD risk) PILRA binding as 100% for each experiment. Each shape is an independent experiment.
(TIF)

S6 Fig PILRA G78R alters conformation of sialic acid-binding pocket. A,B) 293T cells were transfected with various constructs of PILRA (G78 (AD risk), R78 (AD protective), Q140A and S141A). 48 hrs. after the transfection cells were harvested and incubated with soluble mIgG2a-tagged ligand (NPDC1-mFc 50 μg/ml) for 30 min on ice for receptor-ligand interactions. Cells were than stained with anti-PILRA (APC) and anti-mIgG2a (FITC). Binding of NPDC1 to different PILRA variant transfected cells was analyzed by flow cytometry by gating double-positive cells (A) (representative images shown). Results are the mean percentage of different PILRA variant-transfected cells binding to NPDC1-mFC (B). Each shape is an independent experiment.
C) Comparison of binding affinities of different mIgG2a-tagged variants of PILRA to NPDC1-His by Surface Plasmon Resonance (SPR).
(TIF)

S7 Fig G78R PILRA variant reduces cytopathic effect of HSV-1 infection. Macrophages differentiated from healthy genotyped human monocytes were infected with 0.1 MOI of HSV-1 virus for 18 hrs. Cells were then fixed with 4% paraformaldehyde for 20 min, washed with PBS and stained with DAPI. Brightfield and fluorescent images were taken on an inverted microscope at 4X and 10X magnifications. R78 (AD protective) donors have less cytopathic effect as compared to G78 (AD risk) donors after 18 hrs of HSV-1 infection.
(TIF)

S8 Fig G78R PILRA variant does not protect against HSV-1-induced cytoxicity if viral exposure is prolonged. Macrophages differentiated from healthy genotyped human monocytes were infected with 0.01, 0.1, 1, and 10 MOI of HSV1 virus for 6, 18, 36 hrs.
A) LDH cytotoxicity assay was performed on supernatants harvested from HSV-1-infected hMDMs after 36 hrs. Results are % cytotoxicity—ratio of LDH released in culture supernatant after infection to LDH from completely lysed cells using lysis buffer, with completely lysed cells (maximum LDH release) being 100% for each donor. Statistical analysis is two-tailed paired t-test (p values <0.05 = *) performed on five genotyped individual donor pairs. Each shape represents one donor pair. After 36 hrs of HSV-1 infection, homozygous R78 (AD protective) macrophages have no significant difference on cytotoxicity as compared to their homozygous G78 (AD risk) counterparts except at the lowest MOI tested of 0.01.
B) HSV-1 DNA was quantitated by qPCR on DNA extracted from HSV-1-infected hMDMs after 36 hrs. Results are % HSV-1 DNA normalized to GAPDH considering G78 (AD risk) donor as 100% for each donor pair. Statistical analysis is two-tailed unpaired t-test (p values <0.05 = *, <0.005 = **, <0.0005 = ***, <0.0001 = ****) performed on five genotyped individual donor pairs. 36 hrs post infection, hMDMs from homozygous R78 (AD protective) donors showed no significant difference in HSV-1 DNA concentration as compared to homozygous G78 (AD risk) donors.
C,D,E) HSV-1 DNA was quantitated by qPCR on DNA extracted from HSV-1-infected hMDMs after 6, 18, or 36 hrs. Results are HSV-1 DNA (C_t_ values) normalized to GAPDH (C_t_ values). Statistical analysis is two-tailed paired t-test (p values <0.05 = *, <0.005 = **, <0.0005 = ***, <0.0001 = ****) performed on five genotyped individual donor pairs. Homozygous R78 (AD protective) macrophages showed lower amounts of HSV-1 DNA at 6 hrs for all MOI and at 18 hrs for lower MOIs (0.01 and 0.1), compared to homozygous G78 (AD risk) macrophages. At 36 hrs of infection for all MOI tested, there was no significant difference between the two PILRA genotypes.
(TIF)

S9 Fig G78R reduces HSV-1 infection by inhibiting viral entry but not replication. Macrophages differentiated from healthy genotyped human monocytes were infected with 0.01, 0.1, 1, and 10 MOI of HSV-1 virus for 6, 18, or 36 hrs.
Viral titers in the culture supernatant of HSV-1-infected human macrophages were determined by plaque assay on Vero cells. Results are number of plaque forming units (PFU) per ml of supernatant collected from HSV-1-infected human macrophages from two pairs of donors after 6, 18 and 36 hrs of infection (experiment 2). Homozygous R78 (AD protective) supernatant contained reduced PFU, with all MOI at 6hrs and 18hrs as compared to homozygous G78 (AD risk) counterparts. At 36 hrs post infection, only lower MOI (0.01 and 0.1) still showed significant decrease in the number of PFU in hMDMs supernatants from R78 (AD protective) donors as compared to G78 (AD risk) donors.
(TIF)

S10 Fig Proposed model for protection from Alzheimer’s disease conferred by reduced PILRA ligand binding. (TIF)

S1 Table Variants in Linkage Disequilibrium (r^2^ > 0.90) with rs1476679 in European ancestry samples. (DOCX)

S2 Table Association of 7q21 variants with *PILRB* expression in whole blood in the GTEX dataset. (DOCX)

S3 Table Human proteins containing a putative PILRA binding motif. (DOCX)

S4 Table LDH cytotoxicity assay, 18 hrs post infection. (DOC)

S5 Table LDH cytotoxicity assay, 36 hrs post infection. (DOC)
