# Supplementary figures and images for: Paired Immunoglobulin-like Type 2 Receptor Alpha G78R variant alters ligand binding and confers protection to Alzheimer's disease
Source: PLoS Genet. 2018 Nov 2;14(11):e1007427. doi: 10.1371/journal.pgen.1007427 (PMC6235402; doi:10.1371/journal.pgen.1007427)

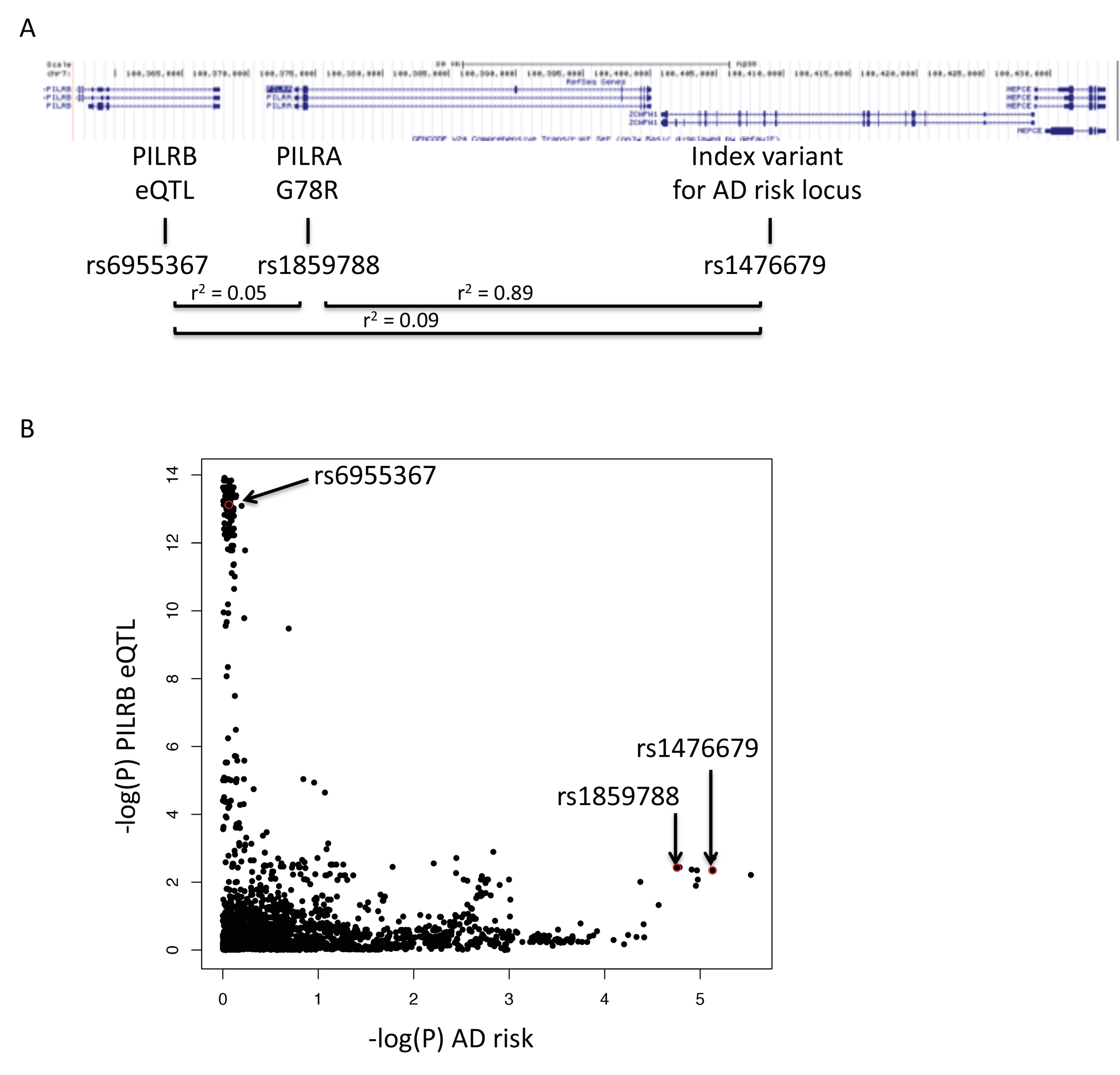

Supplement: S1 Fig — A) 7q21 AD risk locus. The genomic location and linkage relationship (r2) of the index variant for the AD risk (rs1476679), PILRA missense allele (G78R) and the PILRB eQTL (rs6955367). B) The AD risk variant does not co-localize with the PILRB eQTL. The association (-logP) of variants in the 7q21 region with mRNA levels in whole blood (GTEX data) is displayed along the y-axis, and association with AD risk (IGAP Phase 1 data) is shown along the x-axis. (TIF) [file pgen.1007427.s001.tif]

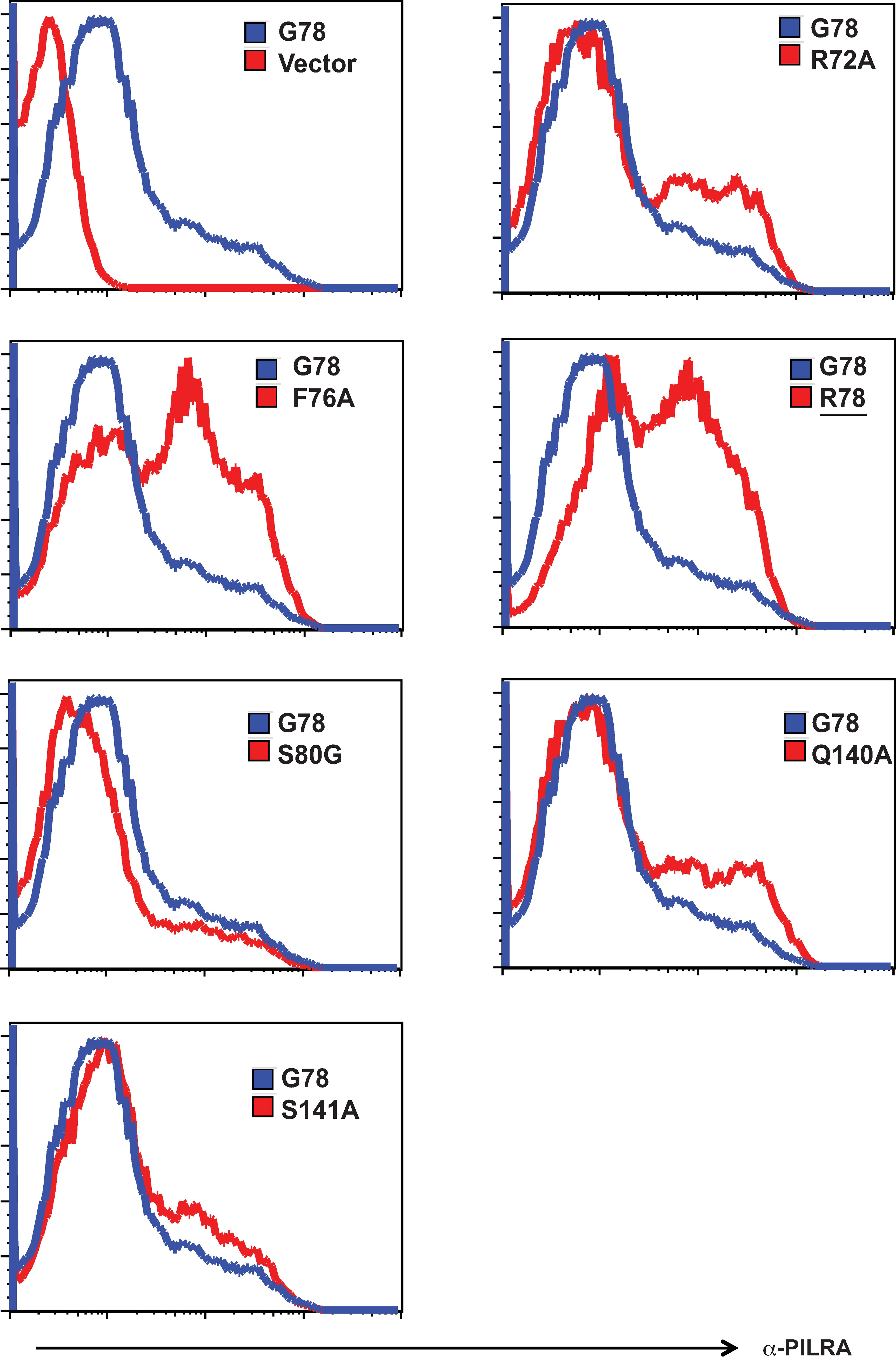

Supplement: S2 Fig — 293T cells were transfected with various constructs of PILRA (G78 (AD risk), R72A, F76A, R78 (AD protective), S80G, Q140A and S141A) and stained with anti-PILRA antibody followed by APC-conjugated anti-human IgG1 secondary to check the expression of PILRA by flow cytometry. (TIF) [file pgen.1007427.s002.tif]

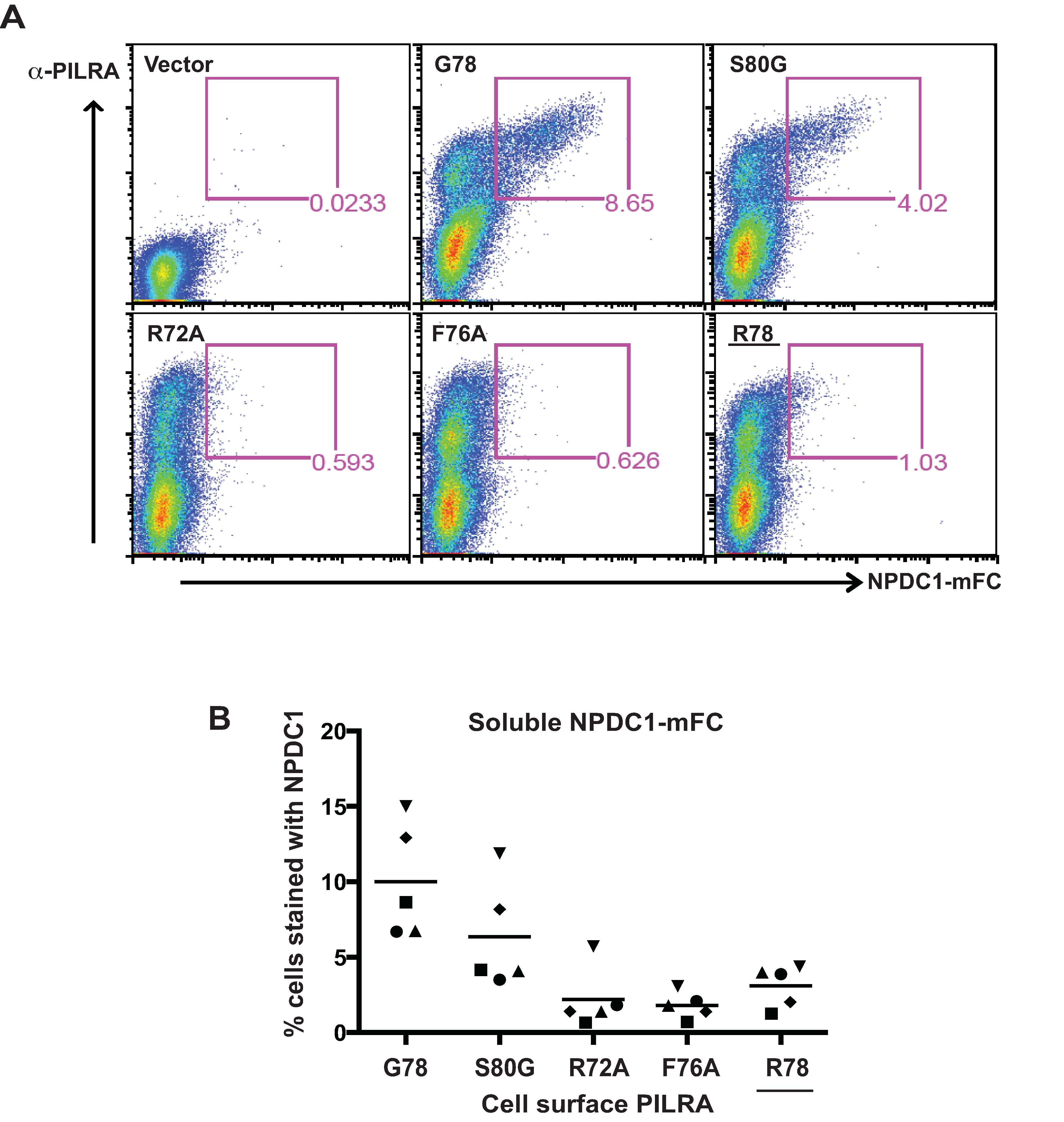

Supplement: S3 Fig — A,B) 293T cells were transfected with various constructs of PILRA (G78 (AD risk), S80G, R72A, F76A and R78 (AD protective)). 48 hrs. after the transfection cells were harvested and incubated with soluble mIgG2a tagged ligand (NPDC1-mFc, 50 μg/ml) for 30 min on ice for receptor-ligand interactions. Cells were than stained with anti-PILRA (APC) and anti-mIgG2a (FITC). (TIF) [file pgen.1007427.s003.tif]

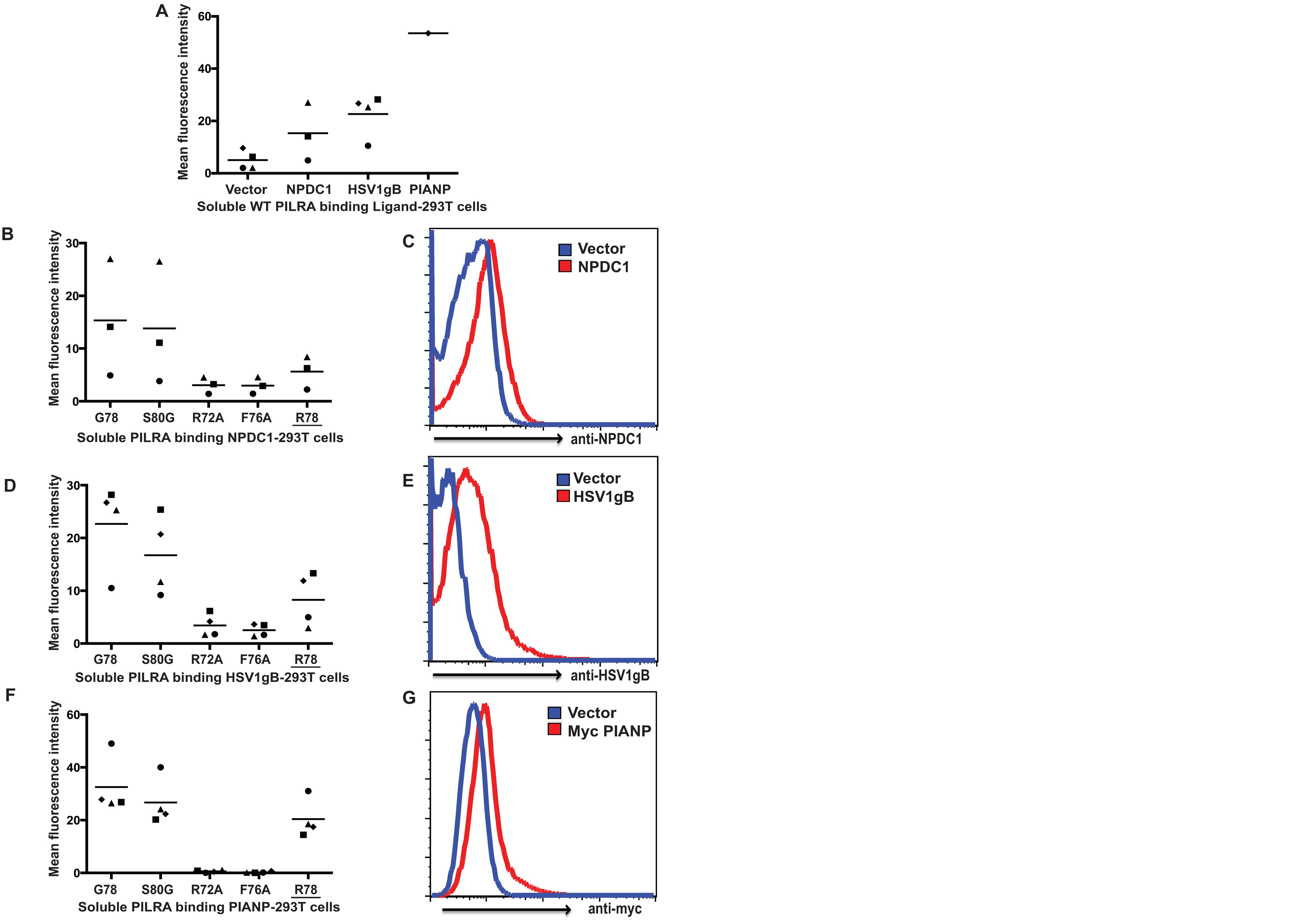

Supplement: S4 Fig — A) 293T cells were transfected with the expression vector or various PILRA ligand constructs (NPDC1, HSV1gB and PIANP). 48 hrs. after the transfection cells were harvested and incubated with 50 μg/ml soluble mIgG2a-tagged G78 PILRA for 30 min on ice for receptor-ligand interactions. Cells were than stained with anti-mIgG2a (FITC). Binding of G78 PILRA to vector or ligand-transfected cells was analyzed by flow cytometry. Results are MFI of PILRA-mFC binding on ligand-transfected cells. Each shape is an independent experiment. B, D, F) 293T cells were transfected with the expression construct of NPDC1 (B), HSV-1 gB (D), or myc-PIANP (F). 48 hrs. after the transfection cells were harvested and incubated with 50 μg/ml soluble mIgG2a-tagged variants of PILRA (G78 (AD risk), S80G, R72A, F76A and R78 (AD protective)) for 30 min on ice for receptor-ligand interactions. Cells were than stained with anti-mIgG2a (FITC). Binding of different PILRA variants to ligand-transfected cells was analyzed by flow cytometry. Results are MFI of PILRA-mFC binding on NPDC1-transfected cells. Each shape is an independent experiment. C, E, G) Transfected cells were also stained with anti-hNPDC1 (C), anti-HSV-1 gB (E), or anti-myc (G) antibodies, followed by appropriate APC-conjugated secondary antibodies, to check the expression of PILRA ligands by flow cytometry. (TIF) [file pgen.1007427.s004.tif]

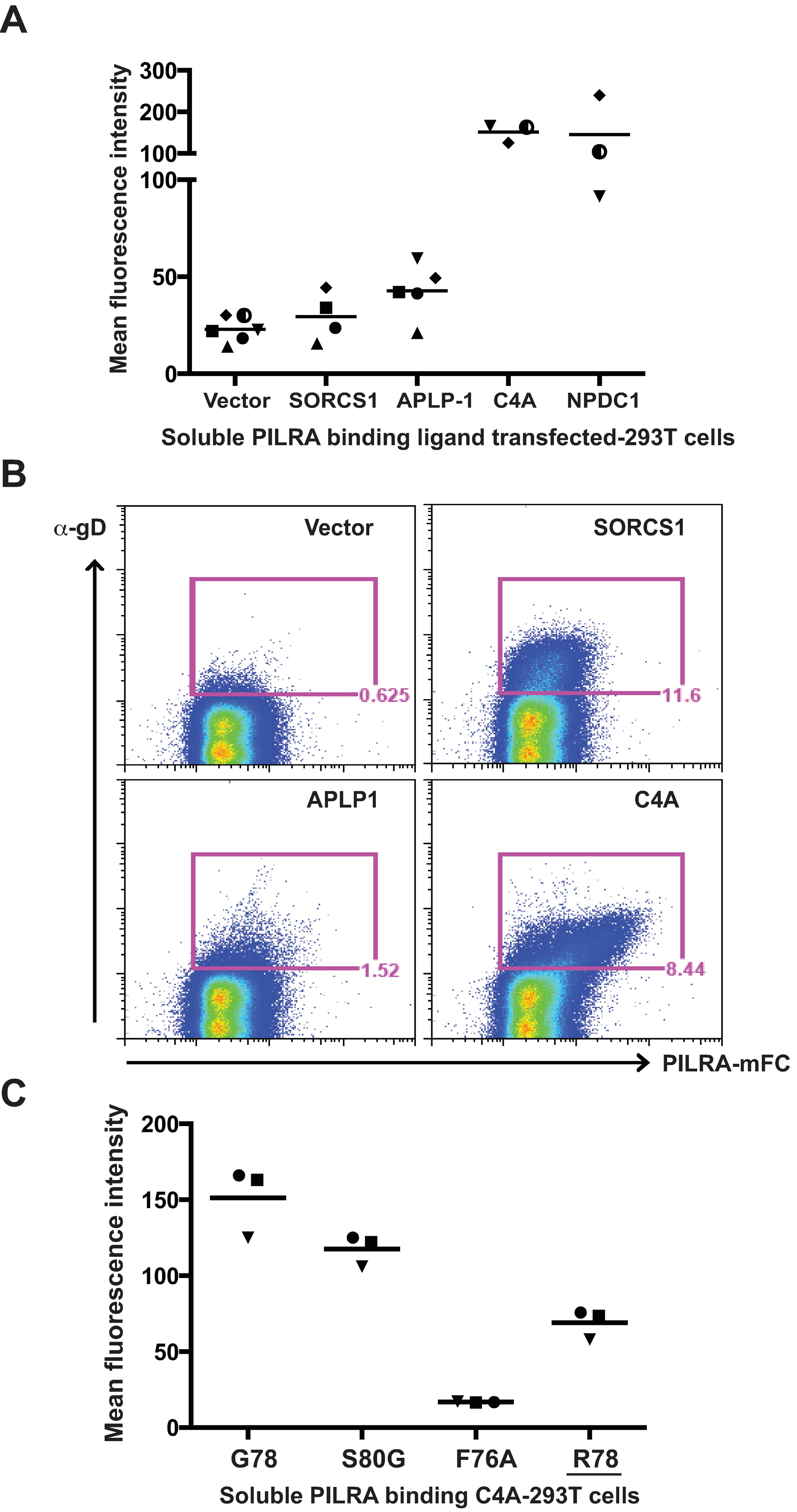

Supplement: S5 Fig — A,B) 293T cells were transfected with putative ligands of PILRA (SORCS ECD, APLP1 ECD or full length C4A, fused with C-terminal gD tag and GPI anchor) and full length NPDC1 as positive control. 48 hrs post transfection, cells were harvested and incubated with soluble mIgG2a-tagged G78 PILRA (50 μg/ml) for 30 min on ice for receptor-ligand interactions. Cells were then stained with anti-mIgG2a (FITC). Binding of G78 PILRA to ligand-transfected cells was analyzed by flow cytometry. (A) representative images shown. (B) Results are fold increase in binding of each putative ligand compared to vector control for each experiment. Each shape is an independent experiment. C) 293T cells were transfected with full length C4A fused with C-terminal gD tag and GPI anchor. 48 hrs post transfection, cells were harvested and incubated with soluble mIgG2a-tagged variants of PILRA (50 μg/ml) for 30 min on ice for receptor-ligand interactions. Cells were then stained with anti-mIgG2a (FITC). Binding of different PILRA variants to ligand-transfected cells was analyzed by flow cytometry. Results are the percentage of MFI of PILRA-mFc binding on ligand-transfected cells considering the G78 (AD risk) PILRA binding as 100% for each experiment. Each shape is an independent experiment. (TIF) [file pgen.1007427.s005.tif]

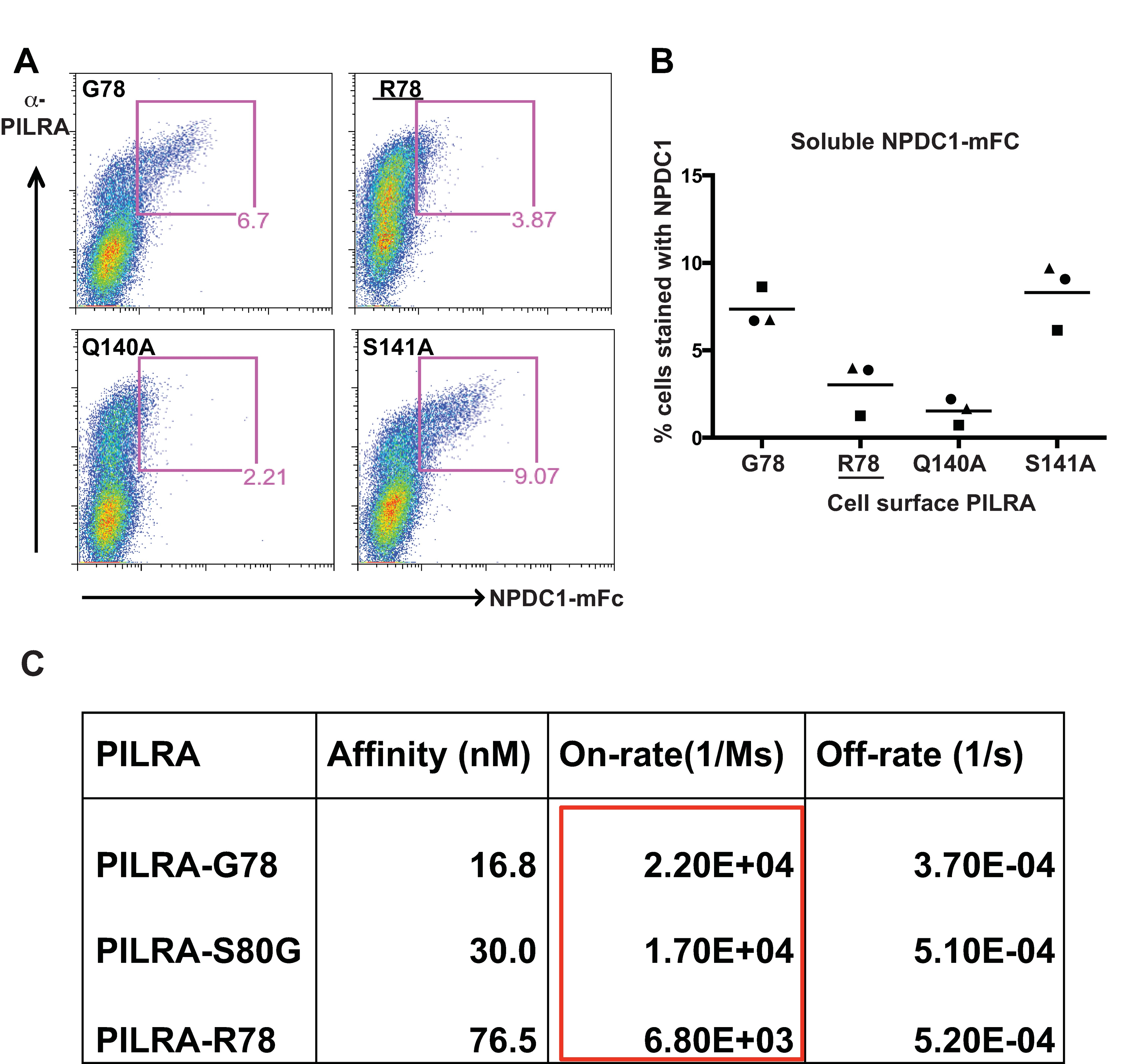

Supplement: S6 Fig — A,B) 293T cells were transfected with various constructs of PILRA (G78 (AD risk), R78 (AD protective), Q140A and S141A). 48 hrs. after the transfection cells were harvested and incubated with soluble mIgG2a-tagged ligand (NPDC1-mFc 50 μg/ml) for 30 min on ice for receptor-ligand interactions. Cells were than stained with anti-PILRA (APC) and anti-mIgG2a (FITC). Binding of NPDC1 to different PILRA variant transfected cells was analyzed by flow cytometry by gating double-positive cells (A) (representative images shown). Results are the mean percentage of different PILRA variant-transfected cells binding to NPDC1-mFC (B). Each shape is an independent experiment. C) Comparison of binding affinities of different mIgG2a-tagged variants of PILRA to NPDC1-His by Surface Plasmon Resonance (SPR). (TIF) [file pgen.1007427.s006.tif]

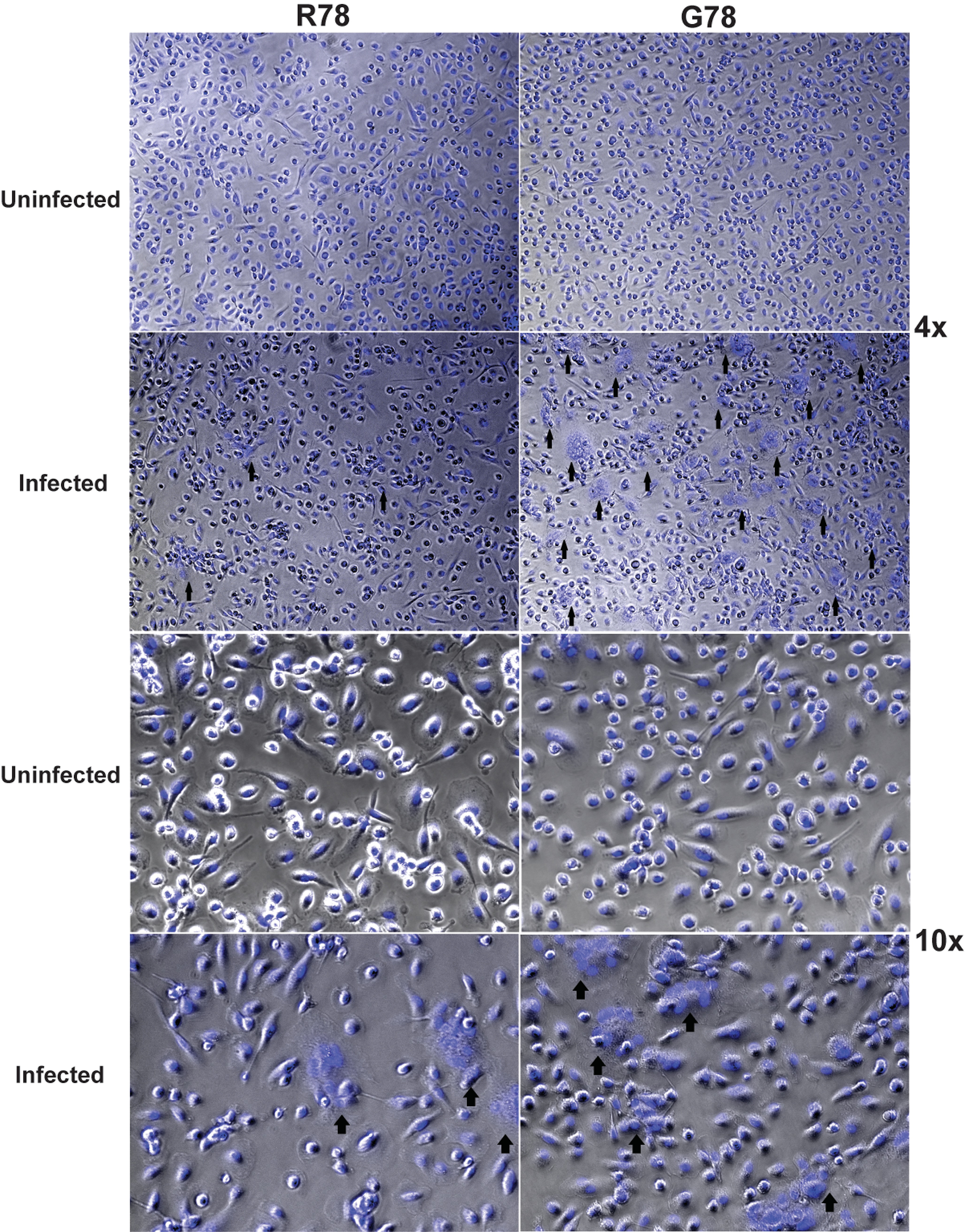

Supplement: S7 Fig — Macrophages differentiated from healthy genotyped human monocytes were infected with 0.1 MOI of HSV-1 virus for 18 hrs. Cells were then fixed with 4% paraformaldehyde for 20 min, washed with PBS and stained with DAPI. Brightfield and fluorescent images were taken on an inverted microscope at 4X and 10X magnifications. R78 (AD protective) donors have less cytopathic effect as compared to G78 (AD risk) donors after 18 hrs of HSV-1 infection. (TIF) [file pgen.1007427.s007.tif]

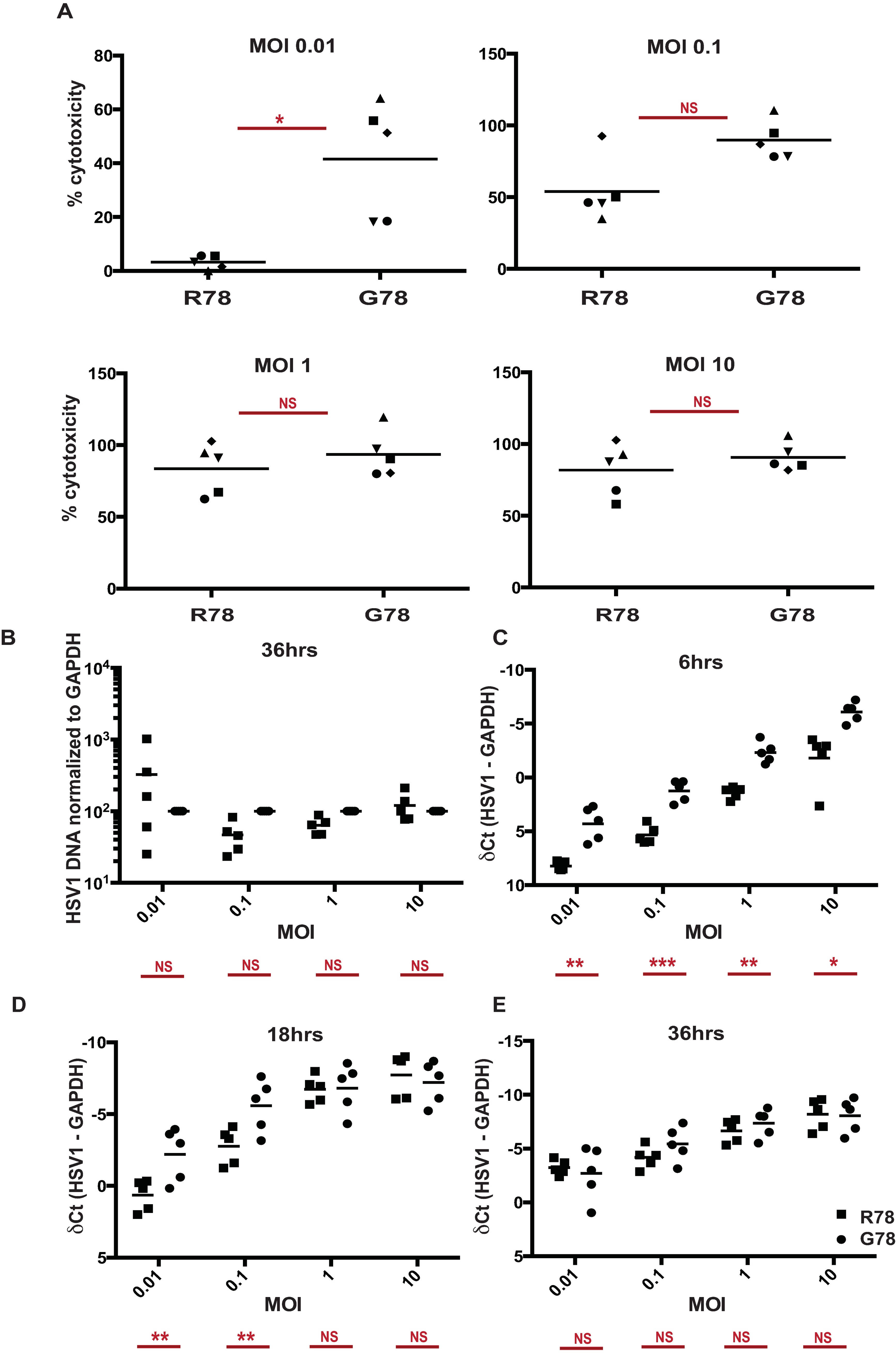

Supplement: S8 Fig — Macrophages differentiated from healthy genotyped human monocytes were infected with 0.01, 0.1, 1, and 10 MOI of HSV1 virus for 6, 18, 36 hrs. A) LDH cytotoxicity assay was performed on supernatants harvested from HSV-1-infected hMDMs after 36 hrs. Results are % cytotoxicity—ratio of LDH released in culture supernatant after infection to LDH from completely lysed cells using lysis buffer, with completely lysed cells (maximum LDH release) being 100% for each donor. Statistical analysis is two-tailed paired t-test (p values <0.05 = *) performed on five genotyped individual donor pairs. Each shape represents one donor pair. After 36 hrs of HSV-1 infection, homozygous R78 (AD protective) macrophages have no significant difference on cytotoxicity as compared to their homozygous G78 (AD risk) counterparts except at the lowest MOI tested of 0.01. B) HSV-1 DNA was quantitated by qPCR on DNA extracted from HSV-1-infected hMDMs after 36 hrs. Results are % HSV-1 DNA normalized to GAPDH considering G78 (AD risk) donor as 100% for each donor pair. Statistical analysis is two-tailed unpaired t-test (p values <0.05 = *, <0.005 = **, <0.0005 = ***, <0.0001 = ****) performed on five genotyped individual donor pairs. 36 hrs post infection, hMDMs from homozygous R78 (AD protective) donors showed no significant difference in HSV-1 DNA concentration as compared to homozygous G78 (AD risk) donors. C,D,E) HSV-1 DNA was quantitated by qPCR on DNA extracted from HSV-1-infected hMDMs after 6, 18, or 36 hrs. Results are HSV-1 DNA (Ct values) normalized to GAPDH (Ct values). Statistical analysis is two-tailed paired t-test (p values <0.05 = *, <0.005 = **, <0.0005 = ***, <0.0001 = ****) performed on five genotyped individual donor pairs. Homozygous R78 (AD protective) macrophages showed lower amounts of HSV-1 DNA at 6 hrs for all MOI and at 18 hrs for lower MOIs (0.01 and 0.1), compared to homozygous G78 (AD risk) macrophages. At 36 hrs of infection for all MOI tested, there was no significant [file pgen.1007427.s008.tif]

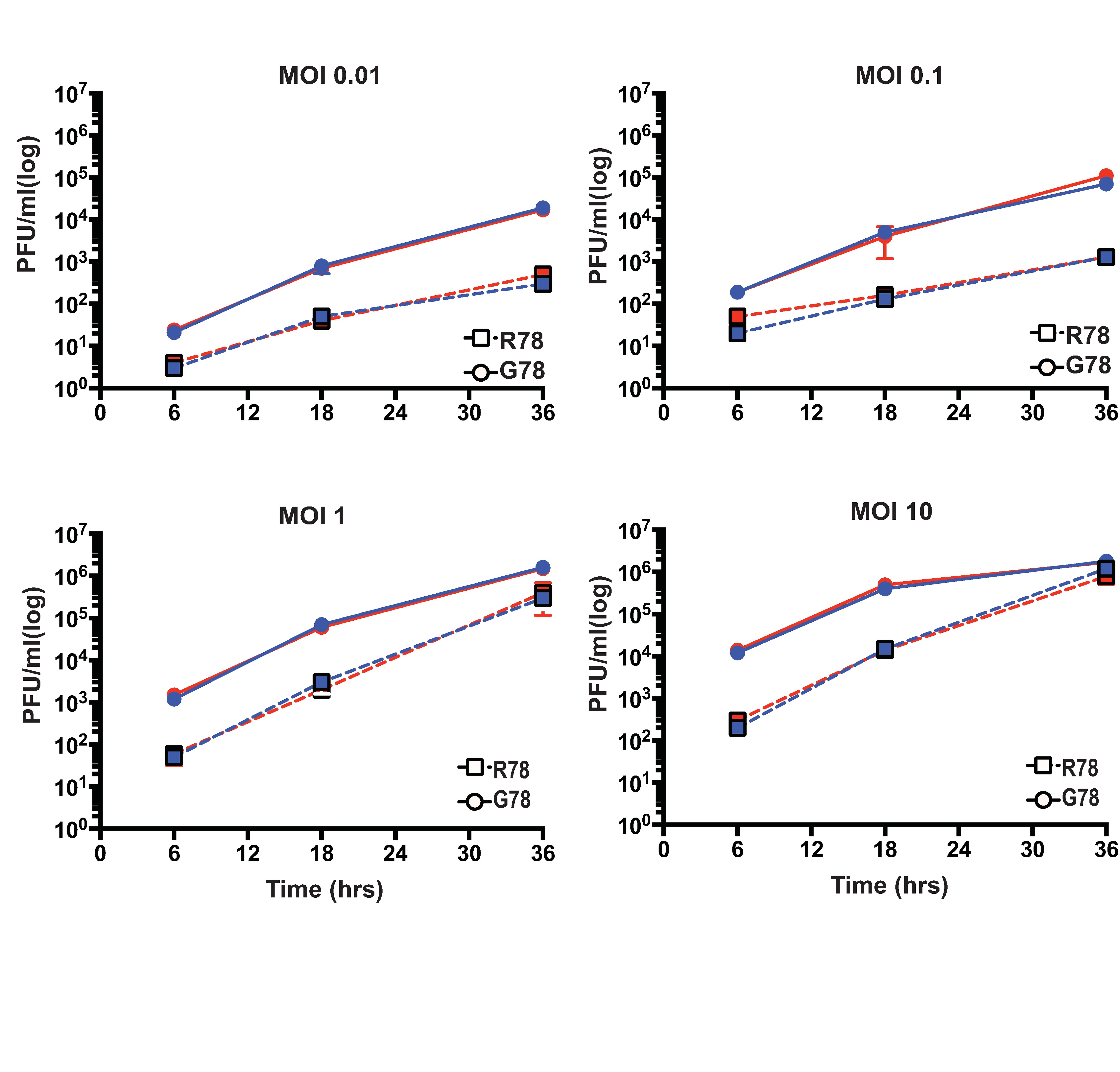

Supplement: S9 Fig — Macrophages differentiated from healthy genotyped human monocytes were infected with 0.01, 0.1, 1, and 10 MOI of HSV-1 virus for 6, 18, or 36 hrs. Viral titers in the culture supernatant of HSV-1-infected human macrophages were determined by plaque assay on Vero cells. Results are number of plaque forming units (PFU) per ml of supernatant collected from HSV-1-infected human macrophages from two pairs of donors after 6, 18 and 36 hrs of infection (experiment 2). Homozygous R78 (AD protective) supernatant contained reduced PFU, with all MOI at 6hrs and 18hrs as compared to homozygous G78 (AD risk) counterparts. At 36 hrs post infection, only lower MOI (0.01 and 0.1) still showed significant decrease in the number of PFU in hMDMs supernatants from R78 (AD protective) donors as compared to G78 (AD risk) donors. (TIF) [file pgen.1007427.s009.tif]

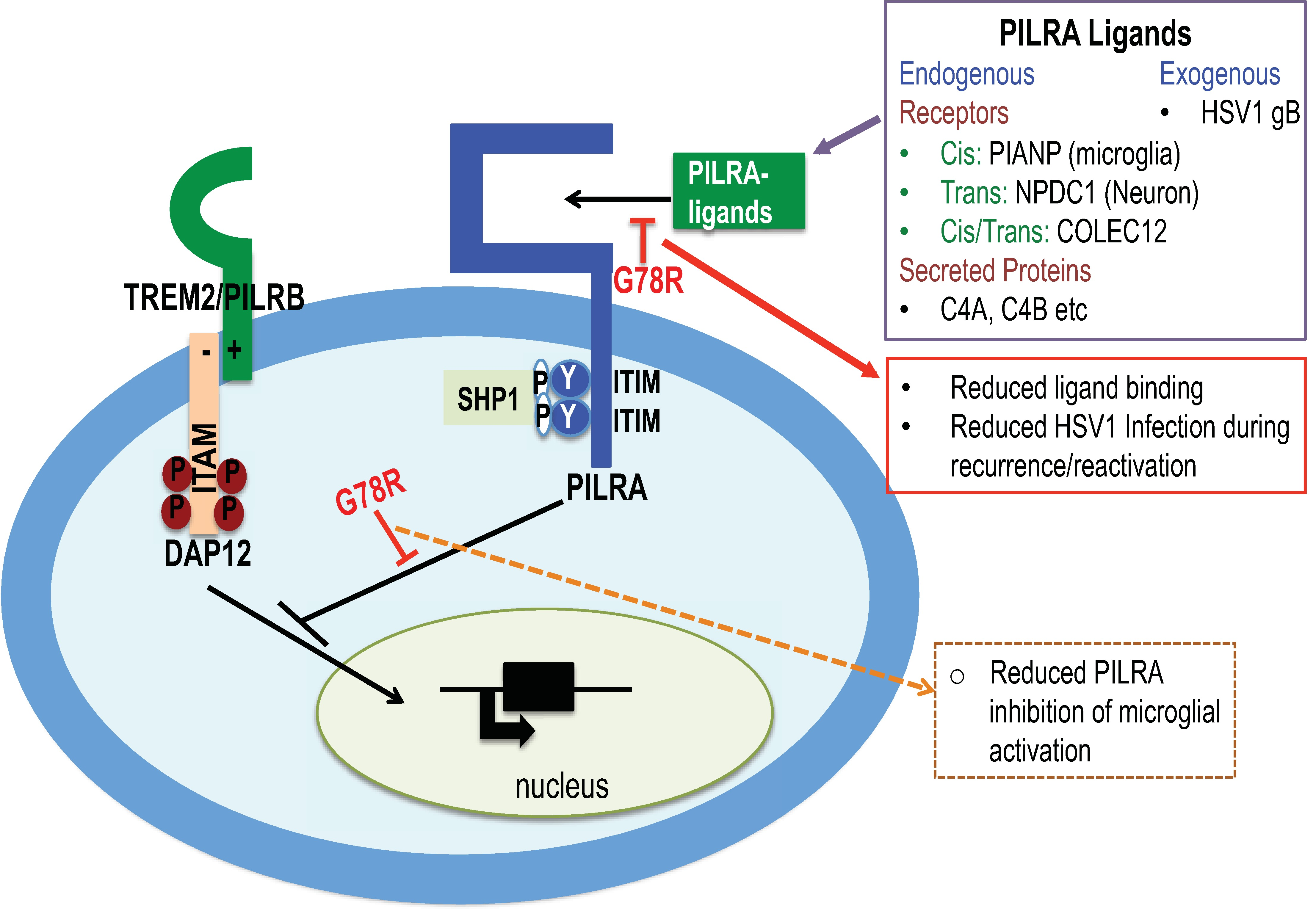

Supplement: S10 Fig — (TIF) [file pgen.1007427.s010.tif]
