# Supplementary material for: Paired Immunoglobulin-like Type 2 Receptor Alpha G78R variant alters ligand binding and confers protection to Alzheimer's disease
Source: PLoS Genet. 2018 Nov 2;14(11):e1007427. doi: 10.1371/journal.pgen.1007427 (PMC6235402; doi:10.1371/journal.pgen.1007427)
Supplement: S1 Table — (DOCX) [file pgen.1007427.s011.docx]

**Table S1**

|  |  |  | r^2^ to rs1476679 | | |
| --- | --- | --- | --- | --- | --- |
| Variant | Chr: position (HG19) | Annotation | CEU | GBR | GNE |
| rs34919929 | 7:100,012,334 | Intronic (*ZCWPW1*) | 1 | 1 | 1 |
| rs60738304 | 7:100,012,579 | Intronic (*ZCWPW1*) | 1 | 1 | 0.97 |
| rs34995835 | 7:99,990,364 | Intronic (*PILRA*) | 0.98 | 1 | 0.97 |
| rs1859788 | 7:99,971,834 | G78R (*PILRA*) | 0.93 | 0.93 | 0.89 |
| rs2405442 | 7:99,971,313 | Synonymous (*PILRA*) | 0.93 | 0.95 | 0.88 |
| rs2906657 | 7:99,984,089 | Intronic (*PILRA*) | 0.91 | 0.87 | NA |
