# Supplementary material for: Paired Immunoglobulin-like Type 2 Receptor Alpha G78R variant alters ligand binding and confers protection to Alzheimer's disease
Source: PLoS Genet. 2018 Nov 2;14(11):e1007427. doi: 10.1371/journal.pgen.1007427 (PMC6235402; doi:10.1371/journal.pgen.1007427)
Supplement: S2 Table — (DOCX) [file pgen.1007427.s012.docx]

**Table S2**

| SNP | P value for *PILRB* expression in whole blood | P value conditional on rs6955367 |
| --- | --- | --- |
| rs6955367 | 7.63e-14 | NA |
| rs1859788 | 0.007564 | 0.442908 |
| rs1476679 | 0.00454 | 0.309146 |
