# Supplementary material for: Paired Immunoglobulin-like Type 2 Receptor Alpha G78R variant alters ligand binding and confers protection to Alzheimer's disease
Source: PLoS Genet. 2018 Nov 2;14(11):e1007427. doi: 10.1371/journal.pgen.1007427 (PMC6235402; doi:10.1371/journal.pgen.1007427)
Supplement: S3 Table — (DOCX) [file pgen.1007427.s013.docx]

| **uniprot ID** | **gene_name** | **gene_description** | **matching_seq** | **begin** | **end** |
| --- | --- | --- | --- | --- | --- |
| **A0AUZ9** | KANSL1L C2orf67 | KAT8 regulatory NSL complex subunit 1-like protein (MSL1v2) | PTPEP | 52 | 56 |
| **A0MZ66** | SHTN1 KIAA1598 | Shootin-1 (Shootin1) | PPTPEP | 535 | 540 |
| **A0PJY2** | FEZF1 FEZ ZNF312B | Fez family zinc finger protein 1 (Zinc finger protein 312B) | PTPGP | 461 | 465 |
| **A1KXE4** | FAM168B KIAA0280L MANI | Myelin-associated neurite-outgrowth inhibitor (Mani) (p20) | PTPVAP | 167 | 172 |
| **A1L453** | PRSS38 MPN2 | Serine protease 38 (EC 3.4.21.-) (Marapsin-2) | PTPAQP | 296 | 301 |
| **A1L4H1** | SSC5D | Soluble scavenger receptor cysteine-rich domain-containing protein SSC5D (Soluble scavenger protein with 5 SRCR domains) (SSc5D) | PPTPSP | 1004 | 1009 |
| **A2A3N6** | PIPSL PSMD4P2 | Putative PIP5K1A and PSMD4-like protein (PIP5K1A-PSMD4) | PTPDP | 474 | 478 |
| **A2RUQ5** | C17orf102 | Uncharacterized protein C17orf102 | PTPTP | 72 | 76 |
| **A2VEC9** | SSPO KIAA2036 | SCO-spondin | PVTPATP | 2370 | 2376 |
| **A4D2P6** | GRID2IP | Delphilin (Glutamate receptor, ionotropic, delta 2-interacting protein 1) | PTPEP | 487 | 491 |
| **A6NCI8** | C2orf78 | Uncharacterized protein C2orf78 | PATPAQP | 780 | 786 |
| **A6ND36** | FAM83G PAWS1 | Protein FAM83G (Protein associated with SMAD1) | PTPPP | 641 | 645 |
| **A6NDK9** | GOLGA6C | Golgin subfamily A member 6C | PTPNIP | 517 | 522 |
| **A6NDN3** | GOLGA6B GOLGA | Golgin subfamily A member 6B | PTPNIP | 517 | 522 |
| **A6NE01** | FAM186A | Protein FAM186A | PPTPGQP | 1831 | 1837 |
| **A6NEH8** | ZNF503-AS2 C10orf41 NCRNA00245 | Putative uncharacterized protein encoded by ZNF503-AS2 (ZNF503 antisense RNA 2) | PTPRP | 66 | 70 |
| **A6NF01** | POM121B | Putative nuclear envelope pore membrane protein POM 121B | PTPPGP | 327 | 332 |
| **A6NGB9** | WIPF3 CR16 | WAS/WASL-interacting protein family member 3 (Corticosteroids and regional expression protein 16 homolog) | PPTPPPP | 176 | 182 |
| **A6NGW2** | STRCP1 STRCP | Putative stereocilin-like protein (Stereocilin pseudogene 1) | PPTPTQP | 153 | 159 |
| **A6NHQ4** | C17orf96 | Uncharacterized protein C17orf96 | PTPRKP | 23 | 28 |
| **A6NIE9** | PRSS29P ISP2 | Putative serine protease 29 (EC 3.4.21.-) (Implantation serine proteinase 2-like protein) (ISP2-like protein) | PTTPDP | 2 | 7 |
| **A6NIK2** | LRRC10B | Leucine-rich repeat-containing protein 10B | PTPRPP | 243 | 248 |
| **A6NJT0** | UNCX UNCX4.1 | Homeobox protein unc-4 homolog (Homeobox protein Uncx4.1) | PTPLLP | 71 | 76 |
| **A6NLJ0** | C2CD4B FAM148B NLF2 | C2 calcium-dependent domain-containing protein 4B (Nuclear-localized factor 2) (Protein FAM148B) | PATPAAP | 151 | 157 |
| **A6NMB9** | FIGNL2 | Putative fidgetin-like protein 2 | PTPLP | 238 | 242 |
| **A6NNE9** | 42440 | E3 ubiquitin-protein ligase MARCH11 (EC 6.3.2.-) (Membrane-associated RING finger protein 11) (Membrane-associated RING-CH protein XI) (MARCH-XI) | PPTPPP | 31 | 36 |
| **A6NNW6** | ENO4 C10orf134 | Enolase 4 (EC 4.2.1.11) (2-phospho-D-glycerate hydro-lyase) | PTPLPP | 189 | 194 |
| **A7XYQ1** | SOBP JXC1 | Sine oculis-binding protein homolog (Jackson circler protein 1) | PTPVP | 346 | 350 |
| **A8CG34** | POM121C | Nuclear envelope pore membrane protein POM 121C (Nuclear pore membrane protein 121-2) (POM121-2) (Pore membrane protein of 121 kDa C) | PTPPGP | 720 | 725 |
| **A8K7I4** | CLCA1 CACC1 | Calcium-activated chloride channel regulator 1 (EC 3.4.-.-) (Calcium-activated chloride channel family member 1) (hCLCA1) (Calcium-activated chloride channel protein 1) (CaCC-1) (hCaCC-1) | PETPSP | 872 | 877 |
| **A8MTQ0** | NOTO | Homeobox protein notochord | PNTPRAP | 36 | 42 |
| **A8MU93** | C17orf100 | Uncharacterized protein C17orf100 | PTPRP | 102 | 106 |
| **A8MV57** | MPTX1 MPTX | Putative mucosal pentraxin homolog | PTPCP | 18 | 22 |
| **B1AK53** | ESPN DFNB36 LP2654 | Espin (Autosomal recessive deafness type 36 protein) (Ectoplasmic specialization protein) | PTPPPP | 428 | 433 |
| **B2RXH4** | BTBD18 | BTB/POZ domain-containing protein 18 | PHTPLP | 163 | 168 |
| **B3EWF7** | EPM2A | Laforin, isoform 9 | PTPPP | 19 | 23 |
| **B4DYI2** | SPATA31C2 FAM75C2 | Spermatogenesis-associated protein 31C2 (Protein FAM75C2) | PRTPDP | 214 | 219 |
| **C9J069** | C9orf172 | Uncharacterized protein C9orf172 | PGTPALP | 827 | 833 |
| **C9JDV5** | C12orf77 | Putative uncharacterized protein C12orf77 | PATPVP | 79 | 84 |
| **C9JH25** | PRRT4 | Proline-rich transmembrane protein 4 | PTPSIP | 25 | 30 |
| **C9JUS6** | ADM5 C19orf76 | Putative adrenomedullin-5-like protein | PLTPAP | 136 | 141 |
| **E7EW31** | PROB1 C5orf65 | Proline-rich basic protein 1 | PPTPSAP | 548 | 554 |
| **E9PAV3** | NACA | Nascent polypeptide-associated complex subunit alpha, muscle-specific form (Alpha-NAC, muscle-specific form) (skNAC) | PTPPSP | 856 | 861 |
| **E9PI22** | PRR23D1 | Proline-rich protein 23D1 | PPTPSP | 218 | 223 |
| **F2Z333** | C1orf233 | Fibronectin type-III domain-containing transmembrane protein C1orf233 | PTPDAP | 30 | 35 |
| **H3BV60** | TGFBR3L | Transforming growth factor-beta receptor type 3-like protein (TGF-beta receptor type-3-like protein) (TGFR-3L) (Transforming growth factor-beta receptor type III-like protein) (TGF-beta receptor type III-like protein) | PLTPPPP | 188 | 194 |
| **H7BZ55** | CROCC2 | Putative ciliary rootlet coiled-coil protein 2 | PTPVP | 55 | 59 |
| **I3L273** | GFY | Golgi-associated olfactory signaling regulator (Protein Goofy) | PGTPYP | 62 | 67 |
| **O00139** | KIF2A KIF2 KNS2 | Kinesin-like protein KIF2A (Kinesin-2) (hK2) | PETPPPP | 76 | 82 |
| **O00159** | MYO1C | Unconventional myosin-Ic (Myosin I beta) (MMI-beta) (MMIb) | PTPPP | 834 | 838 |
| **O00238** | BMPR1B | Bone morphogenetic protein receptor type-1B (BMP type-1B receptor) (BMPR-1B) (EC 2.7.11.30) (CD antigen CDw293) | PTPRP | 23 | 27 |
| **O00255** | MEN1 SCG2 | Menin | PTPGRP | 296 | 301 |
| **O00267** | SUPT5H SPT5 SPT5H | Transcription elongation factor SPT5 (hSPT5) (DRB sensitivity-inducing factor 160 kDa subunit) (DSIF p160) (DRB sensitivity-inducing factor large subunit) (DSIF large subunit) (Tat-cotransactivator 1 protein) (Tat-CT1 protein) | PTPSP | 844 | 848 |
| **O00303** | EIF3F EIF3S5 | Eukaryotic translation initiation factor 3 subunit F (eIF3f) (Deubiquitinating enzyme eIF3f) (EC 3.4.19.12) (Eukaryotic translation initiation factor 3 subunit 5) (eIF-3-epsilon) (eIF3 p47) | PATPTP | 12 | 17 |
| **O00330** | PDHX PDX1 | Pyruvate dehydrogenase protein X component, mitochondrial (Dihydrolipoamide dehydrogenase-binding protein of pyruvate dehydrogenase complex) (E3-binding protein) (E3BP) (Lipoyl-containing pyruvate dehydrogenase complex component X) (proX) | PTPAP | 233 | 237 |
| **O00401** | WASL | Neural Wiskott-Aldrich syndrome protein (N-WASP) | PPTPAP | 458 | 463 |
| **O00443** | PIK3C2A | Phosphatidylinositol 4-phosphate 3-kinase C2 domain-containing subunit alpha (PI3K-C2-alpha) (PtdIns-3-kinase C2 subunit alpha) (EC 2.7.1.154) (Phosphoinositide 3-kinase-C2-alpha) | PLTPATP | 199 | 205 |
| **O00445** | SYT5 | Synaptotagmin-5 (Synaptotagmin V) (SytV) | PPTPGPP | 5 | 11 |
| **O00469** | PLOD2 | Procollagen-lysine,2-oxoglutarate 5-dioxygenase 2 (EC 1.14.11.4) (Lysyl hydroxylase 2) (LH2) | PTPFLP | 307 | 312 |
| **O00472** | ELL2 | RNA polymerase II elongation factor ELL2 | PTPPP | 373 | 377 |
| **O00512** | BCL9 | B-cell CLL/lymphoma 9 protein (B-cell lymphoma 9 protein) (Bcl-9) (Protein legless homolog) | PTPPIP | 256 | 261 |
| **O00555** | CACNA1A CACH4 CACN3 CACNL1A4 | Voltage-dependent P/Q-type calcium channel subunit alpha-1A (Brain calcium channel I) (BI) (Calcium channel, L type, alpha-1 polypeptide isoform 4) (Voltage-gated calcium channel subunit alpha Cav2.1) | PQTPSTP | 2288 | 2294 |
| **O00587** | MFNG | Beta-1,3-N-acetylglucosaminyltransferase manic fringe (EC 2.4.1.222) (O-fucosylpeptide 3-beta-N-acetylglucosaminyltransferase) | PDTPWCP | 310 | 316 |
| **O00592** | PODXL PCLP PCLP1 | Podocalyxin (GCTM-2 antigen) (Gp200) (Podocalyxin-like protein 1) (PC) (PCLP-1) | PKTPSP | 328 | 333 |
| **O00623** | PEX12 PAF3 | Peroxisome assembly protein 12 (Peroxin-12) (Peroxisome assembly factor 3) (PAF-3) | PTPPPP | 280 | 285 |
| **O00716** | E2F3 KIAA0075 | Transcription factor E2F3 (E2F-3) | PKTPKSP | 167 | 173 |
| **O14512** | SOCS7 NAP4 SOCS6 | Suppressor of cytokine signaling 7 (SOCS-7) (Nck, Ash and phospholipase C gamma-binding protein) (Nck-associated protein 4) (NAP-4) | PPTPPPP | 305 | 311 |
| **O14522** | PTPRT KIAA0283 | Receptor-type tyrosine-protein phosphatase T (R-PTP-T) (EC 3.1.3.48) (Receptor-type tyrosine-protein phosphatase rho) (RPTP-rho) | PPTPIAP | 288 | 294 |
| **O14523** | C2CD2L KIAA0285 TMEM24 DLNB23 | C2 domain-containing protein 2-like (Transmembrane protein 24) | PLTPGP | 386 | 391 |
| **O14525** | ASTN1 ASTN KIAA0289 | Astrotactin-1 | PTPDP | 792 | 796 |
| **O14526** | FCHO1 KIAA0290 | F-BAR domain only protein 1 | PGTPQSP | 496 | 502 |
| **O14529** | CUX2 CUTL2 KIAA0293 | Homeobox protein cut-like 2 (Homeobox protein cux-2) | PATPAP | 520 | 525 |
| **O14556** | GAPDHS GAPD2 GAPDH2 GAPDS HSD-35 HSD35 | Glyceraldehyde-3-phosphate dehydrogenase, testis-specific (EC 1.2.1.12) (Spermatogenic cell-specific glyceraldehyde 3-phosphate dehydrogenase 2) (GAPDH-2) (Spermatogenic glyceraldehyde-3-phosphate dehydrogenase) | PATPPP | 62 | 67 |
| **O14559** | ARHGAP33 SNX26 TCGAP | Rho GTPase-activating protein 33 (Rho-type GTPase-activating protein 33) (Sorting nexin-26) (Tc10/CDC42 GTPase-activating protein) | PTTPKAP | 562 | 568 |
| **O14576** | DYNC1I1 DNCI1 DNCIC1 | Cytoplasmic dynein 1 intermediate chain 1 (Cytoplasmic dynein intermediate chain 1) (Dynein intermediate chain 1, cytosolic) (DH IC-1) | PTPMSP | 93 | 98 |
| **O14593** | RFXANK ANKRA1 RFXB | DNA-binding protein RFXANK (Ankyrin repeat family A protein 1) (Regulatory factor X subunit B) (RFX-B) (Regulatory factor X-associated ankyrin-containing protein) | PCTPEP | 44 | 49 |
| **O14594** | NCAN CSPG3 NEUR | Neurocan core protein (Chondroitin sulfate proteoglycan 3) | PTPGDP | 431 | 436 |
| **O14598** | VCY BPY1 VCY1A; VCY1B BPY1B | Testis-specific basic protein Y 1 (Basic charge, Y-linked 1) (Variably charged protein Y) | PSTPLSP | 119 | 125 |
| **O14645** | DNALI1 | Axonemal dynein light intermediate polypeptide 1 (Inner dynein arm light chain, axonemal) (hp28) | PSTPCVP | 53 | 59 |
| **O14669** | PRRG2 PRGP2 TMG2 | Transmembrane gamma-carboxyglutamic acid protein 2 (Proline-rich gamma-carboxyglutamic acid protein 2) (Proline-rich Gla protein 2) | PPTPLPP | 161 | 167 |
| **O14686** | KMT2D ALR MLL2 MLL4 | Histone-lysine N-methyltransferase 2D (Lysine N-methyltransferase 2D) (EC 2.1.1.43) (ALL1-related protein) (Myeloid/lymphoid or mixed-lineage leukemia protein 2) | PSTPTTP | 1981 | 1987 |
| **O14763** | TNFRSF10B DR5 KILLER TRAILR2 TRICK2 ZTNFR9 UNQ160/PRO186 | Tumor necrosis factor receptor superfamily member 10B (Death receptor 5) (TNF-related apoptosis-inducing ligand receptor 2) (TRAIL receptor 2) (TRAIL-R2) (CD antigen CD262) | PGTPASP | 202 | 208 |
| **O14776** | TCERG1 CA150 TAF2S | Transcription elongation regulator 1 (TATA box-binding protein-associated factor 2S) (Transcription factor CA150) | PGTPALP | 125 | 131 |
| **O14798** | TNFRSF10C DCR1 LIT TRAILR3 TRID UNQ321/PRO366 | Tumor necrosis factor receptor superfamily member 10C (Antagonist decoy receptor for TRAIL/Apo-2L) (Decoy TRAIL receptor without death domain) (Decoy receptor 1) (DcR1) (Lymphocyte inhibitor of TRAIL) (TNF-related apoptosis-inducing ligand receptor 3) (TRAIL receptor 3) (TRAIL-R3) (TRAIL receptor without an intracellular domain) (CD antigen CD263) | PGTPAP | 172 | 177 |
| **O14836** | TNFRSF13B TACI | Tumor necrosis factor receptor superfamily member 13B (Transmembrane activator and CAML interactor) (CD antigen CD267) | PGTPDP | 248 | 253 |
| **O14939** | PLD2 | Phospholipase D2 (PLD 2) (hPLD2) (EC 3.1.4.4) (Choline phosphatase 2) (PLD1C) (Phosphatidylcholine-hydrolyzing phospholipase D2) | PPTPRP | 486 | 491 |
| **O14964** | HGS HRS | Hepatocyte growth factor-regulated tyrosine kinase substrate (Hrs) (Protein pp110) | PTPSAP | 346 | 351 |
| **O15013** | ARHGEF10 KIAA0294 | Rho guanine nucleotide exchange factor 10 | PPTPVP | 129 | 134 |
| **O15018** | PDZD2 AIPC KIAA0300 PDZK3 | PDZ domain-containing protein 2 (Activated in prostate cancer protein) (PDZ domain-containing protein 3) [Cleaved into: Processed PDZ domain-containing protein 2] | PTTPKSP | 2014 | 2020 |
| **O15021** | MAST4 KIAA0303 | Microtubule-associated serine/threonine-protein kinase 4 (EC 2.7.11.1) | PTPQP | 1388 | 1392 |
| **O15027** | SEC16A KIAA0310 SEC16 SEC16L | Protein transport protein Sec16A (SEC16 homolog A) | PSTPSPP | 413 | 419 |
| **O15034** | RIMBP2 KIAA0318 RBP2 | RIMS-binding protein 2 (RIM-BP2) | PPTPHP | 588 | 593 |
| **O15047** | SETD1A KIAA0339 KMT2F SET1 SET1A | Histone-lysine N-methyltransferase SETD1A (EC 2.1.1.43) (Lysine N-methyltransferase 2F) (SET domain-containing protein 1A) (hSET1A) (Set1/Ash2 histone methyltransferase complex subunit SET1) | PCTPPP | 538 | 543 |
| **O15049** | N4BP3 KIAA0341 | NEDD4-binding protein 3 (N4BP3) | PPTPWSP | 531 | 537 |
| **O15054** | KDM6B JMJD3 KIAA0346 | Lysine-specific demethylase 6B (EC 1.14.11.-) (JmjC domain-containing protein 3) (Jumonji domain-containing protein 3) (Lysine demethylase 6B) | PTPRP | 81 | 85 |
| **O15056** | SYNJ2 KIAA0348 | Synaptojanin-2 (EC 3.1.3.36) (Synaptic inositol 1,4,5-trisphosphate 5-phosphatase 2) | PETPQAP | 1218 | 1224 |
| **O15063** | KIAA0355 | Uncharacterized protein KIAA0355 | PLTPQP | 710 | 715 |
| **O15067** | PFAS KIAA0361 | Phosphoribosylformylglycinamidine synthase (FGAM synthase) (FGAMS) (EC 6.3.5.3) (Formylglycinamide ribonucleotide amidotransferase) (FGAR amidotransferase) (FGAR-AT) (Formylglycinamide ribotide amidotransferase) | PPTPLP | 617 | 622 |
| **O15078** | CEP290 BBS14 KIAA0373 NPHP6 | Centrosomal protein of 290 kDa (Cep290) (Bardet-Biedl syndrome 14 protein) (Cancer/testis antigen 87) (CT87) (Nephrocystin-6) (Tumor antigen se2-2) | PTPVP | 1611 | 1615 |
| **O15079** | SNPH KIAA0374 | Syntaphilin | PTPQRP | 383 | 388 |
| **O15146** | MUSK | Muscle, skeletal receptor tyrosine-protein kinase (EC 2.7.10.1) (Muscle-specific tyrosine-protein kinase receptor) (MuSK) (Muscle-specific kinase receptor) | PTPIP | 388 | 392 |
| **O15209** | ZBTB22 BING1 ZBTB22A ZNF297 | Zinc finger and BTB domain-containing protein 22 (Protein BING1) (Zinc finger and BTB domain-containing protein 22A) (Zinc finger protein 297) | PTPLVP | 309 | 314 |
| **O15211** | RGL2 RAB2L | Ral guanine nucleotide dissociation stimulator-like 2 (RalGDS-like 2) (RalGDS-like factor) (Ras-associated protein RAB2L) | PTTPAP | 560 | 565 |
| **O15265** | ATXN7 SCA7 | Ataxin-7 (Spinocerebellar ataxia type 7 protein) | PHTPSLP | 447 | 453 |
| **O15357** | INPPL1 SHIP2 | Phosphatidylinositol 3,4,5-trisphosphate 5-phosphatase 2 (EC 3.1.3.86) (Inositol polyphosphate phosphatase-like protein 1) (INPPL-1) (Protein 51C) (SH2 domain-containing inositol 5'-phosphatase 2) (SH2 domain-containing inositol phosphatase 2) (SHIP-2) | PETPTAP | 163 | 169 |
| **O15405** | TOX3 CAGF9 TNRC9 | TOX high mobility group box family member 3 (CAG trinucleotide repeat-containing gene F9 protein) (Trinucleotide repeat-containing gene 9 protein) | PITPPP | 68 | 73 |
| **O15409** | FOXP2 CAGH44 TNRC10 | Forkhead box protein P2 (CAG repeat protein 44) (Trinucleotide repeat-containing gene 10 protein) | PQTPTTP | 444 | 450 |
| **O15417** | TNRC18 CAGL79 KIAA1856 | Trinucleotide repeat-containing gene 18 protein (Long CAG trinucleotide repeat-containing gene 79 protein) | PLTPAP | 2144 | 2149 |
| **O15528** | CYP27B1 CYP1ALPHA CYP27B | 25-hydroxyvitamin D-1 alpha hydroxylase, mitochondrial (EC 1.14.13.13) (25-OHD-1 alpha-hydroxylase) (25-hydroxyvitamin D(3) 1-alpha-hydroxylase) (VD3 1A hydroxylase) (Calcidiol 1-monooxygenase) (Cytochrome P450 subfamily XXVIIB polypeptide 1) (Cytochrome P450C1 alpha) (Cytochrome P450VD1-alpha) (Cytochrome p450 27B1) | PTPHP | 438 | 442 |
| **O15534** | PER1 KIAA0482 PER RIGUI | Period circadian protein homolog 1 (hPER1) (Circadian clock protein PERIOD 1) (Circadian pacemaker protein Rigui) | PSTPWP | 864 | 869 |
| **O43150** | ASAP2 DDEF2 KIAA0400 | Arf-GAP with SH3 domain, ANK repeat and PH domain-containing protein 2 (Development and differentiation-enhancing factor 2) (Paxillin-associated protein with ARF GAP activity 3) (PAG3) (Pyk2 C-terminus-associated protein) (PAP) | PLTPTPP | 841 | 847 |
| **O43182** | ARHGAP6 RHOGAP6 | Rho GTPase-activating protein 6 (Rho-type GTPase-activating protein 6) (Rho-type GTPase-activating protein RhoGAPX-1) | PNTPEP | 347 | 352 |
| **O43281** | EFS CASS3 | Embryonal Fyn-associated substrate (hEFS) (Cas scaffolding protein family member 3) | PLTPKPP | 191 | 197 |
| **O43294** | TGFB1I1 ARA55 | Transforming growth factor beta-1-induced transcript 1 protein (Androgen receptor coactivator 55 kDa protein) (Androgen receptor-associated protein of 55 kDa) (Hydrogen peroxide-inducible clone 5 protein) (Hic-5) | PLTPPP | 31 | 36 |
| **O43306** | ADCY6 KIAA0422 | Adenylate cyclase type 6 (EC 4.6.1.1) (ATP pyrophosphate-lyase 6) (Adenylate cyclase type VI) (Adenylyl cyclase 6) (Ca(2+)-inhibitable adenylyl cyclase) | PTPAGP | 55 | 60 |
| **O43365** | HOXA3 HOX1E | Homeobox protein Hox-A3 (Homeobox protein Hox-1E) | PTPAAP | 114 | 119 |
| **O43395** | PRPF3 HPRP3 PRP3 | U4/U6 small nuclear ribonucleoprotein Prp3 (Pre-mRNA-splicing factor 3) (hPrp3) (U4/U6 snRNP 90 kDa protein) | PPTPQP | 165 | 170 |
| **O43426** | SYNJ1 KIAA0910 | Synaptojanin-1 (EC 3.1.3.36) (Synaptic inositol 1,4,5-trisphosphate 5-phosphatase 1) | PATPLP | 1090 | 1095 |
| **O43432** | EIF4G3 | Eukaryotic translation initiation factor 4 gamma 3 (eIF-4-gamma 3) (eIF-4G 3) (eIF4G 3) (eIF-4-gamma II) (eIF4GII) | PPTPPTP | 415 | 421 |
| **O43439** | CBFA2T2 EHT MTGR1 | Protein CBFA2T2 (ETO homologous on chromosome 20) (MTG8-like protein) (MTG8-related protein 1) (Myeloid translocation-related protein 1) (p85) | PTPPP | 286 | 290 |
| **O43451** | MGAM MGA MGAML | Maltase-glucoamylase, intestinal [Includes: Maltase (EC 3.2.1.20) (Alpha-glucosidase); Glucoamylase (EC 3.2.1.3) (Glucan 1,4-alpha-glucosidase)] | PSTPVNP | 1031 | 1037 |
| **O43497** | CACNA1G KIAA1123 | Voltage-dependent T-type calcium channel subunit alpha-1G (Cav3.1c) (NBR13) (Voltage-gated calcium channel subunit alpha Cav3.1) | PRTPPSP | 2321 | 2327 |
| **O43516** | WIPF1 WASPIP WIP | WAS/WASL-interacting protein family member 1 (Protein PRPL-2) (Wiskott-Aldrich syndrome protein-interacting protein) (WASP-interacting protein) | PSTPRP | 188 | 193 |
| **O43593** | HR | Lysine-specific demethylase hairless (EC 1.14.11.-) | PLTPHP | 180 | 185 |
| **O43760** | SYNGR2 UNQ352/PRO615 | Synaptogyrin-2 (Cellugyrin) | PTPDP | 184 | 188 |
| **O43854** | EDIL3 DEL1 | EGF-like repeat and discoidin I-like domain-containing protein 3 (Developmentally-regulated endothelial cell locus 1 protein) (Integrin-binding protein DEL1) | PCTPNP | 77 | 82 |
| **O43896** | KIF1C KIAA0706 | Kinesin-like protein KIF1C | PTPLQP | 1000 | 1005 |
| **O43909** | EXTL3 EXTL1L EXTR1 KIAA0519 | Exostosin-like 3 (EC 2.4.1.223) (EXT-related protein 1) (Glucuronyl-galactosyl-proteoglycan 4-alpha-N-acetylglucosaminyltransferase) (Hereditary multiple exostoses gene isolog) (Multiple exostosis-like protein 3) (Putative tumor suppressor protein EXTL3) | PHTPFDP | 615 | 621 |
| **O60237** | PPP1R12B MYPT2 | Protein phosphatase 1 regulatory subunit 12B (Myosin phosphatase-targeting subunit 2) (Myosin phosphatase target subunit 2) | PTTPASP | 730 | 736 |
| **O60243** | HS6ST1 HS6ST | Heparan-sulfate 6-O-sulfotransferase 1 (HS6ST-1) (EC 2.8.2.-) | PTPDP | 58 | 62 |
| **O60244** | MED14 ARC150 CRSP2 CXorf4 DRIP150 EXLM1 RGR1 TRAP170 | Mediator of RNA polymerase II transcription subunit 14 (Activator-recruited cofactor 150 kDa component) (ARC150) (Cofactor required for Sp1 transcriptional activation subunit 2) (CRSP complex subunit 2) (Mediator complex subunit 14) (RGR1 homolog) (hRGR1) (Thyroid hormone receptor-associated protein complex 170 kDa component) (Trap170) (Transcriptional coactivator CRSP150) (Vitamin D3 receptor-interacting protein complex 150 kDa component) (DRIP150) | PTPPP | 1067 | 1071 |
| **O60248** | SOX15 SOX12 SOX20 SOX26 SOX27 | Protein SOX-15 (Protein SOX-12) (Protein SOX-20) | PTPYNP | 216 | 221 |
| **O60292** | SIPA1L3 KIAA0545 SPAL3 | Signal-induced proliferation-associated 1-like protein 3 (SIPA1-like protein 3) (SPA-1-like protein 3) | PTTPAMP | 69 | 75 |
| **O60320** | FAM189A1 KIAA0574 TMEM228 | Protein FAM189A1 (Transmembrane protein 228) | PTPEP | 464 | 468 |
| **O60341** | KDM1A AOF2 KDM1 KIAA0601 LSD1 | Lysine-specific histone demethylase 1A (EC 1.-.-.-) (BRAF35-HDAC complex protein BHC110) (Flavin-containing amine oxidase domain-containing protein 2) | PITPGP | 779 | 784 |
| **O60393** | NOBOX | Homeobox protein NOBOX | PTPCP | 630 | 634 |
| **O60496** | DOK2 | Docking protein 2 (Downstream of tyrosine kinase 2) (p56(dok-2)) | PATPQP | 250 | 255 |
| **O60568** | PLOD3 | Procollagen-lysine,2-oxoglutarate 5-dioxygenase 3 (EC 1.14.11.4) (Lysyl hydroxylase 3) (LH3) | PTPFLP | 307 | 312 |
| **O60641** | SNAP91 KIAA0656 | Clathrin coat assembly protein AP180 (91 kDa synaptosomal-associated protein) (Clathrin coat-associated protein AP180) (Phosphoprotein F1-20) | PATPTP | 461 | 466 |
| **O60811** | PRAMEF2 | PRAME family member 2 | PTPCP | 454 | 458 |
| **O60885** | BRD4 HUNK1 | Bromodomain-containing protein 4 (Protein HUNK1) | PPTPLLP | 940 | 946 |
| **O60927** | PPP1R11 HCGV TCTE5 | Protein phosphatase 1 regulatory subunit 11 (Hemochromatosis candidate gene V protein) (HCG V) (Protein phosphatase inhibitor 3) | PTPTTP | 105 | 110 |
| **O75023** | LILRB5 LIR8 | Leukocyte immunoglobulin-like receptor subfamily B member 5 (CD85 antigen-like family member C) (Leukocyte immunoglobulin-like receptor 8) (LIR-8) (CD antigen CD85c) | PTPGP | 433 | 437 |
| **O75051** | PLXNA2 KIAA0463 OCT PLXN2 UNQ209/PRO235 | Plexin-A2 (Semaphorin receptor OCT) | PCTPLP | 900 | 905 |
| **O75052** | NOS1AP CAPON KIAA0464 | Carboxyl-terminal PDZ ligand of neuronal nitric oxide synthase protein (C-terminal PDZ ligand of neuronal nitric oxide synthase protein) (Nitric oxide synthase 1 adaptor protein) | PTTPKP | 391 | 396 |
| **O75056** | SDC3 KIAA0468 | Syndecan-3 (SYND3) | PSTPAAP | 188 | 194 |
| **O75081** | CBFA2T3 MTG16 MTGR2 ZMYND4 | Protein CBFA2T3 (MTG8-related protein 2) (Myeloid translocation gene on chromosome 16 protein) (hMTG16) (Zinc finger MYND domain-containing protein 4) | PTPPP | 336 | 340 |
| **O75112** | LDB3 KIAA0613 ZASP | LIM domain-binding protein 3 (Protein cypher) (Z-band alternatively spliced PDZ-motif protein) | PGTPGTP | 130 | 136 |
| **O75122** | CLASP2 KIAA0627 | CLIP-associating protein 2 (Cytoplasmic linker-associated protein 2) (Protein Orbit homolog 2) (hOrbit2) | PTPRSP | 897 | 902 |
| **O75128** | COBL KIAA0633 | Protein cordon-bleu | PSTPVP | 792 | 797 |
| **O75157** | TSC22D2 KIAA0669 TILZ4 | TSC22 domain family protein 2 (TSC22-related-inducible leucine zipper protein 4) | PTPAQP | 261 | 266 |
| **O75173** | ADAMTS4 KIAA0688 UNQ769/PRO1563 | A disintegrin and metalloproteinase with thrombospondin motifs 4 (ADAM-TS 4) (ADAM-TS4) (ADAMTS-4) (EC 3.4.24.82) (ADMP-1) (Aggrecanase-1) | PTPSTP | 805 | 810 |
| **O75179** | ANKRD17 GTAR KIAA0697 | Ankyrin repeat domain-containing protein 17 (Gene trap ankyrin repeat protein) (Serologically defined breast cancer antigen NY-BR-16) | PQTPTP | 1052 | 1057 |
| **O75298** | RTN2 NSPL1 | Reticulon-2 (Neuroendocrine-specific protein-like 1) (NSP-like protein 1) (Neuroendocrine-specific protein-like I) (NSP-like protein I) (NSPLI) | PTPPTP | 310 | 315 |
| **O75376** | NCOR1 KIAA1047 | Nuclear receptor corepressor 1 (N-CoR) (N-CoR1) | PTPGYP | 1593 | 1598 |
| **O75385** | ULK1 KIAA0722 | Serine/threonine-protein kinase ULK1 (EC 2.7.11.1) (Autophagy-related protein 1 homolog) (ATG1) (hATG1) (Unc-51-like kinase 1) | PYTPSP | 501 | 506 |
| **O75398** | DEAF1 SPN ZMYND5 | Deformed epidermal autoregulatory factor 1 homolog (Nuclear DEAF-1-related transcriptional regulator) (NUDR) (Suppressin) (Zinc finger MYND domain-containing protein 5) | PLTPGP | 169 | 174 |
| **O75417** | POLQ POLH | DNA polymerase theta (EC 2.7.7.7) (DNA polymerase eta) | PPTPIP | 1736 | 1741 |
| **O75553** | DAB1 | Disabled homolog 1 | PPTPATP | 294 | 300 |
| **O75592** | MYCBP2 KIAA0916 PAM | E3 ubiquitin-protein ligase MYCBP2 (EC 6.3.2.-) (Myc-binding protein 2) (Pam/highwire/rpm-1 protein) (Protein associated with Myc) | PTPKRP | 2354 | 2359 |
| **O75593** | FOXH1 FAST1 FAST2 | Forkhead box protein H1 (Forkhead activin signal transducer 1) (Fast-1) (hFAST-1) (Forkhead activin signal transducer 2) (Fast-2) | PTPPLP | 205 | 210 |
| **O75638** | CTAG2 ESO2 LAGE1 | Cancer/testis antigen 2 (CT2) (Autoimmunogenic cancer/testis antigen NY-ESO-2) (Cancer/testis antigen 6.2) (CT6.2) (L antigen family member 1) (LAGE-1) | PGTPGPP | 180 | 186 |
| **O75691** | UTP20 DRIM | Small subunit processome component 20 homolog (Down-regulated in metastasis protein) (Novel nucleolar protein 73) (NNP73) (Protein Key-1A6) | PGTPDP | 1739 | 1744 |
| **O75764** | TCEA3 TFIISH | Transcription elongation factor A protein 3 (Transcription elongation factor S-II protein 3) (Transcription elongation factor TFIIS.h) | PKTPSSP | 159 | 165 |
| **O75808** | CAPN15 SOLH | Calpain-15 (EC 3.4.22.-) (Small optic lobes homolog) | PGTPAP | 906 | 911 |
| **O75864** | PPP1R37 KIAA1986 LRRC68 | Protein phosphatase 1 regulatory subunit 37 (Leucine-rich repeat-containing protein 68) | PTPPSP | 587 | 592 |
| **O75909** | CCNK CPR4 | Cyclin-K | PTPQVP | 281 | 286 |
| **O75923** | DYSF FER1L1 | Dysferlin (Dystrophy-associated fer-1-like protein) (Fer-1-like protein 1) | PPTPLEP | 135 | 141 |
| **O75949** | FAM155B TED TMEM28 | Transmembrane protein FAM155B (Protein TED) (Transmembrane protein 28) | PTTPAPP | 159 | 165 |
| **O75956** | CDK2AP2 DOC1R | Cyclin-dependent kinase 2-associated protein 2 (CDK2-associated protein 2) (DOC-1-related protein) (DOC-1R) | PGTPVP | 21 | 26 |
| **O76013** | KRT36 HHA6 HKA6 KRTHA6 | Keratin, type I cuticular Ha6 (Hair keratin, type I Ha6) (Keratin-36) (K36) | PCTPAP | 432 | 437 |
| **O76039** | CDKL5 STK9 | Cyclin-dependent kinase-like 5 (EC 2.7.11.22) (Serine/threonine-protein kinase 9) | PHTPCVP | 944 | 950 |
| **O76090** | BEST1 VMD2 | Bestrophin-1 (TU15B) (Vitelliform macular dystrophy protein 2) | PQTPLSP | 468 | 474 |
| **O77932** | DXO DOM3L DOM3Z NG6 | Decapping and exoribonuclease protein (DXO) (EC 3.1.13.-) (EC 3.6.1.-) (Dom-3 homolog Z) | PKTPSP | 390 | 395 |
| **O94804** | STK10 LOK | Serine/threonine-protein kinase 10 (EC 2.7.11.1) (Lymphocyte-oriented kinase) | PSTPLAP | 362 | 368 |
| **O94810** | RGS11 | Regulator of G-protein signaling 11 (RGS11) | PTPVEP | 449 | 454 |
| **O94854** | KIAA0754 | Uncharacterized protein KIAA0754 | PTPEEP | 872 | 877 |
| **O94886** | TMEM63A KIAA0489 KIAA0792 | CSC1-like protein 1 (Transmembrane protein 63A) | PFTPYVP | 748 | 754 |
| **O94933** | SLITRK3 KIAA0848 | SLIT and NTRK-like protein 3 | PRTPRPP | 339 | 345 |
| **O94989** | ARHGEF15 KIAA0915 | Rho guanine nucleotide exchange factor 15 (Ephexin-5) (E5) (Vsm-RhoGEF) | PTPSP | 98 | 102 |
| **O94993** | SOX30 | Transcription factor SOX-30 | PLTPVP | 290 | 295 |
| **O95104** | SCAF4 KIAA1172 SFRS15 | Splicing factor, arginine/serine-rich 15 (CTD-binding SR-like protein RA4) (SR-related and CTD-associated factor 4) | PTPPFP | 372 | 377 |
| **O95140** | MFN2 CPRP1 KIAA0214 | Mitofusin-2 (EC 3.6.5.-) (Transmembrane GTPase MFN2) | PLTPANP | 578 | 584 |
| **O95155** | UBE4B HDNB1 KIAA0684 UFD2 | Ubiquitin conjugation factor E4 B (EC 6.3.2.-) (Homozygously deleted in neuroblastoma 1) (Ubiquitin fusion degradation protein 2) | PSTPLSP | 306 | 312 |
| **O95231** | VENTX HPX42B VENTX2 | Homeobox protein VENTX (VENT homeobox homolog) (VENT-like homeobox protein 2) | PPTPGRP | 229 | 235 |
| **O95251** | KAT7 HBO1 HBOa MYST2 | Histone acetyltransferase KAT7 (EC 2.3.1.48) (Histone acetyltransferase binding to ORC1) (Lysine acetyltransferase 7) (MOZ, YBF2/SAS3, SAS2 and TIP60 protein 2) (MYST-2) | PTPVTP | 84 | 89 |
| **O95267** | RASGRP1 RASGRP | RAS guanyl-releasing protein 1 (Calcium and DAG-regulated guanine nucleotide exchange factor II) (CalDAG-GEFII) (Ras guanyl-releasing protein) | PLTPSKP | 442 | 448 |
| **O95382** | MAP3K6 ASK2 MAPKKK6 MEKK6 | Mitogen-activated protein kinase kinase kinase 6 (EC 2.7.11.25) (Apoptosis signal-regulating kinase 2) | PTPEPP | 489 | 494 |
| **O95400** | CD2BP2 KIAA1178 | CD2 antigen cytoplasmic tail-binding protein 2 (CD2 cytoplasmic domain-binding protein 2) (CD2 tail-binding protein 2) (U5 snRNP 52K protein) (U5-52K) | PTPPP | 242 | 246 |
| **O95466** | FMNL1 C17orf1 C17orf1B FMNL FRL1 | Formin-like protein 1 (CLL-associated antigen KW-13) (Leukocyte formin) | PTPGVP | 517 | 522 |
| **O95486** | SEC24A | Protein transport protein Sec24A (SEC24-related protein A) | PSTPLKP | 361 | 367 |
| **O95521** | PRAMEF1 | PRAME family member 1 | PTPCP | 454 | 458 |
| **O95644** | NFATC1 NFAT2 NFATC | Nuclear factor of activated T-cells, cytoplasmic 1 (NF-ATc1) (NFATc1) (NFAT transcription complex cytosolic component) (NF-ATc) (NFATc) | PTPSP | 283 | 287 |
| **O95685** | PPP1R3D PPP1R6 | Protein phosphatase 1 regulatory subunit 3D (Protein phosphatase 1 regulatory subunit 6) (PP1 subunit R6) (Protein phosphatase 1-binding subunit R6) | PPTPAP | 52 | 57 |
| **O95744** | PMS2P2 PMS2L14 PMS2L2 PMS4 | Putative postmeiotic segregation increased 2-like protein 2 (Postmeiotic segregation increased 2-like protein 14) (Postmeiotic segregation increased protein 4) (Putative postmeiotic segregation increased 2 pseudogene 2) | PPTPTP | 8 | 13 |
| **O95785** | WIZ ZNF803 | Protein Wiz (Widely-interspaced zinc finger-containing protein) (Zinc finger protein 803) | PTPKNP | 997 | 1002 |
| **O95831** | AIFM1 AIF PDCD8 | Apoptosis-inducing factor 1, mitochondrial (EC 1.1.1.-) (Programmed cell death protein 8) | PSTPAVP | 545 | 551 |
| **O95832** | CLDN1 CLD1 SEMP1 UNQ481/PRO944 | Claudin-1 (Senescence-associated epithelial membrane protein) | PTPRP | 194 | 198 |
| **O95886** | DLGAP3 DAP3 | Disks large-associated protein 3 (DAP-3) (PSD-95/SAP90-binding protein 3) (SAP90/PSD-95-associated protein 3) (SAPAP3) | PTPGP | 762 | 766 |
| **O95931** | CBX7 | Chromobox protein homolog 7 | PWTPALP | 210 | 216 |
| **O95935** | TBX18 | T-box transcription factor TBX18 (T-box protein 18) | PGTPLP | 129 | 134 |
| **O96005** | CLPTM1 | Cleft lip and palate transmembrane protein 1 | PTPAP | 623 | 627 |
| **O96017** | CHEK2 CDS1 CHK2 RAD53 | Serine/threonine-protein kinase Chk2 (EC 2.7.11.1) (CHK2 checkpoint homolog) (Cds1 homolog) (Hucds1) (hCds1) (Checkpoint kinase 2) | PTPAP | 88 | 92 |
| **P00519** | ABL1 ABL JTK7 | Tyrosine-protein kinase ABL1 (EC 2.7.10.2) (Abelson murine leukemia viral oncogene homolog 1) (Abelson tyrosine-protein kinase 1) (Proto-oncogene c-Abl) (p150) | PATPKP | 961 | 966 |
| **P00748** | F12 | Coagulation factor XII (EC 3.4.21.38) (Hageman factor) (HAF) [Cleaved into: Coagulation factor XIIa heavy chain; Beta-factor XIIa part 1; Coagulation factor XIIa light chain (Beta-factor XIIa part 2)] | PPTPVSP | 303 | 309 |
| **P00846** | MT-ATP6 ATP6 ATPASE6 MTATP6 | ATP synthase subunit a (F-ATPase protein 6) | PTPLIP | 134 | 139 |
| **P00966** | ASS1 ASS | Argininosuccinate synthase (EC 6.3.4.5) (Citrulline--aspartate ligase) | PVTPKNP | 172 | 178 |
| **P01042** | KNG1 BDK KNG | Kininogen-1 (Alpha-2-thiol proteinase inhibitor) (Fitzgerald factor) (High molecular weight kininogen) (HMWK) (Williams-Fitzgerald-Flaujeac factor) [Cleaved into: Kininogen-1 heavy chain; T-kinin (Ile-Ser-Bradykinin); Bradykinin (Kallidin I); Lysyl-bradykinin (Kallidin II); Kininogen-1 light chain; Low molecular weight growth-promoting factor] | PTPIP | 545 | 549 |
| **P01614** | 0 | Ig kappa chain V-II region Cum | PVTPGEP | 13 | 19 |
| **P01732** | CD8A MAL | T-cell surface glycoprotein CD8 alpha chain (T-lymphocyte differentiation antigen T8/Leu-2) (CD antigen CD8a) | PPTPAP | 145 | 150 |
| **P01876** | IGHA1 | Ig alpha-1 chain C region | PSTPPTP | 104 | 110 |
| **P02452** | COL1A1 | Collagen alpha-1(I) chain (Alpha-1 type I collagen) | PGTPGP | 946 | 951 |
| **P02458** | COL2A1 | Collagen alpha-1(II) chain (Alpha-1 type II collagen) [Cleaved into: Collagen alpha-1(II) chain; Chondrocalcin] | PGTPGNP | 155 | 161 |
| **P02462** | COL4A1 | Collagen alpha-1(IV) chain [Cleaved into: Arresten] | PGTPGPP | 181 | 187 |
| **P02686** | MBP | Myelin basic protein (MBP) (Myelin A1 protein) (Myelin membrane encephalitogenic protein) | PRTPPP | 230 | 235 |
| **P02746** | C1QB | Complement C1q subcomponent subunit B | PGTPGP | 44 | 49 |
| **P02786** | TFRC | Transferrin receptor protein 1 (TR) (TfR) (TfR1) (Trfr) (T9) (p90) (CD antigen CD71) [Cleaved into: Transferrin receptor protein 1, serum form (sTfR)] | PYTPGFP | 308 | 314 |
| **P03891** | MT-ND2 MTND2 NADH2 ND2 | NADH-ubiquinone oxidoreductase chain 2 (EC 1.6.5.3) (NADH dehydrogenase subunit 2) | PTPFLP | 322 | 327 |
| **P04049** | RAF1 RAF | RAF proto-oncogene serine/threonine-protein kinase (EC 2.7.11.1) (Proto-oncogene c-RAF) (cRaf) (Raf-1) | PKTPVP | 308 | 313 |
| **P04090** | RLN2 | Prorelaxin H2 [Cleaved into: Relaxin B chain; Relaxin A chain] | PQTPRP | 63 | 68 |
| **P04637** | TP53 P53 | Cellular tumor antigen p53 (Antigen NY-CO-13) (Phosphoprotein p53) (Tumor suppressor p53) | PTPAAP | 80 | 85 |
| **P04808** | RLN1 | Prorelaxin H1 [Cleaved into: Relaxin B chain; Relaxin A chain] | PQTPRP | 63 | 68 |
| **P04920** | SLC4A2 AE2 EPB3L1 HKB3 MPB3L | Anion exchange protein 2 (AE 2) (Anion exchanger 2) (Non-erythroid band 3-like protein) (BND3L) (Solute carrier family 4 member 2) | PGTPGFP | 25 | 31 |
| **P05111** | INHA | Inhibin alpha chain | PPTPAQP | 311 | 317 |
| **P05305** | EDN1 | Endothelin-1 (Preproendothelin-1) (PPET1) [Cleaved into: Endothelin-1 (ET-1); Big endothelin-1] | PTPSPP | 39 | 44 |
| **P05423** | POLR3D BN51 BN51T | DNA-directed RNA polymerase III subunit RPC4 (RNA polymerase III subunit C4) (DNA-directed RNA polymerase III subunit D) (Protein BN51) (RNA polymerase III 47 kDa subunit) (RPC53 homolog) | PSTPGGP | 10 | 16 |
| **P05997** | COL5A2 | Collagen alpha-2(V) chain | PGTPGP | 1085 | 1090 |
| **P06730** | EIF4E EIF4EL1 EIF4F | Eukaryotic translation initiation factor 4E (eIF-4E) (eIF4E) (eIF-4F 25 kDa subunit) (mRNA cap-binding protein) | PTPNPP | 10 | 15 |
| **P06734** | FCER2 CD23A CLEC4J FCE2 IGEBF | Low affinity immunoglobulin epsilon Fc receptor (BLAST-2) (C-type lectin domain family 4 member J) (Fc-epsilon-RII) (Immunoglobulin E-binding factor) (Lymphocyte IgE receptor) (CD antigen CD23) [Cleaved into: Low affinity immunoglobulin epsilon Fc receptor membrane-bound form; Low affinity immunoglobulin epsilon Fc receptor soluble form] | PTPSAP | 313 | 318 |
| **P06748** | NPM1 NPM | Nucleophosmin (NPM) (Nucleolar phosphoprotein B23) (Nucleolar protein NO38) (Numatrin) | PKTPKGP | 235 | 241 |
| **P07101** | TH TYH | Tyrosine 3-monooxygenase (EC 1.14.16.2) (Tyrosine 3-hydroxylase) (TH) | PTPRSP | 58 | 63 |
| **P07199** | CENPB | Major centromere autoantigen B (Centromere protein B) (CENP-B) | PRTPAAP | 148 | 154 |
| **P07359** | GP1BA | Platelet glycoprotein Ib alpha chain (GP-Ib alpha) (GPIb-alpha) (GPIbA) (Glycoprotein Ibalpha) (Antigen CD42b-alpha) (CD antigen CD42b) [Cleaved into: Glycocalicin] | PTPSP | 382 | 386 |
| **P07476** | IVL | Involucrin | PTPLPP | 37 | 42 |
| **P07498** | CSN3 CASK CSN10 CSNK | Kappa-casein | PTPAP | 142 | 146 |
| **P08123** | COL1A2 | Collagen alpha-2(I) chain (Alpha-2 type I collagen) | PGTPGLP | 168 | 174 |
| **P08151** | GLI1 GLI | Zinc finger protein GLI1 (Glioma-associated oncogene) (Oncogene GLI) | PTPSP | 965 | 969 |
| **P08254** | MMP3 STMY1 | Stromelysin-1 (SL-1) (EC 3.4.24.17) (Matrix metalloproteinase-3) (MMP-3) (Transin-1) | PETPLVP | 270 | 276 |
| **P08575** | PTPRC CD45 | Receptor-type tyrosine-protein phosphatase C (EC 3.1.3.48) (Leukocyte common antigen) (L-CA) (T200) (CD antigen CD45) | PTPSP | 26 | 30 |
| **P08684** | CYP3A4 CYP3A3 | Cytochrome P450 3A4 (EC 1.14.13.-) (1,8-cineole 2-exo-monooxygenase) (EC 1.14.13.157) (Albendazole monooxygenase) (EC 1.14.13.32) (Albendazole sulfoxidase) (CYPIIIA3) (CYPIIIA4) (Cholesterol 25-hydroxylase) (EC 1.14.14.1) (Cytochrome P450 3A3) (Cytochrome P450 HLp) (Cytochrome P450 NF-25) (Cytochrome P450-PCN1) (Nifedipine oxidase) (Quinine 3-monooxygenase) (EC 1.14.13.67) (Taurochenodeoxycholate 6-alpha-hydroxylase) (EC 1.14.13.97) | PTPLP | 41 | 45 |
| **P08908** | HTR1A ADRB2RL1 ADRBRL1 | 5-hydroxytryptamine receptor 1A (5-HT-1A) (5-HT1A) (G-21) (Serotonin receptor 1A) | PTPCAP | 313 | 318 |
| **P09172** | DBH | Dopamine beta-hydroxylase (EC 1.14.17.1) (Dopamine beta-monooxygenase) [Cleaved into: Soluble dopamine beta-hydroxylase] | PTPQCP | 592 | 597 |
| **P09529** | INHBB | Inhibin beta B chain (Activin beta-B chain) | PTPPP | 30 | 34 |
| **P09884** | POLA1 POLA | DNA polymerase alpha catalytic subunit (EC 2.7.7.7) (DNA polymerase alpha catalytic subunit p180) | PLTPVP | 217 | 222 |
| **P0C0L4** | C4A CO4 CPAMD2 | Complement C4-A (Acidic complement C4) (C3 and PZP-like alpha-2-macroglobulin domain-containing protein 2) [Cleaved into: Complement C4 beta chain; Complement C4-A alpha chain; C4a anaphylatoxin; C4b-A; C4d-A; Complement C4 gamma chain] | PTPAP | 1243 | 1247 |
| **P0C0L5** | C4B CO4 CPAMD3; C4B_2 | Complement C4-B (Basic complement C4) (C3 and PZP-like alpha-2-macroglobulin domain-containing protein 3) [Cleaved into: Complement C4 beta chain; Complement C4-B alpha chain; C4a anaphylatoxin; C4b-B; C4d-B; Complement C4 gamma chain] | PTPAP | 1243 | 1247 |
| **P0C1S8** | WEE2 WEE1B | Wee1-like protein kinase 2 (EC 2.7.10.2) (Wee1-like protein kinase 1B) (Wee1B kinase) | PETPAQP | 92 | 98 |
| **P0C264** | SBK3 SGK110 | Uncharacterized serine/threonine-protein kinase SBK3 (EC 2.7.11.1) (SH3-binding domain kinase family member 3) (Sugen kinase 110) | PTPAPP | 193 | 198 |
| **P0C6A0** | ZGLP1 GLP1 | GATA-type zinc finger protein 1 (GATA-like protein 1) (GLP-1) | PGTPSAP | 116 | 122 |
| **P0C7P4** | UQCRFS1P1 UQCRFSL1 | Putative cytochrome b-c1 complex subunit Rieske-like protein 1 (Ubiquinol-cytochrome c reductase Rieske iron-sulfur subunit pseudogene 1) | PATPEQP | 44 | 50 |
| **P0C7T5** | ATXN1L BOAT BOAT1 | Ataxin-1-like (Brother of ataxin-1) (Brother of ATXN1) | PTPPP | 455 | 459 |
| **P0C7V0** | LINC00271 C6orf217 NCRNA00271 | Putative uncharacterized protein encoded by LINC00271 | PGTPGP | 27 | 32 |
| **P0C7V6** | SUPT20HL2 FAM48B2 | Putative transcription factor SPT20 homolog-like 2 | PTPPAP | 812 | 817 |
| **P0CG20** | PRR35 C16orf11 | Proline-rich protein 35 (Uncharacterized protein RJD1) | PTPDRP | 88 | 93 |
| **P0CG29** | GSTT2 | Glutathione S-transferase theta-2 (EC 2.5.1.18) (GST class-theta-2) | PTPSP | 226 | 230 |
| **P0CG30** | GSTT2B GSTT2 | Glutathione S-transferase theta-2B (EC 2.5.1.18) (GST class-theta-2) (Glutathione S-transferase theta-2) | PTPSP | 226 | 230 |
| **P0CG33** | GOLGA6D | Golgin subfamily A member 6D | PTPNIP | 517 | 522 |
| **P0CJ78** | ZNF865 | Zinc finger protein 865 | PATPVAP | 317 | 323 |
| **P0DKV0** | SPATA31C1 FAM75C1 | Spermatogenesis-associated protein 31C1 (Protein FAM75C1) | PHTPDP | 220 | 225 |
| **P0DMB1** | PRR23D2 | Proline-rich protein 23D2 | PPTPSP | 218 | 223 |
| **P10070** | GLI2 THP | Zinc finger protein GLI2 (GLI family zinc finger protein 2) (Tax helper protein) | PPTPLP | 901 | 906 |
| **P10071** | GLI3 | Transcriptional activator GLI3 (GLI3 form of 190 kDa) (GLI3-190) (GLI3 full length protein) (GLI3FL) [Cleaved into: Transcriptional repressor GLI3R (GLI3 C-terminally truncated form) (GLI3 form of 83 kDa) (GLI3-83)] | PPTPLP | 941 | 946 |
| **P10163** | PRB4 | Basic salivary proline-rich protein 4 (Salivary proline-rich protein Po) (Parotid o protein) (Salivary proline-rich protein II-1) [Cleaved into: Protein N1; Glycosylated protein A; Peptide P-D (Proline-rich peptide IB-5)] | PPTPGKP | 135 | 141 |
| **P10242** | MYB | Transcriptional activator Myb (Proto-oncogene c-Myb) | PRTPTP | 464 | 469 |
| **P10243** | MYBL1 AMYB | Myb-related protein A (A-Myb) (Myb-like protein 1) | PRTPTP | 553 | 558 |
| **P10244** | MYBL2 BMYB | Myb-related protein B (B-Myb) (Myb-like protein 2) | PHTPTP | 516 | 521 |
| **P10275** | AR DHTR NR3C4 | Androgen receptor (Dihydrotestosterone receptor) (Nuclear receptor subfamily 3 group C member 4) | PTPCAP | 281 | 286 |
| **P10515** | DLAT DLTA | Dihydrolipoyllysine-residue acetyltransferase component of pyruvate dehydrogenase complex, mitochondrial (EC 2.3.1.12) (70 kDa mitochondrial autoantigen of primary biliary cirrhosis) (PBC) (Dihydrolipoamide acetyltransferase component of pyruvate dehydrogenase complex) (M2 antigen complex 70 kDa subunit) (Pyruvate dehydrogenase complex component E2) (PDC-E2) (PDCE2) | PPTPPP | 320 | 325 |
| **P10589** | NR2F1 EAR3 ERBAL3 TFCOUP1 | COUP transcription factor 1 (COUP-TF1) (COUP transcription factor I) (COUP-TF I) (Nuclear receptor subfamily 2 group F member 1) (V-erbA-related protein 3) (EAR-3) | PHTPQTP | 49 | 55 |
| **P10632** | CYP2C8 | Cytochrome P450 2C8 (EC 1.14.14.1) (CYPIIC8) (Cytochrome P450 IIC2) (Cytochrome P450 MP-12) (Cytochrome P450 MP-20) (Cytochrome P450 form 1) (S-mephenytoin 4-hydroxylase) | PTPLP | 33 | 37 |
| **P10745** | RBP3 | Retinol-binding protein 3 (Interphotoreceptor retinoid-binding protein) (IRBP) (Interstitial retinol-binding protein) | PSTPEPP | 93 | 99 |
| **P10826** | RARB HAP NR1B2 | Retinoic acid receptor beta (RAR-beta) (HBV-activated protein) (Nuclear receptor subfamily 1 group B member 2) (RAR-epsilon) | PATPYP | 21 | 26 |
| **P11137** | MAP2 | Microtubule-associated protein 2 (MAP-2) | PGTPGTP | 1614 | 1620 |
| **P11498** | PC | Pyruvate carboxylase, mitochondrial (EC 6.4.1.1) (Pyruvic carboxylase) (PCB) | PTTPIP | 512 | 517 |
| **P11509** | CYP2A6 CYP2A3 | Cytochrome P450 2A6 (EC 1.14.13.-) (1,4-cineole 2-exo-monooxygenase) (CYPIIA6) (Coumarin 7-hydroxylase) (Cytochrome P450 IIA3) (Cytochrome P450(I)) | PTPLP | 37 | 41 |
| **P11532** | DMD | Dystrophin | PTTPSEP | 857 | 863 |
| **P11712** | CYP2C9 CYP2C10 | Cytochrome P450 2C9 (EC 1.14.13.-) ((R)-limonene 6-monooxygenase) (EC 1.14.13.80) ((S)-limonene 6-monooxygenase) (EC 1.14.13.48) ((S)-limonene 7-monooxygenase) (EC 1.14.13.49) (CYPIIC9) (Cholesterol 25-hydroxylase) (EC 1.14.99.38) (Cytochrome P-450MP) (Cytochrome P450 MP-4) (Cytochrome P450 MP-8) (Cytochrome P450 PB-1) (S-mephenytoin 4-hydroxylase) | PTPLP | 33 | 37 |
| **P12980** | LYL1 BHLHA18 | Protein lyl-1 (Class A basic helix-loop-helix protein 18) (bHLHa18) (Lymphoblastic leukemia-derived sequence 1) | PTPPGP | 217 | 222 |
| **P13725** | OSM | Oncostatin-M (OSM) | PPTPTP | 175 | 180 |
| **P14651** | HOXB3 HOX2G | Homeobox protein Hox-B3 (Homeobox protein Hox-2.7) (Homeobox protein Hox-2G) | PPTPAP | 322 | 327 |
| **P14784** | IL2RB | Interleukin-2 receptor subunit beta (IL-2 receptor subunit beta) (IL-2R subunit beta) (IL-2RB) (High affinity IL-2 receptor subunit beta) (p70-75) (p75) (CD antigen CD122) | PPTPGVP | 474 | 480 |
| **P15144** | ANPEP APN CD13 PEPN | Aminopeptidase N (AP-N) (hAPN) (EC 3.4.11.2) (Alanyl aminopeptidase) (Aminopeptidase M) (AP-M) (Microsomal aminopeptidase) (Myeloid plasma membrane glycoprotein CD13) (gp150) (CD antigen CD13) | PSTPLP | 254 | 259 |
| **P15407** | FOSL1 FRA1 | Fos-related antigen 1 (FRA-1) | PSTPEP | 238 | 243 |
| **P15502** | ELN | Elastin (Tropoelastin) | PSTPSSP | 637 | 643 |
| **P15812** | CD1E | T-cell surface glycoprotein CD1e, membrane-associated (hCD1e) (R2G1) (CD antigen CD1e) [Cleaved into: T-cell surface glycoprotein CD1e, soluble (sCD1e)] | PHTPSP | 341 | 346 |
| **P15822** | HIVEP1 ZNF40 | Zinc finger protein 40 (Cirhin interaction protein) (CIRIP) (Gate keeper of apoptosis-activating protein) (GAAP) (Human immunodeficiency virus type I enhancer-binding protein 1) (HIV-EP1) (Major histocompatibility complex-binding protein 1) (MBP-1) (Positive regulatory domain II-binding factor 1) (PRDII-BF1) | PTPGLP | 2369 | 2374 |
| **P16112** | ACAN AGC1 CSPG1 MSK16 | Aggrecan core protein (Cartilage-specific proteoglycan core protein) (CSPCP) (Chondroitin sulfate proteoglycan core protein 1) (Chondroitin sulfate proteoglycan 1) [Cleaved into: Aggrecan core protein 2] | PYTPSPP | 840 | 846 |
| **P17096** | HMGA1 HMGIY | High mobility group protein HMG-I/HMG-Y (HMG-I(Y)) (High mobility group AT-hook protein 1) (High mobility group protein A1) (High mobility group protein R) | PTPKRP | 52 | 57 |
| **P17676** | CEBPB TCF5 PP9092 | CCAAT/enhancer-binding protein beta (C/EBP beta) (Liver activator protein) (LAP) (Liver-enriched inhibitory protein) (LIP) (Nuclear factor NF-IL6) (Transcription factor 5) (TCF-5) | PGTPSP | 233 | 238 |
| **P18583** | SON C21orf50 DBP5 KIAA1019 NREBP HSPC310 HSPC312 | Protein SON (Bax antagonist selected in saccharomyces 1) (BASS1) (Negative regulatory element-binding protein) (NRE-binding protein) (Protein DBP-5) (SON3) | PMTPPLP | 1166 | 1172 |
| **P18858** | LIG1 | DNA ligase 1 (EC 6.5.1.1) (DNA ligase I) (Polydeoxyribonucleotide synthase [ATP] 1) | PTTPPKP | 181 | 187 |
| **P19022** | CDH2 CDHN NCAD | Cadherin-2 (CDw325) (Neural cadherin) (N-cadherin) (CD antigen CD325) | PSTPSP | 313 | 318 |
| **P20226** | TBP GTF2D1 TF2D TFIID | TATA-box-binding protein (TATA sequence-binding protein) (TATA-binding factor) (TATA-box factor) (Transcription initiation factor TFIID TBP subunit) | PMTPMTP | 142 | 148 |
| **P20333** | TNFRSF1B TNFBR TNFR2 | Tumor necrosis factor receptor superfamily member 1B (Tumor necrosis factor receptor 2) (TNF-R2) (Tumor necrosis factor receptor type II) (TNF-RII) (TNFR-II) (p75) (p80 TNF-alpha receptor) (CD antigen CD120b) (Etanercept) [Cleaved into: Tumor necrosis factor receptor superfamily member 1b, membrane form; Tumor necrosis factor-binding protein 2 (TBP-2) (TBPII)] | PTPEP | 229 | 233 |
| **P20393** | NR1D1 EAR1 HREV THRAL | Nuclear receptor subfamily 1 group D member 1 (Rev-erbA-alpha) (V-erbA-related protein 1) (EAR-1) | PTPGP | 245 | 249 |
| **P20700** | LMNB1 LMN2 LMNB | Lamin-B1 | PTTPLSP | 18 | 24 |
| **P20749** | BCL3 BCL4 D19S37 | B-cell lymphoma 3 protein (BCL-3) (Proto-oncogene BCL3) | PTPLYP | 107 | 112 |
| **P20815** | CYP3A5 | Cytochrome P450 3A5 (EC 1.14.14.1) (CYPIIIA5) (Cytochrome P450 HLp2) (Cytochrome P450-PCN3) | PTPLP | 41 | 45 |
| **P20853** | CYP2A7 | Cytochrome P450 2A7 (EC 1.14.14.1) (CYPIIA7) (Cytochrome P450 IIA4) | PTPLP | 37 | 41 |
| **P20929** | NEB | Nebulin | PTPITP | 6256 | 6261 |
| **P21675** | TAF1 BA2R CCG1 CCGS TAF2A | Transcription initiation factor TFIID subunit 1 (EC 2.3.1.48) (EC 2.7.11.1) (Cell cycle gene 1 protein) (TBP-associated factor 250 kDa) (p250) (Transcription initiation factor TFIID 250 kDa subunit) (TAF(II)250) (TAFII-250) (TAFII250) | PMTPGP | 1636 | 1641 |
| **P21817** | RYR1 RYDR | Ryanodine receptor 1 (RYR-1) (RyR1) (Skeletal muscle calcium release channel) (Skeletal muscle ryanodine receptor) (Skeletal muscle-type ryanodine receptor) (Type 1 ryanodine receptor) | PATPTLP | 1402 | 1408 |
| **P21860** | ERBB3 HER3 | Receptor tyrosine-protein kinase erbB-3 (EC 2.7.10.1) (Proto-oncogene-like protein c-ErbB-3) (Tyrosine kinase-type cell surface receptor HER3) | PVTPLSP | 1142 | 1148 |
| **P21917** | DRD4 | D(4) dopamine receptor (D(2C) dopamine receptor) (Dopamine D4 receptor) | PTPPAP | 246 | 251 |
| **P22083** | FUT4 ELFT FCT3A | Alpha-(1,3)-fucosyltransferase 4 (EC 2.4.1.-) (ELAM-1 ligand fucosyltransferase) (Fucosyltransferase 4) (Fucosyltransferase IV) (Fuc-TIV) (FucT-IV) (Galactoside 3-L-fucosyltransferase) | PTPSRP | 183 | 188 |
| **P22105** | TNXB HXBL TNX TNXB1 TNXB2 XB | Tenascin-X (TN-X) (Hexabrachion-like protein) | PTPPAP | 1875 | 1880 |
| **P22528** | SPRR1B | Cornifin-B (14.9 kDa pancornulin) (Small proline-rich protein IB) (SPR-IB) | PCTPPP | 9 | 14 |
| **P22531** | SPRR2E | Small proline-rich protein 2E (SPR-2E) (Small proline-rich protein II) (SPR-II) | PTPKCP | 19 | 24 |
| **P22532** | SPRR2D | Small proline-rich protein 2D (SPR-2D) (Small proline-rich protein II) (SPR-II) | PTPKCP | 19 | 24 |
| **P22670** | RFX1 | MHC class II regulatory factor RFX1 (Enhancer factor C) (EF-C) (Regulatory factor X 1) (RFX) (Transcription factor RFX1) | PTPSP | 91 | 95 |
| **P22735** | TGM1 KTG | Protein-glutamine gamma-glutamyltransferase K (EC 2.3.2.13) (Epidermal TGase) (Transglutaminase K) (TG(K)) (TGK) (TGase K) (Transglutaminase-1) (TGase-1) | PTTPSP | 20 | 25 |
| **P22894** | MMP8 CLG1 | Neutrophil collagenase (EC 3.4.24.34) (Matrix metalloproteinase-8) (MMP-8) (PMNL collagenase) (PMNL-CL) | PSTPKP | 273 | 278 |
| **P22897** | MRC1 CLEC13D CLEC13DL MRC1L1 | Macrophage mannose receptor 1 (MMR) (C-type lectin domain family 13 member D) (C-type lectin domain family 13 member D-like) (Human mannose receptor) (hMR) (Macrophage mannose receptor 1-like protein 1) (CD antigen CD206) | PTPAP | 789 | 793 |
| **P23219** | PTGS1 COX1 | Prostaglandin G/H synthase 1 (EC 1.14.99.1) (Cyclooxygenase-1) (COX-1) (Prostaglandin H2 synthase 1) (PGH synthase 1) (PGHS-1) (PHS 1) (Prostaglandin-endoperoxide synthase 1) | PTPVNP | 29 | 34 |
| **P23246** | SFPQ PSF | Splicing factor, proline- and glutamine-rich (100 kDa DNA-pairing protein) (hPOMp100) (DNA-binding p52/p100 complex, 100 kDa subunit) (Polypyrimidine tract-binding protein-associated-splicing factor) (PSF) (PTB-associated-splicing factor) | PTPTPP | 149 | 154 |
| **P23443** | RPS6KB1 STK14A | Ribosomal protein S6 kinase beta-1 (S6K-beta-1) (S6K1) (EC 2.7.11.1) (70 kDa ribosomal protein S6 kinase 1) (P70S6K1) (p70-S6K 1) (Ribosomal protein S6 kinase I) (Serine/threonine-protein kinase 14A) (p70 ribosomal S6 kinase alpha) (p70 S6 kinase alpha) (p70 S6K-alpha) (p70 S6KA) | PRTPVSP | 442 | 448 |
| **P23470** | PTPRG PTPG | Receptor-type tyrosine-protein phosphatase gamma (Protein-tyrosine phosphatase gamma) (R-PTP-gamma) (EC 3.1.3.48) | PTPSSP | 625 | 630 |
| **P23471** | PTPRZ1 HTPZP2 PTPRZ PTPRZ2 PTPZ | Receptor-type tyrosine-protein phosphatase zeta (R-PTP-zeta) (EC 3.1.3.48) (Protein-tyrosine phosphatase receptor type Z polypeptide 1) (Protein-tyrosine phosphatase receptor type Z polypeptide 2) (R-PTP-zeta-2) | PPTPIFP | 1685 | 1691 |
| **P23490** | LOR LRN | Loricrin | PTPQPP | 8 | 13 |
| **P23769** | GATA2 | Endothelial transcription factor GATA-2 (GATA-binding protein 2) | PTPTP | 454 | 458 |
| **P23771** | GATA3 | Trans-acting T-cell-specific transcription factor GATA-3 (GATA-binding factor 3) | PTPMHP | 419 | 424 |
| **P24043** | LAMA2 LAMM | Laminin subunit alpha-2 (Laminin M chain) (Laminin-12 subunit alpha) (Laminin-2 subunit alpha) (Laminin-4 subunit alpha) (Merosin heavy chain) | PTPAFP | 2735 | 2740 |
| **P24462** | CYP3A7 | Cytochrome P450 3A7 (EC 1.14.14.1) (CYPIIIA7) (Cytochrome P450-HFLA) | PTPLP | 41 | 45 |
| **P24468** | NR2F2 ARP1 TFCOUP2 | COUP transcription factor 2 (COUP-TF2) (Apolipoprotein A-I regulatory protein 1) (ARP-1) (COUP transcription factor II) (COUP-TF II) (Nuclear receptor subfamily 2 group F member 2) | PHTPQTP | 37 | 43 |
| **P24928** | POLR2A POLR2 | DNA-directed RNA polymerase II subunit RPB1 (RNA polymerase II subunit B1) (EC 2.7.7.6) (DNA-directed RNA polymerase II subunit A) (DNA-directed RNA polymerase III largest subunit) (RNA-directed RNA polymerase II subunit RPB1) (EC 2.7.7.48) | PTPGSP | 1567 | 1572 |
| **P25940** | COL5A3 | Collagen alpha-3(V) chain | PETPRP | 239 | 244 |
| **P26368** | U2AF2 U2AF65 | Splicing factor U2AF 65 kDa subunit (U2 auxiliary factor 65 kDa subunit) (hU2AF(65)) (hU2AF65) (U2 snRNP auxiliary factor large subunit) | PTPVP | 134 | 138 |
| **P26439** | HSD3B2 HSDB3B | 3 beta-hydroxysteroid dehydrogenase/Delta 5-->4-isomerase type 2 (3 beta-hydroxysteroid dehydrogenase/Delta 5-->4-isomerase type II) (3-beta-HSD II) (3-beta-HSD adrenal and gonadal type) [Includes: 3-beta-hydroxy-Delta(5)-steroid dehydrogenase (EC 1.1.1.145) (3-beta-hydroxy-5-ene steroid dehydrogenase) (Progesterone reductase); Steroid Delta-isomerase (EC 5.3.3.1) (Delta-5-3-ketosteroid isomerase)] | PTPYP | 151 | 155 |
| **P26572** | MGAT1 GGNT1 GLCT1 GLYT1 MGAT | Alpha-1,3-mannosyl-glycoprotein 2-beta-N-acetylglucosaminyltransferase (EC 2.4.1.101) (N-glycosyl-oligosaccharide-glycoprotein N-acetylglucosaminyltransferase I) (GNT-I) (GlcNAc-T I) | PVTPAP | 98 | 103 |
| **P27037** | ACVR2A ACVR2 | Activin receptor type-2A (EC 2.7.11.30) (Activin receptor type IIA) (ACTR-IIA) (ACTRIIA) | PVTPKPP | 129 | 135 |
| **P27540** | ARNT BHLHE2 | Aryl hydrocarbon receptor nuclear translocator (ARNT protein) (Class E basic helix-loop-helix protein 2) (bHLHe2) (Dioxin receptor, nuclear translocator) (Hypoxia-inducible factor 1-beta) (HIF-1-beta) (HIF1-beta) | PPTPRP | 591 | 596 |
| **P27658** | COL8A1 C3orf7 | Collagen alpha-1(VIII) chain (Endothelial collagen) [Cleaved into: Vastatin] | PPTPPP | 575 | 580 |
| **P27815** | PDE4A DPDE2 | cAMP-specific 3',5'-cyclic phosphodiesterase 4A (EC 3.1.4.53) (DPDE2) (PDE46) | PTPSP | 114 | 118 |
| **P27986** | PIK3R1 GRB1 | Phosphatidylinositol 3-kinase regulatory subunit alpha (PI3-kinase regulatory subunit alpha) (PI3K regulatory subunit alpha) (PtdIns-3-kinase regulatory subunit alpha) (Phosphatidylinositol 3-kinase 85 kDa regulatory subunit alpha) (PI3-kinase subunit p85-alpha) (PtdIns-3-kinase regulatory subunit p85-alpha) | PPTPKP | 84 | 89 |
| **P28067** | HLA-DMA DMA RING6 | HLA class II histocompatibility antigen, DM alpha chain (MHC class II antigen DMA) (Really interesting new gene 6 protein) | PTPMWP | 31 | 36 |
| **P28324** | ELK4 SAP1 | ETS domain-containing protein Elk-4 (Serum response factor accessory protein 1) (SAP-1) (SRF accessory protein 1) | PRTPSPP | 265 | 271 |
| **P28698** | MZF1 MZF ZNF42 ZSCAN6 | Myeloid zinc finger 1 (MZF-1) (Zinc finger and SCAN domain-containing protein 6) (Zinc finger protein 42) | PPTPEP | 160 | 165 |
| **P28908** | TNFRSF8 CD30 D1S166E | Tumor necrosis factor receptor superfamily member 8 (CD30L receptor) (Ki-1 antigen) (Lymphocyte activation antigen CD30) (CD antigen CD30) | PTPVSP | 179 | 184 |
| **P29372** | MPG AAG ANPG MID1 | DNA-3-methyladenine glycosylase (EC 3.2.2.21) (3-alkyladenine DNA glycosylase) (3-methyladenine DNA glycosidase) (ADPG) (N-methylpurine-DNA glycosylase) | PTTPGP | 65 | 70 |
| **P29400** | COL4A5 | Collagen alpha-5(IV) chain | PGTPGLP | 101 | 107 |
| **P29474** | NOS3 | Nitric oxide synthase, endothelial (EC 1.14.13.39) (Constitutive NOS) (cNOS) (EC-NOS) (Endothelial NOS) (eNOS) (NOS type III) (NOSIII) | PATPAP | 31 | 36 |
| **P30203** | CD6 | T-cell differentiation antigen CD6 (T12) (TP120) (CD antigen CD6) [Cleaved into: Soluble CD6] | PPTPELP | 99 | 105 |
| **P30988** | CALCR | Calcitonin receptor (CT-R) | PTPILP | 37 | 42 |
| **P30989** | NTSR1 NTRR | Neurotensin receptor type 1 (NT-R-1) (NTR1) (High-affinity levocabastine-insensitive neurotensin receptor) (NTRH) | PGTPGTP | 8 | 14 |
| **P31321** | PRKAR1B | cAMP-dependent protein kinase type I-beta regulatory subunit | PTPPNP | 84 | 89 |
| **P31629** | HIVEP2 | Transcription factor HIVEP2 (Human immunodeficiency virus type I enhancer-binding protein 2) (HIV-EP2) (MHC-binding protein 2) (MBP-2) | PSTPSSP | 2296 | 2302 |
| **P31997** | CEACAM8 CGM6 | Carcinoembryonic antigen-related cell adhesion molecule 8 (CD67 antigen) (Carcinoembryonic antigen CGM6) (Non-specific cross-reacting antigen NCA-95) (CD antigen CD66b) | PETPKP | 142 | 147 |
| **P33241** | LSP1 WP34 | Lymphocyte-specific protein 1 (47 kDa actin-binding protein) (52 kDa phosphoprotein) (pp52) (Lymphocyte-specific antigen WP34) | PRTPSP | 173 | 178 |
| **P33260** | CYP2C18 | Cytochrome P450 2C18 (EC 1.14.14.1) (CYPIIC18) (Cytochrome P450-6b/29c) | PTPLP | 33 | 37 |
| **P33261** | CYP2C19 | Cytochrome P450 2C19 (EC 1.14.13.-) ((R)-limonene 6-monooxygenase) (EC 1.14.13.80) ((S)-limonene 6-monooxygenase) (EC 1.14.13.48) ((S)-limonene 7-monooxygenase) (EC 1.14.13.49) (CYPIIC17) (CYPIIC19) (Cytochrome P450-11A) (Cytochrome P450-254C) (Mephenytoin 4-hydroxylase) | PTPLP | 33 | 37 |
| **P35321** | SPRR1A | Cornifin-A (19 kDa pancornulin) (SPRK) (Small proline-rich protein IA) (SPR-IA) | PCTPPP | 9 | 14 |
| **P35325** | SPRR2B | Small proline-rich protein 2B (SPR-2B) | PTPKCP | 19 | 24 |
| **P35326** | SPRR2A | Small proline-rich protein 2A (SPR-2A) (2-1) | PTPKCP | 19 | 24 |
| **P35523** | CLCN1 CLC1 | Chloride channel protein 1 (ClC-1) (Chloride channel protein, skeletal muscle) | PETPVP | 927 | 932 |
| **P35712** | SOX6 | Transcription factor SOX-6 | PSTPQPP | 386 | 392 |
| **P36402** | TCF7 TCF1 | Transcription factor 7 (TCF-7) (T-cell-specific transcription factor 1) (T-cell factor 1) (TCF-1) | PTPAP | 171 | 175 |
| **P36776** | LONP1 PRSS15 | Lon protease homolog, mitochondrial (EC 3.4.21.-) (LONHs) (Lon protease-like protein) (LONP) (Mitochondrial ATP-dependent protease Lon) (Serine protease 15) | PTPELP | 262 | 267 |
| **P36915** | GNL1 HSR1 | Guanine nucleotide-binding protein-like 1 (GTP-binding protein HSR1) | PRTPQDP | 261 | 267 |
| **P36941** | LTBR D12S370 TNFCR TNFR3 TNFRSF3 | Tumor necrosis factor receptor superfamily member 3 (Lymphotoxin-beta receptor) (Tumor necrosis factor C receptor) (Tumor necrosis factor receptor 2-related protein) (Tumor necrosis factor receptor type III) (TNF-RIII) (TNFR-III) | PATPEPP | 379 | 385 |
| **P36956** | SREBF1 BHLHD1 SREBP1 | Sterol regulatory element-binding protein 1 (SREBP-1) (Class D basic helix-loop-helix protein 1) (bHLHd1) (Sterol regulatory element-binding transcription factor 1) [Cleaved into: Processed sterol regulatory element-binding protein 1] | PTPQP | 135 | 139 |
| **P37198** | NUP62 | Nuclear pore glycoprotein p62 (62 kDa nucleoporin) (Nucleoporin Nup62) | PFTPATP | 189 | 195 |
| **P39059** | COL15A1 | Collagen alpha-1(XV) chain [Cleaved into: Restin (Endostatin-XV) (Related to endostatin) (Restin-I); Restin-2 (Restin-II); Restin-3 (Restin-III); Restin-4 (Restin-IV)] | PPTPSSP | 280 | 286 |
| **P39060** | COL18A1 | Collagen alpha-1(XVIII) chain [Cleaved into: Endostatin] | PTPSPP | 191 | 196 |
| **P39880** | CUX1 CUTL1 | Homeobox protein cut-like 1 (CCAAT displacement protein) (CDP) (Homeobox protein cux-1) | PTTPLP | 1102 | 1107 |
| **P40225** | THPO MGDF | Thrombopoietin (C-mpl ligand) (ML) (Megakaryocyte colony-stimulating factor) (Megakaryocyte growth and development factor) (MGDF) (Myeloproliferative leukemia virus oncogene ligand) | PTPTP | 330 | 334 |
| **P41002** | CCNF FBX1 FBXO1 | Cyclin-F (F-box only protein 1) | PATPGP | 678 | 683 |
| **P41161** | ETV5 ERM | ETS translocation variant 5 (Ets-related protein ERM) | PTTPLSP | 137 | 143 |
| **P41235** | HNF4A HNF4 NR2A1 TCF14 | Hepatocyte nuclear factor 4-alpha (HNF-4-alpha) (Nuclear receptor subfamily 2 group A member 1) (Transcription factor 14) (TCF-14) (Transcription factor HNF-4) | PETPQP | 430 | 435 |
| **P42345** | MTOR FRAP FRAP1 FRAP2 RAFT1 RAPT1 | Serine/threonine-protein kinase mTOR (EC 2.7.11.1) (FK506-binding protein 12-rapamycin complex-associated protein 1) (FKBP12-rapamycin complex-associated protein) (Mammalian target of rapamycin) (mTOR) (Mechanistic target of rapamycin) (Rapamycin and FKBP12 target 1) (Rapamycin target protein 1) | PTPSP | 1860 | 1864 |
| **P42768** | WAS IMD2 | Wiskott-Aldrich syndrome protein (WASp) | PPTPRGP | 353 | 359 |
| **P43234** | CTSO CTSO1 | Cathepsin O (EC 3.4.22.42) | PFTPTWP | 28 | 34 |
| **P43354** | NR4A2 NOT NURR1 TINUR | Nuclear receptor subfamily 4 group A member 2 (Immediate-early response protein NOT) (Orphan nuclear receptor NURR1) (Transcriptionally-inducible nuclear receptor) | PPTPTTP | 127 | 133 |
| **P43694** | GATA4 | Transcription factor GATA-4 (GATA-binding factor 4) | PTPRVP | 40 | 45 |
| **P45985** | MAP2K4 JNKK1 MEK4 MKK4 PRKMK4 SEK1 SERK1 SKK1 | Dual specificity mitogen-activated protein kinase kinase 4 (MAP kinase kinase 4) (MAPKK 4) (EC 2.7.12.2) (JNK-activating kinase 1) (MAPK/ERK kinase 4) (MEK 4) (SAPK/ERK kinase 1) (SEK1) (Stress-activated protein kinase kinase 1) (SAPK kinase 1) (SAPKK-1) (SAPKK1) (c-Jun N-terminal kinase kinase 1) (JNKK) | PATPSSP | 389 | 395 |
| **P46013** | MKI67 | Antigen KI-67 | PATPKKP | 692 | 698 |
| **P46379** | BAG6 BAT3 G3 | Large proline-rich protein BAG6 (BAG family molecular chaperone regulator 6) (BCL2-associated athanogene 6) (BAG-6) (BAG6) (HLA-B-associated transcript 3) (Protein G3) (Protein Scythe) | PTPAP | 259 | 263 |
| **P46695** | IER3 DIF2 IEX1 PRG1 | Radiation-inducible immediate-early gene IEX-1 (Differentiation-dependent gene 2 protein) (Protein DIF-2) (Immediate early protein GLY96) (Immediate early response 3 protein) (PACAP-responsive gene 1 protein) (Protein PRG1) | PTPAP | 17 | 21 |
| **P47902** | CDX1 | Homeobox protein CDX-1 (Caudal-type homeobox protein 1) | PGTPSSP | 119 | 125 |
| **P47985** | UQCRFS1 | Cytochrome b-c1 complex subunit Rieske, mitochondrial (EC 1.10.2.2) (Complex III subunit 5) (Cytochrome b-c1 complex subunit 5) (Rieske iron-sulfur protein) (RISP) (Ubiquinol-cytochrome c reductase iron-sulfur subunit) [Cleaved into: Cytochrome b-c1 complex subunit 11 (Complex III subunit IX) (Ubiquinol-cytochrome c reductase 8 kDa protein)] | PATPEQP | 35 | 41 |
| **P48634** | PRRC2A BAT2 G2 | Protein PRRC2A (HLA-B-associated transcript 2) (Large proline-rich protein BAT2) (Proline-rich and coiled-coil-containing protein 2A) (Protein G2) | PTPDGP | 274 | 279 |
| **P48681** | NES Nbla00170 | Nestin | PPTPQAP | 391 | 397 |
| **P48730** | CSNK1D HCKID | Casein kinase I isoform delta (CKI-delta) (CKId) (EC 2.7.11.1) (Tau-protein kinase CSNK1D) (EC 2.7.11.26) | PPTPLTP | 342 | 348 |
| **P48736** | PIK3CG | Phosphatidylinositol 4,5-bisphosphate 3-kinase catalytic subunit gamma isoform (PI3-kinase subunit gamma) (PI3K-gamma) (PI3Kgamma) (PtdIns-3-kinase subunit gamma) (EC 2.7.1.153) (Phosphatidylinositol 4,5-bisphosphate 3-kinase 110 kDa catalytic subunit gamma) (PtdIns-3-kinase subunit p110-gamma) (p110gamma) (Phosphoinositide-3-kinase catalytic gamma polypeptide) (Serine/threonine protein kinase PIK3CG) (EC 2.7.11.1) (p120-PI3K) | PTPDP | 534 | 538 |
| **P49006** | MARCKSL1 MLP MRP | MARCKS-related protein (MARCKS-like protein 1) (Macrophage myristoylated alanine-rich C kinase substrate) (Mac-MARCKS) (MacMARCKS) | PSTPSGP | 176 | 182 |
| **P49137** | MAPKAPK2 | MAP kinase-activated protein kinase 2 (MAPK-activated protein kinase 2) (MAPKAP kinase 2) (MAPKAP-K2) (MAPKAPK-2) (MK-2) (MK2) (EC 2.7.11.1) | PPTPALP | 23 | 29 |
| **P49327** | FASN FAS | Fatty acid synthase (EC 2.3.1.85) [Includes: [Acyl-carrier-protein] S-acetyltransferase (EC 2.3.1.38); [Acyl-carrier-protein] S-malonyltransferase (EC 2.3.1.39); 3-oxoacyl-[acyl-carrier-protein] synthase (EC 2.3.1.41); 3-oxoacyl-[acyl-carrier-protein] reductase (EC 1.1.1.100); 3-hydroxyacyl-[acyl-carrier-protein] dehydratase (EC 4.2.1.59); Enoyl-[acyl-carrier-protein] reductase (EC 1.3.1.39); Oleoyl-[acyl-carrier-protein] hydrolase (EC 3.1.2.14)] | PTPNP | 975 | 979 |
| **P49418** | AMPH AMPH1 | Amphiphysin | PTPSAP | 596 | 601 |
| **P49450** | CENPA | Histone H3-like centromeric protein A (Centromere autoantigen A) (Centromere protein A) (CENP-A) | PTPTP | 20 | 24 |
| **P49454** | CENPF | Centromere protein F (CENP-F) (AH antigen) (Kinetochore protein CENPF) (Mitosin) | PTPATP | 3041 | 3046 |
| **P49643** | PRIM2 PRIM2A | DNA primase large subunit (EC 2.7.7.-) (DNA primase 58 kDa subunit) (p58) | PETPQP | 468 | 473 |
| **P49711** | CTCF | Transcriptional repressor CTCF (11-zinc finger protein) (CCCTC-binding factor) (CTCFL paralog) | PVTPAPP | 640 | 646 |
| **P49715** | CEBPA CEBP | CCAAT/enhancer-binding protein alpha (C/EBP alpha) | PTPPP | 225 | 229 |
| **P49756** | RBM25 RNPC7 | RNA-binding protein 25 (Arg/Glu/Asp-rich protein of 120 kDa) (RED120) (Protein S164) (RNA-binding motif protein 25) (RNA-binding region-containing protein 7) | PGTPMIP | 35 | 41 |
| **P49765** | VEGFB VRF | Vascular endothelial growth factor B (VEGF-B) (VEGF-related factor) (VRF) | PTPAP | 168 | 172 |
| **P49913** | CAMP CAP18 FALL39 HSD26 | Cathelicidin antimicrobial peptide (18 kDa cationic antimicrobial protein) (CAP-18) (hCAP-18) [Cleaved into: Antibacterial peptide FALL-39 (FALL-39 peptide antibiotic); Antibacterial peptide LL-37] | PDTPKP | 71 | 76 |
| **P50406** | HTR6 | 5-hydroxytryptamine receptor 6 (5-HT-6) (5-HT6) (Serotonin receptor 6) | PRTPRP | 240 | 245 |
| **P50440** | GATM AGAT | Glycine amidinotransferase, mitochondrial (EC 2.1.4.1) (L-arginine:glycine amidinotransferase) (Transamidinase) | PPTPIIP | 340 | 346 |
| **P50548** | ERF | ETS domain-containing transcription factor ERF (Ets2 repressor factor) (PE-2) | PETPPVP | 355 | 361 |
| **P50570** | DNM2 DYN2 | Dynamin-2 (EC 3.6.5.5) | PTPGPP | 787 | 792 |
| **P51168** | SCNN1B | Amiloride-sensitive sodium channel subunit beta (Beta-NaCH) (Epithelial Na(+) channel subunit beta) (Beta-ENaC) (ENaCB) (Nonvoltage-gated sodium channel 1 subunit beta) (SCNEB) | PGTPPP | 613 | 618 |
| **P51170** | SCNN1G | Amiloride-sensitive sodium channel subunit gamma (Epithelial Na(+) channel subunit gamma) (ENaCG) (Gamma-ENaC) (Gamma-NaCH) (Nonvoltage-gated sodium channel 1 subunit gamma) (SCNEG) | PGTPPP | 620 | 625 |
| **P51610** | HCFC1 HCF1 HFC1 | Host cell factor 1 (HCF) (HCF-1) (C1 factor) (CFF) (VCAF) (VP16 accessory protein) [Cleaved into: HCF N-terminal chain 1; HCF N-terminal chain 2; HCF N-terminal chain 3; HCF N-terminal chain 4; HCF N-terminal chain 5; HCF N-terminal chain 6; HCF C-terminal chain 1; HCF C-terminal chain 2; HCF C-terminal chain 3; HCF C-terminal chain 4; HCF C-terminal chain 5; HCF C-terminal chain 6] | PTPNP | 412 | 416 |
| **P51654** | GPC3 OCI5 | Glypican-3 (GTR2-2) (Intestinal protein OCI-5) (MXR7) [Cleaved into: Secreted glypican-3] | PETPVP | 53 | 58 |
| **P51671** | CCL11 SCYA11 | Eotaxin (C-C motif chemokine 11) (Eosinophil chemotactic protein) (Small-inducible cytokine A11) | PTPKP | 93 | 97 |
| **P51693** | APLP1 | Amyloid-like protein 1 (APLP) (APLP-1) [Cleaved into: C30] | PGTPDP | 213 | 218 |
| **P51888** | PRELP SLRR2A | Prolargin (Proline-arginine-rich end leucine-rich repeat protein) | PTPSFP | 42 | 47 |
| **P52630** | STAT2 | Signal transducer and activator of transcription 2 (p113) | PQTPHRP | 325 | 331 |
| **P52746** | ZNF142 KIAA0236 | Zinc finger protein 142 (HA4654) | PGTPAP | 537 | 542 |
| **P53814** | SMTN SMSMO | Smoothelin | PTPTP | 173 | 177 |
| **P54253** | ATXN1 ATX1 SCA1 | Ataxin-1 (Spinocerebellar ataxia type 1 protein) | PGTPVSP | 111 | 117 |
| **P54278** | PMS2 PMSL2 | Mismatch repair endonuclease PMS2 (EC 3.1.-.-) (DNA mismatch repair protein PMS2) (PMS1 protein homolog 2) | PKTPEP | 437 | 442 |
| **P54727** | RAD23B | UV excision repair protein RAD23 homolog B (HR23B) (hHR23B) (XP-C repair-complementing complex 58 kDa protein) (p58) | PTPVP | 111 | 115 |
| **P54829** | PTPN5 | Tyrosine-protein phosphatase non-receptor type 5 (EC 3.1.3.48) (Neural-specific protein-tyrosine phosphatase) (Striatum-enriched protein-tyrosine phosphatase) (STEP) | PPTPLPP | 172 | 178 |
| **P55001** | MFAP2 MAGP1 | Microfibrillar-associated protein 2 (MFAP-2) (Microfibril-associated glycoprotein 1) (MAGP) (MAGP-1) | PTPEP | 78 | 82 |
| **P55199** | ELL C19orf17 | RNA polymerase II elongation factor ELL (Eleven-nineteen lysine-rich leukemia protein) | PTPGPP | 367 | 372 |
| **P55211** | CASP9 MCH6 | Caspase-9 (CASP-9) (EC 3.4.22.62) (Apoptotic protease Mch-6) (Apoptotic protease-activating factor 3) (APAF-3) (ICE-like apoptotic protease 6) (ICE-LAP6) [Cleaved into: Caspase-9 subunit p35; Caspase-9 subunit p10] | PETPRP | 123 | 128 |
| **P55318** | FOXA3 HNF3G TCF3G | Hepatocyte nuclear factor 3-gamma (HNF-3-gamma) (HNF-3G) (Fork head-related protein FKH H3) (Forkhead box protein A3) (Transcription factor 3G) (TCF-3G) | PVTPVP | 27 | 32 |
| **P55771** | PAX9 | Paired box protein Pax-9 | PTPQP | 148 | 152 |
| **P56270** | MAZ ZNF801 | Myc-associated zinc finger protein (MAZI) (Pur-1) (Purine-binding transcription factor) (Serum amyloid A-activating factor-1) (SAF-1) (Transcription factor Zif87) (ZF87) (Zinc finger protein 801) | PPTPQAP | 70 | 76 |
| **P56945** | BCAR1 CAS CASS1 CRKAS | Breast cancer anti-estrogen resistance protein 1 (CRK-associated substrate) (Cas scaffolding protein family member 1) (p130cas) | PATPAQP | 79 | 85 |
| **P57071** | PRDM15 C21orf83 ZNF298 | PR domain zinc finger protein 15 (EC 2.1.1.-) (PR domain-containing protein 15) (Zinc finger protein 298) | PWTPNP | 125 | 130 |
| **P57789** | KCNK10 TREK2 | Potassium channel subfamily K member 10 (Outward rectifying potassium channel protein TREK-2) (TREK-2 K(+) channel subunit) | PTPTP | 37 | 41 |
| **P58180** | OR4D2 | Olfactory receptor 4D2 (B-lymphocyte membrane protein BC2009) (Olfactory receptor OR17-24) | PFTPFP | 261 | 266 |
| **P58658** | EVA1C C21orf63 C21orf64 FAM176C PRED34 UNQ2504/PRO5993 | Protein eva-1 homolog C (Protein FAM176C) (SUE21) | PPTPQP | 10 | 15 |
| **P59797** | SELV | Selenoprotein V (SelV) | PTPVP | 94 | 98 |
| **P60008** | HILS1 | Spermatid-specific linker histone H1-like protein | PTPAP | 41 | 45 |
| **P60852** | ZP1 | Zona pellucida sperm-binding protein 1 (Zona pellucida glycoprotein 1) (Zp-1) [Cleaved into: Processed zona pellucida sperm-binding protein 1] | PTPALP | 192 | 197 |
| **P61964** | WDR5 BIG3 | WD repeat-containing protein 5 (BMP2-induced 3-kb gene protein) | PTPVKP | 28 | 33 |
| **P62995** | TRA2B SFRS10 | Transformer-2 protein homolog beta (TRA-2 beta) (TRA2-beta) (hTRA2-beta) (Splicing factor, arginine/serine-rich 10) (Transformer-2 protein homolog B) | PHTPTP | 199 | 204 |
| **P68400** | CSNK2A1 CK2A1 | Casein kinase II subunit alpha (CK II alpha) (EC 2.7.11.1) | PTPSP | 359 | 363 |
| **P78312** | FAM193A C4orf8 RES4-22 | Protein FAM193A (Protein IT14) | PTPMEP | 1122 | 1127 |
| **P78314** | SH3BP2 3BP2 RES4-23 | SH3 domain-binding protein 2 (3BP-2) | PTPRKP | 210 | 215 |
| **P78357** | CNTNAP1 CASPR NRXN4 | Contactin-associated protein 1 (Caspr) (Caspr1) (Neurexin IV) (Neurexin-4) (p190) | PTPAP | 1361 | 1365 |
| **P78362** | SRPK2 | SRSF protein kinase 2 (EC 2.7.11.1) (SFRS protein kinase 2) (Serine/arginine-rich protein-specific kinase 2) (SR-protein-specific kinase 2) [Cleaved into: SRSF protein kinase 2 N-terminal; SRSF protein kinase 2 C-terminal] | PTPPEP | 40 | 45 |
| **P78424** | POU6F2 RPF1 | POU domain, class 6, transcription factor 2 (Retina-derived POU domain factor 1) (RPF-1) | PLTPPNP | 287 | 293 |
| **P78524** | ST5 DENND2B HTS1 | Suppression of tumorigenicity 5 protein (DENN domain-containing protein 2B) (HeLa tumor suppression 1) | PTPAAP | 414 | 419 |
| **P78540** | ARG2 | Arginase-2, mitochondrial (EC 3.5.3.1) (Kidney-type arginase) (Non-hepatic arginase) (Type II arginase) | PTPSSP | 338 | 343 |
| **P78559** | MAP1A MAP1L | Microtubule-associated protein 1A (MAP-1A) (Proliferation-related protein p80) [Cleaved into: MAP1A heavy chain; MAP1 light chain LC2] | PTPKSP | 1168 | 1173 |
| **P81877** | SSBP2 SSDP2 | Single-stranded DNA-binding protein 2 (Sequence-specific single-stranded-DNA-binding protein 2) | PGTPIMP | 250 | 256 |
| **P82673** | MRPS35 MRPS28 HDCMD11P MDS023 PSEC0213 | 28S ribosomal protein S35, mitochondrial (MRP-S35) (S35mt) (28S ribosomal protein S28, mitochondrial) (MRP-S28) (S28mt) | PTPSLP | 33 | 38 |
| **P83111** | LACTB MRPL56 UNQ843/PRO1781 | Serine beta-lactamase-like protein LACTB, mitochondrial (EC 3.4.-.-) | PQTPAPP | 93 | 99 |
| **P84022** | SMAD3 MADH3 | Mothers against decapentaplegic homolog 3 (MAD homolog 3) (Mad3) (Mothers against DPP homolog 3) (hMAD-3) (JV15-2) (SMAD family member 3) (SMAD 3) (Smad3) (hSMAD3) | PETPPP | 177 | 182 |
| **P84996** | GNAS GNAS1 | Protein ALEX (Alternative gene product encoded by XL-exon) | PTPGQP | 367 | 372 |
| **P98073** | TMPRSS15 ENTK PRSS7 | Enteropeptidase (EC 3.4.21.9) (Enterokinase) (Serine protease 7) (Transmembrane protease serine 15) [Cleaved into: Enteropeptidase non-catalytic heavy chain; Enteropeptidase catalytic light chain] | PTPPP | 514 | 518 |
| **P98082** | DAB2 DOC2 | Disabled homolog 2 (Adaptor molecule disabled-2) (Differentially expressed in ovarian carcinoma 2) (DOC-2) (Differentially-expressed protein 2) | PTPNP | 288 | 292 |
| **P98088** | MUC5AC MUC5 | Mucin-5AC (MUC-5AC) (Gastric mucin) (Lewis B blood group antigen) (LeB) (Major airway glycoprotein) (Mucin-5 subtype AC, tracheobronchial) (Tracheobronchial mucin) (TBM) | PTPPLP | 1229 | 1234 |
| **P98164** | LRP2 | Low-density lipoprotein receptor-related protein 2 (LRP-2) (Glycoprotein 330) (gp330) (Megalin) | PTPKP | 3923 | 3927 |
| **P98168** | ZXDA | Zinc finger X-linked protein ZXDA | PTPAP | 147 | 151 |
| **P98169** | ZXDB | Zinc finger X-linked protein ZXDB | PTPAP | 151 | 155 |
| **Q00056** | HOXA4 HOX1D | Homeobox protein Hox-A4 (Homeobox protein Hox-1.4) (Homeobox protein Hox-1D) | PATPGVP | 161 | 167 |
| **Q00653** | NFKB2 LYT10 | Nuclear factor NF-kappa-B p100 subunit (DNA-binding factor KBF2) (H2TF1) (Lymphocyte translocation chromosome 10 protein) (Nuclear factor of kappa light polypeptide gene enhancer in B-cells 2) (Oncogene Lyt-10) (Lyt10) [Cleaved into: Nuclear factor NF-kappa-B p52 subunit] | PLTPPSP | 757 | 763 |
| **Q00975** | CACNA1B CACH5 CACNL1A5 | Voltage-dependent N-type calcium channel subunit alpha-1B (Brain calcium channel III) (BIII) (Calcium channel, L type, alpha-1 polypeptide isoform 5) (Voltage-gated calcium channel subunit alpha Cav2.2) | PQTPLTP | 2191 | 2197 |
| **Q00G26** | PLIN5 LSDP5 OXPAT PAT-1 | Perilipin-5 (Lipid storage droplet protein 5) | PETPSCP | 446 | 452 |
| **Q01094** | E2F1 RBBP3 | Transcription factor E2F1 (E2F-1) (PBR3) (Retinoblastoma-associated protein 1) (RBAP-1) (Retinoblastoma-binding protein 3) (RBBP-3) (pRB-binding protein E2F-1) | PTPSAP | 74 | 79 |
| **Q01196** | RUNX1 AML1 CBFA2 | Runt-related transcription factor 1 (Acute myeloid leukemia 1 protein) (Core-binding factor subunit alpha-2) (CBF-alpha-2) (Oncogene AML-1) (Polyomavirus enhancer-binding protein 2 alpha B subunit) (PEA2-alpha B) (PEBP2-alpha B) (SL3-3 enhancer factor 1 alpha B subunit) (SL3/AKV core-binding factor alpha B subunit) | PTPNP | 218 | 222 |
| **Q01201** | RELB | Transcription factor RelB (I-Rel) | PATPPP | 101 | 106 |
| **Q01432** | AMPD3 | AMP deaminase 3 (EC 3.5.4.6) (AMP deaminase isoform E) (Erythrocyte AMP deaminase) | PTPAP | 120 | 124 |
| **Q01955** | COL4A3 | Collagen alpha-3(IV) chain (Goodpasture antigen) [Cleaved into: Tumstatin] | PGTPGFP | 752 | 758 |
| **Q01970** | PLCB3 | 1-phosphatidylinositol 4,5-bisphosphate phosphodiesterase beta-3 (EC 3.1.4.11) (Phosphoinositide phospholipase C-beta-3) (Phospholipase C-beta-3) (PLC-beta-3) | PTPSP | 908 | 912 |
| **Q02388** | COL7A1 | Collagen alpha-1(VII) chain (Long-chain collagen) (LC collagen) | PGTPGAP | 1316 | 1322 |
| **Q02410** | APBA1 MINT1 X11 | Amyloid beta A4 precursor protein-binding family A member 1 (Adapter protein X11alpha) (Neuron-specific X11 protein) (Neuronal Munc18-1-interacting protein 1) (Mint-1) | PYTPDEP | 368 | 374 |
| **Q02505** | MUC3A MUC3 | Mucin-3A (MUC-3A) (Intestinal mucin-3A) | PTPVTP | 2472 | 2477 |
| **Q02750** | MAP2K1 MEK1 PRKMK1 | Dual specificity mitogen-activated protein kinase kinase 1 (MAP kinase kinase 1) (MAPKK 1) (MKK1) (EC 2.7.12.2) (ERK activator kinase 1) (MAPK/ERK kinase 1) (MEK 1) | PRTPGRP | 290 | 296 |
| **Q02779** | MAP3K10 MLK2 MST | Mitogen-activated protein kinase kinase kinase 10 (EC 2.7.11.25) (Mixed lineage kinase 2) (Protein kinase MST) | PTPSAP | 654 | 659 |
| **Q02817** | MUC2 SMUC | Mucin-2 (MUC-2) (Intestinal mucin-2) | PTTPCVP | 1778 | 1784 |
| **Q02833** | RASSF7 C11orf13 HRC1 | Ras association domain-containing protein 7 (HRAS1-related cluster protein 1) | PVTPTP | 144 | 149 |
| **Q03001** | DST BP230 BP240 BPAG1 DMH DT KIAA0728 | Dystonin (230 kDa bullous pemphigoid antigen) (230/240 kDa bullous pemphigoid antigen) (Bullous pemphigoid antigen 1) (BPA) (Bullous pemphigoid antigen) (Dystonia musculorum protein) (Hemidesmosomal plaque protein) | PDTPEAP | 4587 | 4593 |
| **Q03014** | HHEX HEX PRH PRHX | Hematopoietically-expressed homeobox protein HHEX (Homeobox protein HEX) (Homeobox protein PRH) | PTPAP | 45 | 49 |
| **Q03112** | MECOM EVI1 | MDS1 and EVI1 complex locus protein EVI1 (Ecotropic virus integration site 1 protein homolog) (EVI-1) | PLTPVP | 565 | 570 |
| **Q03164** | KMT2A ALL1 CXXC7 HRX HTRX MLL MLL1 TRX1 | Histone-lysine N-methyltransferase 2A (Lysine N-methyltransferase 2A) (EC 2.1.1.43) (ALL-1) (CXXC-type zinc finger protein 7) (Myeloid/lymphoid or mixed-lineage leukemia) (Myeloid/lymphoid or mixed-lineage leukemia protein 1) (Trithorax-like protein) (Zinc finger protein HRX) [Cleaved into: MLL cleavage product N320 (N-terminal cleavage product of 320 kDa) (p320); MLL cleavage product C180 (C-terminal cleavage product of 180 kDa) (p180)] | PTPLFP | 832 | 837 |
| **Q03252** | LMNB2 LMN2 | Lamin-B2 | PATPLSP | 32 | 38 |
| **Q03468** | ERCC6 CSB | DNA excision repair protein ERCC-6 (EC 3.6.4.-) (ATP-dependent helicase ERCC6) (Cockayne syndrome protein CSB) | PVTPPAP | 301 | 307 |
| **Q04609** | FOLH1 FOLH NAALAD1 PSM PSMA GIG27 | Glutamate carboxypeptidase 2 (EC 3.4.17.21) (Cell growth-inhibiting gene 27 protein) (Folate hydrolase 1) (Folylpoly-gamma-glutamate carboxypeptidase) (FGCP) (Glutamate carboxypeptidase II) (GCPII) (Membrane glutamate carboxypeptidase) (mGCP) (N-acetylated-alpha-linked acidic dipeptidase I) (NAALADase I) (Prostate-specific membrane antigen) (PSM) (PSMA) (Pteroylpoly-gamma-glutamate carboxypeptidase) | PLTPGYP | 267 | 273 |
| **Q04637** | EIF4G1 EIF4F EIF4G EIF4GI | Eukaryotic translation initiation factor 4 gamma 1 (eIF-4-gamma 1) (eIF-4G 1) (eIF-4G1) (p220) | PTPSP | 264 | 268 |
| **Q04724** | TLE1 | Transducin-like enhancer protein 1 (E(Sp1) homolog) (Enhancer of split groucho-like protein 1) (ESG1) | PLTPHP | 142 | 147 |
| **Q04725** | TLE2 | Transducin-like enhancer protein 2 (Enhancer of split groucho-like protein 2) (ESG2) | PLTPRP | 137 | 142 |
| **Q04727** | TLE4 GRG4 KIAA1261 | Transducin-like enhancer protein 4 (Grg-4) (Groucho-related protein 4) | PLTPHP | 147 | 152 |
| **Q05215** | EGR4 | Early growth response protein 4 (EGR-4) (AT133) | PTPPP | 177 | 181 |
| **Q05469** | LIPE | Hormone-sensitive lipase (HSL) (EC 3.1.1.79) | PITPLEP | 21 | 27 |
| **Q05513** | PRKCZ PKC2 | Protein kinase C zeta type (EC 2.7.11.13) (nPKC-zeta) | PSTPEQP | 97 | 103 |
| **Q05519** | SRSF11 SFRS11 | Serine/arginine-rich splicing factor 11 (Arginine-rich 54 kDa nuclear protein) (p54) (Splicing factor, arginine/serine-rich 11) | PTPNP | 142 | 146 |
| **Q05707** | COL14A1 UND | Collagen alpha-1(XIV) chain (Undulin) | PDTPQEP | 1296 | 1302 |
| **Q05C16** | LRRC63 | Leucine-rich repeat-containing protein 63 | PTPVLP | 240 | 245 |
| **Q05DH4** | FAM160A1 | Protein FAM160A1 | PTPDP | 775 | 779 |
| **Q06124** | PTPN11 PTP2C SHPTP2 | Tyrosine-protein phosphatase non-receptor type 11 (EC 3.1.3.48) (Protein-tyrosine phosphatase 1D) (PTP-1D) (Protein-tyrosine phosphatase 2C) (PTP-2C) (SH-PTP2) (SHP-2) (Shp2) (SH-PTP3) | PCTPTPP | 566 | 572 |
| **Q06190** | PPP2R3A PPP2R3 | Serine/threonine-protein phosphatase 2A regulatory subunit B'' subunit alpha (PP2A subunit B isoform PR72/PR130) (PP2A subunit B isoform R3 isoform) (PP2A subunit B isoforms B''-PR72/PR130) (PP2A subunit B isoforms B72/B130) (Serine/threonine-protein phosphatase 2A 72/130 kDa regulatory subunit B) | PGTPLPP | 674 | 680 |
| **Q06330** | RBPJ IGKJRB IGKJRB1 RBPJK RBPSUH | Recombining binding protein suppressor of hairless (CBF-1) (J kappa-recombination signal-binding protein) (RBP-J kappa) (RBP-J) (RBP-JK) (Renal carcinoma antigen NY-REN-30) | PVTPVP | 350 | 355 |
| **Q06455** | RUNX1T1 AML1T1 CBFA2T1 CDR ETO MTG8 ZMYND2 | Protein CBFA2T1 (Cyclin-D-related protein) (Eight twenty one protein) (Protein ETO) (Protein MTG8) (Zinc finger MYND domain-containing protein 2) | PTPPPP | 292 | 297 |
| **Q06481** | APLP2 APPL2 | Amyloid-like protein 2 (APLP-2) (APPH) (Amyloid protein homolog) (CDEI box-binding protein) (CDEBP) | PPTPLP | 365 | 370 |
| **Q06495** | SLC34A1 NPT2 SLC17A2 | Sodium-dependent phosphate transport protein 2A (Sodium-phosphate transport protein 2A) (Na(+)-dependent phosphate cotransporter 2A) (NaPi-3) (Sodium/phosphate cotransporter 2A) (Na(+)/Pi cotransporter 2A) (NaPi-2a) (Solute carrier family 34 member 1) | PATPSP | 621 | 626 |
| **Q07092** | COL16A1 FP1572 | Collagen alpha-1(XVI) chain | PGTPGDP | 463 | 469 |
| **Q07157** | TJP1 ZO1 | Tight junction protein ZO-1 (Tight junction protein 1) (Zona occludens protein 1) (Zonula occludens protein 1) | PTPAP | 959 | 963 |
| **Q07666** | KHDRBS1 SAM68 | KH domain-containing, RNA-binding, signal transduction-associated protein 1 (GAP-associated tyrosine phosphoprotein p62) (Src-associated in mitosis 68 kDa protein) (Sam68) (p21 Ras GTPase-activating protein-associated p62) (p68) | PTPLLP | 83 | 88 |
| **Q07820** | MCL1 BCL2L3 | Induced myeloid leukemia cell differentiation protein Mcl-1 (Bcl-2-like protein 3) (Bcl2-L-3) (Bcl-2-related protein EAT/mcl1) (mcl1/EAT) | PSTPPP | 161 | 166 |
| **Q07889** | SOS1 | Son of sevenless homolog 1 (SOS-1) | PRTPLTP | 1083 | 1089 |
| **Q07890** | SOS2 | Son of sevenless homolog 2 (SOS-2) | PNTPSTP | 1086 | 1092 |
| **Q07912** | TNK2 ACK1 | Activated CDC42 kinase 1 (ACK-1) (EC 2.7.10.2) (EC 2.7.11.1) (Tyrosine kinase non-receptor protein 2) | PTPLP | 885 | 889 |
| **Q07954** | LRP1 A2MR APR | Prolow-density lipoprotein receptor-related protein 1 (LRP-1) (Alpha-2-macroglobulin receptor) (A2MR) (Apolipoprotein E receptor) (APOER) (CD antigen CD91) [Cleaved into: Low-density lipoprotein receptor-related protein 1 85 kDa subunit (LRP-85); Low-density lipoprotein receptor-related protein 1 515 kDa subunit (LRP-515); Low-density lipoprotein receptor-related protein 1 intracellular domain (LRPICD)] | PTPPP | 4188 | 4192 |
| **Q08050** | FOXM1 FKHL16 HFH11 MPP2 WIN | Forkhead box protein M1 (Forkhead-related protein FKHL16) (Hepatocyte nuclear factor 3 forkhead homolog 11) (HFH-11) (HNF-3/fork-head homolog 11) (M-phase phosphoprotein 2) (MPM-2 reactive phosphoprotein 2) (Transcription factor Trident) (Winged-helix factor from INS-1 cells) | PTPRP | 509 | 513 |
| **Q08117** | AES GRG GRG5 | Amino-terminal enhancer of split (Amino enhancer of split) (Gp130-associated protein GAM) (Grg-5) (Groucho-related protein 5) (Protein ESP1) (Protein GRG) | PLTPLP | 141 | 146 |
| **Q08378** | GOLGA3 | Golgin subfamily A member 3 (Golgi complex-associated protein of 170 kDa) (GCP170) (Golgin-160) | PTPPFP | 71 | 76 |
| **Q08AD1** | CAMSAP2 CAMSAP1L1 KIAA1078 | Calmodulin-regulated spectrin-associated protein 2 (Calmodulin-regulated spectrin-associated protein 1-like protein 1) | PHTPQP | 567 | 572 |
| **Q08AE8** | SPIRE1 KIAA1135 SPIR1 | Protein spire homolog 1 (Spir-1) | PTPPRP | 361 | 366 |
| **Q09161** | NCBP1 CBP80 NCBP | Nuclear cap-binding protein subunit 1 (80 kDa nuclear cap-binding protein) (CBP80) (NCBP 80 kDa subunit) | PFTPPP | 270 | 275 |
| **Q09428** | ABCC8 HRINS SUR SUR1 | ATP-binding cassette sub-family C member 8 (Sulfonylurea receptor 1) | PTPQGP | 629 | 634 |
| **Q09472** | EP300 P300 | Histone acetyltransferase p300 (p300 HAT) (EC 2.3.1.48) (E1A-associated protein p300) | PTPDP | 593 | 597 |
| **Q10713** | PMPCA INPP5E KIAA0123 MPPA | Mitochondrial-processing peptidase subunit alpha (EC 3.4.24.64) (Alpha-MPP) (P-55) | PTPIP | 311 | 315 |
| **Q12774** | ARHGEF5 TIM | Rho guanine nucleotide exchange factor 5 (Ephexin-3) (Guanine nucleotide regulatory protein TIM) (Oncogene TIM) (Transforming immortalized mammary oncogene) (p60 TIM) | PPTPDLP | 814 | 820 |
| **Q12830** | BPTF FAC1 FALZ | Nucleosome-remodeling factor subunit BPTF (Bromodomain and PHD finger-containing transcription factor) (Fetal Alz-50 clone 1 protein) (Fetal Alzheimer antigen) | PVTPAPP | 2798 | 2804 |
| **Q12857** | NFIA KIAA1439 | Nuclear factor 1 A-type (NF1-A) (Nuclear factor 1/A) (CCAAT-box-binding transcription factor) (CTF) (Nuclear factor I/A) (NF-I/A) (NFI-A) (TGGCA-binding protein) | PTPMLP | 433 | 438 |
| **Q12873** | CHD3 | Chromodomain-helicase-DNA-binding protein 3 (CHD-3) (EC 3.6.4.12) (ATP-dependent helicase CHD3) (Mi-2 autoantigen 240 kDa protein) (Mi2-alpha) (Zinc finger helicase) (hZFH) | PATPAP | 1555 | 1560 |
| **Q12929** | EPS8 | Epidermal growth factor receptor kinase substrate 8 | PPTPAP | 627 | 632 |
| **Q12948** | FOXC1 FKHL7 FREAC3 | Forkhead box protein C1 (Forkhead-related protein FKHL7) (Forkhead-related transcription factor 3) (FREAC-3) | PYTPQP | 66 | 71 |
| **Q12955** | ANK3 | Ankyrin-3 (ANK-3) (Ankyrin-G) | PLTPETP | 3166 | 3172 |
| **Q12967** | RALGDS KIAA1308 RGF | Ral guanine nucleotide dissociation stimulator (RalGDS) (Ral guanine nucleotide exchange factor) (RalGEF) | PTPAP | 297 | 301 |
| **Q13061** | TRDN | Triadin | PTPASP | 302 | 307 |
| **Q13111** | CHAF1A CAF CAF1P150 | Chromatin assembly factor 1 subunit A (CAF-1 subunit A) (Chromatin assembly factor I p150 subunit) (CAF-I 150 kDa subunit) (CAF-I p150) (hp150) | PKTPQAP | 452 | 458 |
| **Q13118** | KLF10 TIEG TIEG1 | Krueppel-like factor 10 (EGR-alpha) (Transforming growth factor-beta-inducible early growth response protein 1) (TGFB-inducible early growth response protein 1) (TIEG-1) | PPTPAP | 473 | 478 |
| **Q13164** | MAPK7 BMK1 ERK5 PRKM7 | Mitogen-activated protein kinase 7 (MAP kinase 7) (MAPK 7) (EC 2.7.11.24) (Big MAP kinase 1) (BMK-1) (Extracellular signal-regulated kinase 5) (ERK-5) | PTPTP | 593 | 597 |
| **Q13202** | DUSP8 C11orf81 VH5 | Dual specificity protein phosphatase 8 (EC 3.1.3.16) (EC 3.1.3.48) (Dual specificity protein phosphatase hVH-5) | PGTPSP | 515 | 520 |
| **Q13286** | CLN3 BTS | Battenin (Batten disease protein) (Protein CLN3) | PTPIP | 79 | 83 |
| **Q13370** | PDE3B | cGMP-inhibited 3',5'-cyclic phosphodiesterase B (EC 3.1.4.17) (CGIPDE1) (CGIP1) (Cyclic GMP-inhibited phosphodiesterase B) (CGI-PDE B) | PLTPFP | 400 | 405 |
| **Q13434** | MKRN4P MKRN4 MKRNP5 RNF64 ZNF127L1 | Putative E3 ubiquitin-protein ligase makorin-4 (EC 6.3.2.-) (Makorin RING finger protein pseudogene 4) (Makorin RING finger protein pseudogene 5) (RING finger protein 64) (Zinc finger protein 127-Xp) (ZNF127-Xp) (Zinc finger protein 127-like 1) | PTPIP | 32 | 36 |
| **Q13459** | MYO9B MYR5 | Unconventional myosin-IXb (Unconventional myosin-9b) | PTPSP | 2032 | 2036 |
| **Q13461** | FOXE3 FKHL12 FREAC8 | Forkhead box protein E3 (Forkhead-related protein FKHL12) (Forkhead-related transcription factor 8) (FREAC-8) | PTPAP | 53 | 57 |
| **Q13485** | SMAD4 DPC4 MADH4 | Mothers against decapentaplegic homolog 4 (MAD homolog 4) (Mothers against DPP homolog 4) (Deletion target in pancreatic carcinoma 4) (SMAD family member 4) (SMAD 4) (Smad4) (hSMAD4) | PYTPNLP | 275 | 281 |
| **Q13487** | SNAPC2 SNAP45 | snRNA-activating protein complex subunit 2 (SNAPc subunit 2) (Proximal sequence element-binding transcription factor subunit delta) (PSE-binding factor subunit delta) (PTF subunit delta) (Small nuclear RNA-activating complex polypeptide 2) (snRNA-activating protein complex 45 kDa subunit) (SNAPc 45 kDa subunit) | PRTPDP | 179 | 184 |
| **Q13508** | ART3 TMART | Ecto-ADP-ribosyltransferase 3 (EC 2.4.2.31) (ADP-ribosyltransferase C2 and C3 toxin-like 3) (ARTC3) (Mono(ADP-ribosyl)transferase 3) (NAD(P)(+)--arginine ADP-ribosyltransferase 3) | PTPGP | 345 | 349 |
| **Q13530** | SERINC3 DIFF33 TDE1 SBBI99 | Serine incorporator 3 (Tumor differentially expressed protein 1) | PTPTPP | 321 | 326 |
| **Q13574** | DGKZ DAGK6 | Diacylglycerol kinase zeta (DAG kinase zeta) (EC 2.7.1.107) (Diglyceride kinase zeta) (DGK-zeta) | PPTPGAP | 258 | 264 |
| **Q13586** | STIM1 GOK | Stromal interaction molecule 1 | PDTPSP | 624 | 629 |
| **Q13591** | SEMA5A SEMAF | Semaphorin-5A (Semaphorin-F) (Sema F) | PWTPWTP | 709 | 715 |
| **Q13635** | PTCH1 PTCH | Protein patched homolog 1 (PTC) (PTC1) | PTPSP | 1194 | 1198 |
| **Q13670** | PMS2P11 PMS2L11 PMSR6 | Putative postmeiotic segregation increased 2-like protein 11 (PMS2-related protein 6) (Putative postmeiotic segregation increased 2 pseudogene 11) | PPTPTP | 259 | 264 |
| **Q13761** | RUNX3 AML2 CBFA3 PEBP2A3 | Runt-related transcription factor 3 (Acute myeloid leukemia 2 protein) (Core-binding factor subunit alpha-3) (CBF-alpha-3) (Oncogene AML-2) (Polyomavirus enhancer-binding protein 2 alpha C subunit) (PEA2-alpha C) (PEBP2-alpha C) (SL3-3 enhancer factor 1 alpha C subunit) (SL3/AKV core-binding factor alpha C subunit) | PSTPSP | 210 | 215 |
| **Q13796** | SHROOM2 APXL | Protein Shroom2 (Apical-like protein) (Protein APXL) | PTPSP | 1095 | 1099 |
| **Q14028** | CNGB1 CNCG2 CNCG3L CNCG4 RCNC2 | Cyclic nucleotide-gated cation channel beta-1 (Cyclic nucleotide-gated cation channel 4) (CNG channel 4) (CNG-4) (CNG4) (Cyclic nucleotide-gated cation channel gamma) (Cyclic nucleotide-gated cation channel modulatory subunit) (Cyclic nucleotide-gated channel beta-1) (CNG channel beta-1) (Glutamic acid-rich protein) (GARP) | PTPIP | 216 | 220 |
| **Q14031** | COL4A6 | Collagen alpha-6(IV) chain | PGTPGFP | 669 | 675 |
| **Q14112** | NID2 | Nidogen-2 (NID-2) (Osteonidogen) | PTPGNP | 757 | 762 |
| **Q14118** | DAG1 | Dystroglycan (Dystrophin-associated glycoprotein 1) [Cleaved into: Alpha-dystroglycan (Alpha-DG); Beta-dystroglycan (Beta-DG)] | PTPTSP | 340 | 345 |
| **Q14126** | DSG2 CDHF5 | Desmoglein-2 (Cadherin family member 5) (HDGC) | PTPIP | 370 | 374 |
| **Q14140** | SERTAD2 KIAA0127 | SERTA domain-containing protein 2 (Transcriptional regulator interacting with the PHD-bromodomain 2) (TRIP-Br2) | PVTPSQP | 290 | 296 |
| **Q14146** | URB2 KIAA0133 | Unhealthy ribosome biogenesis protein 2 homolog | PDTPGP | 588 | 593 |
| **Q14147** | DHX34 DDX34 KIAA0134 | Probable ATP-dependent RNA helicase DHX34 (EC 3.6.4.13) (DEAH box protein 34) | PATPHLP | 1018 | 1024 |
| **Q14155** | ARHGEF7 COOL1 KIAA0142 P85SPR PAK3BP PIXB Nbla10314 | Rho guanine nucleotide exchange factor 7 (Beta-Pix) (COOL-1) (PAK-interacting exchange factor beta) (p85) | PKTPKP | 644 | 649 |
| **Q14157** | UBAP2L KIAA0144 NICE4 | Ubiquitin-associated protein 2-like (Protein NICE-4) | PTPTTP | 840 | 845 |
| **Q14160** | SCRIB CRIB1 KIAA0147 LAP4 SCRB1 VARTUL | Protein scribble homolog (Scribble) (hScrib) (Protein LAP4) | PPTPGP | 1340 | 1345 |
| **Q14165** | MLEC KIAA0152 | Malectin | PRTPNP | 258 | 263 |
| **Q14185** | DOCK1 | Dedicator of cytokinesis protein 1 (180 kDa protein downstream of CRK) (DOCK180) | PTPPPP | 1824 | 1829 |
| **Q14188** | TFDP2 DP2 | Transcription factor Dp-2 (E2F dimerization partner 2) | PYTPAP | 81 | 86 |
| **Q14190** | SIM2 BHLHE15 | Single-minded homolog 2 (Class E basic helix-loop-helix protein 15) (bHLHe15) | PPTPEAP | 583 | 589 |
| **Q14202** | ZMYM3 DXS6673E KIAA0385 ZNF261 | Zinc finger MYM-type protein 3 (Zinc finger protein 261) | PTPPPP | 816 | 821 |
| **Q14207** | NPAT CAND3 E14 | Protein NPAT (Nuclear protein of the ataxia telangiectasia mutated locus) (Nuclear protein of the ATM locus) (p220) | PVTPDLP | 1308 | 1314 |
| **Q14209** | E2F2 | Transcription factor E2F2 (E2F-2) | PKTPKSP | 118 | 124 |
| **Q14244** | MAP7 | Ensconsin (Epithelial microtubule-associated protein of 115 kDa) (E-MAP-115) (Microtubule-associated protein 7) (MAP-7) | PGTPRP | 340 | 345 |
| **Q14451** | GRB7 | Growth factor receptor-bound protein 7 (B47) (Epidermal growth factor receptor GRB-7) (GRB7 adapter protein) | PGTPRPP | 25 | 31 |
| **Q14541** | HNF4G NR2A2 | Hepatocyte nuclear factor 4-gamma (HNF-4-gamma) (Nuclear receptor subfamily 2 group A member 2) | PETPLP | 371 | 376 |
| **Q14653** | IRF3 | Interferon regulatory factor 3 (IRF-3) | PTPFP | 179 | 183 |
| **Q14674** | ESPL1 ESP1 KIAA0165 | Separin (EC 3.4.22.49) (Caspase-like protein ESPL1) (Extra spindle poles-like 1 protein) (Separase) | PCTPKPP | 1344 | 1350 |
| **Q14676** | MDC1 KIAA0170 NFBD1 | Mediator of DNA damage checkpoint protein 1 (Nuclear factor with BRCT domains 1) | PTPEP | 965 | 969 |
| **Q14686** | NCOA6 AIB3 KIAA0181 RAP250 TRBP | Nuclear receptor coactivator 6 (Activating signal cointegrator 2) (ASC-2) (Amplified in breast cancer protein 3) (Cancer-amplified transcriptional coactivator ASC-2) (Nuclear receptor coactivator RAP250) (NRC RAP250) (Nuclear receptor-activating protein, 250 kDa) (Peroxisome proliferator-activated receptor-interacting protein) (PPAR-interacting protein) (PRIP) (Thyroid hormone receptor-binding protein) | PRTPRP | 205 | 210 |
| **Q14687** | GSE1 KIAA0182 | Genetic suppressor element 1 | PLTPSP | 28 | 33 |
| **Q14693** | LPIN1 KIAA0188 | Phosphatidate phosphatase LPIN1 (EC 3.1.3.4) (Lipin-1) | PTPSP | 238 | 242 |
| **Q14839** | CHD4 | Chromodomain-helicase-DNA-binding protein 4 (CHD-4) (EC 3.6.4.12) (ATP-dependent helicase CHD4) (Mi-2 autoantigen 218 kDa protein) (Mi2-beta) | PTPVP | 516 | 520 |
| **Q14863** | POU6F1 BRN5 MPOU TCFB1 | POU domain, class 6, transcription factor 1 (Brain-specific homeobox/POU domain protein 5) (Brain-5) (Brn-5) (mPOU homeobox protein) | PSTPESP | 73 | 79 |
| **Q14865** | ARID5B DESRT MRF2 | AT-rich interactive domain-containing protein 5B (ARID domain-containing protein 5B) (MRF1-like protein) (Modulator recognition factor 2) (MRF-2) | PTPPLP | 538 | 543 |
| **Q14896** | MYBPC3 | Myosin-binding protein C, cardiac-type (Cardiac MyBP-C) (C-protein, cardiac muscle isoform) | PTPGAP | 145 | 150 |
| **Q14919** | DRAP1 | Dr1-associated corepressor (Dr1-associated protein 1) (Negative cofactor 2-alpha) (NC2-alpha) | PPTPFLP | 172 | 178 |
| **Q14934** | NFATC4 NFAT3 | Nuclear factor of activated T-cells, cytoplasmic 4 (NF-ATc4) (NFATc4) (T-cell transcription factor NFAT3) (NF-AT3) | PTPEPP | 123 | 128 |
| **Q14957** | GRIN2C NMDAR2C | Glutamate receptor ionotropic, NMDA 2C (GluN2C) (Glutamate [NMDA] receptor subunit epsilon-3) (N-methyl D-aspartate receptor subtype 2C) (NMDAR2C) (NR2C) | PTPDPP | 947 | 952 |
| **Q14993** | COL19A1 | Collagen alpha-1(XIX) chain (Collagen alpha-1(Y) chain) | PGTPGTP | 652 | 658 |
| **Q14CN2** | CLCA4 CaCC2 UNQ562/PRO1124 | Calcium-activated chloride channel regulator 4 (EC 3.4.-.-) (Calcium-activated chloride channel family member 4) (hCLCA4) (Calcium-activated chloride channel protein 2) (CaCC-2) (hCaCC-2) (Chloride channel accessory 4) [Cleaved into: Calcium-activated chloride channel regulator 4, 110 kDa form; Calcium-activated chloride channel regulator 4, 30 kDa form] | PTPTP | 877 | 881 |
| **Q15031** | LARS2 KIAA0028 | Probable leucine--tRNA ligase, mitochondrial (EC 6.1.1.4) (Leucyl-tRNA synthetase) (LeuRS) | PTPVP | 469 | 473 |
| **Q15035** | TRAM2 KIAA0057 | Translocating chain-associated membrane protein 2 | PATPRLP | 332 | 338 |
| **Q15053** | KIAA0040 | Uncharacterized protein KIAA0040 | PHTPSIP | 147 | 153 |
| **Q15058** | KIF14 KIAA0042 | Kinesin-like protein KIF14 | PLTPNP | 79 | 84 |
| **Q15059** | BRD3 KIAA0043 RING3L | Bromodomain-containing protein 3 (RING3-like protein) | PATPGP | 13 | 18 |
| **Q15119** | PDK2 PDHK2 | [Pyruvate dehydrogenase (acetyl-transferring)] kinase isozyme 2, mitochondrial (EC 2.7.11.2) (Pyruvate dehydrogenase kinase isoform 2) (PDH kinase 2) (PDKII) | PTPQP | 312 | 316 |
| **Q15149** | PLEC PLEC1 | Plectin (PCN) (PLTN) (Hemidesmosomal protein 1) (HD1) (Plectin-1) | PETPVVP | 150 | 156 |
| **Q15223** | NECTIN1 HVEC PRR1 PVRL1 | Nectin-1 (Herpes virus entry mediator C) (Herpesvirus entry mediator C) (HveC) (Herpesvirus Ig-like receptor) (HIgR) (Nectin cell adhesion molecule 1) (Poliovirus receptor-related protein 1) (CD antigen CD111) | PYTPSPP | 337 | 343 |
| **Q15323** | KRT31 HHA1 HKA1 KRTHA1 | Keratin, type I cuticular Ha1 (Hair keratin, type I Ha1) (Keratin-31) (K31) | PCTPCAP | 398 | 404 |
| **Q15334** | LLGL1 DLG4 HUGL HUGL1 | Lethal(2) giant larvae protein homolog 1 (LLGL) (DLG4) (Hugl-1) (Human homolog to the D-lgl gene protein) | PDTPEPP | 998 | 1004 |
| **Q15427** | SF3B4 SAP49 | Splicing factor 3B subunit 4 (Pre-mRNA-splicing factor SF3b 49 kDa subunit) (SF3b50) (Spliceosome-associated protein 49) (SAP 49) | PTPRPP | 406 | 411 |
| **Q15428** | SF3A2 SAP62 | Splicing factor 3A subunit 2 (SF3a66) (Spliceosome-associated protein 62) (SAP 62) | PPTPMPP | 437 | 443 |
| **Q15459** | SF3A1 SAP114 | Splicing factor 3A subunit 1 (SF3a120) (Spliceosome-associated protein 114) (SAP 114) | PETPMPP | 372 | 378 |
| **Q15468** | STIL SIL | SCL-interrupting locus protein (TAL-1-interrupting locus protein) | PTPPLP | 590 | 595 |
| **Q15477** | SKIV2L DDX13 SKI2W SKIV2 W | Helicase SKI2W (Ski2) (EC 3.6.4.-) (Helicase-like protein) (HLP) | PTPAP | 202 | 206 |
| **Q15562** | TEAD2 TEF4 | Transcriptional enhancer factor TEF-4 (TEA domain family member 2) (TEAD-2) | PPTPSPP | 210 | 216 |
| **Q15572** | TAF1C | TATA box-binding protein-associated factor RNA polymerase I subunit C (RNA polymerase I-specific TBP-associated factor 110 kDa) (TAFI110) (TATA box-binding protein-associated factor 1C) (TBP-associated factor 1C) (Transcription initiation factor SL1/TIF-IB subunit C) | PATPGP | 60 | 65 |
| **Q15643** | TRIP11 CEV14 | Thyroid receptor-interacting protein 11 (TR-interacting protein 11) (TRIP-11) (Clonal evolution-related gene on chromosome 14 protein) (Golgi-associated microtubule-binding protein 210) (GMAP-210) (Trip230) | PNTPLRP | 1844 | 1850 |
| **Q15735** | INPP5J PIB5PA PIPP | Phosphatidylinositol 4,5-bisphosphate 5-phosphatase A (EC 3.1.3.56) (Inositol polyphosphate 5-phosphatase J) | PSTPSP | 199 | 204 |
| **Q15744** | CEBPE | CCAAT/enhancer-binding protein epsilon (C/EBP epsilon) | PGTPAFP | 72 | 78 |
| **Q15750** | TAB1 MAP3K7IP1 | TGF-beta-activated kinase 1 and MAP3K7-binding protein 1 (Mitogen-activated protein kinase kinase kinase 7-interacting protein 1) (TGF-beta-activated kinase 1-binding protein 1) (TAK1-binding protein 1) | PTPSP | 375 | 379 |
| **Q15788** | NCOA1 BHLHE74 SRC1 | Nuclear receptor coactivator 1 (NCoA-1) (EC 2.3.1.48) (Class E basic helix-loop-helix protein 74) (bHLHe74) (Protein Hin-2) (RIP160) (Renal carcinoma antigen NY-REN-52) (Steroid receptor coactivator 1) (SRC-1) | PTPAQP | 1304 | 1309 |
| **Q15796** | SMAD2 MADH2 MADR2 | Mothers against decapentaplegic homolog 2 (MAD homolog 2) (Mothers against DPP homolog 2) (JV18-1) (Mad-related protein 2) (hMAD-2) (SMAD family member 2) (SMAD 2) (Smad2) (hSMAD2) | PETPPP | 218 | 223 |
| **Q15911** | ZFHX3 ATBF1 | Zinc finger homeobox protein 3 (AT motif-binding factor 1) (AT-binding transcription factor 1) (Alpha-fetoprotein enhancer-binding protein) (Zinc finger homeodomain protein 3) (ZFH-3) | PQTPEPP | 2034 | 2040 |
| **Q16204** | CCDC6 D10S170 TST1 | Coiled-coil domain-containing protein 6 (Papillary thyroid carcinoma-encoded protein) (Protein H4) | PYTPSP | 355 | 360 |
| **Q16206** | ENOX2 COVA1 | Ecto-NOX disulfide-thiol exchanger 2 (APK1 antigen) (Cytosolic ovarian carcinoma antigen 1) (Tumor-associated hydroquinone oxidase) (tNOX) [Includes: Hydroquinone [NADH] oxidase (EC 1.-.-.-); Protein disulfide-thiol oxidoreductase (EC 1.-.-.-)] | PITPMMP | 75 | 81 |
| **Q16445** | GABRA6 | Gamma-aminobutyric acid receptor subunit alpha-6 (GABA(A) receptor subunit alpha-6) | PVTPPP | 401 | 406 |
| **Q16513** | PKN2 PRK2 PRKCL2 | Serine/threonine-protein kinase N2 (EC 2.7.11.13) (PKN gamma) (Protein kinase C-like 2) (Protein-kinase C-related kinase 2) | PRTPDTP | 119 | 125 |
| **Q16584** | MAP3K11 MLK3 PTK1 SPRK | Mitogen-activated protein kinase kinase kinase 11 (EC 2.7.11.25) (Mixed lineage kinase 3) (Src-homology 3 domain-containing proline-rich kinase) | PTTPPTP | 675 | 681 |
| **Q16643** | DBN1 D0S117E | Drebrin (Developmentally-regulated brain protein) | PTPIP | 330 | 334 |
| **Q16666** | IFI16 IFNGIP1 | Gamma-interferon-inducible protein 16 (Ifi-16) (Interferon-inducible myeloid differentiation transcriptional activator) | PHTPQMP | 482 | 488 |
| **Q16690** | DUSP5 VH3 | Dual specificity protein phosphatase 5 (EC 3.1.3.16) (EC 3.1.3.48) (Dual specificity protein phosphatase hVH3) | PSTPNP | 319 | 324 |
| **Q16696** | CYP2A13 | Cytochrome P450 2A13 (EC 1.14.14.1) (CYPIIA13) | PTPLP | 37 | 41 |
| **Q16787** | LAMA3 LAMNA | Laminin subunit alpha-3 (Epiligrin 170 kDa subunit) (E170) (Epiligrin subunit alpha) (Kalinin subunit alpha) (Laminin-5 subunit alpha) (Laminin-6 subunit alpha) (Laminin-7 subunit alpha) (Nicein subunit alpha) | PTPAHP | 927 | 932 |
| **Q16799** | RTN1 NSP | Reticulon-1 (Neuroendocrine-specific protein) | PETPMLP | 545 | 551 |
| **Q17R89** | ARHGAP44 KIAA0672 RICH2 | Rho GTPase-activating protein 44 (NPC-A-10) (Rho-type GTPase-activating protein RICH2) (RhoGAP interacting with CIP4 homologs protein 2) (RICH-2) | PSTPSP | 684 | 689 |
| **Q1RMZ1** | C7orf60 | Probable methyltransferase BTM2 homolog (EC 2.1.1.-) | PNTPPP | 19 | 24 |
| **Q1W209** | ESRG HESRG | Embryonic stem cell-related gene protein (hES cell-related gene protein) | PKTPAP | 138 | 143 |
| **Q1W6H9** | FAM110C | Protein FAM110C | PTTPGP | 153 | 158 |
| **Q2KHR2** | RFX7 RFXDC2 | DNA-binding protein RFX7 (Regulatory factor X 7) (Regulatory factor X domain-containing protein 2) | PTPTP | 851 | 855 |
| **Q2M296** | MTHFSD | Methenyltetrahydrofolate synthase domain-containing protein | PDTPGP | 280 | 285 |
| **Q2M2I3** | FAM83E | Protein FAM83E | PATPGP | 343 | 348 |
| **Q2M3G4** | SHROOM1 APXL2 KIAA1960 | Protein Shroom1 (Apical protein 2) | PGTPGP | 101 | 106 |
| **Q2M3V2** | SOWAHA ANKRD43 | Ankyrin repeat domain-containing protein SOWAHA (Ankyrin repeat domain-containing protein 43) (Protein sosondowah homolog A) | PTPGP | 533 | 537 |
| **Q2MV58** | TCTN1 TECT1 UNQ9369/PRO34160 | Tectonic-1 | PGTPRAP | 54 | 60 |
| **Q2NKJ3** | CTC1 C17orf68 | CST complex subunit CTC1 (Conserved telomere maintenance component 1) (HBV DNAPTP1-transactivated protein B) | PVTPIP | 197 | 202 |
| **Q2NKQ1** | SGSM1 KIAA1941 RUTBC2 | Small G protein signaling modulator 1 (RUN and TBC1 domain-containing protein 2) | PTPSP | 476 | 480 |
| **Q2NL68** | PROSER3 C19orf55 | Proline and serine-rich protein 3 | PTPAP | 267 | 271 |
| **Q2TAC6** | KIF19 | Kinesin-like protein KIF19 | PTPPP | 726 | 730 |
| **Q2TAL5** | SMTNL2 | Smoothelin-like protein 2 | PGTPGTP | 94 | 100 |
| **Q2TAL6** | VWC2 UNQ739/PRO1434 | Brorin (Brain-specific chordin-like protein) (von Willebrand factor C domain-containing protein 2) | PTPEPP | 137 | 142 |
| **Q2TAZ0** | ATG2A KIAA0404 | Autophagy-related protein 2 homolog A | PTPDP | 851 | 855 |
| **Q2V2M9** | FHOD3 FHOS2 KIAA1695 | FH1/FH2 domain-containing protein 3 (Formactin-2) (Formin homolog overexpressed in spleen 2) (hFHOS2) | PGTPHHP | 546 | 552 |
| **Q2VYF4** | LETM2 | LETM1 domain-containing protein LETM2, mitochondrial (LETM1 and EF-hand domain-containing protein 2) (Leucine zipper-EF-hand-containing transmembrane protein 1-like) | PITPSTP | 451 | 457 |
| **Q30KQ2** | DEFB130 DEFB30; DEFB130L | Beta-defensin 130 (Beta-defensin 30) (DEFB-30) (Defensin, beta 130) | PTPVP | 70 | 74 |
| **Q33E94** | RFX4 | Transcription factor RFX4 (Regulatory factor X 4) (Testis development protein NYD-SP10) | PTPSP | 505 | 509 |
| **Q3B7T1** | EDRF1 C10orf137 | Erythroid differentiation-related factor 1 | PSTPIP | 630 | 635 |
| **Q3KQU3** | MAP7D1 KIAA1187 PARCC1 RPRC1 PP2464 | MAP7 domain-containing protein 1 (Arginine/proline-rich coiled-coil domain-containing protein 1) (Proline/arginine-rich coiled-coil domain-containing protein 1) | PTPAPP | 553 | 558 |
| **Q3L8U1** | CHD9 KIAA0308 KISH2 PRIC320 AD-013 x0008 | Chromodomain-helicase-DNA-binding protein 9 (CHD-9) (EC 3.6.4.12) (ATP-dependent helicase CHD9) (Chromatin-related mesenchymal modulator) (CReMM) (Chromatin-remodeling factor CHROM1) (Kismet homolog 2) (PPAR-alpha-interacting complex protein 320 kDa) (Peroxisomal proliferator-activated receptor A-interacting complex 320 kDa protein) | PLTPNP | 2111 | 2116 |
| **Q3SXR2** | C3orf36 | Uncharacterized protein C3orf36 | PSTPTGP | 97 | 103 |
| **Q3SXY8** | ARL13B ARL2L1 | ADP-ribosylation factor-like protein 13B (ADP-ribosylation factor-like protein 2-like 1) (ARL2-like protein 1) | PTPPPP | 372 | 377 |
| **Q3T8J9** | GON4L GON4 KIAA1606 | GON-4-like protein (GON-4 homolog) | PETPQFP | 1978 | 1984 |
| **Q3YEC7** | RABL6 C9orf86 PARF | Rab-like protein 6 (GTP-binding protein Parf) (Partner of ARF) (Rab-like protein 1) (RBEL1) | PGTPQP | 314 | 319 |
| **Q3ZLR7** | SUPT20HL1 FAM48B1 | Transcription factor SPT20 homolog-like 1 | PTPPAP | 818 | 823 |
| **Q494U1** | PLEKHN1 | Pleckstrin homology domain-containing family N member 1 (PH domain-containing family N member 1) | PTPSSP | 541 | 546 |
| **Q496M5** | PLK5 PLK5P FG060302 | Inactive serine/threonine-protein kinase PLK5 (Polo-like kinase 5) (PLK-5) | PTPVPP | 243 | 248 |
| **Q49AN0** | CRY2 KIAA0658 | Cryptochrome-2 | PTPELP | 584 | 589 |
| **Q4G0P3** | HYDIN HYDIN1 KIAA1864 | Hydrocephalus-inducing protein homolog | PATPEP | 1106 | 1111 |
| **Q4LE39** | ARID4B BRCAA1 RBBP1L1 RBP1L1 SAP180 | AT-rich interactive domain-containing protein 4B (ARID domain-containing protein 4B) (180 kDa Sin3-associated polypeptide) (Sin3-associated polypeptide p180) (Breast cancer-associated antigen BRCAA1) (Histone deacetylase complex subunit SAP180) (Retinoblastoma-binding protein 1-like 1) | PTTPESP | 1024 | 1030 |
| **Q4VC12** | MSS51 ZMYND17 | Putative protein MSS51 homolog, mitochondrial (Zinc finger MYND domain-containing protein 17) | PLTPSKP | 32 | 38 |
| **Q4VCS5** | AMOT KIAA1071 | Angiomotin | PSTPSP | 838 | 843 |
| **Q4VX76** | SYTL3 SLP3 | Synaptotagmin-like protein 3 (Exophilin-6) | PPTPPP | 155 | 160 |
| **Q4VXU2** | PABPC1L C20orf119 | Polyadenylate-binding protein 1-like | PVTPTQP | 425 | 431 |
| **Q4ZG55** | GREB1 KIAA0575 | Protein GREB1 (Gene regulated in breast cancer 1 protein) | PTPQP | 1196 | 1200 |
| **Q4ZHG4** | FNDC1 FNDC2 KIAA1866 MEL4B3 | Fibronectin type III domain-containing protein 1 (Activation-associated cDNA protein) (Expressed in synovial lining protein) | PTPTTP | 1503 | 1508 |
| **Q52MB2** | CCDC184 C12orf68 | Coiled-coil domain-containing protein 184 | PETPSP | 118 | 123 |
| **Q53EL9** | SEZ6 | Seizure protein 6 homolog (SEZ-6) (hSEZ-6) | PTPEQP | 46 | 51 |
| **Q53ET0** | CRTC2 TORC2 | CREB-regulated transcription coactivator 2 (Transducer of regulated cAMP response element-binding protein 2) (TORC-2) (Transducer of CREB protein 2) | PTPLDP | 288 | 293 |
| **Q53GL0** | PLEKHO1 CKIP1 OC120 HQ0024c | Pleckstrin homology domain-containing family O member 1 (PH domain-containing family O member 1) (C-Jun-binding protein) (JBP) (Casein kinase 2-interacting protein 1) (CK2-interacting protein 1) (CKIP-1) (Osteoclast maturation-associated gene 120 protein) | PPTPALP | 294 | 300 |
| **Q53H76** | PLA1A NMD PSPLA1 | Phospholipase A1 member A (EC 3.1.1.-) (Phosphatidylserine-specific phospholipase A1) (PS-PLA1) | PPTPQP | 28 | 33 |
| **Q53LP3** | SOWAHC ANKRD57 C2orf26 | Ankyrin repeat domain-containing protein SOWAHC (Ankyrin repeat domain-containing protein 57) (Protein sosondowah homolog C) | PRTPAP | 165 | 170 |
| **Q53SF7** | COBLL1 KIAA0977 | Cordon-bleu protein-like 1 | PTPIIP | 176 | 181 |
| **Q569H4** | PRR16 | Protein Largen (Mesenchymal stem cell protein DSC54) (Proline-rich protein 16) | PPTPHLP | 252 | 258 |
| **Q58A44** | C1QTNF9B-AS1 PCOTH | Prostate collagen triple helix protein (C1QTNF9B antisense RNA 1) (C1QTNF9B antisense gene protein 1) | PRTPGSP | 50 | 56 |
| **Q58DX5** | NAALADL2 | Inactive N-acetylated-alpha-linked acidic dipeptidase-like protein 2 (NAALADase L2) | PSTPGYP | 355 | 361 |
| **Q58EX2** | SDK2 KIAA1514 | Protein sidekick-2 | PTPQNP | 2135 | 2140 |
| **Q5BKX5** | C19orf54 | UPF0692 protein C19orf54 | PKTPVP | 16 | 21 |
| **Q5BKX8** | MURC | Muscle-related coiled-coil protein (Muscle-restricted coiled-coil protein) | PTPEP | 335 | 339 |
| **Q5CZC0** | FSIP2 | Fibrous sheath-interacting protein 2 | PTTPIKP | 595 | 601 |
| **Q5EBL2** | ZNF628 | Zinc finger protein 628 | PPTPPPP | 730 | 736 |
| **Q5FWE3** | PRRT3 UNQ5823/PRO19642 | Proline-rich transmembrane protein 3 | PTPAP | 944 | 948 |
| **Q5H9F3** | BCORL1 | BCL-6 corepressor-like protein 1 (BCoR-L1) (BCoR-like protein 1) | PTPTP | 358 | 362 |
| **Q5HY92** | FIGN | Fidgetin | PTPLPP | 282 | 287 |
| **Q5HYK7** | SH3D19 | SH3 domain-containing protein 19 (ADAM-binding protein Eve-1) (EEN-binding protein) (EBP) | PTPAP | 136 | 140 |
| **Q5IJ48** | CRB2 | Protein crumbs homolog 2 (Crumbs-like protein 2) | PGTPDP | 6 | 11 |
| **Q5JPB2** | ZNF831 C20orf174 | Zinc finger protein 831 | PTPGPP | 19 | 24 |
| **Q5JR12** | PPM1J PPP2CZ | Protein phosphatase 1J (EC 3.1.3.16) (Protein phosphatase 2C isoform zeta) (PP2C-zeta) | PTTPGTP | 199 | 205 |
| **Q5JS13** | RALGPS1 KIAA0351 RALGEF2 | Ras-specific guanine nucleotide-releasing factor RalGPS1 (Ral GEF with PH domain and SH3-binding motif 1) (Ral guanine nucleotide exchange factor 2) (RalGEF 2) (RalA exchange factor RalGPS1) | PTPPVP | 330 | 335 |
| **Q5JTC6** | AMER1 FAM123B WTX | APC membrane recruitment protein 1 (Amer1) (Protein FAM123B) (Wilms tumor gene on the X chromosome protein) | PTPEP | 241 | 245 |
| **Q5JTD0** | TJAP1 PILT TJP4 | Tight junction-associated protein 1 (Protein incorporated later into tight junctions) (Tight junction protein 4) | PTPQP | 283 | 287 |
| **Q5JU85** | IQSEC2 KIAA0522 | IQ motif and SEC7 domain-containing protein 2 | PHTPHSP | 1435 | 1441 |
| **Q5KSL6** | DGKK | Diacylglycerol kinase kappa (DAG kinase kappa) (EC 2.7.1.107) (142 kDa diacylglycerol kinase) (Diglyceride kinase kappa) (DGK-kappa) | PTPEP | 81 | 85 |
| **Q5KU26** | COLEC12 CLP1 NSR2 SCARA4 SRCL | Collectin-12 (Collectin placenta protein 1) (CL-P1) (hCL-P1) (Nurse cell scavenger receptor 2) (Scavenger receptor class A member 4) (Scavenger receptor with C-type lectin) | PTPAP | 598 | 602 |
| **Q5SQI0** | ATAT1 C6orf134 MEC17 Nbla00487 | Alpha-tubulin N-acetyltransferase 1 (Alpha-TAT) (Alpha-TAT1) (TAT) (EC 2.3.1.108) (Acetyltransferase mec-17 homolog) | PTPAAP | 214 | 219 |
| **Q5SV97** | PERM1 C1orf170 | PGC-1 and ERR-induced regulator in muscle protein 1 (PPARGC1 and ESRR-induced regulator in muscle 1) (Peroxisome proliferator-activated receptor gamma coactivator 1 and estrogen-related receptor-induced regulator in muscle 1) | PGTPLKP | 697 | 703 |
| **Q5SWA1** | PPP1R15B | Protein phosphatase 1 regulatory subunit 15B | PTPLGP | 37 | 42 |
| **Q5SWL7** | PRAMEF14 | PRAME family member 14 | PTPCP | 406 | 410 |
| **Q5SXM2** | SNAPC4 SNAP190 | snRNA-activating protein complex subunit 4 (SNAPc subunit 4) (Proximal sequence element-binding transcription factor subunit alpha) (PSE-binding factor subunit alpha) (PTF subunit alpha) (snRNA-activating protein complex 190 kDa subunit) (SNAPc 190 kDa subunit) | PTPLP | 1050 | 1054 |
| **Q5T0F9** | CC2D1B KIAA1836 | Coiled-coil and C2 domain-containing protein 1B (Five prime repressor element under dual repression-binding protein 2) (FRE under dual repression-binding protein 2) (Freud-2) | PATPVAP | 372 | 378 |
| **Q5T0Z8** | C6orf132 | Uncharacterized protein C6orf132 | PTPSVP | 95 | 100 |
| **Q5T124** | UBXN11 SOC UBXD5 PP2243 | UBX domain-containing protein 11 (Colorectal tumor-associated antigen COA-1) (Socius) (UBX domain-containing protein 5) | PNTPAPP | 389 | 395 |
| **Q5T1B0** | AXDND1 C1orf125 | Axonemal dynein light chain domain-containing protein 1 | PKTPSTP | 4 | 10 |
| **Q5T1N1** | AKNAD1 C1orf62 | Protein AKNAD1 | PATPSP | 763 | 768 |
| **Q5T1R4** | HIVEP3 KBP1 KIAA1555 KRC ZAS3 | Transcription factor HIVEP3 (Human immunodeficiency virus type I enhancer-binding protein 3) (Kappa-B and V(D)J recombination signal sequences-binding protein) (Kappa-binding protein 1) (KBP-1) (Zinc finger protein ZAS3) | PRTPKP | 775 | 780 |
| **Q5T200** | ZC3H13 KIAA0853 | Zinc finger CCCH domain-containing protein 13 | PRTPSPP | 261 | 267 |
| **Q5T2E6** | C10orf76 | UPF0668 protein C10orf76 | PTTPVTP | 334 | 340 |
| **Q5T481** | RBM20 | RNA-binding protein 20 (RNA-binding motif protein 20) | PQTPGQP | 204 | 210 |
| **Q5T5P2** | KIAA1217 SKT | Sickle tail protein homolog | PSTPVP | 315 | 320 |
| **Q5T5Y3** | CAMSAP1 | Calmodulin-regulated spectrin-associated protein 1 | PRTPTDP | 1142 | 1148 |
| **Q5T749** | KPRP C1orf45 | Keratinocyte proline-rich protein (hKPRP) | PTPRP | 442 | 446 |
| **Q5T751** | LCE1C LEP3 | Late cornified envelope protein 1C (Late envelope protein 3) | PTPKCP | 25 | 30 |
| **Q5T753** | LCE1E LEP5 | Late cornified envelope protein 1E (Late envelope protein 5) | PTPKCP | 25 | 30 |
| **Q5T754** | LCE1F LEP6 | Late cornified envelope protein 1F (Late envelope protein 6) | PTPKCP | 25 | 30 |
| **Q5T7N3** | KANK4 ANKRD38 | KN motif and ankyrin repeat domain-containing protein 4 (Ankyrin repeat domain-containing protein 38) | PTPSPP | 282 | 287 |
| **Q5T7P2** | LCE1A LEP1 | Late cornified envelope protein 1A (Late envelope protein 1) | PTPKCP | 25 | 30 |
| **Q5T8A7** | PPP1R26 KIAA0649 | Protein phosphatase 1 regulatory subunit 26 | PATPCRP | 321 | 327 |
| **Q5TAT6** | COL13A1 | Collagen alpha-1(XIII) chain (COLXIIIA1) | PGTPGP | 627 | 632 |
| **Q5TCY1** | TTBK1 BDTK KIAA1855 | Tau-tubulin kinase 1 (EC 2.7.11.1) (Brain-derived tau kinase) | PPTPGSP | 632 | 638 |
| **Q5TEA3** | C20orf194 | Uncharacterized protein C20orf194 | PTPPP | 1044 | 1048 |
| **Q5TGY3** | AHDC1 | AT-hook DNA-binding motif-containing protein 1 | PPTPRP | 32 | 37 |
| **Q5THJ4** | VPS13D KIAA0453 | Vacuolar protein sorting-associated protein 13D | PNTPPP | 776 | 781 |
| **Q5TIE3** | VWA5B1 | von Willebrand factor A domain-containing protein 5B1 | PATPAP | 802 | 807 |
| **Q5TZJ5** | SPATA31A1 C9orf36 FAM75A1 FAM75A2 SPATA31A2 | Spermatogenesis-associated protein 31A1 (Protein FAM75A1) | PHTPDP | 208 | 213 |
| **Q5VST9** | OBSCN KIAA1556 KIAA1639 | Obscurin (EC 2.7.11.1) (Obscurin-RhoGEF) (Obscurin-myosin light chain kinase) (Obscurin-MLCK) | PSTPRP | 6969 | 6974 |
| **Q5VT52** | RPRD2 KIAA0460 HSPC099 | Regulation of nuclear pre-mRNA domain-containing protein 2 | PGTPTSP | 480 | 486 |
| **Q5VTJ3** | KLHDC7A | Kelch domain-containing protein 7A | PTPDLP | 768 | 773 |
| **Q5VTT5** | MYOM3 | Myomesin-3 (Myomesin family member 3) | PVTPGP | 850 | 855 |
| **Q5VU36** | SPATA31A5 FAM75A5 | Spermatogenesis-associated protein 31A5 (Protein FAM75A5) | PHTPDP | 208 | 213 |
| **Q5VUE5** | C1orf53 | Uncharacterized protein C1orf53 | PSTPGRP | 54 | 60 |
| **Q5VVP1** | SPATA31A6 FAM75A6 | Spermatogenesis-associated protein 31A6 (Protein FAM75A6) | PHTPDP | 208 | 213 |
| **Q5VWG9** | TAF3 | Transcription initiation factor TFIID subunit 3 (140 kDa TATA box-binding protein-associated factor) (TBP-associated factor 3) (Transcription initiation factor TFIID 140 kDa subunit) (TAF(II)140) (TAF140) (TAFII-140) (TAFII140) | PQTPVRP | 331 | 337 |
| **Q5VWM6** | PRAMEF13 | Putative PRAME family member 13 | PTPCP | 454 | 458 |
| **Q5VYP0** | SPATA31A3 FAM75A3 | Spermatogenesis-associated protein 31A3 (Protein FAM75A3) | PHTPDP | 208 | 213 |
| **Q5VYV0** | FOXB2 | Forkhead box protein B2 | PTPALP | 368 | 373 |
| **Q5VZ46** | KIAA1614 | Uncharacterized protein KIAA1614 | PTPPP | 808 | 812 |
| **Q5VZF2** | MBNL2 MBLL MBLL39 MLP1 | Muscleblind-like protein 2 (Muscleblind-like protein 1) (Muscleblind-like protein-like) (Muscleblind-like protein-like 39) | PGTPLHP | 106 | 112 |
| **Q5VZP5** | DUSP27 | Inactive dual specificity phosphatase 27 | PTTPLP | 703 | 708 |
| **Q5W0A0** | ERICH6B FAM194B | Glutamate-rich protein 6B (Protein FAM194B) | PTPEKP | 434 | 439 |
| **Q5W0B1** | RNF219 C13orf7 | RING finger protein 219 | PITPENP | 58 | 64 |
| **Q5W0V3** | FAM160B1 KIAA1600 | Protein FAM160B1 | PATPDHP | 551 | 557 |
| **Q5XKR4** | OTP | Homeobox protein orthopedia | PTPGLP | 177 | 182 |
| **Q63HR2** | TNS2 KIAA1075 TENC1 | Tensin-2 (EC 3.1.3.-) (C1 domain-containing phosphatase and tensin homolog) (C1-TEN) (Tensin-like C1 domain-containing phosphatase) | PLTPVP | 1031 | 1036 |
| **Q63ZY3** | KANK2 ANKRD25 KIAA1518 MXRA3 SIP | KN motif and ankyrin repeat domain-containing protein 2 (Ankyrin repeat domain-containing protein 25) (Matrix-remodeling-associated protein 3) (SRC-1-interacting protein) (SIP) (SRC-interacting protein) (SRC1-interacting protein) | PGTPGP | 12 | 17 |
| **Q643R3** | LPCAT4 AGPAT7 AYTL3 LPEAT2 | Lysophospholipid acyltransferase LPCAT4 (1-acylglycerol-3-phosphate O-acyltransferase 7) (1-AGP acyltransferase 7) (1-AGPAT 7) (1-acylglycerophosphocholine O-acyltransferase) (EC 2.3.1.23) (1-acylglycerophosphoserine O-acyltransferase) (EC 2.3.1.n6) (1-alkenylglycerophosphoethanolamine O-acyltransferase) (EC 2.3.1.121) (1-alkylglycerophosphocholine O-acetyltransferase) (EC 2.3.1.67) (Acyltransferase-like 3) (Lysophosphatidylcholine acyltransferase 4) (Lysophosphatidylethanolamine acyltransferase 2) (EC 2.3.1.n7) (Plasmalogen synthase) | PTPGPP | 14 | 19 |
| **Q659C4** | LARP1B LARP2 | La-related protein 1B (La ribonucleoprotein domain family member 1B) (La ribonucleoprotein domain family member 2) (La-related protein 2) | PKTPRTP | 602 | 608 |
| **Q66K41** | ZNF385C | Zinc finger protein 385C | PPTPDP | 158 | 163 |
| **Q66K74** | MAP1S BPY2IP1 C19orf5 MAP8 VCY2IP1 | Microtubule-associated protein 1S (MAP-1S) (BPY2-interacting protein 1) (Microtubule-associated protein 8) (Variable charge Y chromosome 2-interacting protein 1) (VCY2-interacting protein 1) (VCY2IP-1) [Cleaved into: MAP1S heavy chain; MAP1S light chain] | PRTPSP | 636 | 641 |
| **Q674R7** | ATG9B APG9L2 NOS3AS | Autophagy-related protein 9B (APG9-like 2) (Nitric oxide synthase 3-overlapping antisense gene protein) (Protein sONE) | PATPTP | 124 | 129 |
| **Q68CZ2** | TNS3 TEM6 TENS1 TPP | Tensin-3 (Tensin-like SH2 domain-containing protein 1) (Tumor endothelial marker 6) | PTPSIP | 1049 | 1054 |
| **Q68D20** | PMS2CL PMS2P13 | Protein PMS2CL (PMS2-C terminal-like protein) | PKTPEP | 51 | 56 |
| **Q68DV7** | RNF43 | E3 ubiquitin-protein ligase RNF43 (EC 6.3.2.-) (RING finger protein 43) | PETPGP | 712 | 717 |
| **Q68EM7** | ARHGAP17 RICH1 MSTP066 MSTP110 | Rho GTPase-activating protein 17 (Rho-type GTPase-activating protein 17) (RhoGAP interacting with CIP4 homologs protein 1) (RICH-1) | PQTPTPP | 755 | 761 |
| **Q69YN4** | KIAA1429 MSTP054 | Protein virilizer homolog | PRTPPGP | 182 | 188 |
| **Q69YQ0** | SPECC1L CYTSA KIAA0376 | Cytospin-A (Renal carcinoma antigen NY-REN-22) (Sperm antigen with calponin homology and coiled-coil domains 1-like) (SPECC1-like protein) | PRTPLSP | 863 | 869 |
| **Q6AI08** | HEATR6 ABC1 | HEAT repeat-containing protein 6 (Amplified in breast cancer protein 1) | PTPLP | 287 | 291 |
| **Q6DKI7** | PVRIG C7orf15 | Transmembrane protein PVRIG (Poliovirus receptor-related immunoglobulin domain-containing protein) | PPTPAP | 161 | 166 |
| **Q6DN03** | HIST2H2BC | Putative histone H2B type 2-C (Histone H2B.t) (H2B/t) | PSTPCP | 108 | 113 |
| **Q6DRA6** | HIST2H2BD | Putative histone H2B type 2-D | PSTPCP | 108 | 113 |
| **Q6EMK4** | VASN SLITL2 UNQ314/PRO357/PRO1282 | Vasorin (Protein slit-like 2) | PTPVTP | 453 | 458 |
| **Q6ICB4** | FAM109B | Sesquipedalian-2 (Ses2) (27 kDa inositol polyphosphate phosphatase interacting protein B) (IPIP27B) | PGTPPTP | 30 | 36 |
| **Q6IE81** | JADE1 KIAA1807 PHF17 | Protein Jade-1 (Jade family PHD finger protein 1) (PHD finger protein 17) | PTTPASP | 738 | 744 |
| **Q6IQ16** | SPOPL | Speckle-type POZ protein-like (HIB homolog 2) (Roadkill homolog 2) | PTPPLP | 5 | 10 |
| **Q6J272** | FAM166A HSD46 | Protein FAM166A | PDTPHPP | 132 | 138 |
| **Q6JBY9** | RCSD1 CAPZIP | CapZ-interacting protein (Protein kinase substrate CapZIP) (RCSD domain-containing protein 1) | PSTPSSP | 122 | 128 |
| **Q6JVE5** | LCN12 | Epididymal-specific lipocalin-12 | PTPLP | 22 | 26 |
| **Q6MZP7** | LIN54 CXCDC1 KIAA2037 | Protein lin-54 homolog (CXC domain-containing protein 1) | PATPLP | 439 | 444 |
| **Q6NSI3** | FAM53A | Protein FAM53A (Dorsal neural-tube nuclear protein) | PATPRP | 182 | 187 |
| **Q6NSI8** | KIAA1841 | Uncharacterized protein KIAA1841 | PNTPWGP | 524 | 530 |
| **Q6NSJ2** | PHLDB3 | Pleckstrin homology-like domain family B member 3 | PPTPPHP | 497 | 503 |
| **Q6NT89** | TRNP1 C1orf225 TRNP | TMF-regulated nuclear protein 1 | PTPTP | 44 | 48 |
| **Q6NUN9** | ZNF746 PARIS | Zinc finger protein 746 (Parkin-interacting substrate) (PARIS) | PTPPAP | 602 | 607 |
| **Q6NUP7** | PPP4R4 KIAA1622 PP4R4 | Serine/threonine-protein phosphatase 4 regulatory subunit 4 | PNTPLP | 834 | 839 |
| **Q6NWY9** | PRPF40B HYPC | Pre-mRNA-processing factor 40 homolog B (Huntingtin yeast partner C) (Huntingtin-interacting protein C) | PTPVP | 208 | 212 |
| **Q6NYC8** | PPP1R18 HKMT1098 KIAA1949 | Phostensin (Protein phosphatase 1 F-actin cytoskeleton-targeting subunit) (Protein phosphatase 1 regulatory subunit 18) | PATPATP | 482 | 488 |
| **Q6NZY4** | ZCCHC8 | Zinc finger CCHC domain-containing protein 8 (TRAMP-like complex RNA-binding factor ZCCHC8) | PDTPPLP | 470 | 476 |
| **Q6P047** | C8orf74 | Uncharacterized protein C8orf74 | PTPIPP | 262 | 267 |
| **Q6P3S6** | FBXO42 FBX42 JFK KIAA1332 | F-box only protein 42 (Just one F-box and Kelch domain-containing protein) | PSTPSAP | 465 | 471 |
| **Q6P4R8** | NFRKB INO80G | Nuclear factor related to kappa-B-binding protein (DNA-binding protein R kappa-B) (INO80 complex subunit G) | PPTPVTP | 716 | 722 |
| **Q6P9F7** | LRRC8B KIAA0231 UNQ6413/PRO21207 | Volume-regulated anion channel subunit LRRC8B (Leucine-rich repeat-containing protein 8B) (T-cell activation leucine repeat-rich protein) (TA-LRRP) | PGTPLP | 83 | 88 |
| **Q6PCE3** | PGM2L1 BM32A | Glucose 1,6-bisphosphate synthase (EC 2.7.1.106) (PMMLP) (Phosphoglucomutase-2-like 1) | PTPFVP | 153 | 158 |
| **Q6PII3** | CCDC174 C3orf19 | Coiled-coil domain-containing protein 174 | PTPRP | 333 | 337 |
| **Q6PJ61** | FBXO46 FBX46 FBXO34L | F-box only protein 46 (F-box only protein 34-like) | PTTPAP | 193 | 198 |
| **Q6PJG2** | ELMSAN1 C14orf117 C14orf43 | ELM2 and SANT domain-containing protein 1 (MIDEAS) | PYTPPP | 653 | 658 |
| **Q6PKG0** | LARP1 KIAA0731 LARP | La-related protein 1 (La ribonucleoprotein domain family member 1) | PRTPRTP | 783 | 789 |
| **Q6PRD1** | GPR179 GPR158L GPR158L1 | Probable G-protein coupled receptor 179 (Probable G-protein coupled receptor 158-like 1) (GPR158-like) | PTPAP | 970 | 974 |
| **Q6Q4G3** | LVRN AQPEP | Aminopeptidase Q (AP-Q) (APQ) (EC 3.4.11.-) (CHL2 antigen) (Laeverin) | PTPTP | 66 | 70 |
| **Q6Q6R5** | CRIP3 CRP3 | Cysteine-rich protein 3 (CRP-3) (Chromosome 6 LIM domain only protein) (h6LIMo) | PPTPSP | 79 | 84 |
| **Q6QEF8** | CORO6 PP1009 PP1782 PP1881 | Coronin-6 (Coronin-like protein E) (Clipin-E) | PDTPGP | 364 | 369 |
| **Q6SJ96** | TBPL2 TBP2 TRF3 | TATA box-binding protein-like protein 2 (TBP-like protein 2) (TATA box-binding protein-related factor 3) (TBP-related factor 3) | PMTPMTP | 185 | 191 |
| **Q6SPF0** | SAMD1 | Atherin (Sterile alpha motif domain-containing protein 1) (SAM domain-containing protein 1) | PPTPAPP | 128 | 134 |
| **Q6UB99** | ANKRD11 ANCO1 | Ankyrin repeat domain-containing protein 11 (Ankyrin repeat-containing cofactor 1) | PTPAP | 2361 | 2365 |
| **Q6UUV9** | CRTC1 KIAA0616 MECT1 TORC1 WAMTP1 | CREB-regulated transcription coactivator 1 (Mucoepidermoid carcinoma translocated protein 1) (Transducer of regulated cAMP response element-binding protein 1) (TORC-1) (Transducer of CREB protein 1) | PTPLDP | 259 | 264 |
| **Q6UW60** | PCSK4 PC4 UNQ2757/PRO6496 | Proprotein convertase subtilisin/kexin type 4 (EC 3.4.21.-) (Proprotein convertase 4) (PC4) | PTPILP | 463 | 468 |
| **Q6UWB4** | PRSS55 TSP1 UNQ9391/PRO34284 | Serine protease 55 (EC 3.4.21.-) (Testis serine protease 1) (T-SP1) | PRTPLP | 20 | 25 |
| **Q6UWD8** | C16orf54 UNQ9389/PRO34280 | Transmembrane protein C16orf54 | PLTPEPP | 2 | 8 |
| **Q6UWL6** | KIRREL2 NEPH3 UNQ5827/PRO19646 | Kin of IRRE-like protein 2 (Kin of irregular chiasm-like protein 2) (Nephrin-like protein 3) | PGTPPFP | 686 | 692 |
| **Q6UXI9** | NPNT EGFL6L POEM UNQ295/PRO334 | Nephronectin (Preosteoblast EGF-like repeat protein with MAM domain) (Protein EGFL6-like) | PKTPYIP | 297 | 303 |
| **Q6UXK2** | ISLR2 KIAA1465 LINX UNQ1885/PRO4329 | Immunoglobulin superfamily containing leucine-rich repeat protein 2 (Leucine-rich repeat domain and immunoglobulin domain-containing axon extension protein) | PTPAP | 317 | 321 |
| **Q6UXU6** | TMEM92 UNQ5801/PRO19608 | Transmembrane protein 92 | PTPTEP | 131 | 136 |
| **Q6UXX5** | ITIH6 ITIH5L UNQ6369/PRO21074 | Inter-alpha-trypsin inhibitor heavy chain H6 (Inter-alpha-trypsin inhibitor heavy chain H5-like protein) (Inter-alpha inhibitor H5-like protein) | PETPNP | 874 | 879 |
| **Q6UXY1** | BAIAP2L2 UNQ9336/PRO34007 | Brain-specific angiogenesis inhibitor 1-associated protein 2-like protein 2 (BAI1-associated protein 2-like protein 2) (Planar intestinal- and kidney-specific BAR domain protein) (Pinkbar) | PMTPVTP | 392 | 398 |
| **Q6UY14** | ADAMTSL4 TSRC1 PP1396 UNQ2803/PRO34012 | ADAMTS-like protein 4 (ADAMTSL-4) (Thrombospondin repeat-containing protein 1) | PTPEPP | 609 | 614 |
| **Q6V0I7** | FAT4 CDHF14 FATJ Nbla00548 | Protocadherin Fat 4 (hFat4) (Cadherin family member 14) (FAT tumor suppressor homolog 4) (Fat-like cadherin protein FAT-J) | PTPNP | 4693 | 4697 |
| **Q6VAB6** | KSR2 | Kinase suppressor of Ras 2 (hKSR2) (EC 2.7.11.1) | PTPGAP | 214 | 219 |
| **Q6W4X9** | MUC6 | Mucin-6 (MUC-6) (Gastric mucin-6) | PTTPQPP | 1199 | 1205 |
| **Q6WCQ1** | MPRIP KIAA0864 MRIP RHOIP3 | Myosin phosphatase Rho-interacting protein (M-RIP) (Rho-interacting protein 3) (RIP3) (p116Rip) | PPTPQEP | 164 | 170 |
| **Q6WN34** | CHRDL2 BNF1 CHL2 UNQ765/PRO1557 | Chordin-like protein 2 (Breast tumor novel factor 1) (BNF-1) (Chordin-related protein 2) | PGTPAP | 211 | 216 |
| **Q6Y288** | B3GLCT B3GALTL B3GTL | Beta-1,3-glucosyltransferase (Beta3Glc-T) (EC 2.4.1.-) (Beta 3-glucosyltransferase) (Beta-3-glycosyltransferase-like) | PLTPVP | 233 | 238 |
| **Q6ZMN7** | PDZRN4 LNX4 SEMCAP3L | PDZ domain-containing RING finger protein 4 (Ligand of Numb protein X 4) (SEMACAP3-like protein) | PPTPPVP | 351 | 357 |
| **Q6ZMQ8** | AATK AATYK KIAA0641 LMR1 LMTK1 | Serine/threonine-protein kinase LMTK1 (EC 2.7.11.1) (Apoptosis-associated tyrosine kinase) (AATYK) (Brain apoptosis-associated tyrosine kinase) (CDK5-binding protein) (Lemur tyrosine kinase 1) (p35-binding protein) (p35BP) | PGTPRAP | 1136 | 1142 |
| **Q6ZMS7** | ZNF783 | Protein ZNF783 | PSTPLP | 21 | 26 |
| **Q6ZMT1** | STAC2 | SH3 and cysteine-rich domain-containing protein 2 (24b2/STAC2) (Src homology 3 and cysteine-rich domain-containing protein 2) | PPTPLPP | 70 | 76 |
| **Q6ZN01** | MAMSTR MASTR | MEF2-activating motif and SAP domain-containing transcriptional regulator (MEF2-activating SAP transcriptional regulatory protein) | PLTPCPP | 147 | 153 |
| **Q6ZN30** | BNC2 | Zinc finger protein basonuclin-2 | PTPPP | 6 | 10 |
| **Q6ZN55** | ZNF574 | Zinc finger protein 574 | PTTPVPP | 421 | 427 |
| **Q6ZNC4** | ZNF704 | Zinc finger protein 704 | PSTPSPP | 142 | 148 |
| **Q6ZNJ1** | NBEAL2 KIAA0540 UNQ253/PRO290 | Neurobeachin-like protein 2 | PTPAP | 618 | 622 |
| **Q6ZNR8** | LINC00176 NCRNA00176 PRR17 | Putative uncharacterized protein encoded by LINC00176 (Putative proline-rich protein 17) | PPTPTPP | 18 | 24 |
| **Q6ZRI6** | C15orf39 FP6578 | Uncharacterized protein C15orf39 | PLTPRCP | 362 | 368 |
| **Q6ZRS2** | SRCAP KIAA0309 | Helicase SRCAP (EC 3.6.4.-) (Domino homolog 2) (Snf2-related CBP activator) | PPTPGP | 570 | 575 |
| **Q6ZS17** | FAM65A KIAA1930 | Protein FAM65A | PRTPHP | 726 | 731 |
| **Q6ZS30** | NBEAL1 ALS2CR16 ALS2CR17 | Neurobeachin-like protein 1 (Amyotrophic lateral sclerosis 2 chromosomal region candidate gene 16 protein) (Amyotrophic lateral sclerosis 2 chromosomal region candidate gene 17 protein) | PSTPSP | 1395 | 1400 |
| **Q6ZS94** | C1orf229 | Putative uncharacterized protein C1orf229 | PTPSP | 133 | 137 |
| **Q6ZSY5** | PPP1R3F | Protein phosphatase 1 regulatory subunit 3F (R3F) | PTPLRP | 369 | 374 |
| **Q6ZSZ5** | ARHGEF18 KIAA0521 | Rho guanine nucleotide exchange factor 18 (114 kDa Rho-specific guanine nucleotide exchange factor) (p114-Rho-GEF) (p114RhoGEF) (Septin-associated RhoGEF) (SA-RhoGEF) | PTPSP | 169 | 173 |
| **Q6ZSZ6** | TSHZ1 SDCCAG33 TSH1 | Teashirt homolog 1 (Antigen NY-CO-33) (Serologically defined colon cancer antigen 33) | PTPTPP | 156 | 161 |
| **Q6ZTN6** | ANKRD13D | Ankyrin repeat domain-containing protein 13D | PRTPPAP | 467 | 473 |
| **Q6ZU64** | CCDC108 | Coiled-coil domain-containing protein 108 | PTPQP | 1816 | 1820 |
| **Q6ZU65** | UBN2 KIAA2030 | Ubinuclein-2 | PTPKP | 1081 | 1085 |
| **Q6ZU67** | BEND4 CCDC4 | BEN domain-containing protein 4 (Coiled-coil domain-containing protein 4) | PTPNP | 263 | 267 |
| **Q6ZV73** | FGD6 KIAA1362 ZFYVE24 | FYVE, RhoGEF and PH domain-containing protein 6 (Zinc finger FYVE domain-containing protein 24) | PYTPKFP | 307 | 313 |
| **Q6ZVF9** | GPRIN3 KIAA2027 | G protein-regulated inducer of neurite outgrowth 3 (GRIN3) | PTPSP | 580 | 584 |
| **Q70CQ4** | USP31 KIAA1203 | Ubiquitin carboxyl-terminal hydrolase 31 (EC 3.4.19.12) (Deubiquitinating enzyme 31) (Ubiquitin thioesterase 31) (Ubiquitin-specific-processing protease 31) | PTPPP | 102 | 106 |
| **Q70E73** | RAPH1 ALS2CR18 ALS2CR9 KIAA1681 LPD PREL2 RMO1 | Ras-associated and pleckstrin homology domains-containing protein 1 (RAPH1) (Amyotrophic lateral sclerosis 2 chromosomal region candidate gene 18 protein) (Amyotrophic lateral sclerosis 2 chromosomal region candidate gene 9 protein) (Lamellipodin) (Proline-rich EVH1 ligand 2) (PREL-2) (Protein RMO1) | PPTPPPP | 760 | 766 |
| **Q71F56** | MED13L KIAA1025 PROSIT240 THRAP2 TRAP240L | Mediator of RNA polymerase II transcription subunit 13-like (Mediator complex subunit 13-like) (Thyroid hormone receptor-associated protein 2) (Thyroid hormone receptor-associated protein complex 240 kDa component-like) | PTPRTP | 1049 | 1054 |
| **Q75N03** | CBLL1 HAKAI RNF188 | E3 ubiquitin-protein ligase Hakai (EC 6.3.2.-) (Casitas B-lineage lymphoma-transforming sequence-like protein 1) (RING finger protein 188) (c-Cbl-like protein 1) | PITPPP | 383 | 388 |
| **Q75N90** | FBN3 KIAA1776 | Fibrillin-3 [Cleaved into: Fibrillin-3 C-terminal peptide] | PTPISP | 1692 | 1697 |
| **Q75VX8** | GAREM2 FAM59B GAREML KIAA2038 HRIHFB2063 | GRB2-associated and regulator of MAPK protein 2 (GRB2-associated and regulator of MAPK1-like) | PTPLSP | 754 | 759 |
| **Q765P7** | MTSS1L | MTSS1-like protein (Actin-bundling with BAIAP2 homology protein 1) (ABBA-1) | PTPPP | 708 | 712 |
| **Q76KP1** | B4GALNT4 | N-acetyl-beta-glucosaminyl-glycoprotein 4-beta-N-acetylgalactosaminyltransferase 1 (NGalNAc-T1) (EC 2.4.1.244) (Beta-1,4-N-acetylgalactosaminyltransferase IV) (Beta4GalNAc-T4) (Beta4GalNAcT4) | PPTPPRP | 476 | 482 |
| **Q76M96** | CCDC80 DRO1 URB HBE245 | Coiled-coil domain-containing protein 80 (Down-regulated by oncogenes protein 1) (Up-regulated in BRS-3 deficient mouse homolog) | PWTPSP | 387 | 392 |
| **Q76NI1** | KNDC1 C10orf23 KIAA1768 RASGEF2 VKIND hucep-9 | Protein very KIND (Cerebral protein 9) (KIND domain-containing protein 1) (Kinase non-catalytic C-lobe domain-containing protein 1) (Ras-GEF domain-containing family member 2) | PTPEGP | 241 | 246 |
| **Q7L3V2** | BOP C22orf29 | Protein Bop (BH3-only protein) | PTPVP | 297 | 301 |
| **Q7L4S7** | ARMCX6 | Protein ARMCX6 | PTPDP | 168 | 172 |
| **Q7L8A9** | VASH1 KIAA1036 VASH | Vasohibin-1 | PSTPVP | 114 | 119 |
| **Q7LBE3** | SLC26A9 | Solute carrier family 26 member 9 (Anion transporter/exchanger protein 9) | PTPVSP | 320 | 325 |
| **Q7RTP6** | MICAL3 KIAA0819 KIAA1364 | Protein-methionine sulfoxide oxidase MICAL3 (EC 1.14.13.-) (Molecule interacting with CasL protein 3) (MICAL-3) | PTPRSP | 1402 | 1407 |
| **Q7RTU5** | ASCL5 BHLHA47 | Achaete-scute homolog 5 (ASH-5) (hASH5) (Class A basic helix-loop-helix protein 47) (bHLHa47) | PATPRP | 236 | 241 |
| **Q7RTU9** | STRC | Stereocilin | PPTPTRP | 153 | 159 |
| **Q7RTY7** | OVCH1 | Ovochymase-1 (EC 3.4.21.-) | PPTPSP | 832 | 837 |
| **Q7Z2Z1** | TICRR C15orf42 | Treslin (TopBP1-interacting checkpoint and replication regulator) (TopBP1-interacting, replication-stimulating protein) | PWTPSP | 1666 | 1671 |
| **Q7Z3B3** | KANSL1 CENP-36 KIAA1267 MSL1V1 NSL1 | KAT8 regulatory NSL complex subunit 1 (MLL1/MLL complex subunit KANSL1) (MSL1 homolog 1) (hMSL1v1) (NSL complex protein NSL1) (Non-specific lethal 1 homolog) | PSTPQP | 964 | 969 |
| **Q7Z3E5** | ARMC9 KIAA1868 | LisH domain-containing protein ARMC9 (Melanoma/melanocyte-specific tumor antigen KU-MEL-1) (NS21) | PTTPRQP | 733 | 739 |
| **Q7Z3K3** | POGZ KIAA0461 SUHW5 ZNF280E ZNF635 Nbla00003 | Pogo transposable element with ZNF domain (Suppressor of hairy wing homolog 5) (Zinc finger protein 280E) (Zinc finger protein 635) | PPTPTHP | 918 | 924 |
| **Q7Z449** | CYP2U1 | Cytochrome P450 2U1 (EC 1.14.14.1) | PTPWP | 63 | 67 |
| **Q7Z570** | ZNF804A C2orf10 | Zinc finger protein 804A | PSTPLQP | 1077 | 1083 |
| **Q7Z572** | SPATA21 | Spermatogenesis-associated protein 21 | PSTPGP | 21 | 26 |
| **Q7Z5J4** | RAI1 KIAA1820 | Retinoic acid-induced protein 1 | PRTPGPP | 1066 | 1072 |
| **Q7Z5L9** | IRF2BP2 | Interferon regulatory factor 2-binding protein 2 (IRF-2-binding protein 2) (IRF-2BP2) | PPTPQPP | 145 | 151 |
| **Q7Z5P9** | MUC19 | Mucin-19 (MUC-19) | PGTPGTP | 2001 | 2007 |
| **Q7Z695** | ADCK2 AARF | Uncharacterized aarF domain-containing protein kinase 2 (EC 2.7.11.-) | PTPLRP | 386 | 391 |
| **Q7Z6J2** | GRASP | General receptor for phosphoinositides 1-associated scaffold protein (GRP1-associated scaffold protein) | PTPGPP | 37 | 42 |
| **Q7Z6L0** | PRRT2 | Proline-rich transmembrane protein 2 (Dispanin subfamily B member 3) (DSPB3) | PTPKP | 150 | 154 |
| **Q7Z6Z7** | HUWE1 KIAA0312 KIAA1578 UREB1 HSPC272 | E3 ubiquitin-protein ligase HUWE1 (EC 6.3.2.-) (ARF-binding protein 1) (ARF-BP1) (HECT, UBA and WWE domain-containing protein 1) (Homologous to E6AP carboxyl terminus homologous protein 9) (HectH9) (Large structure of UREB1) (LASU1) (Mcl-1 ubiquitin ligase E3) (Mule) (Upstream regulatory element-binding protein 1) (URE-B1) (URE-binding protein 1) | PYTPTP | 1119 | 1124 |
| **Q7Z736** | PLEKHH3 | Pleckstrin homology domain-containing family H member 3 (PH domain-containing family H member 3) | PTPRPP | 576 | 581 |
| **Q7Z7A3** | CTU1 ATPBD3 NCS6 | Cytoplasmic tRNA 2-thiolation protein 1 (EC 2.7.7.-) (ATP-binding domain-containing protein 3) (Cancer-associated gene protein) (Cytoplasmic tRNA adenylyltransferase 1) | PGTPGDP | 330 | 336 |
| **Q7Z7G0** | ABI3BP NESHBP TARSH | Target of Nesh-SH3 (Tarsh) (ABI gene family member 3-binding protein) (Nesh-binding protein) (NeshBP) | PTTPAP | 455 | 460 |
| **Q7Z7K2** | ZNF467 | Zinc finger protein 467 | PTPSFP | 345 | 350 |
| **Q7Z7K6** | CENPV PRR6 | Centromere protein V (CENP-V) (Nuclear protein p30) (Proline-rich protein 6) | PPTPATP | 96 | 102 |
| **Q7Z7M0** | MEGF8 C19orf49 EGFL4 KIAA0817 | Multiple epidermal growth factor-like domains protein 8 (Multiple EGF-like domains protein 8) (Epidermal growth factor-like protein 4) (EGF-like protein 4) | PPTPAP | 1147 | 1152 |
| **Q7Z7M1** | ADGRD2 GPR144 PGR24 | Adhesion G-protein coupled receptor D2 (G-protein coupled receptor 144) (G-protein coupled receptor PGR24) | PSTPRHP | 952 | 958 |
| **Q86SS6** | SYT9 | Synaptotagmin-9 (Synaptotagmin IX) (SytIX) | PPTPCP | 118 | 123 |
| **Q86TC9** | MYPN MYOP | Myopalladin (145 kDa sarcomeric protein) | PTPPP | 792 | 796 |
| **Q86U70** | LDB1 CLIM2 | LIM domain-binding protein 1 (LDB-1) (Carboxyl-terminal LIM domain-binding protein 2) (CLIM-2) (LIM domain-binding factor CLIM2) (hLdb1) (Nuclear LIM interactor) | PTPMYP | 44 | 49 |
| **Q86UC2** | RSPH3 RSHL2 RSP3 | Radial spoke head protein 3 homolog (A-kinase anchor protein RSPH3) (Radial spoke head-like protein 2) | PQTPEP | 241 | 246 |
| **Q86UL8** | MAGI2 ACVRINP1 AIP1 KIAA0705 | Membrane-associated guanylate kinase, WW and PDZ domain-containing protein 2 (Atrophin-1-interacting protein 1) (AIP-1) (Atrophin-1-interacting protein A) (Membrane-associated guanylate kinase inverted 2) (MAGI-2) | PATPNSP | 1044 | 1050 |
| **Q86UP3** | ZFHX4 | Zinc finger homeobox protein 4 (Zinc finger homeodomain protein 4) (ZFH-4) | PSTPGTP | 595 | 601 |
| **Q86US8** | SMG6 C17orf31 EST1A KIAA0732 | Telomerase-binding protein EST1A (EC 3.1.-.-) (EST1-like protein A) (Ever shorter telomeres 1A) (Smg-6 homolog) (Telomerase subunit EST1A) (hSmg5/7a) | PRTPGP | 511 | 516 |
| **Q86UU0** | BCL9L DLNB11 | B-cell CLL/lymphoma 9-like protein (B-cell lymphoma 9-like protein) (BCL9-like protein) (Protein BCL9-2) | PPTPEP | 283 | 288 |
| **Q86UU5** | GGN | Gametogenetin | PPTPPP | 442 | 447 |
| **Q86V15** | CASZ1 CST SRG ZNF693 | Zinc finger protein castor homolog 1 (Castor-related protein) (Putative survival-related protein) (Zinc finger protein 693) | PTPTP | 796 | 800 |
| **Q86V87** | FAM160B2 RAI16 FP13191 | Protein FAM160B2 (Retinoic acid-induced protein 16) | PLTPTP | 583 | 588 |
| **Q86VH4** | LRRTM4 UNQ3075/PRO9907 | Leucine-rich repeat transmembrane neuronal protein 4 | PQTPQKP | 375 | 381 |
| **Q86VM9** | ZC3H18 NHN1 | Zinc finger CCCH domain-containing protein 18 (Nuclear protein NHN1) | PTPSP | 610 | 614 |
| **Q86VR2** | FAM134C | Protein FAM134C | PTTPGP | 8 | 13 |
| **Q86VW2** | ARHGEF25 GEFT | Rho guanine nucleotide exchange factor 25 (Guanine nucleotide exchange factor GEFT) (Rac/Cdc42/Rho exchange factor GEFT) (RhoA/Rac/Cdc42 guanine nucleotide exchange factor GEFT) (p63RhoGEF) | PTPKTP | 561 | 566 |
| **Q86W28** | NLRP8 NALP8 NOD16 PAN4 | NACHT, LRR and PYD domains-containing protein 8 (Nucleotide-binding oligomerization domain protein 16) (PYRIN and NACHT-containing protein 4) | PTPHPP | 1029 | 1034 |
| **Q86WA8** | LONP2 LONP | Lon protease homolog 2, peroxisomal (EC 3.4.21.-) (Lon protease-like protein 2) (Lon protease 2) (Peroxisomal Lon protease) | PNTPDP | 61 | 66 |
| **Q86WK7** | AMIGO3 ALI3 KIAA1851 UNQ6084/PRO20089 | Amphoterin-induced protein 3 (AMIGO-3) (Alivin-3) | PQTPSP | 420 | 425 |
| **Q86WR7** | PROSER2 C10orf47 | Proline and serine-rich protein 2 | PSTPDPP | 165 | 171 |
| **Q86X27** | RALGPS2 | Ras-specific guanine nucleotide-releasing factor RalGPS2 (Ral GEF with PH domain and SH3-binding motif 2) (RalA exchange factor RalGPS2) | PQTPPSP | 324 | 330 |
| **Q86X51** | CXorf67 | Uncharacterized protein CXorf67 | PATPPEP | 497 | 503 |
| **Q86X59** | C17orf82 | Putative uncharacterized protein C17orf82 | PTPEAP | 138 | 143 |
| **Q86XN7** | PROSER1 C13orf23 KIAA2032 | Proline and serine-rich protein 1 | PYTPNP | 235 | 240 |
| **Q86XX4** | FRAS1 KIAA1500 | Extracellular matrix protein FRAS1 | PQTPEAP | 1458 | 1464 |
| **Q86Y91** | KIF18B | Kinesin-like protein KIF18B | PCTPELP | 436 | 442 |
| **Q86Y97** | KMT5C SUV420H2 PP7130 | Histone-lysine N-methyltransferase KMT5C (EC 2.1.1.43) (Lysine N-methyltransferase 5C) (Lysine-specific methyltransferase 5C) (Suppressor of variegation 4-20 homolog 2) (Su(var)4-20 homolog 2) (Suv4-20h2) | PATPAP | 414 | 419 |
| **Q86YC2** | PALB2 FANCN | Partner and localizer of BRCA2 | PGTPPP | 807 | 812 |
| **Q86YN6** | PPARGC1B PERC PGC1 PGC1B PPARGC1 | Peroxisome proliferator-activated receptor gamma coactivator 1-beta (PGC-1-beta) (PPAR-gamma coactivator 1-beta) (PPARGC-1-beta) (PGC-1-related estrogen receptor alpha coactivator) | PQTPEP | 306 | 311 |
| **Q86YV0** | RASAL3 | RAS protein activator like-3 | PPTPQIP | 119 | 125 |
| **Q86YV9** | HPS6 | Hermansky-Pudlak syndrome 6 protein (Ruby-eye protein homolog) (Ru) | PPTPFP | 718 | 723 |
| **Q86Z02** | HIPK1 KIAA0630 MYAK NBAK2 | Homeodomain-interacting protein kinase 1 (EC 2.7.11.1) (Nuclear body-associated kinase 2) | PDTPSPP | 896 | 902 |
| **Q8IUC6** | TICAM1 PRVTIRB TRIF | TIR domain-containing adapter molecule 1 (TICAM-1) (Proline-rich, vinculin and TIR domain-containing protein B) (Putative NF-kappa-B-activating protein 502H) (Toll-interleukin-1 receptor domain-containing adapter protein inducing interferon beta) (MyD88-3) (TIR domain-containing adapter protein inducing IFN-beta) | PDTPAAP | 288 | 294 |
| **Q8IUM7** | NPAS4 BHLHE79 NXF PASD10 | Neuronal PAS domain-containing protein 4 (Neuronal PAS4) (Class E basic helix-loop-helix protein 79) (bHLHe79) (HLH-PAS transcription factor NXF) (PAS domain-containing protein 10) | PYTPHQP | 425 | 431 |
| **Q8IUW5** | RELL1 PSEC0162 | RELT-like protein 1 | PVTPSTP | 149 | 155 |
| **Q8IV13** | CCNJL | Cyclin-J-like protein | PGTPPTP | 317 | 323 |
| **Q8IV31** | TMEM139 UNQ1932/PRO4407 | Transmembrane protein 139 | PLTPPP | 188 | 193 |
| **Q8IV56** | PRR15 | Proline-rich protein 15 | PTPPAP | 42 | 47 |
| **Q8IV61** | RASGRP3 GRP3 KIAA0846 | Ras guanyl-releasing protein 3 (Calcium and DAG-regulated guanine nucleotide exchange factor III) (Guanine nucleotide exchange factor for Rap1) | PTTPNKP | 392 | 398 |
| **Q8IVF2** | AHNAK2 C14orf78 KIAA2019 | Protein AHNAK2 | PTPEDP | 5153 | 5158 |
| **Q8IVH2** | FOXP4 FKHLA | Forkhead box protein P4 (Fork head-related protein-like A) | PVTPLRP | 418 | 424 |
| **Q8IVL1** | NAV2 HELAD1 KIAA1419 POMFIL2 RAINB1 STEERIN2 | Neuron navigator 2 (EC 3.6.4.12) (Helicase APC down-regulated 1) (Pore membrane and/or filament-interacting-like protein 2) (Retinoic acid inducible in neuroblastoma 1) (Steerin-2) (Unc-53 homolog 2) (unc53H2) | PVTPQAP | 230 | 236 |
| **Q8IVT2** | MISP C19orf21 | Mitotic interactor and substrate of PLK1 (Mitotic spindle positioning protein) | PRTPGPP | 170 | 176 |
| **Q8IVT5** | KSR1 KSR | Kinase suppressor of Ras 1 | PRTPPPP | 286 | 292 |
| **Q8IVW8** | SPNS2 | Protein spinster homolog 2 | PGTPGTP | 72 | 78 |
| **Q8IW52** | SLITRK4 | SLIT and NTRK-like protein 4 | PLTPCP | 338 | 343 |
| **Q8IW92** | GLB1L2 MSTP014 UNQ210/PRO236 | Beta-galactosidase-1-like protein 2 (EC 3.2.1.-) | PETPTLP | 542 | 548 |
| **Q8IWA4** | MFN1 | Mitofusin-1 (EC 3.6.5.-) (Fzo homolog) (Transmembrane GTPase MFN1) | PTTPATP | 566 | 572 |
| **Q8IWB1** | ITPRIP DANGER KIAA1754 | Inositol 1,4,5-trisphosphate receptor-interacting protein (Protein DANGER) | PFTPPEP | 213 | 219 |
| **Q8IWB4** | SPATA31A7 FAM75A4 FAM75A7 SPATA31A4 | Spermatogenesis-associated protein 31A7 (Protein FAM75A7) | PHTPDP | 208 | 213 |
| **Q8IWE2** | FAM114A1 NOXP20 | Protein NOXP20 (Nervous system overexpressed protein 20) (Protein FAM114A1) | PTPADP | 43 | 48 |
| **Q8IWN7** | RP1L1 | Retinitis pigmentosa 1-like 1 protein | PKTPSGP | 110 | 116 |
| **Q8IWY9** | CDAN1 UNQ664/PRO1295 | Codanin-1 | PTPTCP | 266 | 271 |
| **Q8IWZ8** | SUGP1 SF4 | SURP and G-patch domain-containing protein 1 (RNA-binding protein RBP) (Splicing factor 4) | PSTPTP | 114 | 119 |
| **Q8IX07** | ZFPM1 FOG1 ZFN89A | Zinc finger protein ZFPM1 (Friend of GATA protein 1) (FOG-1) (Friend of GATA 1) (Zinc finger protein 89A) (Zinc finger protein multitype 1) | PTPSP | 422 | 426 |
| **Q8IX15** | HOMEZ KIAA1443 | Homeobox and leucine zipper protein Homez (Homeodomain leucine zipper-containing factor) | PTPPP | 436 | 440 |
| **Q8IXK0** | PHC2 EDR2 PH2 | Polyhomeotic-like protein 2 (hPH2) (Early development regulatory protein 2) | PTPTQP | 240 | 245 |
| **Q8IXK2** | GALNT12 | Polypeptide N-acetylgalactosaminyltransferase 12 (EC 2.4.1.41) (Polypeptide GalNAc transferase 12) (GalNAc-T12) (pp-GaNTase 12) (Protein-UDP acetylgalactosaminyltransferase 12) (UDP-GalNAc:polypeptide N-acetylgalactosaminyltransferase 12) | PRTPRP | 53 | 58 |
| **Q8IXP5** | C11orf53 | Uncharacterized protein C11orf53 | PTPPP | 177 | 181 |
| **Q8IY17** | PNPLA6 NTE | Neuropathy target esterase (EC 3.1.1.5) (Patatin-like phospholipase domain-containing protein 6) | PKTPAP | 77 | 82 |
| **Q8IY84** | NIM1K NIM1 | Serine/threonine-protein kinase NIM1 (EC 2.7.11.1) (NIM1 serine/threonine-protein kinase) | PTPLEP | 331 | 336 |
| **Q8IY92** | SLX4 BTBD12 KIAA1784 KIAA1987 | Structure-specific endonuclease subunit SLX4 (BTB/POZ domain-containing protein 12) | PQTPPP | 1313 | 1318 |
| **Q8IYB3** | SRRM1 SRM160 | Serine/arginine repetitive matrix protein 1 (SR-related nuclear matrix protein of 160 kDa) (SRm160) (Ser/Arg-related nuclear matrix protein) | PATPPP | 404 | 409 |
| **Q8IYG6** | LRRC56 | Leucine-rich repeat-containing protein 56 | PKTPSSP | 427 | 433 |
| **Q8IYJ0** | PIANP C12orf53 PANP UNQ828/PRO1755 | PILR alpha-associated neural protein (PILR-associating neural protein) (Paired immunoglobin-like type 2 receptor-associating neural protein) | PRTPPAP | 36 | 42 |
| **Q8IYK2** | CCDC105 | Coiled-coil domain-containing protein 105 | PRTPPP | 486 | 491 |
| **Q8IYK8** | REM2 | GTP-binding protein REM 2 (Rad and Gem-like GTP-binding protein 2) | PGTPTP | 29 | 34 |
| **Q8IYR2** | SMYD4 KIAA1936 | SET and MYND domain-containing protein 4 (EC 2.1.1.-) | PPTPVGP | 796 | 802 |
| **Q8IYY4** | DZIP1L | Zinc finger protein DZIP1L (DAZ-interacting protein 1-like protein) | PSTPAEP | 563 | 569 |
| **Q8IZ21** | PHACTR4 PRO2963 | Phosphatase and actin regulator 4 | PRTPPFP | 356 | 362 |
| **Q8IZ69** | TRMT2A HTF9C | tRNA (uracil-5-)-methyltransferase homolog A (EC 2.1.1.-) (HpaII tiny fragments locus 9c protein) | PTPGPP | 607 | 612 |
| **Q8IZC6** | COL27A1 KIAA1870 | Collagen alpha-1(XXVII) chain | PATPTKP | 294 | 300 |
| **Q8IZD2** | KMT2E MLL5 | Histone-lysine N-methyltransferase 2E (Lysine N-methyltransferase 2E) (EC 2.1.1.43) (Myeloid/lymphoid or mixed-lineage leukemia protein 5) | PTPSP | 890 | 894 |
| **Q8IZD4** | DCP1B | mRNA-decapping enzyme 1B (EC 3.-.-.-) | PSTPAP | 371 | 376 |
| **Q8IZL8** | PELP1 HMX3 MNAR | Proline-, glutamic acid- and leucine-rich protein 1 (Modulator of non-genomic activity of estrogen receptor) (Transcription factor HMX3) | PTPAP | 646 | 650 |
| **Q8IZN3** | ZDHHC14 | Probable palmitoyltransferase ZDHHC14 (EC 2.3.1.225) (NEW1 domain-containing protein) (NEW1CP) (Zinc finger DHHC domain-containing protein 14) (DHHC-14) | PDTPQP | 332 | 337 |
| **Q8IZP0** | ABI1 SSH3BP1 | Abl interactor 1 (Abelson interactor 1) (Abi-1) (Abl-binding protein 4) (AblBP4) (Eps8 SH3 domain-binding protein) (Eps8-binding protein) (Nap1-binding protein) (Nap1BP) (Spectrin SH3 domain-binding protein 1) (e3B1) | PTPSPP | 264 | 269 |
| **Q8IZQ1** | WDFY3 KIAA0993 | WD repeat and FYVE domain-containing protein 3 (Autophagy-linked FYVE protein) (Alfy) | PETPAP | 3252 | 3257 |
| **Q8IZW8** | TNS4 CTEN PP14434 | Tensin-4 (C-terminal tensin-like protein) | PRTPHSP | 345 | 351 |
| **Q8IZX4** | TAF1L | Transcription initiation factor TFIID subunit 1-like (TAF(II)210) (TBP-associated factor 1-like) (TBP-associated factor 210 kDa) (Transcription initiation factor TFIID 210 kDa subunit) | PMTPGP | 1655 | 1660 |
| **Q8IZY2** | ABCA7 | ATP-binding cassette sub-family A member 7 (ABCA-SSN) (Autoantigen SS-N) (Macrophage ABC transporter) | PTPLDP | 787 | 792 |
| **Q8N145** | LGI3 LGIL4 UNQ8190/PRO23199 | Leucine-rich repeat LGI family member 3 (LGI1-like protein 4) (Leucine-rich glioma-inactivated protein 3) | PKTPPCP | 34 | 40 |
| **Q8N1G0** | ZNF687 KIAA1441 | Zinc finger protein 687 | PGTPHSP | 135 | 141 |
| **Q8N1G1** | REXO1 ELOABP1 KIAA1138 TCEB3BP1 | RNA exonuclease 1 homolog (EC 3.1.-.-) (Elongin-A-binding protein 1) (EloA-BP1) (Transcription elongation factor B polypeptide 3-binding protein 1) | PYTPAP | 267 | 272 |
| **Q8N1I0** | DOCK4 KIAA0716 | Dedicator of cytokinesis protein 4 | PTPVEP | 1728 | 1733 |
| **Q8N1N2** | DYNAP C18orf26 | Dynactin-associated protein (Full) | PGTPSP | 162 | 167 |
| **Q8N1W2** | ZNF710 | Zinc finger protein 710 | PRTPRP | 175 | 180 |
| **Q8N2Y8** | RUSC2 KIAA0375 | Iporin (Interacting protein of Rab1) (RUN and SH3 domain-containing protein 2) | PTPSP | 1505 | 1509 |
| **Q8N365** | CIART C1orf51 | Circadian-associated transcriptional repressor (ChIP-derived repressor of network oscillator) (Chrono) (Computationally highlighted repressor of the network oscillator) | PTTPVPP | 308 | 314 |
| **Q8N398** | VWA5B2 | von Willebrand factor A domain-containing protein 5B2 | PTPAP | 739 | 743 |
| **Q8N3D4** | EHBP1L1 | EH domain-binding protein 1-like protein 1 | PTPAP | 300 | 304 |
| **Q8N3F8** | MICALL1 KIAA1668 MIRAB13 | MICAL-like protein 1 (Molecule interacting with Rab13) (MIRab13) | PTPVEP | 132 | 137 |
| **Q8N3J6** | CADM2 IGSF4D NECL3 | Cell adhesion molecule 2 (Immunoglobulin superfamily member 4D) (IgSF4D) (Nectin-like protein 3) (NECL-3) (Synaptic cell adhesion molecule 2) (SynCAM 2) | PSTPFP | 233 | 238 |
| **Q8N3L3** | TXLNB C6orf198 MDP77 | Beta-taxilin (Muscle-derived protein 77) (hMDP77) | PTPVQP | 39 | 44 |
| **Q8N3X1** | FNBP4 FBP30 KIAA1014 | Formin-binding protein 4 (Formin-binding protein 30) | PPTPPRP | 170 | 176 |
| **Q8N3Z6** | ZCCHC7 HSPC086 | Zinc finger CCHC domain-containing protein 7 (TRAMP-like complex RNA-binding factor ZCCHC7) | PKTPSRP | 338 | 344 |
| **Q8N441** | FGFRL1 FGFR5 FHFR UNQ480/PRO943 | Fibroblast growth factor receptor-like 1 (FGF receptor-like protein 1) (FGF homologous factor receptor) (FGFR-like protein) (Fibroblast growth factor receptor 5) (FGFR-5) | PCTPAP | 405 | 410 |
| **Q8N465** | D2HGDH D2HGD | D-2-hydroxyglutarate dehydrogenase, mitochondrial (EC 1.1.99.-) | PGTPEVP | 40 | 46 |
| **Q8N4X5** | AFAP1L2 KIAA1914 XB130 | Actin filament-associated protein 1-like 2 (AFAP1-like protein 2) | PTPDEP | 580 | 585 |
| **Q8N531** | FBXL6 FBL6 | F-box/LRR-repeat protein 6 (F-box and leucine-rich repeat protein 6) (F-box protein FBL6) (FBL6A) | PTPTP | 99 | 103 |
| **Q8N568** | DCLK2 DCAMKL2 DCDC3B DCK2 | Serine/threonine-protein kinase DCLK2 (EC 2.7.11.1) (CaMK-like CREB regulatory kinase 2) (CL2) (CLICK-II) (CLICK2) (Doublecortin domain-containing protein 3B) (Doublecortin-like and CAM kinase-like 2) (Doublecortin-like kinase 2) | PTPHPP | 744 | 749 |
| **Q8N5G0** | SMIM20 C4orf52 | Small integral membrane protein 20 | PTPAP | 38 | 42 |
| **Q8N5U0** | C11orf42 | Uncharacterized protein C11orf42 | PPTPPP | 258 | 263 |
| **Q8N5Y8** | PARP16 ARTD15 C15orf30 | Mono [ADP-ribose] polymerase PARP16 (EC 2.4.2.30) (ADP-ribosyltransferase diphtheria toxin-like 15) (Poly [ADP-ribose] polymerase 16) (PARP-16) | PHTPVP | 115 | 120 |
| **Q8N6C5** | IGSF1 IGDC1 KIAA0364 PGSF2 | Immunoglobulin superfamily member 1 (IgSF1) (Immunoglobulin-like domain-containing protein 1) (Inhibin-binding protein) (InhBP) (Pituitary gland-specific factor 2) (p120) | PTPKP | 588 | 592 |
| **Q8N6N2** | TTC9B | Tetratricopeptide repeat protein 9B (TPR repeat protein 9B) | PTPEP | 46 | 50 |
| **Q8N6P7** | IL22RA1 IL22R | Interleukin-22 receptor subunit alpha-1 (IL-22 receptor subunit alpha-1) (IL-22R-alpha-1) (IL-22RA1) (Cytokine receptor class-II member 9) (Cytokine receptor family 2 member 9) (CRF2-9) (ZcytoR11) | PTPTP | 141 | 145 |
| **Q8N6Q8** | METTL25 C12orf26 | Methyltransferase-like protein 25 (EC 2.1.1.-) | PVTPDLP | 8 | 14 |
| **Q8N6S5** | ARL6IP6 PFAAP1 | ADP-ribosylation factor-like protein 6-interacting protein 6 (ARL-6-interacting protein 6) (Aip-6) (Phosphonoformate immuno-associated protein 1) | PGTPGP | 17 | 22 |
| **Q8N6V9** | TEX9 | Testis-expressed sequence 9 protein | PGTPFPP | 14 | 20 |
| **Q8N6W0** | CELF5 BRUNOL5 | CUGBP Elav-like family member 5 (CELF-5) (Bruno-like protein 5) (CUG-BP- and ETR-3-like factor 5) (RNA-binding protein BRUNOL-5) | PATPIAP | 289 | 295 |
| **Q8N895** | ZNF366 | Zinc finger protein 366 | PTPTP | 164 | 168 |
| **Q8N8E2** | ZNF513 | Zinc finger protein 513 | PPTPPCP | 231 | 237 |
| **Q8N8Q3** | ENDOV | Endonuclease V (hEndoV) (EC 3.1.26.-) (Inosine-specific endoribonuclease) | PPTPRSP | 256 | 262 |
| **Q8N8W4** | PNPLA1 | Patatin-like phospholipase domain-containing protein 1 (EC 3.1.1.-) | PTPPP | 390 | 394 |
| **Q8N944** | AMER3 FAM123C | APC membrane recruitment protein 3 (Amer3) (Protein FAM123C) | PTPRAP | 516 | 521 |
| **Q8N9B5** | JMY | Junction-mediating and -regulatory protein | PPTPPPP | 808 | 814 |
| **Q8N9W5** | DNAAF3 C19orf51 | Dynein assembly factor 3, axonemal | PGTPAAP | 346 | 352 |
| **Q8NAC3** | IL17RC UNQ6118/PRO20040/PRO38901 | Interleukin-17 receptor C (IL-17 receptor C) (IL-17RC) (Interleukin-17 receptor homolog) (IL17Rhom) (Interleukin-17 receptor-like protein) (IL-17RL) (ZcytoR14) | PGTPAP | 770 | 775 |
| **Q8NAG6** | ANKLE1 ANKRD41 LEM3 | Ankyrin repeat and LEM domain-containing protein 1 (Ankyrin repeat domain-containing protein 41) (LEM-domain containing protein 3) | PRTPTP | 307 | 312 |
| **Q8NAJ2** | C9orf106 | Putative uncharacterized protein C9orf106 | PGTPFLP | 41 | 47 |
| **Q8NAV2** | C8orf58 | Uncharacterized protein C8orf58 | PETPVEP | 276 | 282 |
| **Q8NBB4** | ZSCAN1 | Zinc finger and SCAN domain-containing protein 1 | PQTPTP | 14 | 19 |
| **Q8NBS9** | TXNDC5 TLP46 UNQ364/PRO700 | Thioredoxin domain-containing protein 5 (Endoplasmic reticulum resident protein 46) (ER protein 46) (ERp46) (Thioredoxin-like protein p46) | PVTPEP | 172 | 177 |
| **Q8NBZ9** | NEXN-AS1 C1orf118 | Putative uncharacterized protein NEXN-AS1 (NEXN antisense RNA 1) | PTPPSP | 169 | 174 |
| **Q8NC44** | FAM134A C2orf17 | Protein FAM134A | PETPPKP | 511 | 517 |
| **Q8NCK7** | SLC16A11 MCT11 | Monocarboxylate transporter 11 (MCT 11) (Solute carrier family 16 member 11) | PATPPP | 439 | 444 |
| **Q8NCU7** | C2CD4A FAM148A NLF1 | C2 calcium-dependent domain-containing protein 4A (Nuclear-localized factor 1) (Protein FAM148A) | PATPAAP | 156 | 162 |
| **Q8ND04** | SMG8 ABC2 C17orf71 | Protein SMG8 (Amplified in breast cancer gene 2 protein) (Protein smg-8 homolog) | PSTPDP | 655 | 660 |
| **Q8ND24** | RNF214 | RING finger protein 214 | PTPTLP | 435 | 440 |
| **Q8ND90** | PNMA1 MA1 | Paraneoplastic antigen Ma1 (37 kDa neuronal protein) (Neuron- and testis-specific protein 1) | PTPTP | 128 | 132 |
| **Q8NDB2** | BANK1 | B-cell scaffold protein with ankyrin repeats | PTPRP | 614 | 618 |
| **Q8NDC0** | MAPK1IP1L C14orf32 | MAPK-interacting and spindle-stabilizing protein-like (Mitogen-activated protein kinase 1-interacting protein 1-like) | PTPNMP | 117 | 122 |
| **Q8NEM7** | SUPT20H C13orf19 FAM48A FP757 | Transcription factor SPT20 homolog (p38-interacting protein) (p38IP) | PTPPP | 493 | 497 |
| **Q8NEN9** | PDZD8 PDZK8 | PDZ domain-containing protein 8 (Sarcoma antigen NY-SAR-84/NY-SAR-104) | PETPAPP | 84 | 90 |
| **Q8NEP4** | C17orf47 | Uncharacterized protein C17orf47 | PTPRGP | 83 | 88 |
| **Q8NES3** | LFNG | Beta-1,3-N-acetylglucosaminyltransferase lunatic fringe (EC 2.4.1.222) (O-fucosylpeptide 3-beta-N-acetylglucosaminyltransferase) | PDTPWCP | 368 | 374 |
| **Q8NEV1** | CSNK2A3 CSNK2A1P | Casein kinase II subunit alpha 3 (CK II alpha 3) (EC 2.7.11.1) (Casein kinase II alpha 1 polypeptide pseudogene) | PTPSP | 359 | 363 |
| **Q8NEY1** | NAV1 KIAA1151 KIAA1213 POMFIL3 STEERIN1 | Neuron navigator 1 (Pore membrane and/or filament-interacting-like protein 3) (Steerin-1) (Unc-53 homolog 1) (unc53H1) | PTPPAP | 905 | 910 |
| **Q8NEZ4** | KMT2C HALR KIAA1506 MLL3 | Histone-lysine N-methyltransferase 2C (Lysine N-methyltransferase 2C) (EC 2.1.1.43) (Homologous to ALR protein) (Myeloid/lymphoid or mixed-lineage leukemia protein 3) | PTPLP | 1535 | 1539 |
| **Q8NF64** | ZMIZ2 KIAA1886 ZIMP7 HRIHFB2007 | Zinc finger MIZ domain-containing protein 2 (PIAS-like protein Zimp7) | PGTPGP | 578 | 583 |
| **Q8NFC6** | BOD1L1 BOD1L FAM44A KIAA1327 | Biorientation of chromosomes in cell division protein 1-like 1 | PTPGP | 189 | 193 |
| **Q8NFD5** | ARID1B BAF250B DAN15 KIAA1235 OSA2 | AT-rich interactive domain-containing protein 1B (ARID domain-containing protein 1B) (BRG1-associated factor 250b) (BAF250B) (BRG1-binding protein hELD/OSA1) (Osa homolog 2) (hOsa2) (p250R) | PGTPGP | 503 | 508 |
| **Q8NFH5** | NUP35 MP44 NUP53 | Nucleoporin NUP53 (35 kDa nucleoporin) (Mitotic phosphoprotein 44) (MP-44) (Nuclear pore complex protein Nup53) (Nucleoporin Nup35) | PVTPQP | 46 | 51 |
| **Q8NFH8** | REPS2 POB1 | RalBP1-associated Eps domain-containing protein 2 (Partner of RalBP1) (RalBP1-interacting protein 2) | PPTPPP | 477 | 482 |
| **Q8NFU4** | FDCSP C4orf7 UNQ733/PRO1419 | Follicular dendritic cell secreted peptide (FDC secreted protein) (FDC-SP) | PTTPLP | 77 | 82 |
| **Q8NFU7** | TET1 CXXC6 KIAA1676 LCX | Methylcytosine dioxygenase TET1 (EC 1.14.11.n2) (CXXC-type zinc finger protein 6) (Leukemia-associated protein with a CXXC domain) (Ten-eleven translocation 1 gene protein) | PLTPHQP | 1933 | 1939 |
| **Q8NFY4** | SEMA6D KIAA1479 | Semaphorin-6D | PTTPGVP | 948 | 954 |
| **Q8NG04** | SLC26A10 | Solute carrier family 26 member 10 | PTPIP | 227 | 231 |
| **Q8NH09** | OR8S1 | Olfactory receptor 8S1 | PVTPQP | 337 | 342 |
| **Q8NH80** | OR10D3 OR10D3P | Putative olfactory receptor 10D3 (HTPCRX09) (Olfactory receptor OR11-293) | PTPNP | 262 | 266 |
| **Q8NHY3** | GAS2L2 GAR17 | GAS2-like protein 2 (GAS2-related protein on chromosome 17) (Growth arrest-specific protein 2-like 2) | PPTPSSP | 335 | 341 |
| **Q8NHY6** | ZFP28 KIAA1431 | Zinc finger protein 28 homolog (Zfp-28) (Krueppel-like zinc finger factor X6) | PTPLP | 12 | 16 |
| **Q8NI32** | LYPD6B | Ly6/PLAUR domain-containing protein 6B | PTPFP | 53 | 57 |
| **Q8TAP9** | MPLKIP C7orf11 TTDN1 | M-phase-specific PLK1-interacting protein (TTD non-photosensitive 1 protein) | PPTPPYP | 8 | 14 |
| **Q8TAQ2** | SMARCC2 BAF170 | SWI/SNF complex subunit SMARCC2 (BRG1-associated factor 170) (BAF170) (SWI/SNF complex 170 kDa subunit) (SWI/SNF-related matrix-associated actin-dependent regulator of chromatin subfamily C member 2) | PTPEKP | 229 | 234 |
| **Q8TAY7** | FAM110D GRRP1 | Protein FAM110D | PLTPHP | 49 | 54 |
| **Q8TB05** | UBALD1 FAM100A PP11303 | UBA-like domain-containing protein 1 | PTPPSP | 146 | 151 |
| **Q8TB24** | RIN3 | Ras and Rab interactor 3 (Ras interaction/interference protein 3) | PTPGPP | 493 | 498 |
| **Q8TC44** | POC1B WDR51B | POC1 centriolar protein homolog B (Pix1) (Proteome of centriole protein 1B) (WD repeat-containing protein 51B) | PRTPHP | 330 | 335 |
| **Q8TC57** | M1AP C2orf65 SPATA37 | Meiosis 1 arrest protein (Meiosis 1-arresting protein) (Meiosis 1-associated protein) (Spermatogenesis-associated protein 37) | PMTPVP | 495 | 500 |
| **Q8TCU4** | ALMS1 KIAA0328 | Alstrom syndrome protein 1 | PTPTVP | 993 | 998 |
| **Q8TCY9** | URGCP KIAA1507 URG4 | Up-regulator of cell proliferation (HBV X protein up-regulated gene 4 protein) (HBxAg up-regulated gene 4 protein) | PTPDTP | 175 | 180 |
| **Q8TD55** | PLEKHO2 PLEKHQ1 PP9099 | Pleckstrin homology domain-containing family O member 2 (PH domain-containing family O member 2) (Pleckstrin homology domain-containing family Q member 1) (PH domain-containing family Q member 1) | PPTPPP | 309 | 314 |
| **Q8TD84** | DSCAML1 DSCAM2 KIAA1132 | Down syndrome cell adhesion molecule-like protein 1 (Down syndrome cell adhesion molecule 2) | PTPAEP | 1990 | 1995 |
| **Q8TDC0** | MYOZ3 FRP3 | Myozenin-3 (Calsarcin-3) (FATZ-related protein 3) | PGTPFIP | 213 | 219 |
| **Q8TDF6** | RASGRP4 | RAS guanyl-releasing protein 4 | PSTPAP | 608 | 613 |
| **Q8TDI0** | CHD5 KIAA0444 | Chromodomain-helicase-DNA-binding protein 5 (CHD-5) (EC 3.6.4.12) (ATP-dependent helicase CHD5) | PVTPIP | 1215 | 1220 |
| **Q8TDM6** | DLG5 KIAA0583 PDLG | Disks large homolog 5 (Discs large protein P-dlg) (Placenta and prostate DLG) | PLTPPKP | 1009 | 1015 |
| **Q8TDR0** | TRAF3IP1 IFT54 MIPT3 | TRAF3-interacting protein 1 (Interleukin-13 receptor alpha 1-binding protein 1) (Intraflagellar transport protein 54 homolog) (Microtubule-interacting protein associated with TRAF3) (MIP-T3) | PETPEIP | 442 | 448 |
| **Q8TDY2** | RB1CC1 KIAA0203 RBICC | RB1-inducible coiled-coil protein 1 (FAK family kinase-interacting protein of 200 kDa) (FIP200) | PRTPPP | 668 | 673 |
| **Q8TEH3** | DENND1A FAM31A KIAA1608 | DENN domain-containing protein 1A (Connecdenn 1) (Connecdenn) (Protein FAM31A) | PTPFP | 823 | 827 |
| **Q8TER0** | SNED1 | Sushi, nidogen and EGF-like domain-containing protein 1 (Insulin-responsive sequence DNA-binding protein 1) (IRE-BP1) | PETPTQP | 1237 | 1243 |
| **Q8TES7** | FBF1 ALB KIAA1863 | Fas-binding factor 1 (FBF-1) (Protein albatross) | PVTPSVP | 371 | 377 |
| **Q8TF01** | PNISR C6orf111 SFRS18 SRRP130 HSPC261 HSPC306 | Arginine/serine-rich protein PNISR (PNN-interacting serine/arginine-rich protein) (SR-related protein) (SR-rich protein) (Serine/arginine-rich-splicing regulatory protein 130) (SRrp130) (Splicing factor, arginine/serine-rich 130) (Splicing factor, arginine/serine-rich 18) | PPTPGP | 111 | 116 |
| **Q8TF50** | ZNF526 KIAA1951 | Zinc finger protein 526 | PPTPLPP | 179 | 185 |
| **Q8TF66** | LRRC15 LIB | Leucine-rich repeat-containing protein 15 (Leucine-rich repeat protein induced by beta-amyloid homolog) (hLib) | PTPLP | 48 | 52 |
| **Q8TF74** | WIPF2 WICH WIRE PP10631 | WAS/WASL-interacting protein family member 2 (WASP-interacting protein-related protein) (WIP- and CR16-homologous protein) (WIP-related protein) | PPTPLP | 189 | 194 |
| **Q8WTR8** | NTN5 | Netrin-5 (Netrin-1-like protein) | PATPRHP | 200 | 206 |
| **Q8WTU2** | SSC4D SRCRB4D | Scavenger receptor cysteine-rich domain-containing group B protein (Four scavenger receptor cysteine-rich domains-containing protein) (S4D-SRCRB) | PTPLP | 50 | 54 |
| **Q8WU20** | FRS2 | Fibroblast growth factor receptor substrate 2 (FGFR substrate 2) (FGFR-signaling adaptor SNT) (Suc1-associated neurotrophic factor target 1) (SNT-1) | PRTPRTP | 130 | 136 |
| **Q8WU58** | FAM222B C17orf63 | Protein FAM222B | PTPMP | 316 | 320 |
| **Q8WUA4** | GTF3C2 KIAA0011 | General transcription factor 3C polypeptide 2 (TF3C-beta) (Transcription factor IIIC 110 kDa subunit) (TFIIIC 110 kDa subunit) (TFIIIC110) (Transcription factor IIIC subunit beta) | PTPLP | 54 | 58 |
| **Q8WUF5** | PPP1R13L IASPP NKIP1 PPP1R13BL RAI | RelA-associated inhibitor (Inhibitor of ASPP protein) (Protein iASPP) (NFkB-interacting protein 1) (PPP1R13B-like protein) | PQTPTP | 432 | 437 |
| **Q8WUM4** | PDCD6IP AIP1 ALIX KIAA1375 | Programmed cell death 6-interacting protein (PDCD6-interacting protein) (ALG-2-interacting protein 1) (ALG-2-interacting protein X) (Hp95) | PTPPTP | 737 | 742 |
| **Q8WUQ7** | CACTIN C19orf29 | Cactin (Renal carcinoma antigen NY-REN-24) | PTPPGP | 501 | 506 |
| **Q8WUU4** | ZNF296 ZNF342 | Zinc finger protein 296 (ZFP296) (Zinc finger protein 342) | PLTPNYP | 93 | 99 |
| **Q8WV44** | TRIM41 RINCK | E3 ubiquitin-protein ligase TRIM41 (EC 6.3.2.-) (RING finger-interacting protein with C kinase) (RINCK) (Tripartite motif-containing protein 41) | PVTPLPP | 167 | 173 |
| **Q8WVC0** | LEO1 RDL | RNA polymerase-associated protein LEO1 (Replicative senescence down-regulated leo1-like protein) | PPTPGQP | 331 | 337 |
| **Q8WVN6** | SECTM1 K12 | Secreted and transmembrane protein 1 (Protein K-12) | PTPRP | 216 | 220 |
| **Q8WXA9** | SREK1 SFRS12 SRRP86 | Splicing regulatory glutamine/lysine-rich protein 1 (Serine/arginine-rich-splicing regulatory protein 86) (SRrp86) (Splicing factor, arginine/serine-rich 12) (Splicing regulatory protein 508) (SRrp508) | PTPNP | 14 | 18 |
| **Q8WXD9** | CASKIN1 KIAA1306 | Caskin-1 (CASK-interacting protein 1) | PTPRP | 1028 | 1032 |
| **Q8WXE0** | CASKIN2 KIAA1139 | Caskin-2 (CASK-interacting protein 2) | PTPLRP | 360 | 365 |
| **Q8WXE1** | ATRIP AGS1 | ATR-interacting protein (ATM and Rad3-related-interacting protein) | PETPLP | 596 | 601 |
| **Q8WXI7** | MUC16 CA125 | Mucin-16 (MUC-16) (Ovarian cancer-related tumor marker CA125) (CA-125) (Ovarian carcinoma antigen CA125) | PTPVFP | 894 | 899 |
| **Q8WXS8** | ADAMTS14 | A disintegrin and metalloproteinase with thrombospondin motifs 14 (ADAM-TS 14) (ADAM-TS14) (ADAMTS-14) (EC 3.4.24.-) | PETPIP | 1168 | 1173 |
| **Q8WXX0** | DNAH7 KIAA0944 | Dynein heavy chain 7, axonemal (Axonemal beta dynein heavy chain 7) (Ciliary dynein heavy chain 7) (Dynein heavy chain-like protein 2) (hDHC2) | PLTPAP | 3700 | 3705 |
| **Q8WXX7** | AUTS2 KIAA0442 | Autism susceptibility gene 2 protein | PTPAPP | 557 | 562 |
| **Q8WY21** | SORCS1 SORCS | VPS10 domain-containing receptor SorCS1 (hSorCS) | PATPLP | 66 | 71 |
| **Q8WYB5** | KAT6B KIAA0383 MORF MOZ2 MYST4 | Histone acetyltransferase KAT6B (EC 2.3.1.48) (Histone acetyltransferase MOZ2) (MOZ, YBF2/SAS3, SAS2 and TIP60 protein 4) (MYST-4) (Monocytic leukemia zinc finger protein-related factor) | PETPMEP | 1277 | 1283 |
| **Q8WYP3** | RIN2 RASSF4 | Ras and Rab interactor 2 (Ras association domain family 4) (Ras inhibitor JC265) (Ras interaction/interference protein 2) | PTPIPP | 353 | 358 |
| **Q8WYQ3** | CHCHD10 C22orf16 | Coiled-coil-helix-coiled-coil-helix domain-containing protein 10, mitochondrial (Protein N27C7-4) | PTPAAP | 89 | 94 |
| **Q8WZA9** | IRGQ IRGQ1 FKSG27 | Immunity-related GTPase family Q protein | PTPFP | 261 | 265 |
| **Q92538** | GBF1 KIAA0248 | Golgi-specific brefeldin A-resistance guanine nucleotide exchange factor 1 (BFA-resistant GEF 1) | PTPDGP | 1795 | 1800 |
| **Q92545** | TMEM131 KIAA0257 RW1 | Transmembrane protein 131 (Protein RW1) | PTPASP | 1600 | 1605 |
| **Q92547** | TOPBP1 KIAA0259 | DNA topoisomerase 2-binding protein 1 (DNA topoisomerase II-beta-binding protein 1) (TopBP1) (DNA topoisomerase II-binding protein 1) | PTPQAP | 1230 | 1235 |
| **Q92558** | WASF1 KIAA0269 SCAR1 WAVE1 | Wiskott-Aldrich syndrome protein family member 1 (WASP family protein member 1) (Protein WAVE-1) (Verprolin homology domain-containing protein 1) | PTPPPP | 322 | 327 |
| **Q92570** | NR4A3 CHN CSMF MINOR NOR1 TEC | Nuclear receptor subfamily 4 group A member 3 (Mitogen-induced nuclear orphan receptor) (Neuron-derived orphan receptor 1) (Nuclear hormone receptor NOR-1) | PSTPTTP | 140 | 146 |
| **Q92615** | LARP4B KIAA0217 LARP5 | La-related protein 4B (La ribonucleoprotein domain family member 4B) (La ribonucleoprotein domain family member 5) (La-related protein 5) | PTPPKP | 517 | 522 |
| **Q92664** | GTF3A | Transcription factor IIIA (TFIIIA) | PTPPRP | 29 | 34 |
| **Q92729** | PTPRU FMI PCP2 PTPRO | Receptor-type tyrosine-protein phosphatase U (R-PTP-U) (EC 3.1.3.48) (Pancreatic carcinoma phosphatase 2) (PCP-2) (Protein-tyrosine phosphatase J) (PTP-J) (hPTP-J) (Protein-tyrosine phosphatase pi) (PTP pi) (Protein-tyrosine phosphatase receptor omicron) (PTP-RO) (Receptor-type protein-tyrosine phosphatase psi) (R-PTP-psi) | PPTPIAP | 285 | 291 |
| **Q92777** | SYN2 | Synapsin-2 (Synapsin II) | PTPPPP | 35 | 40 |
| **Q92793** | CREBBP CBP | CREB-binding protein (EC 2.3.1.48) | PTPDP | 614 | 618 |
| **Q92823** | NRCAM KIAA0343 | Neuronal cell adhesion molecule (Nr-CAM) (Neuronal surface protein Bravo) (hBravo) (NgCAM-related cell adhesion molecule) (Ng-CAM-related) | PTPTP | 636 | 640 |
| **Q92830** | KAT2A GCN5 GCN5L2 HGCN5 | Histone acetyltransferase KAT2A (EC 2.3.1.48) (General control of amino acid synthesis protein 5-like 2) (Histone acetyltransferase GCN5) (HsGCN5) (Lysine acetyltransferase 2A) (STAF97) | PTPAP | 8 | 12 |
| **Q92835** | INPP5D SHIP SHIP1 | Phosphatidylinositol 3,4,5-trisphosphate 5-phosphatase 1 (EC 3.1.3.86) (Inositol polyphosphate-5-phosphatase of 145 kDa) (SIP-145) (SH2 domain-containing inositol 5'-phosphatase 1) (SH2 domain-containing inositol phosphatase 1) (SHIP-1) (p150Ship) (hp51CN) | PPTPTP | 1134 | 1139 |
| **Q92859** | NEO1 IGDCC2 NGN | Neogenin (Immunoglobulin superfamily DCC subclass member 2) | PYTPVP | 844 | 849 |
| **Q92908** | GATA6 | Transcription factor GATA-6 (GATA-binding factor 6) | PSTPPSP | 32 | 38 |
| **Q92922** | SMARCC1 BAF155 | SWI/SNF complex subunit SMARCC1 (BRG1-associated factor 155) (BAF155) (SWI/SNF complex 155 kDa subunit) (SWI/SNF-related matrix-associated actin-dependent regulator of chromatin subfamily C member 1) | PTPVP | 374 | 378 |
| **Q92949** | FOXJ1 FKHL13 HFH4 | Forkhead box protein J1 (Forkhead-related protein FKHL13) (Hepatocyte nuclear factor 3 forkhead homolog 4) (HFH-4) | PHTPGKP | 85 | 91 |
| **Q92954** | PRG4 MSF SZP | Proteoglycan 4 (Lubricin) (Megakaryocyte-stimulating factor) (Superficial zone proteoglycan) [Cleaved into: Proteoglycan 4 C-terminal part] | PTPKPP | 212 | 217 |
| **Q92982** | NINJ1 | Ninjurin-1 (Nerve injury-induced protein 1) | PGTPGSP | 16 | 22 |
| **Q92993** | KAT5 HTATIP TIP60 | Histone acetyltransferase KAT5 (EC 2.3.1.48) (60 kDa Tat-interactive protein) (Tip60) (Histone acetyltransferase HTATIP) (HIV-1 Tat interactive protein) (Lysine acetyltransferase 5) (cPLA(2)-interacting protein) | PATPVP | 156 | 161 |
| **Q93062** | RBPMS HERMES | RNA-binding protein with multiple splicing (RBP-MS) (Heart and RRM expressed sequence) (Hermes) | PSTPLP | 116 | 121 |
| **Q93073** | SECISBP2L KIAA0256 | Selenocysteine insertion sequence-binding protein 2-like (SECIS-binding protein 2-like) | PTPIP | 47 | 51 |
| **Q93074** | MED12 ARC240 CAGH45 HOPA KIAA0192 TNRC11 TRAP230 | Mediator of RNA polymerase II transcription subunit 12 (Activator-recruited cofactor 240 kDa component) (ARC240) (CAG repeat protein 45) (Mediator complex subunit 12) (OPA-containing protein) (Thyroid hormone receptor-associated protein complex 230 kDa component) (Trap230) (Trinucleotide repeat-containing gene 11 protein) | PTTPAP | 329 | 334 |
| **Q969G3** | SMARCE1 BAF57 | SWI/SNF-related matrix-associated actin-dependent regulator of chromatin subfamily E member 1 (BRG1-associated factor 57) (BAF57) | PPTPAP | 10 | 15 |
| **Q969G5** | PRKCDBP SRBC | Protein kinase C delta-binding protein (Cavin-3) (Serum deprivation response factor-related gene product that binds to C-kinase) (hSRBC) | PPTPVKP | 203 | 209 |
| **Q969S2** | NEIL2 | Endonuclease 8-like 2 (EC 3.2.2.-) (EC 4.2.99.18) (DNA glycosylase/AP lyase Neil2) (DNA-(apurinic or apyrimidinic site) lyase Neil2) (Endonuclease VIII-like 2) (Nei homolog 2) (NEH2) (Nei-like protein 2) | PTPEPP | 69 | 74 |
| **Q969T3** | SNX21 C20orf161 SNXL | Sorting nexin-21 (Sorting nexin L) (SNX-L) | PTPPP | 357 | 361 |
| **Q969W3** | FAM104A | Protein FAM104A | PNTPQP | 146 | 151 |
| **Q96A59** | MARVELD3 MRVLDC3 | MARVEL domain-containing protein 3 | PSTPRP | 165 | 170 |
| **Q96A73** | KIAA1191 P33MONOX | Putative monooxygenase p33MONOX (EC 1.-.-.-) (Brain-derived rescue factor p60MONOX) (Flavin monooxygenase motif-containing protein of 33 kDa) | PMTPPP | 42 | 47 |
| **Q96A84** | EMID1 EMU1 | EMI domain-containing protein 1 (Emilin and multimerin domain-containing protein 1) (Emu1) | PPTPATP | 165 | 171 |
| **Q96A98** | PTH2 TIP39 TIPF39 | Tuberoinfundibular peptide of 39 residues (TIP39) (Parathyroid hormone 2) | PATPRP | 54 | 59 |
| **Q96A99** | PTX4 C16orf38 | Pentraxin-4 | PTPTQP | 191 | 196 |
| **Q96AE4** | FUBP1 | Far upstream element-binding protein 1 (FBP) (FUSE-binding protein 1) (DNA helicase V) (hDH V) | PGTPMGP | 477 | 483 |
| **Q96B36** | AKT1S1 PRAS40 | Proline-rich AKT1 substrate 1 (40 kDa proline-rich AKT substrate) | PTPSPP | 89 | 94 |
| **Q96BD0** | SLCO4A1 OATP1 OATP4A1 OATPE SLC21A12 | Solute carrier organic anion transporter family member 4A1 (OATP4A1) (Colon organic anion transporter) (Organic anion transporter polypeptide-related protein 1) (OATP-RP1) (OATPRP1) (POAT) (Organic anion-transporting polypeptide E) (OATP-E) (Sodium-independent organic anion transporter E) (Solute carrier family 21 member 12) | PGTPLSP | 35 | 41 |
| **Q96BT3** | CENPT C16orf56 ICEN22 | Centromere protein T (CENP-T) (Interphase centromere complex protein 22) | PRTPRRP | 25 | 31 |
| **Q96C00** | ZBTB9 | Zinc finger and BTB domain-containing protein 9 | PTPLPP | 4 | 9 |
| **Q96CC6** | RHBDF1 C16orf8 DIST1 IRHOM1 | Inactive rhomboid protein 1 (iRhom1) (Epidermal growth factor receptor-related protein) (Rhomboid 5 homolog 1) (Rhomboid family member 1) (p100hRho) | PHTPVTP | 178 | 184 |
| **Q96CG8** | CTHRC1 UNQ762/PRO1550 | Collagen triple helix repeat-containing protein 1 (Protein NMTC1) | PGTPGIP | 75 | 81 |
| **Q96DB9** | FXYD5 DYSAD IWU1 HSPC113 UNQ2561/PRO6241 | FXYD domain-containing ion transport regulator 5 (Dysadherin) | PTPTWP | 59 | 64 |
| **Q96DN2** | VWCE URG11 | von Willebrand factor C and EGF domain-containing protein (HBV X protein up-regulated gene 11 protein) (HBxAg up-regulated gene 11 protein) | PTPRLP | 321 | 326 |
| **Q96DN6** | MBD6 KIAA1887 | Methyl-CpG-binding domain protein 6 (Methyl-CpG-binding protein MBD6) | PTPGP | 419 | 423 |
| **Q96DR7** | ARHGEF26 SGEF HMFN1864 | Rho guanine nucleotide exchange factor 26 (SH3 domain-containing guanine exchange factor) | PRTPNAP | 147 | 153 |
| **Q96DU7** | ITPKC IP3KC | Inositol-trisphosphate 3-kinase C (EC 2.7.1.127) (Inositol 1,4,5-trisphosphate 3-kinase C) (IP3 3-kinase C) (IP3K C) (InsP 3-kinase C) | PETPEP | 334 | 339 |
| **Q96EG3** | ZNF837 | Zinc finger protein 837 | PRTPKP | 180 | 185 |
| **Q96EH8** | NEURL3 LINCR | E3 ubiquitin-protein ligase NEURL3 (EC 6.3.2.-) (Lung-inducible neuralized-related C3CH4 RING domain protein) (Neuralized-like protein 3) | PTPMP | 179 | 183 |
| **Q96EK2** | PHF21B KIAA1661 | PHD finger protein 21B | PLTPPSP | 212 | 218 |
| **Q96EP9** | SLC10A4 | Sodium/bile acid cotransporter 4 (Na(+)/bile acid cotransporter 4) (Solute carrier family 10 member 4) | PTPTP | 62 | 66 |
| **Q96EV2** | RBM33 PRR8 | RNA-binding protein 33 (Proline-rich protein 8) (RNA-binding motif protein 33) | PVTPQQP | 376 | 382 |
| **Q96EX2** | RNFT2 TMEM118 | RING finger and transmembrane domain-containing protein 2 (Transmembrane protein 118) | PGTPAP | 146 | 151 |
| **Q96EZ8** | MCRS1 INO80Q MSP58 | Microspherule protein 1 (58 kDa microspherule protein) (Cell cycle-regulated factor p78) (INO80 complex subunit J) (MCRS2) | PSTPVPP | 101 | 107 |
| **Q96F45** | ZNF503 NOLZ1 | Zinc finger protein 503 | PSTPVSP | 97 | 103 |
| **Q96F46** | IL17RA IL17R | Interleukin-17 receptor A (IL-17 receptor A) (IL-17RA) (CDw217) (CD antigen CD217) | PDTPEP | 307 | 312 |
| **Q96FS4** | SIPA1 SPA1 | Signal-induced proliferation-associated protein 1 (Sipa-1) (GTPase-activating protein Spa-1) (p130 SPA-1) | PPTPASP | 62 | 68 |
| **Q96G03** | PGM2 MSTP006 | Phosphoglucomutase-2 (PGM 2) (EC 5.4.2.2) (Glucose phosphomutase 2) (Phosphodeoxyribomutase) (Phosphopentomutase) (EC 5.4.2.7) | PTPFVP | 143 | 148 |
| **Q96G27** | WBP1 | WW domain-binding protein 1 (WBP-1) | PGTPPPP | 134 | 140 |
| **Q96GE9** | TMEM261 C9orf123 | Transmembrane protein 261 | PATPGAP | 29 | 35 |
| **Q96GP6** | SCARF2 SREC2 SREPCR | Scavenger receptor class F member 2 (SRECRP-1) (Scavenger receptor expressed by endothelial cells 2 protein) (SREC-II) | PTPRPP | 724 | 729 |
| **Q96GS4** | C17orf59 | Uncharacterized protein C17orf59 | PTPTP | 247 | 251 |
| **Q96GY3** | LIN37 MSTP064 | Protein lin-37 homolog (Antolefinin) | PPTPPGP | 165 | 171 |
| **Q96HA1** | POM121 KIAA0618 NUP121 POM121A | Nuclear envelope pore membrane protein POM 121 (Nuclear envelope pore membrane protein POM 121A) (Nucleoporin Nup121) (Pore membrane protein of 121 kDa) | PTPPGP | 743 | 748 |
| **Q96HD9** | ACY3 ASPA2 | N-acyl-aromatic-L-amino acid amidohydrolase (carboxylate-forming) (EC 3.5.1.114) (Acylase III) (Aminoacylase-3) (ACY-3) (Aspartoacylase-2) (Hepatitis C virus core-binding protein 1) (HCBP1) (HCV core-binding protein 1) | PTPDDP | 82 | 87 |
| **Q96HE9** | PRR11 | Proline-rich protein 11 | PTPTLP | 345 | 350 |
| **Q96IG2** | FBXL20 FBL2 | F-box/LRR-repeat protein 20 (F-box and leucine-rich repeat protein 20) (F-box/LRR-repeat protein 2-like) | PVTPPP | 415 | 420 |
| **Q96IQ7** | VSIG2 CTH CTXL UNQ2770/PRO7154 | V-set and immunoglobulin domain-containing protein 2 (Cortical thymocyte-like protein) (CT-like protein) | PTPSP | 186 | 190 |
| **Q96IU2** | ZBED3 | Zinc finger BED domain-containing protein 3 (Axin-interacting protein) | PTPTPP | 33 | 38 |
| **Q96IV0** | NGLY1 PNG1 | Peptide-N(4)-(N-acetyl-beta-glucosaminyl)asparagine amidase (PNGase) (hPNGase) (EC 3.5.1.52) (N-glycanase 1) (Peptide:N-glycanase) | PKTPKP | 447 | 452 |
| **Q96J02** | ITCH | E3 ubiquitin-protein ligase Itchy homolog (Itch) (EC 6.3.2.-) (Atrophin-1-interacting protein 4) (AIP4) (NFE2-associated polypeptide 1) (NAPP1) | PPTPRRP | 261 | 267 |
| **Q96J84** | KIRREL KIRREL1 NEPH1 | Kin of IRRE-like protein 1 (Kin of irregular chiasm-like protein 1) (Nephrin-like protein 1) | PTPPGP | 665 | 670 |
| **Q96J86** | CYYR1 C21orf95 | Cysteine and tyrosine-rich protein 1 (Proline-rich domain-containing protein) | PTPQGP | 134 | 139 |
| **Q96J92** | WNK4 PRKWNK4 | Serine/threonine-protein kinase WNK4 (EC 2.7.11.1) (Protein kinase lysine-deficient 4) (Protein kinase with no lysine 4) | PGTPLSP | 807 | 813 |
| **Q96JB3** | HIC2 HRG22 KIAA1020 ZBTB30 | Hypermethylated in cancer 2 protein (Hic-2) (HIC1-related gene on chromosome 22 protein) (Hic-3) (Zinc finger and BTB domain-containing protein 30) | PATPGP | 258 | 263 |
| **Q96JG8** | MAGED4 KIAA1859 MAGED4A MAGEE1; MAGED4B | Melanoma-associated antigen D4 (MAGE-D4 antigen) (MAGE-E1 antigen) | PTPEPP | 159 | 164 |
| **Q96JG9** | ZNF469 KIAA1858 | Zinc finger protein 469 | PQTPRP | 908 | 913 |
| **Q96JH8** | RADIL KIAA1849 | Ras-associating and dilute domain-containing protein | PSTPLGP | 906 | 912 |
| **Q96JJ6** | JPH4 JPHL1 KIAA1831 | Junctophilin-4 (JP-4) (Junctophilin-like 1 protein) | PPTPPPP | 170 | 176 |
| **Q96JK4** | HHIPL1 HHIP2 KIAA1822 UNQ9245/PRO34761 | HHIP-like protein 1 | PTPRP | 637 | 641 |
| **Q96JM2** | ZNF462 KIAA1803 | Zinc finger protein 462 | PTPFP | 1434 | 1438 |
| **Q96JM3** | CHAMP1 C13orf8 CAMP CHAMP KIAA1802 ZNF828 | Chromosome alignment-maintaining phosphoprotein 1 (Zinc finger protein 828) | PTPLTP | 148 | 153 |
| **Q96JN2** | CCDC136 KIAA1793 NAG6 | Coiled-coil domain-containing protein 136 (Nasopharyngeal carcinoma-associated gene 6 protein) | PTPNPP | 1124 | 1129 |
| **Q96JN8** | NEURL4 KIAA1787 | Neuralized-like protein 4 | PPTPIP | 213 | 218 |
| **Q96JQ0** | DCHS1 CDH19 CDH25 FIB1 KIAA1773 PCDH16 | Protocadherin-16 (Cadherin-19) (Cadherin-25) (Fibroblast cadherin-1) (Protein dachsous homolog 1) | PGTPTPP | 782 | 788 |
| **Q96K58** | ZNF668 | Zinc finger protein 668 | PTPLEP | 601 | 606 |
| **Q96K62** | ZBTB45 ZNF499 | Zinc finger and BTB domain-containing protein 45 (Zinc finger protein 499) | PTPLP | 133 | 137 |
| **Q96KG9** | SCYL1 CVAK90 GKLP NTKL TAPK TEIF TRAP HT019 | N-terminal kinase-like protein (Coated vesicle-associated kinase of 90 kDa) (SCY1-like protein 1) (Telomerase regulation-associated protein) (Telomerase transcriptional element-interacting factor) (Teratoma-associated tyrosine kinase) | PTPVP | 612 | 616 |
| **Q96KQ4** | PPP1R13B ASPP1 KIAA0771 | Apoptosis-stimulating of p53 protein 1 (Protein phosphatase 1 regulatory subunit 13B) | PTPLGP | 468 | 473 |
| **Q96KR1** | ZFR | Zinc finger RNA-binding protein (hZFR) (M-phase phosphoprotein homolog) | PATPSP | 215 | 220 |
| **Q96KR7** | PHACTR3 C20orf101 SCAPIN1 | Phosphatase and actin regulator 3 (Scaffold-associated PP1-inhibiting protein) (Scapinin) | PTPPP | 235 | 239 |
| **Q96KV7** | WDR90 C16orf15 C16orf16 C16orf17 C16orf18 C16orf19 KIAA1924 | WD repeat-containing protein 90 | PVTPMP | 197 | 202 |
| **Q96KW2** | POM121L2 POM121L | POM121-like protein 2 | PTPMP | 809 | 813 |
| **Q96L33** | RHOV ARHV WRCH2 | Rho-related GTP-binding protein RhoV (CDC42-like GTPase 2) (GTP-binding protein-like 2) (Rho GTPase-like protein ARHV) (Wnt-1 responsive Cdc42 homolog 2) (WRCH-2) | PTPPP | 17 | 21 |
| **Q96LC7** | SIGLEC10 SLG2 UNQ477/PRO940 | Sialic acid-binding Ig-like lectin 10 (Siglec-10) (Siglec-like protein 2) | PRTPLPP | 620 | 626 |
| **Q96LL9** | DNAJC30 WBSCR18 | DnaJ homolog subfamily C member 30 (Williams-Beuren syndrome chromosomal region 18 protein) | PRTPPP | 138 | 143 |
| **Q96LT9** | RNPC3 KIAA1839 RBM40 RNP | RNA-binding protein 40 (RNA-binding motif protein 40) (RNA-binding region-containing protein 3) (U11/U12 small nuclear ribonucleoprotein 65 kDa protein) (U11/U12 snRNP 65 kDa protein) (U11/U12-65K) | PTPFGP | 196 | 201 |
| **Q96M02** | C10orf90 FATS | Centrosomal protein C10orf90 (Fragile-site associated tumor suppressor homolog) | PTTPEP | 532 | 537 |
| **Q96M27** | PRRC1 | Protein PRRC1 (Proline-rich and coiled-coil-containing protein 1) | PGTPPP | 12 | 17 |
| **Q96MP5** | ZSWIM3 C20orf164 | Zinc finger SWIM domain-containing protein 3 | PTPPP | 435 | 439 |
| **Q96MT7** | CFAP44 WDR52 | Cilia- and flagella-associated protein 44 (WD repeat-containing protein 52) | PSTPSP | 745 | 750 |
| **Q96MX3** | ZNF48 ZNF553 | Zinc finger protein 48 (Zinc finger protein 553) | PLTPRSP | 411 | 417 |
| **Q96MY1** | NOL4L C20orf112 C20orf113 | Nucleolar protein 4-like | PTPTP | 381 | 385 |
| **Q96MY7** | FAM161B C14orf44 | Protein FAM161B | PATPLP | 430 | 435 |
| **Q96N21** | ENTHD2 C17orf56 | AP-4 complex accessory subunit tepsin (ENTH domain-containing protein 2) (Epsin for AP-4) (Tetra-epsin) | PTPPP | 416 | 420 |
| **Q96N66** | MBOAT7 BB1 LENG4 OACT7 | Lysophospholipid acyltransferase 7 (LPLAT 7) (EC 2.3.1.-) (1-acylglycerophosphatidylinositol O-acyltransferase) (EC 2.3.1.n4) (Bladder and breast carcinoma-overexpressed gene 1 protein) (Leukocyte receptor cluster member 4) (Lysophosphatidylinositol acyltransferase) (LPIAT) (Lyso-PI acyltransferase) (Membrane-bound O-acyltransferase domain-containing protein 7) (O-acyltransferase domain-containing protein 7) (h-mboa-7) | PTPTP | 95 | 99 |
| **Q96NR7** | WWC2-AS2 C4orf38 | Putative uncharacterized protein WWC2-AS2 (WWC2 antisense RNA 2) (WWC2 antisense gene protein 2) | PWTPLQP | 176 | 182 |
| **Q96NW7** | LRRC7 KIAA1365 LAP1 | Leucine-rich repeat-containing protein 7 (Densin-180) (Densin) (Protein LAP1) | PTPYP | 569 | 573 |
| **Q96P26** | NT5C1B AIRP FKSG85 | Cytosolic 5'-nucleotidase 1B (cN1B) (EC 3.1.3.5) (Autoimmune infertility-related protein) (Cytosolic 5'-nucleotidase IB) (cN-IB) | PPTPPEP | 193 | 199 |
| **Q96P48** | ARAP1 CENTD2 KIAA0782 | Arf-GAP with Rho-GAP domain, ANK repeat and PH domain-containing protein 1 (Centaurin-delta-2) (Cnt-d2) | PTPRP | 77 | 81 |
| **Q96P71** | NECAB3 APBA2BP NIP1 SYTIP2 XB51 | N-terminal EF-hand calcium-binding protein 3 (Amyloid beta A4 protein-binding family A member 2-binding protein) (Nek2-interacting protein 1) (Neuronal calcium-binding protein 3) (X11L-binding protein 51) | PQTPRHP | 21 | 27 |
| **Q96PE1** | ADGRA2 GPR124 KIAA1531 TEM5 | Adhesion G protein-coupled receptor A2 (G-protein coupled receptor 124) (Tumor endothelial marker 5) | PTPSP | 879 | 883 |
| **Q96PE2** | ARHGEF17 KIAA0337 TEM4 | Rho guanine nucleotide exchange factor 17 (164 kDa Rho-specific guanine-nucleotide exchange factor) (p164-RhoGEF) (p164RhoGEF) (Tumor endothelial marker 4) | PGTPSP | 148 | 153 |
| **Q96PM9** | ZNF385A HZF RZF ZNF385 | Zinc finger protein 385A (Hematopoietic zinc finger protein) (Retinal zinc finger protein) | PPTPGEP | 246 | 252 |
| **Q96PN7** | TRERF1 BCAR2 RAPA TREP132 | Transcriptional-regulating factor 1 (Breast cancer anti-estrogen resistance 2) (Transcriptional-regulating protein 132) (Zinc finger protein rapa) (Zinc finger transcription factor TReP-132) | PYTPPP | 707 | 712 |
| **Q96PP9** | GBP4 | Guanylate-binding protein 4 (EC 3.6.5.-) (GTP-binding protein 4) (GBP-4) (Guanine nucleotide-binding protein 4) | PTPGYP | 11 | 16 |
| **Q96PU8** | QKI HKQ | Protein quaking (Hqk) (HqkI) | PTPAGP | 244 | 249 |
| **Q96PV7** | FAM193B IRIZIO KIAA1931 | Protein FAM193B | PTTPAAP | 279 | 285 |
| **Q96PX1** | RNF157 KIAA1917 | RING finger protein 157 | PLTPSP | 376 | 381 |
| **Q96PY5** | FMNL2 FHOD2 KIAA1902 | Formin-like protein 2 (Formin homology 2 domain-containing protein 2) | PVTPPMP | 547 | 553 |
| **Q96Q04** | LMTK3 KIAA1883 TYKLM3 | Serine/threonine-protein kinase LMTK3 (EC 2.7.11.1) (Lemur tyrosine kinase 3) | PSTPPAP | 1383 | 1389 |
| **Q96QC0** | PPP1R10 CAT53 FB19 PNUTS | Serine/threonine-protein phosphatase 1 regulatory subunit 10 (MHC class I region proline-rich protein CAT53) (PP1-binding protein of 114 kDa) (Phosphatase 1 nuclear targeting subunit) (Protein FB19) (p99) | PGTPVPP | 352 | 358 |
| **Q96QP1** | ALPK1 KIAA1527 LAK | Alpha-protein kinase 1 (EC 2.7.11.-) (Chromosome 4 kinase) (Lymphocyte alpha-protein kinase) | PATPIAP | 139 | 145 |
| **Q96QT4** | TRPM7 CHAK1 LTRPC7 | Transient receptor potential cation channel subfamily M member 7 (EC 2.7.11.1) (Channel-kinase 1) (Long transient receptor potential channel 7) (LTrpC-7) (LTrpC7) | PFTPVPP | 1571 | 1577 |
| **Q96QU1** | PCDH15 USH1F | Protocadherin-15 | PTPLPP | 1815 | 1820 |
| **Q96RJ3** | TNFRSF13C BAFFR BR3 | Tumor necrosis factor receptor superfamily member 13C (B-cell-activating factor receptor) (BAFF receptor) (BAFF-R) (BLyS receptor 3) (CD antigen CD268) | PTPCVP | 16 | 21 |
| **Q96RK0** | CIC KIAA0306 | Protein capicua homolog | PTPGIP | 882 | 887 |
| **Q96RM1** | SPRR2F | Small proline-rich protein 2F (SPR-2F) | PVTPSPP | 56 | 62 |
| **Q96RN5** | MED15 ARC105 CTG7A PCQAP TIG1 TNRC7 | Mediator of RNA polymerase II transcription subunit 15 (Activator-recruited cofactor 105 kDa component) (ARC105) (CTG repeat protein 7a) (Mediator complex subunit 15) (Positive cofactor 2 glutamine/Q-rich-associated protein) (PC2 glutamine/Q-rich-associated protein) (TPA-inducible gene 1 protein) (TIG-1) (Trinucleotide repeat-containing gene 7 protein) | PTPPPP | 602 | 607 |
| **Q96RP3** | UCN2 SRP URP | Urocortin-2 (Stresscopin-related peptide) (Urocortin II) (Ucn II) (Urocortin-related peptide) | PVTPIP | 21 | 26 |
| **Q96RR1** | PEO1 C10orf2 | Twinkle protein, mitochondrial (EC 3.6.4.12) (Progressive external ophthalmoplegia 1 protein) (T7 gp4-like protein with intramitochondrial nucleoid localization) (T7-like mitochondrial DNA helicase) | PTPDQP | 672 | 677 |
| **Q96RT1** | ERBB2IP ERBIN KIAA1225 LAP2 | Protein LAP2 (Densin-180-like protein) (Erbb2-interacting protein) (Erbin) | PTPYP | 484 | 488 |
| **Q96RT7** | TUBGCP6 GCP6 KIAA1669 | Gamma-tubulin complex component 6 (GCP-6) | PNTPRP | 1288 | 1293 |
| **Q96S07** | PRR25 gs64 | Proline-rich protein 25 | PPTPNP | 132 | 137 |
| **Q96SL8** | FIZ1 ZNF798 | Flt3-interacting zinc finger protein 1 (Zinc finger protein 798) | PTPAP | 7 | 11 |
| **Q96SQ7** | ATOH8 ATH6 BHLHA21 | Protein atonal homolog 8 (Class A basic helix-loop-helix protein 21) (bHLHa21) (Helix-loop-helix protein hATH-6) (hATH6) | PTPPPP | 122 | 127 |
| **Q96SQ9** | CYP2S1 UNQ891/PRO1906 | Cytochrome P450 2S1 (EC 1.14.14.1) (CYPIIS1) | PTPLP | 36 | 40 |
| **Q96ST8** | CEP89 CCDC123 | Centrosomal protein of 89 kDa (Cep89) (Centrosomal protein 123) (Cep123) (Coiled-coil domain-containing protein 123) | PRTPPP | 33 | 38 |
| **Q96T25** | ZIC5 | Zinc finger protein ZIC 5 (Zinc finger protein of the cerebellum 5) | PPTPSPP | 158 | 164 |
| **Q96T58** | SPEN KIAA0929 MINT SHARP | Msx2-interacting protein (SMART/HDAC1-associated repressor protein) (SPEN homolog) | PTPTP | 3243 | 3247 |
| **Q99490** | AGAP2 CENTG1 KIAA0167 | Arf-GAP with GTPase, ANK repeat and PH domain-containing protein 2 (AGAP-2) (Centaurin-gamma-1) (Cnt-g1) (GTP-binding and GTPase-activating protein 2) (GGAP2) (Phosphatidylinositol 3-kinase enhancer) (PIKE) | PPTPSP | 296 | 301 |
| **Q99501** | GAS2L1 GAR22 | GAS2-like protein 1 (GAS2-related protein on chromosome 22) (Growth arrest-specific protein 2-like 1) | PETPPRP | 332 | 338 |
| **Q99583** | MNT BHLHD3 ROX | Max-binding protein MNT (Class D basic helix-loop-helix protein 3) (bHLHd3) (Myc antagonist MNT) (Protein ROX) | PTPAP | 144 | 148 |
| **Q99607** | ELF4 ELFR MEF | ETS-related transcription factor Elf-4 (E74-like factor 4) (Myeloid Elf-1-like factor) | PTPAP | 642 | 646 |
| **Q99618** | CDCA3 C8 GRCC8 TOME1 | Cell division cycle-associated protein 3 (Gene-rich cluster protein C8) (Trigger of mitotic entry protein 1) (TOME-1) | PVTPARP | 8 | 14 |
| **Q99638** | RAD9A | Cell cycle checkpoint control protein RAD9A (hRAD9) (EC 3.1.11.2) (DNA repair exonuclease rad9 homolog A) | PGTPPP | 353 | 358 |
| **Q99665** | IL12RB2 | Interleukin-12 receptor subunit beta-2 (IL-12 receptor subunit beta-2) (IL-12R subunit beta-2) (IL-12R-beta-2) (IL-12RB2) | PTPEDP | 697 | 702 |
| **Q99697** | PITX2 ARP1 RGS RIEG RIEG1 | Pituitary homeobox 2 (ALL1-responsive protein ARP1) (Homeobox protein PITX2) (Paired-like homeodomain transcription factor 2) (RIEG bicoid-related homeobox transcription factor) (Solurshin) | PTPACP | 258 | 263 |
| **Q99700** | ATXN2 ATX2 SCA2 TNRC13 | Ataxin-2 (Spinocerebellar ataxia type 2 protein) (Trinucleotide repeat-containing gene 13 protein) | PTPASP | 729 | 734 |
| **Q99715** | COL12A1 COL12A1L | Collagen alpha-1(XII) chain | PPTPPPP | 2301 | 2307 |
| **Q99732** | LITAF PIG7 SIMPLE | Lipopolysaccharide-induced tumor necrosis factor-alpha factor (LPS-induced TNF-alpha factor) (Small integral membrane protein of lysosome/late endosome) (p53-induced gene 7 protein) | PTPPAP | 34 | 39 |
| **Q99742** | NPAS1 BHLHE11 MOP5 PASD5 | Neuronal PAS domain-containing protein 1 (Neuronal PAS1) (Basic-helix-loop-helix-PAS protein MOP5) (Class E basic helix-loop-helix protein 11) (bHLHe11) (Member of PAS protein 5) (PAS domain-containing protein 5) | PTPGPP | 203 | 208 |
| **Q99801** | NKX3-1 NKX3.1 NKX3A | Homeobox protein Nkx-3.1 (Homeobox protein NK-3 homolog A) | PPTPSKP | 19 | 25 |
| **Q99954** | SMR3A PBI PROL5 | Submaxillary gland androgen-regulated protein 3A (Proline-rich protein 5) (Proline-rich protein PBI) | PTPAP | 130 | 134 |
| **Q99958** | FOXC2 FKHL14 MFH1 | Forkhead box protein C2 (Forkhead-related protein FKHL14) (Mesenchyme fork head protein 1) (MFH-1 protein) (Transcription factor FKH-14) | PTPQP | 410 | 414 |
| **Q9BQ15** | NABP2 OBFC2B SSB1 LP3587 | SOSS complex subunit B1 (Nucleic acid-binding protein 2) (Oligonucleotide/oligosaccharide-binding fold-containing protein 2B) (Sensor of single-strand DNA complex subunit B1) (Sensor of ssDNA subunit B1) (SOSS-B1) (Single-stranded DNA-binding protein 1) (hSSB1) | PHTPSHP | 168 | 174 |
| **Q9BQ89** | FAM110A C20orf55 F10 | Protein FAM110A | PATPPRP | 138 | 144 |
| **Q9BQ90** | KLHDC3 PEAS | Kelch domain-containing protein 3 (Protein Peas) (Testis intracellular mediator protein) | PPTPVLP | 230 | 236 |
| **Q9BQI5** | SGIP1 | SH3-containing GRB2-like protein 3-interacting protein 1 (Endophilin-3-interacting protein) | PATPDNP | 337 | 343 |
| **Q9BQQ3** | GORASP1 GOLPH5 GRASP65 | Golgi reassembly-stacking protein 1 (Golgi peripheral membrane protein p65) (Golgi phosphoprotein 5) (GOLPH5) (Golgi reassembly-stacking protein of 65 kDa) (GRASP65) | PGTPPP | 214 | 219 |
| **Q9BR26** | OCSTAMP C20orf123 | Osteoclast stimulatory transmembrane protein (OC-STAMP) | PTPRP | 470 | 474 |
| **Q9BRP8** | PYM1 PYM WIBG | Partner of Y14 and mago (PYM homolog 1 exon junction complex-associated factor) (Protein wibg homolog) | PVTPSRP | 70 | 76 |
| **Q9BRQ5** | ORAI3 TMEM142C | Protein orai-3 (Transmembrane protein 142C) | PTPMVP | 184 | 189 |
| **Q9BT40** | INPP5K PPS SKIP | Inositol polyphosphate 5-phosphatase K (EC 3.1.3.56) (Skeletal muscle and kidney-enriched inositol phosphatase) | PDTPIPP | 283 | 289 |
| **Q9BT49** | THAP7 | THAP domain-containing protein 7 | PTTPFSP | 133 | 139 |
| **Q9BT76** | UPK3B | Uroplakin-3b (UP3b) (Uroplakin IIIb) (UPIIIb) (p35) | PGTPTP | 66 | 71 |
| **Q9BTN0** | LRFN3 SALM4 UNQ5865/PRO34192 | Leucine-rich repeat and fibronectin type-III domain-containing protein 3 (Synaptic adhesion-like molecule 4) | PTPTP | 592 | 596 |
| **Q9BTP6** | ZBED2 | Zinc finger BED domain-containing protein 2 | PTPMP | 43 | 47 |
| **Q9BTV7** | CABLES2 C20orf150 | CDK5 and ABL1 enzyme substrate 2 (Interactor with CDK3 2) (Ik3-2) | PTPRP | 264 | 268 |
| **Q9BUJ2** | HNRNPUL1 E1BAP5 HNRPUL1 | Heterogeneous nuclear ribonucleoprotein U-like protein 1 (Adenovirus early region 1B-associated protein 5) (E1B-55 kDa-associated protein 5) (E1B-AP5) | PYTPPPP | 796 | 802 |
| **Q9BUK6** | MSTO1 LST005 SLTP005 | Protein misato homolog 1 | PGTPPP | 432 | 437 |
| **Q9BV87** | CNPPD1 C2orf24 CDABP0125 CGI-57 | Protein CNPPD1 (Cyclin Pas1/PHO80 domain-containing protein 1) | PTPGPP | 263 | 268 |
| **Q9BVT8** | TMUB1 C7orf21 DULP HOPS SB144 UNQ763/PRO1555 | Transmembrane and ubiquitin-like domain-containing protein 1 (Dendritic cell-derived ubiquitin-like protein) (DULP) (Hepatocyte odd protein shuttling protein) (Ubiquitin-like protein SB144) [Cleaved into: iHOPS] | PTPSQP | 47 | 52 |
| **Q9BVV6** | KIAA0586 TALPID3 | Protein TALPID3 | PTPQP | 1041 | 1045 |
| **Q9BW19** | KIFC1 HSET KNSL2 | Kinesin-like protein KIFC1 (Kinesin-like protein 2) (Kinesin-related protein HSET) | PTPPP | 325 | 329 |
| **Q9BW85** | CCDC94 | Coiled-coil domain-containing protein 94 | PTPGAP | 284 | 289 |
| **Q9BWE0** | REPIN1 RIP60 ZNF464 | Replication initiator 1 (60 kDa origin-specific DNA-binding protein) (60 kDa replication initiation region protein) (ATT-binding protein) (DHFR oribeta-binding protein RIP60) (Zinc finger protein 464) | PTPAVP | 343 | 348 |
| **Q9BWG4** | SSBP4 | Single-stranded DNA-binding protein 4 | PGTPIMP | 271 | 277 |
| **Q9BWN1** | PRR14 | Proline-rich protein 14 | PTPPP | 193 | 197 |
| **Q9BWW4** | SSBP3 SSDP SSDP1 | Single-stranded DNA-binding protein 3 (Sequence-specific single-stranded-DNA-binding protein) | PGTPIMP | 276 | 282 |
| **Q9BWW7** | SCRT1 | Transcriptional repressor scratch 1 (Scratch homolog 1 zinc finger protein) (SCRT) (Scratch 1) (hScrt) | PPTPRP | 87 | 92 |
| **Q9BX66** | SORBS1 KIAA0894 KIAA1296 SH3D5 | Sorbin and SH3 domain-containing protein 1 (Ponsin) (SH3 domain protein 5) (SH3P12) (c-Cbl-associated protein) (CAP) | PPTPLGP | 179 | 185 |
| **Q9BX70** | BTBD2 | BTB/POZ domain-containing protein 2 | PTPPAP | 57 | 62 |
| **Q9BXA9** | SALL3 ZNF796 | Sal-like protein 3 (Zinc finger protein 796) (Zinc finger protein SALL3) (hSALL3) | PNTPLP | 767 | 772 |
| **Q9BXF3** | CECR2 KIAA1740 | Cat eye syndrome critical region protein 2 | PYTPQRP | 1368 | 1374 |
| **Q9BXG8** | SPZ1 TSP1 | Spermatogenic leucine zipper protein 1 (Testis-specific protein 1) (Testis-specific protein NYD-TSP1) | PTPDP | 19 | 23 |
| **Q9BXJ3** | C1QTNF4 CTRP4 | Complement C1q tumor necrosis factor-related protein 4 | PTPGP | 19 | 23 |
| **Q9BXK1** | KLF16 BTEB4 NSLP2 | Krueppel-like factor 16 (Basic transcription element-binding protein 4) (BTE-binding protein 4) (Novel Sp1-like zinc finger transcription factor 2) (Transcription factor BTEB4) (Transcription factor NSLP2) | PGTPGPP | 53 | 59 |
| **Q9BXL8** | CDCA4 HEPP | Cell division cycle-associated protein 4 (Hematopoietic progenitor protein) | PATPGP | 215 | 220 |
| **Q9BXP5** | SRRT ARS2 ASR2 | Serrate RNA effector molecule homolog (Arsenite-resistance protein 2) | PGTPPLP | 542 | 548 |
| **Q9BXP8** | PAPPA2 PLAC3 | Pappalysin-2 (EC 3.4.24.-) (Pregnancy-associated plasma protein A2) (PAPP-A2) (Pregnancy-associated plasma protein E1) (PAPP-E) | PTPIP | 843 | 847 |
| **Q9BXX0** | EMILIN2 | EMILIN-2 (Elastin microfibril interface-located protein 2) (Elastin microfibril interfacer 2) (Protein FOAP-10) | PTPARP | 131 | 136 |
| **Q9BY12** | SCAPER KIAA1454 ZNF291 MSTP063 | S phase cyclin A-associated protein in the endoplasmic reticulum (S phase cyclin A-associated protein in the ER) (Zinc finger protein 291) | PATPKIP | 1075 | 1081 |
| **Q9BY44** | EIF2A CDA02 MSTP004 MSTP089 | Eukaryotic translation initiation factor 2A (eIF-2A) (65 kDa eukaryotic translation initiation factor 2A) [Cleaved into: Eukaryotic translation initiation factor 2A, N-terminally processed] | PTPAP | 511 | 515 |
| **Q9BYB0** | SHANK3 KIAA1650 PROSAP2 PSAP2 | SH3 and multiple ankyrin repeat domains protein 3 (Shank3) (Proline-rich synapse-associated protein 2) (ProSAP2) | PTPAFP | 600 | 605 |
| **Q9BYE4** | SPRR2G | Small proline-rich protein 2G (SPR-2G) | PTPKCP | 19 | 24 |
| **Q9BYE9** | CDHR2 PCDH24 PCLKC | Cadherin-related family member 2 (Protocadherin LKC) (PC-LKC) (Protocadherin-24) | PHTPPEP | 1269 | 1275 |
| **Q9BYG4** | PARD6G PAR6G | Partitioning defective 6 homolog gamma (PAR-6 gamma) (PAR6D) | PQTPGAP | 313 | 319 |
| **Q9BYH1** | SEZ6L KIAA0927 UNQ2542/PRO6094 | Seizure 6-like protein | PTTPAP | 194 | 199 |
| **Q9BYH8** | NFKBIZ IKBZ INAP MAIL | NF-kappa-B inhibitor zeta (I-kappa-B-zeta) (IkB-zeta) (IkappaBzeta) (IL-1 inducible nuclear ankyrin-repeat protein) (INAP) (Molecule possessing ankyrin repeats induced by lipopolysaccharide) (MAIL) | PQTPTP | 191 | 196 |
| **Q9BYV9** | BACH2 | Transcription regulator protein BACH2 (BTB and CNC homolog 2) | PTPTAP | 318 | 323 |
| **Q9BYW3** | DEFB126 C20orf8 DEFB26 | Beta-defensin 126 (Beta-defensin 26) (DEFB-26) (Defensin, beta 126) (Epididymal secretory protein 13.2) (ESP13.2) (HBD26) | PTPVSP | 104 | 109 |
| **Q9BZ72** | PITPNM2 KIAA1457 NIR3 | Membrane-associated phosphatidylinositol transfer protein 2 (Phosphatidylinositol transfer protein, membrane-associated 2) (PITPnm 2) (Pyk2 N-terminal domain-interacting receptor 3) (NIR-3) | PTTPGP | 881 | 886 |
| **Q9BZE0** | GLIS2 NKL | Zinc finger protein GLIS2 (GLI-similar 2) (Neuronal Krueppel-like protein) | PTPGSP | 47 | 52 |
| **Q9BZJ8** | GPR61 BALGR | Probable G-protein coupled receptor 61 (Biogenic amine receptor-like G-protein coupled receptor) | PQTPGP | 20 | 25 |
| **Q9BZL4** | PPP1R12C LENG3 MBS85 | Protein phosphatase 1 regulatory subunit 12C (Protein phosphatase 1 myosin-binding subunit of 85 kDa) (Protein phosphatase 1 myosin-binding subunit p85) | PTPSP | 473 | 477 |
| **Q9BZR9** | TRIM8 GERP RNF27 | Probable E3 ubiquitin-protein ligase TRIM8 (EC 6.3.2.-) (Glioblastoma-expressed RING finger protein) (RING finger protein 27) (Tripartite motif-containing protein 8) | PPTPSVP | 507 | 513 |
| **Q9BZS1** | FOXP3 IPEX JM2 | Forkhead box protein P3 (Scurfin) [Cleaved into: Forkhead box protein P3, C-terminally processed; Forkhead box protein P3 41 kDa form] | PTPGP | 427 | 431 |
| **Q9BZY9** | TRIM31 C6orf13 RNF | E3 ubiquitin-protein ligase TRIM31 (EC 6.3.2.-) (Tripartite motif-containing protein 31) | PTPVP | 265 | 269 |
| **Q9C093** | SPEF2 KIAA1770 KPL2 | Sperm flagellar protein 2 (Protein KPL2) | PTPPP | 912 | 916 |
| **Q9C0A1** | ZFHX2 KIAA1056 KIAA1762 ZNF409 | Zinc finger homeobox protein 2 (Zinc finger homeodomain protein 2) (ZFH-2) | PPTPPPP | 1465 | 1471 |
| **Q9C0A6** | SETD5 KIAA1757 | SET domain-containing protein 5 | PVTPPPP | 853 | 859 |
| **Q9C0C6** | CIPC KIAA1737 | CLOCK-interacting pacemaker (CLOCK-interacting circadian protein) | PSTPAPP | 212 | 218 |
| **Q9C0C9** | UBE2O KIAA1734 | (E3-independent) E2 ubiquitin-conjugating enzyme (EC 2.3.2.24) (E2/E3 hybrid ubiquitin-protein ligase UBE2O) (Ubiquitin carrier protein O) (Ubiquitin-conjugating enzyme E2 O) (Ubiquitin-conjugating enzyme E2 of 230 kDa) (Ubiquitin-conjugating enzyme E2-230K) (Ubiquitin-protein ligase O) | PTPAAP | 7 | 12 |
| **Q9C0D6** | FHDC1 KIAA1727 | FH2 domain-containing protein 1 | PKTPSVP | 1017 | 1023 |
| **Q9C0E8** | LNP KIAA1715 | Protein lunapark | PTPASP | 178 | 183 |
| **Q9C0H5** | ARHGAP39 KIAA1688 | Rho GTPase-activating protein 39 | PPTPLP | 461 | 466 |
| **Q9C0I1** | MTMR12 KIAA1682 PIP3AP | Myotubularin-related protein 12 (Phosphatidylinositol 3 phosphate 3-phosphatase adapter subunit) (3-PAP) (3-phosphatase adapter protein) | PTPLP | 246 | 250 |
| **Q9GZW5** | SCAND2P SCAND2 | Putative SCAN domain-containing protein SCAND2P (SCAN domain-containing protein 2 pseudogene) | PTPRRP | 263 | 268 |
| **Q9H091** | ZMYND15 | Zinc finger MYND domain-containing protein 15 | PTPSAP | 716 | 721 |
| **Q9H0A8** | COMMD4 | COMM domain-containing protein 4 | PGTPAQP | 165 | 171 |
| **Q9H0D2** | ZNF541 | Zinc finger protein 541 | PYTPPP | 993 | 998 |
| **Q9H0I2** | ENKD1 C16orf48 UNQ6410/PRO21183 | Enkurin domain-containing protein 1 | PTPPGP | 185 | 190 |
| **Q9H0I9** | TKTL2 | Transketolase-like protein 2 (EC 2.2.1.1) | PTPRLP | 112 | 117 |
| **Q9H0J4** | QRICH2 | Glutamine-rich protein 2 | PVTPAGP | 1442 | 1448 |
| **Q9H0W8** | SMG9 C19orf61 | Protein SMG9 (Protein smg-9 homolog) | PSTPSP | 342 | 347 |
| **Q9H0X6** | RNF208 | RING finger protein 208 | PHTPPLP | 89 | 95 |
| **Q9H161** | ALX4 KIAA1788 | Homeobox protein aristaless-like 4 | PSTPQP | 94 | 99 |
| **Q9H171** | ZBP1 C20orf183 DLM1 | Z-DNA-binding protein 1 (Tumor stroma and activated macrophage protein DLM-1) | PETPGP | 98 | 103 |
| **Q9H195** | MUC3B | Mucin-3B (MUC-3B) (Intestinal mucin-3B) (Fragments) | PTPLP | 794 | 798 |
| **Q9H1B7** | IRF2BPL C14orf4 EAP1 KIAA1865 My039 | Interferon regulatory factor 2-binding protein-like (Enhanced at puberty protein 1) | PPTPAPP | 284 | 290 |
| **Q9H1P6** | C20orf85 | Uncharacterized protein C20orf85 | PTPKP | 59 | 63 |
| **Q9H1U4** | MEGF9 EGFL5 KIAA0818 UNQ671/PRO1305 | Multiple epidermal growth factor-like domains protein 9 (Multiple EGF-like domains protein 9) (Epidermal growth factor-like protein 5) (EGF-like protein 5) | PTTPRTP | 167 | 173 |
| **Q9H211** | CDT1 | DNA replication factor Cdt1 (Double parked homolog) (DUP) | PSTPEAP | 80 | 86 |
| **Q9H2C1** | LHX5 | LIM/homeobox protein Lhx5 (LIM homeobox protein 5) | PDTPSP | 348 | 353 |
| **Q9H2S5** | RNF39 HZFW | RING finger protein 39 (Protein HZFw) | PGTPAPP | 255 | 261 |
| **Q9H2V7** | SPNS1 SPIN1 PP20300 | Protein spinster homolog 1 (HSpin1) (Spinster-like protein 1) | PGTPGLP | 22 | 28 |
| **Q9H2X0** | CHRD UNQ217/PRO243 | Chordin | PGTPGP | 590 | 595 |
| **Q9H2X6** | HIPK2 | Homeodomain-interacting protein kinase 2 (hHIPk2) (EC 2.7.11.1) | PDTPSP | 878 | 883 |
| **Q9H2Y7** | ZNF106 SH3BP3 ZFP106 ZNF474 | Zinc finger protein 106 (Zfp-106) (Zinc finger protein 474) | PTPFFP | 1152 | 1157 |
| **Q9H2Z4** | NKX2-4 NKX2D | Homeobox protein Nkx-2.4 (Homeobox protein NK-2 homolog D) | PTPAP | 306 | 310 |
| **Q9H305** | CDIP1 C16orf5 CDIP LITAFL | Cell death-inducing p53-target protein 1 (Cell death involved p53-target) (Cell death-inducing protein) (LITAF-like protein) (Lipopolysaccharide-induced tumor necrosis factor-alpha-like protein) (Transmembrane protein I1) | PYTPGP | 99 | 104 |
| **Q9H324** | ADAMTS10 | A disintegrin and metalloproteinase with thrombospondin motifs 10 (ADAM-TS 10) (ADAM-TS10) (ADAMTS-10) (EC 3.4.24.-) | PGTPQP | 761 | 766 |
| **Q9H334** | FOXP1 HSPC215 | Forkhead box protein P1 (Mac-1-regulated forkhead) (MFH) | PHTPTTP | 404 | 410 |
| **Q9H361** | PABPC3 PABP3 PABPL3 | Polyadenylate-binding protein 3 (PABP-3) (Poly(A)-binding protein 3) (Testis-specific poly(A)-binding protein) | PSTPSYP | 3 | 9 |
| **Q9H3M7** | TXNIP VDUP1 | Thioredoxin-interacting protein (Thioredoxin-binding protein 2) (Vitamin D3 up-regulated protein 1) | PDTPEAP | 325 | 331 |
| **Q9H3P2** | NELFA WHSC2 P/OKcl.15 | Negative elongation factor A (NELF-A) (Wolf-Hirschhorn syndrome candidate 2 protein) | PATPTP | 383 | 388 |
| **Q9H3R2** | MUC13 DRCC1 RECC UNQ6194/PRO20221 | Mucin-13 (MUC-13) (Down-regulated in colon cancer 1) | PTPAPP | 83 | 88 |
| **Q9H3S7** | PTPN23 KIAA1471 | Tyrosine-protein phosphatase non-receptor type 23 (EC 3.1.3.48) (His domain-containing protein tyrosine phosphatase) (HD-PTP) (Protein tyrosine phosphatase TD14) (PTP-TD14) | PTPAPP | 901 | 906 |
| **Q9H3T3** | SEMA6B SEMAN SEMAZ UNQ1907/PRO4353 | Semaphorin-6B (Semaphorin-Z) (Sema Z) | PTPHP | 721 | 725 |
| **Q9H3U1** | UNC45A SMAP1 | Protein unc-45 homolog A (Unc-45A) (GCUNC-45) (Smooth muscle cell-associated protein 1) (SMAP-1) | PGTPEP | 6 | 11 |
| **Q9H4A3** | WNK1 HSN2 KDP KIAA0344 PRKWNK1 | Serine/threonine-protein kinase WNK1 (EC 2.7.11.1) (Erythrocyte 65 kDa protein) (p65) (Kinase deficient protein) (Protein kinase lysine-deficient 1) (Protein kinase with no lysine 1) (hWNK1) | PTPLLP | 832 | 837 |
| **Q9H4M7** | PLEKHA4 PEPP1 | Pleckstrin homology domain-containing family A member 4 (PH domain-containing family A member 4) (Phosphoinositol 3-phosphate-binding protein 1) (PEPP-1) | PTPWGP | 745 | 750 |
| **Q9H4Z2** | ZNF335 | Zinc finger protein 335 (NRC-interacting factor 1) (NIF-1) | PTPSTP | 340 | 345 |
| **Q9H5V7** | IKZF5 ZNFN1A5 | Zinc finger protein Pegasus (Ikaros family zinc finger protein 5) | PSTPAP | 348 | 353 |
| **Q9H665** | IGFLR1 TMEM149 U2AF1L4 | IGF-like family receptor 1 (Transmembrane protein 149) (U2 small nuclear RNA auxiliary factor 1-like 4) | PLTPGNP | 124 | 130 |
| **Q9H6K5** | PRR36 | Proline-rich protein 36 | PTPGTP | 167 | 172 |
| **Q9H6R4** | NOL6 | Nucleolar protein 6 (Nucleolar RNA-associated protein) (Nrap) | PPTPVRP | 700 | 706 |
| **Q9H6X2** | ANTXR1 ATR TEM8 | Anthrax toxin receptor 1 (Tumor endothelial marker 8) | PTPPIP | 529 | 534 |
| **Q9H792** | PEAK1 KIAA2002 | Pseudopodium-enriched atypical kinase 1 (EC 2.7.10.2) (Sugen kinase 269) (Tyrosine-protein kinase SgK269) | PVTPSP | 850 | 855 |
| **Q9H799** | C5orf42 | Uncharacterized protein C5orf42 | PSTPIQP | 2383 | 2389 |
| **Q9H7M9** | C10orf54 SISP1 VISTA PP2135 UNQ730/PRO1412 | V-type immunoglobulin domain-containing suppressor of T-cell activation (Platelet receptor Gi24) (Stress-induced secreted protein-1) (Sisp-1) (V-set domain-containing immunoregulatory receptor) | PSTPLSP | 282 | 288 |
| **Q9H7N4** | SCAF1 SFRS19 SRA1 | Splicing factor, arginine/serine-rich 19 (SR-related and CTD-associated factor 1) (SR-related-CTD-associated factor) (SCAF) (Serine arginine-rich pre-mRNA splicing factor SR-A1) (SR-A1) | PTPAP | 329 | 333 |
| **Q9H7P9** | PLEKHG2 | Pleckstrin homology domain-containing family G member 2 (PH domain-containing family G member 2) | PTTPALP | 1017 | 1023 |
| **Q9H7T3** | C10orf95 | Uncharacterized protein C10orf95 | PTPRP | 126 | 130 |
| **Q9H987** | SYNPO2L | Synaptopodin 2-like protein | PTPAP | 457 | 461 |
| **Q9H9B1** | EHMT1 EUHMTASE1 GLP KIAA1876 KMT1D | Histone-lysine N-methyltransferase EHMT1 (EC 2.1.1.-) (EC 2.1.1.43) (Euchromatic histone-lysine N-methyltransferase 1) (Eu-HMTase1) (G9a-like protein 1) (GLP) (GLP1) (Histone H3-K9 methyltransferase 5) (H3-K9-HMTase 5) (Lysine N-methyltransferase 1D) | PQTPAAP | 169 | 175 |
| **Q9HA65** | TBC1D17 | TBC1 domain family member 17 | PPTPPP | 618 | 623 |
| **Q9HAB3** | SLC52A2 GPR172A PAR1 RFT3 | Solute carrier family 52, riboflavin transporter, member 2 (Porcine endogenous retrovirus A receptor 1) (PERV-A receptor 1) (Protein GPR172A) (Riboflavin transporter 3) (hRFT3) | PTPARP | 4 | 9 |
| **Q9HAZ2** | PRDM16 KIAA1675 MEL1 PFM13 | PR domain zinc finger protein 16 (PR domain-containing protein 16) (Transcription factor MEL1) (MDS1/EVI1-like gene 1) | PLTPSP | 462 | 467 |
| **Q9HB19** | PLEKHA2 TAPP2 | Pleckstrin homology domain-containing family A member 2 (PH domain-containing family A member 2) (Tandem PH domain-containing protein 2) (TAPP-2) | PWTPVP | 356 | 361 |
| **Q9HB55** | CYP3A43 | Cytochrome P450 3A43 (EC 1.14.14.1) | PTPLP | 41 | 45 |
| **Q9HBD1** | RC3H2 MNAB RNF164 | Roquin-2 (EC 6.3.2.-) (Membrane-associated nucleic acid-binding protein) (RING finger and CCCH-type zinc finger domain-containing protein 2) (RING finger protein 164) | PTPPSP | 804 | 809 |
| **Q9HBI1** | PARVB CGI-56 | Beta-parvin (Affixin) | PTPRP | 8 | 12 |
| **Q9HBL0** | TNS1 TNS | Tensin-1 | PRTPTQP | 1103 | 1109 |
| **Q9HBX8** | LGR6 UNQ6427/PRO21331 VTS20631 | Leucine-rich repeat-containing G-protein coupled receptor 6 | PTPGP | 547 | 551 |
| **Q9HC35** | EML4 C2orf2 EMAPL4 | Echinoderm microtubule-associated protein-like 4 (EMAP-4) (Restrictedly overexpressed proliferation-associated protein) (Ropp 120) | PTPPP | 898 | 902 |
| **Q9HC73** | CRLF2 CRL2 ILXR TSLPR | Cytokine receptor-like factor 2 (Cytokine receptor-like 2) (IL-XR) (Thymic stromal lymphopoietin protein receptor) (TSLP receptor) | PTPPKP | 222 | 227 |
| **Q9HC84** | MUC5B MUC5 | Mucin-5B (MUC-5B) (Cervical mucin) (High molecular weight salivary mucin MG1) (Mucin-5 subtype B, tracheobronchial) (Sublingual gland mucin) | PTPTP | 5280 | 5284 |
| **Q9HCD5** | NCOA5 KIAA1637 | Nuclear receptor coactivator 5 (NCoA-5) (Coactivator independent of AF-2) (CIA) | PTPSAP | 419 | 424 |
| **Q9HCD6** | TANC2 KIAA1148 KIAA1636 | Protein TANC2 (Tetratricopeptide repeat, ankyrin repeat and coiled-coil domain-containing protein 2) | PPTPRP | 1766 | 1771 |
| **Q9HCE3** | ZNF532 KIAA1629 | Zinc finger protein 532 | PITPAMP | 735 | 741 |
| **Q9HCH0** | NCKAP5L CEP169 KIAA1602 FP1193 | Nck-associated protein 5-like (NCKAP5-like) (Centrosomal protein of 169 kDa) (Cep169) | PLTPLQP | 115 | 121 |
| **Q9HCJ0** | TNRC6C KIAA1582 | Trinucleotide repeat-containing gene 6C protein | PTPSP | 1028 | 1032 |
| **Q9HCJ6** | VAT1L KIAA1576 | Synaptic vesicle membrane protein VAT-1 homolog-like (EC 1.-.-.-) | PKTPLVP | 96 | 102 |
| **Q9HCM7** | FBRSL1 AUTS2L KIAA1545 XTP9 | Fibrosin-1-like protein (AUTS2-like protein) (HBV X-transactivated gene 9 protein) (HBV XAg-transactivated protein 9) | PPTPPGP | 1008 | 1014 |
| **Q9HCU4** | CELSR2 CDHF10 EGFL2 KIAA0279 MEGF3 | Cadherin EGF LAG seven-pass G-type receptor 2 (Cadherin family member 10) (Epidermal growth factor-like protein 2) (EGF-like protein 2) (Flamingo homolog 3) (Multiple epidermal growth factor-like domains protein 3) (Multiple EGF-like domains protein 3) | PTPPPP | 11 | 16 |
| **Q9HD36** | BCL2L10 BCLB | Bcl-2-like protein 10 (Bcl2-L-10) (Anti-apoptotic protein NrH) (Apoptosis regulator Bcl-B) | PGTPEP | 24 | 29 |
| **Q9NNX6** | CD209 CLEC4L | CD209 antigen (C-type lectin domain family 4 member L) (Dendritic cell-specific ICAM-3-grabbing non-integrin 1) (DC-SIGN) (DC-SIGN1) (CD antigen CD209) | PATPNPP | 396 | 402 |
| **Q9NP71** | MLXIPL BHLHD14 MIO WBSCR14 | Carbohydrate-responsive element-binding protein (ChREBP) (Class D basic helix-loop-helix protein 14) (bHLHd14) (MLX interactor) (MLX-interacting protein-like) (WS basic-helix-loop-helix leucine zipper protein) (WS-bHLH) (Williams-Beuren syndrome chromosomal region 14 protein) | PTPFP | 467 | 471 |
| **Q9NP74** | PALMD C1orf11 PALML | Palmdelphin (Paralemmin-like protein) | PTPLP | 478 | 482 |
| **Q9NPC1** | LTB4R2 BLT2R BLTR2 | Leukotriene B4 receptor 2 (LTB4-R 2) (LTB4-R2) (LTB4 receptor JULF2) (Leukotriene B4 receptor BLT2) (Seven transmembrane receptor BLTR2) | PTPERP | 14 | 19 |
| **Q9NPC6** | MYOZ2 C4orf5 | Myozenin-2 (Calsarcin-1) (FATZ-related protein 2) | PNTPDP | 109 | 114 |
| **Q9NPR2** | SEMA4B KIAA1745 SEMAC UNQ749/PRO1480 | Semaphorin-4B | PTPRP | 387 | 391 |
| **Q9NQ03** | SCRT2 FP7030 | Transcriptional repressor scratch 2 (Scratch homolog 2 zinc finger protein) | PPTPAGP | 299 | 305 |
| **Q9NQ75** | CASS4 C20orf32 HEFL | Cas scaffolding protein family member 4 (HEF-like protein) (HEF1-EFS-p130Cas-like protein) (HEPL) | PPTPGP | 106 | 111 |
| **Q9NQ87** | HEYL BHLHB33 HRT3 | Hairy/enhancer-of-split related with YRPW motif-like protein (hHeyL) (Class B basic helix-loop-helix protein 33) (bHLHb33) (Hairy-related transcription factor 3) (HRT-3) (hHRT3) | PTPTGP | 164 | 169 |
| **Q9NQC3** | RTN4 KIAA0886 NOGO My043 SP1507 | Reticulon-4 (Foocen) (Neurite outgrowth inhibitor) (Nogo protein) (Neuroendocrine-specific protein) (NSP) (Neuroendocrine-specific protein C homolog) (RTN-x) (Reticulon-5) | PSTPAAP | 170 | 176 |
| **Q9NQS3** | NECTIN3 PRR3 PVRL3 | Nectin-3 (CDw113) (Nectin cell adhesion molecule 3) (Poliovirus receptor-related protein 3) (CD antigen CD113) | PPTPPP | 33 | 38 |
| **Q9NQS7** | INCENP | Inner centromere protein | PTPESP | 144 | 149 |
| **Q9NQW1** | SEC31B SEC31L2 | Protein transport protein Sec31B (SEC31-like protein 2) (SEC31-related protein B) (SEC31B-1) | PTPSP | 826 | 830 |
| **Q9NQW6** | ANLN | Actin-binding protein anillin | PKTPISP | 318 | 324 |
| **Q9NQX4** | MYO5C | Unconventional myosin-Vc | PTPPSP | 594 | 599 |
| **Q9NQX5** | NPDC1 | Neural proliferation differentiation and control protein 1 (NPDC-1) | PSTPGTP | 145 | 151 |
| **Q9NR12** | PDLIM7 ENIGMA | PDZ and LIM domain protein 7 (LIM mineralization protein) (LMP) (Protein enigma) | PATPTP | 249 | 254 |
| **Q9NR71** | ASAH2 HNAC1 | Neutral ceramidase (N-CDase) (NCDase) (EC 3.5.1.23) (Acylsphingosine deacylase 2) (BCDase) (LCDase) (hCD) (N-acylsphingosine amidohydrolase 2) (Non-lysosomal ceramidase) [Cleaved into: Neutral ceramidase soluble form] | PLTPESP | 88 | 94 |
| **Q9NR83** | SLC2A4RG HDBP1 | SLC2A4 regulator (GLUT4 enhancer factor) (GEF) (Huntington disease gene regulatory region-binding protein 1) (HDBP-1) | PSTPSPP | 168 | 174 |
| **Q9NRB3** | CHST12 UNQ500/PRO1017 | Carbohydrate sulfotransferase 12 (EC 2.8.2.5) (Chondroitin 4-O-sulfotransferase 2) (Chondroitin 4-sulfotransferase 2) (C4ST-2) (C4ST2) (Sulfotransferase Hlo) | PTPGP | 50 | 54 |
| **Q9NRL3** | STRN4 ZIN | Striatin-4 (Zinedin) | PGTPQP | 375 | 380 |
| **Q9NRN7** | AASDHPPT CGI-80 HAH-P HSPC223 x0005 | L-aminoadipate-semialdehyde dehydrogenase-phosphopantetheinyl transferase (EC 2.7.8.-) (4'-phosphopantetheinyl transferase) (Alpha-aminoadipic semialdehyde dehydrogenase-phosphopantetheinyl transferase) (AASD-PPT) (LYS5 ortholog) | PMTPEDP | 283 | 289 |
| **Q9NRR5** | UBQLN4 C1orf6 CIP75 UBIN | Ubiquilin-4 (Ataxin-1 interacting ubiquitin-like protein) (A1Up) (Ataxin-1 ubiquitin-like-interacting protein A1U) (Connexin43-interacting protein of 75 kDa) (CIP75) | PSTPDP | 99 | 104 |
| **Q9NRS6** | SNX15 | Sorting nexin-15 | PTPPP | 147 | 151 |
| **Q9NRY4** | ARHGAP35 GRF1 GRLF1 KIAA1722 | Rho GTPase-activating protein 35 (Glucocorticoid receptor DNA-binding factor 1) (Glucocorticoid receptor repression factor 1) (GRF-1) (Rho GAP p190A) (p190-A) | PPTPQSP | 1478 | 1484 |
| **Q9NRY6** | PLSCR3 | Phospholipid scramblase 3 (PL scramblase 3) (Ca(2+)-dependent phospholipid scramblase 3) | PVTPGYP | 19 | 25 |
| **Q9NRZ5** | AGPAT4 UNQ499/PRO1016 | 1-acyl-sn-glycerol-3-phosphate acyltransferase delta (EC 2.3.1.51) (1-acylglycerol-3-phosphate O-acyltransferase 4) (1-AGP acyltransferase 4) (1-AGPAT 4) (Lysophosphatidic acid acyltransferase delta) (LPAAT-delta) | PETPMVP | 295 | 301 |
| **Q9NS39** | ADARB2 ADAR3 RED2 | Double-stranded RNA-specific editase B2 (EC 3.5.-.-) (RNA-dependent adenosine deaminase 3) (RNA-editing deaminase 2) (RNA-editing enzyme 2) (dsRNA adenosine deaminase B2) | PTPATP | 266 | 271 |
| **Q9NSC2** | SALL1 SAL1 ZNF794 | Sal-like protein 1 (Spalt-like transcription factor 1) (Zinc finger protein 794) (Zinc finger protein SALL1) (Zinc finger protein Spalt-1) (HSal1) (Sal-1) | PNTPVP | 794 | 799 |
| **Q9NSE2** | CISH G18 | Cytokine-inducible SH2-containing protein (CIS) (CIS-1) (Protein G18) (Suppressor of cytokine signaling) (SOCS) | PTPALP | 180 | 185 |
| **Q9NSU2** | TREX1 | Three-prime repair exonuclease 1 (EC 3.1.11.2) (3'-5' exonuclease TREX1) (DNase III) | PPTPLPP | 23 | 29 |
| **Q9NSY0** | NRBP2 PP9320 TRG16 | Nuclear receptor-binding protein 2 (Transformation-related gene 16 protein) (TRG-16) | PTPEP | 410 | 414 |
| **Q9NTN9** | SEMA4G KIAA1619 | Semaphorin-4G | PATPAP | 652 | 657 |
| **Q9NTX7** | RNF146 | E3 ubiquitin-protein ligase RNF146 (EC 6.3.2.-) (Dactylidin) (Iduna) (RING finger protein 146) | PATPSP | 232 | 237 |
| **Q9NTZ6** | RBM12 KIAA0765 HRIHFB2091 | RNA-binding protein 12 (RNA-binding motif protein 12) (SH3/WW domain anchor protein in the nucleus) (SWAN) | PMTPLPP | 234 | 240 |
| **Q9NUE0** | ZDHHC18 | Palmitoyltransferase ZDHHC18 (EC 2.3.1.225) (Zinc finger DHHC domain-containing protein 18) (DHHC-18) | PTPGP | 31 | 35 |
| **Q9NUK0** | MBNL3 CHCR MBLX39 MBXL | Muscleblind-like protein 3 (Cys3His CCG1-required protein) (Muscleblind-like X-linked protein) (Protein HCHCR) | PMTPSIP | 118 | 124 |
| **Q9NVD7** | PARVA MXRA2 | Alpha-parvin (Actopaxin) (CH-ILKBP) (Calponin-like integrin-linked kinase-binding protein) (Matrix-remodeling-associated protein 2) | PTPKSP | 15 | 20 |
| **Q9NW97** | TMEM51 C1orf72 | Transmembrane protein 51 | PLTPPP | 232 | 237 |
| **Q9NWM3** | CUEDC1 | CUE domain-containing protein 1 | PPTPPP | 149 | 154 |
| **Q9NWS9** | ZNF446 ZKSCAN20 | Zinc finger protein 446 (Zinc finger protein with KRAB and SCAN domains 20) | PGTPPVP | 306 | 312 |
| **Q9NX09** | DDIT4 REDD1 RTP801 | DNA damage-inducible transcript 4 protein (HIF-1 responsive protein RTP801) (Protein regulated in development and DNA damage response 1) (REDD-1) | PRTPTP | 21 | 26 |
| **Q9NX94** | WBP1L C10orf26 OPA1L | WW domain binding protein 1-like (Outcome predictor in acute leukemia 1) | PPTPPPP | 115 | 121 |
| **Q9NXE4** | SMPD4 KIAA1418 | Sphingomyelin phosphodiesterase 4 (EC 3.1.4.12) (Neutral sphingomyelinase 3) (nSMase-3) (nSMase3) (Neutral sphingomyelinase III) | PRTPAIP | 219 | 225 |
| **Q9NXR1** | NDE1 NUDE | Nuclear distribution protein nudE homolog 1 (NudE) | PRTPMP | 189 | 194 |
| **Q9NY27** | PPP4R2 SBBI57 | Serine/threonine-protein phosphatase 4 regulatory subunit 2 | PGTPRP | 171 | 176 |
| **Q9NY43** | BARHL2 | BarH-like 2 homeobox protein | PGTPHP | 381 | 386 |
| **Q9NYA3** | GOLGA6A GLP GOLGA6 | Golgin subfamily A member 6A (Golgin linked to PML) (Golgin-like protein) | PTPNIP | 517 | 522 |
| **Q9NYB9** | ABI2 ARGBPIA | Abl interactor 2 (Abelson interactor 2) (Abi-2) (Abl-binding protein 3) (AblBP3) (Arg-binding protein 1) (ArgBP1) | PTPSPP | 274 | 279 |
| **Q9NYQ6** | CELSR1 CDHF9 FMI2 | Cadherin EGF LAG seven-pass G-type receptor 1 (Cadherin family member 9) (Flamingo homolog 2) (hFmi2) | PTPDFP | 624 | 629 |
| **Q9NYV4** | CDK12 CRK7 CRKRS KIAA0904 | Cyclin-dependent kinase 12 (EC 2.7.11.22) (EC 2.7.11.23) (Cdc2-related kinase, arginine/serine-rich) (CrkRS) (Cell division cycle 2-related protein kinase 7) (CDC2-related protein kinase 7) (Cell division protein kinase 12) (hCDK12) | PQTPPLP | 546 | 552 |
| **Q9NZJ4** | SACS KIAA0730 | Sacsin (DnaJ homolog subfamily C member 29) (DNAJC29) | PNTPVP | 1375 | 1380 |
| **Q9NZK5** | CECR1 ADA2 ADGF IDGFL | Adenosine deaminase CECR1 (EC 3.5.4.4) (Cat eye syndrome critical region protein 1) | PTPRP | 151 | 155 |
| **Q9NZM3** | ITSN2 KIAA1256 SH3D1B SWAP | Intersectin-2 (SH3 domain-containing protein 1B) (SH3P18) (SH3P18-like WASP-associated protein) | PTPLVP | 155 | 160 |
| **Q9NZM4** | GLTSCR1 | Glioma tumor suppressor candidate region gene 1 protein | PTPIQP | 348 | 353 |
| **Q9NZP6** | NPAP1 C15orf2 | Nuclear pore-associated protein 1 | PTPRP | 42 | 46 |
| **Q9NZQ3** | NCKIPSD AF3P21 SPIN90 | NCK-interacting protein with SH3 domain (54 kDa VacA-interacting protein) (54 kDa vimentin-interacting protein) (VIP54) (90 kDa SH3 protein interacting with Nck) (AF3p21) (Dia-interacting protein 1) (DIP-1) (Diaphanous protein-interacting protein) (SH3 adapter protein SPIN90) (WASP-interacting SH3-domain protein) (WISH) (Wiskott-Aldrich syndrome protein-interacting protein) | PTTPPPP | 179 | 185 |
| **Q9P107** | GMIP | GEM-interacting protein (GMIP) | PGTPSP | 658 | 663 |
| **Q9P1W9** | PIM2 | Serine/threonine-protein kinase pim-2 (EC 2.7.11.1) (Pim-2h) | PGTPTPP | 13 | 19 |
| **Q9P1Y5** | CAMSAP3 KIAA1543 | Calmodulin-regulated spectrin-associated protein 3 (Protein Nezha) | PETPSKP | 523 | 529 |
| **Q9P1Y6** | PHRF1 KIAA1542 | PHD and RING finger domain-containing protein 1 | PLTPRTP | 328 | 334 |
| **Q9P1Z0** | ZBTB4 KIAA1538 | Zinc finger and BTB domain-containing protein 4 (KAISO-like zinc finger protein 1) (KAISO-L1) | PPTPAP | 187 | 192 |
| **Q9P206** | KIAA1522 | Uncharacterized protein KIAA1522 | PGTPRAP | 183 | 189 |
| **Q9P218** | COL20A1 KIAA1510 | Collagen alpha-1(XX) chain | PRTPAGP | 162 | 168 |
| **Q9P281** | BAHCC1 BAHD2 KIAA1447 | BAH and coiled-coil domain-containing protein 1 (Bromo adjacent homology domain-containing protein 2) (BAH domain-containing protein 2) | PTPPP | 1006 | 1010 |
| **Q9P283** | SEMA5B KIAA1445 SEMAG UNQ5867/PRO34001 | Semaphorin-5B | PVTPEP | 457 | 462 |
| **Q9P2D1** | CHD7 KIAA1416 | Chromodomain-helicase-DNA-binding protein 7 (CHD-7) (EC 3.6.4.12) (ATP-dependent helicase CHD7) | PKTPKAP | 668 | 674 |
| **Q9P2E9** | RRBP1 KIAA1398 | Ribosome-binding protein 1 (180 kDa ribosome receptor homolog) (RRp) (ES/130-related protein) (Ribosome receptor protein) | PTPVQP | 109 | 114 |
| **Q9P2G1** | ANKIB1 KIAA1386 | Ankyrin repeat and IBR domain-containing protein 1 | PTPPP | 289 | 293 |
| **Q9P2G4** | MAP10 KIAA1383 MTR120 | Microtubule-associated protein 10 (Microtubule regulator of 120 KDa) | PTPTP | 125 | 129 |
| **Q9P2H0** | CEP126 KIAA1377 | Centrosomal protein of 126 kDa | PVTPENP | 823 | 829 |
| **Q9P2J2** | IGSF9 IGSF9A KIAA1355 NRT1 | Protein turtle homolog A (Immunoglobulin superfamily member 9A) (IgSF9A) | PETPLP | 330 | 335 |
| **Q9P2K1** | CC2D2A KIAA1345 | Coiled-coil and C2 domain-containing protein 2A | PTPRP | 124 | 128 |
| **Q9P2K3** | RCOR3 KIAA1343 | REST corepressor 3 | PTPTAP | 402 | 407 |
| **Q9P2K8** | EIF2AK4 GCN2 KIAA1338 | eIF-2-alpha kinase GCN2 (EC 2.7.11.1) (Eukaryotic translation initiation factor 2-alpha kinase 4) (GCN2-like protein) | PGTPPP | 665 | 670 |
| **Q9P2N5** | RBM27 KIAA1311 | RNA-binding protein 27 (RNA-binding motif protein 27) | PTPLVP | 476 | 481 |
| **Q9P2P1** | NYNRIN CGIN1 KIAA1305 | Protein NYNRIN (NYN domain and retroviral integrase catalytic domain-containing protein) (Protein cousin of GIN1) | PTTPKTP | 619 | 625 |
| **Q9P2P6** | STARD9 KIAA1300 | StAR-related lipid transfer protein 9 (START domain-containing protein 9) (StARD9) | PATPPYP | 3402 | 3408 |
| **Q9P2R6** | RERE ARG ARP ATN1L KIAA0458 | Arginine-glutamic acid dipeptide repeats protein (Atrophin-1-like protein) (Atrophin-1-related protein) | PGTPQLP | 756 | 762 |
| **Q9P2S2** | NRXN2 KIAA0921 | Neurexin-2 (Neurexin II-alpha) (Neurexin-2-alpha) | PTPPP | 9 | 13 |
| **Q9P2Y4** | ZNF219 | Zinc finger protein 219 | PTPAP | 258 | 262 |
| **Q9UBB6** | NCDN KIAA0607 | Neurochondrin | PTTPGP | 449 | 454 |
| **Q9UBF2** | COPG2 | Coatomer subunit gamma-2 (Gamma-2-coat protein) (Gamma-2-COP) | PRTPVP | 464 | 469 |
| **Q9UBG0** | MRC2 CLEC13E ENDO180 KIAA0709 UPARAP | C-type mannose receptor 2 (C-type lectin domain family 13 member E) (Endocytic receptor 180) (Macrophage mannose receptor 2) (Urokinase-type plasminogen activator receptor-associated protein) (UPAR-associated protein) (Urokinase receptor-associated protein) (CD antigen CD280) | PVTPELP | 651 | 657 |
| **Q9UBG7** | RBPJL RBPL RBPSUHL | Recombining binding protein suppressor of hairless-like protein (Transcription factor RBP-L) | PVTPVP | 381 | 386 |
| **Q9UBK2** | PPARGC1A LEM6 PGC1 PGC1A PPARGC1 | Peroxisome proliferator-activated receptor gamma coactivator 1-alpha (PGC-1-alpha) (PPAR-gamma coactivator 1-alpha) (PPARGC-1-alpha) (Ligand effect modulator 6) | PLTPESP | 261 | 267 |
| **Q9UBS5** | GABBR1 GPRC3A | Gamma-aminobutyric acid type B receptor subunit 1 (GABA-B receptor 1) (GABA-B-R1) (GABA-BR1) (GABABR1) (Gb1) | PPTPPEP | 928 | 934 |
| **Q9UBS9** | SUCO C1orf9 CH1 OPT SLP1 | SUN domain-containing ossification factor (Membrane protein CH1) (Protein osteopotentia homolog) (SUN-like protein 1) | PSTPDTP | 566 | 572 |
| **Q9UBY0** | SLC9A2 NHE2 | Sodium/hydrogen exchanger 2 (Na(+)/H(+) exchanger 2) (NHE-2) (Solute carrier family 9 member 2) | PSTPPTP | 744 | 750 |
| **Q9UDY2** | TJP2 X104 ZO2 | Tight junction protein ZO-2 (Tight junction protein 2) (Zona occludens protein 2) (Zonula occludens protein 2) | PSTPIPP | 1052 | 1058 |
| **Q9UF56** | FBXL17 FBL17 FBX13 FBXO13 | F-box/LRR-repeat protein 17 (F-box and leucine-rich repeat protein 17) (F-box only protein 13) | PTPSP | 182 | 186 |
| **Q9UGL9** | CRCT1 C1orf42 NICE1 | Cysteine-rich C-terminal protein 1 (Protein NICE-1) | PTPAP | 29 | 33 |
| **Q9UGM3** | DMBT1 GP340 | Deleted in malignant brain tumors 1 protein (Glycoprotein 340) (Gp-340) (Hensin) (Salivary agglutinin) (SAG) (Surfactant pulmonary-associated D-binding protein) | PTPSP | 340 | 344 |
| **Q9UGR2** | ZC3H7B KIAA1031 | Zinc finger CCCH domain-containing protein 7B (Rotavirus 'X'-associated non-structural protein) (RoXaN) | PSTPTMP | 238 | 244 |
| **Q9UH03** | SEPT3 SEP3 | Neuronal-specific septin-3 | PATPCP | 350 | 355 |
| **Q9UHB4** | NDOR1 NR1 | NADPH-dependent diflavin oxidoreductase 1 (EC 1.18.1.-) (NADPH-dependent FMN and FAD-containing oxidoreductase) (Novel reductase 1) | PETPDTP | 446 | 452 |
| **Q9UHC7** | MKRN1 RNF61 | E3 ubiquitin-protein ligase makorin-1 (EC 6.3.2.-) (RING finger protein 61) | PTPIP | 28 | 32 |
| **Q9UHD9** | UBQLN2 N4BP4 PLIC2 HRIHFB2157 | Ubiquilin-2 (Chap1) (DSK2 homolog) (Protein linking IAP with cytoskeleton 2) (PLIC-2) (hPLIC-2) (Ubiquitin-like product Chap1/Dsk2) | PVTPIGP | 494 | 500 |
| **Q9UHI6** | DDX20 DP103 GEMIN3 | Probable ATP-dependent RNA helicase DDX20 (EC 3.6.4.13) (Component of gems 3) (DEAD box protein 20) (DEAD box protein DP 103) (Gemin-3) | PTPGP | 32 | 36 |
| **Q9UHI7** | SLC23A1 SVCT1 YSPL3 | Solute carrier family 23 member 1 (Na(+)/L-ascorbic acid transporter 1) (Sodium-dependent vitamin C transporter 1) (hSVCT1) (Yolk sac permease-like molecule 3) | PSTPLP | 19 | 24 |
| **Q9UHR4** | BAIAP2L1 IRTKS | Brain-specific angiogenesis inhibitor 1-associated protein 2-like protein 1 (BAI1-associated protein 2-like protein 1) (Insulin receptor tyrosine kinase substrate) | PTPSP | 411 | 415 |
| **Q9UHV7** | MED13 ARC250 KIAA0593 THRAP1 TRAP240 | Mediator of RNA polymerase II transcription subunit 13 (Activator-recruited cofactor 250 kDa component) (ARC250) (Mediator complex subunit 13) (Thyroid hormone receptor-associated protein 1) (Thyroid hormone receptor-associated protein complex 240 kDa component) (Trap240) (Vitamin D3 receptor-interacting protein complex component DRIP250) (DRIP250) | PTPSP | 291 | 295 |
| **Q9UHY1** | NRBP1 BCON3 NRBP | Nuclear receptor-binding protein | PTPEP | 432 | 436 |
| **Q9UIF8** | BAZ2B KIAA1476 | Bromodomain adjacent to zinc finger domain protein 2B (hWALp4) | PTPAP | 1597 | 1601 |
| **Q9UIF9** | BAZ2A KIAA0314 TIP5 | Bromodomain adjacent to zinc finger domain protein 2A (Transcription termination factor I-interacting protein 5) (TTF-I-interacting protein 5) (Tip5) (hWALp3) | PTPPP | 1336 | 1340 |
| **Q9UIH9** | KLF15 KKLF | Krueppel-like factor 15 (Kidney-enriched krueppel-like factor) | PTPDGP | 211 | 216 |
| **Q9UJQ4** | SALL4 ZNF797 | Sal-like protein 4 (Zinc finger protein 797) (Zinc finger protein SALL4) | PNTPLP | 654 | 659 |
| **Q9UK55** | SERPINA10 ZPI UNQ707/PRO1358 | Protein Z-dependent protease inhibitor (PZ-dependent protease inhibitor) (PZI) (Serpin A10) | PETPAP | 29 | 34 |
| **Q9UKA4** | AKAP11 AKAP220 KIAA0629 | A-kinase anchor protein 11 (AKAP-11) (A-kinase anchor protein 220 kDa) (AKAP 220) (hAKAP220) (Protein kinase A-anchoring protein 11) (PRKA11) | PTPRKP | 415 | 420 |
| **Q9UKN7** | MYO15A MYO15 | Unconventional myosin-XV (Unconventional myosin-15) | PTPEKP | 1007 | 1012 |
| **Q9UKP4** | ADAMTS7 | A disintegrin and metalloproteinase with thrombospondin motifs 7 (ADAM-TS 7) (ADAM-TS7) (ADAMTS-7) (EC 3.4.24.-) (COMPase) | PGTPSFP | 1312 | 1318 |
| **Q9ULC8** | ZDHHC8 KIAA1292 ZDHHCL1 ZNF378 | Probable palmitoyltransferase ZDHHC8 (EC 2.3.1.225) (Zinc finger DHHC domain-containing protein 8) (DHHC-8) (Zinc finger protein 378) | PGTPHSP | 677 | 683 |
| **Q9ULD8** | KCNH3 KIAA1282 | Potassium voltage-gated channel subfamily H member 3 (Brain-specific eag-like channel 1) (BEC1) (Ether-a-go-go-like potassium channel 2) (ELK channel 2) (ELK2) (Voltage-gated potassium channel subunit Kv12.2) | PSTPASP | 1012 | 1018 |
| **Q9ULD9** | ZNF608 KIAA1281 | Zinc finger protein 608 (Renal carcinoma antigen NY-REN-36) | PSTPEP | 1157 | 1162 |
| **Q9ULE3** | DENND2A KIAA1277 | DENN domain-containing protein 2A | PTPAP | 60 | 64 |
| **Q9ULH1** | ASAP1 DDEF1 KIAA1249 | Arf-GAP with SH3 domain, ANK repeat and PH domain-containing protein 1 (130 kDa phosphatidylinositol 4,5-bisphosphate-dependent ARF1 GTPase-activating protein) (ADP-ribosylation factor-directed GTPase-activating protein 1) (ARF GTPase-activating protein 1) (Development and differentiation-enhancing factor 1) (DEF-1) (Differentiation-enhancing factor 1) (PIP2-dependent ARF1 GAP) | PETPVP | 1052 | 1057 |
| **Q9ULH7** | MKL2 KIAA1243 MRTFB | MKL/myocardin-like protein 2 (Megakaryoblastic leukemia 2) (Myocardin-related transcription factor B) (MRTF-B) | PNTPNKP | 839 | 845 |
| **Q9ULI3** | HEG1 KIAA1237 | Protein HEG homolog 1 | PATPGP | 75 | 80 |
| **Q9ULI4** | KIF26A KIAA1236 | Kinesin-like protein KIF26A | PTPQP | 1127 | 1131 |
| **Q9ULL0** | KIAA1210 | Uncharacterized protein KIAA1210 | PCTPRFP | 85 | 91 |
| **Q9ULL1** | PLEKHG1 KIAA1209 | Pleckstrin homology domain-containing family G member 1 | PRTPKKP | 1327 | 1333 |
| **Q9ULL5** | PRR12 KIAA1205 | Proline-rich protein 12 | PATPAVP | 506 | 512 |
| **Q9ULM3** | YEATS2 KIAA1197 | YEATS domain-containing protein 2 | PTPNP | 1020 | 1024 |
| **Q9ULT6** | ZNRF3 KIAA1133 RNF203 | E3 ubiquitin-protein ligase ZNRF3 (EC 6.3.2.-) (RING finger protein 203) (Zinc/RING finger protein 3) | PDTPRP | 861 | 866 |
| **Q9ULU8** | CADPS CAPS CAPS1 KIAA1121 | Calcium-dependent secretion activator 1 (Calcium-dependent activator protein for secretion 1) (CAPS-1) | PTPNSP | 484 | 489 |
| **Q9ULV3** | CIZ1 LSFR1 NP94 ZNF356 | Cip1-interacting zinc finger protein (CDKN1A-interacting zinc finger protein 1) (Nuclear protein NP94) (Zinc finger protein 356) | PLTPVP | 565 | 570 |
| **Q9UM47** | NOTCH3 | Neurogenic locus notch homolog protein 3 (Notch 3) [Cleaved into: Notch 3 extracellular truncation; Notch 3 intracellular domain] | PCTPNP | 774 | 779 |
| **Q9UM73** | ALK | ALK tyrosine kinase receptor (EC 2.7.10.1) (Anaplastic lymphoma kinase) (CD antigen CD246) | PTPEP | 1025 | 1029 |
| **Q9UMD9** | COL17A1 BP180 BPAG2 | Collagen alpha-1(XVII) chain (180 kDa bullous pemphigoid antigen 2) (Bullous pemphigoid antigen 2) [Cleaved into: 120 kDa linear IgA disease antigen (120 kDa linear IgA dermatosis antigen) (Linear IgA disease antigen 1) (LAD-1); 97 kDa linear IgA disease antigen (97 kDa linear IgA bullous dermatosis antigen) (97 kDa LAD antigen) (97-LAD) (Linear IgA bullous disease antigen of 97 kDa) (LABD97)] | PGTPGIP | 587 | 593 |
| **Q9UMN6** | KMT2B HRX2 KIAA0304 MLL2 MLL4 TRX2 WBP7 | Histone-lysine N-methyltransferase 2B (Lysine N-methyltransferase 2B) (EC 2.1.1.43) (Myeloid/lymphoid or mixed-lineage leukemia protein 4) (Trithorax homolog 2) (WW domain-binding protein 7) (WBP-7) | PLTPPAP | 405 | 411 |
| **Q9UMR3** | TBX20 | T-box transcription factor TBX20 (T-box protein 20) | PTTPIIP | 87 | 93 |
| **Q9UMS6** | SYNPO2 | Synaptopodin-2 (Genethonin-2) (Myopodin) | PATPFSP | 599 | 605 |
| **Q9UNU6** | CYP8B1 CYP12 | 7-alpha-hydroxycholest-4-en-3-one 12-alpha-hydroxylase (EC 1.14.18.8) (7-alpha-hydroxy-4-cholesten-3-one 12-alpha-hydroxylase) (CYPVIIIB1) (Cytochrome P450 8B1) (Sterol 12-alpha-hydroxylase) | PDTPLP | 468 | 473 |
| **Q9UNX4** | WDR3 | WD repeat-containing protein 3 | PTPQP | 399 | 403 |
| **Q9UP52** | TFR2 | Transferrin receptor protein 2 (TfR2) | PYTPGFP | 330 | 336 |
| **Q9UPA5** | BSN KIAA0434 ZNF231 | Protein bassoon (Zinc finger protein 231) | PTPLPP | 528 | 533 |
| **Q9UPM6** | LHX6 LHX6.1 | LIM/homeobox protein Lhx6 (LIM homeobox protein 6) (LIM/homeobox protein Lhx6.1) | PCTPSTP | 44 | 50 |
| **Q9UPN3** | MACF1 ABP620 ACF7 KIAA0465 KIAA1251 | Microtubule-actin cross-linking factor 1, isoforms 1/2/3/5 (620 kDa actin-binding protein) (ABP620) (Actin cross-linking family protein 7) (Macrophin-1) (Trabeculin-alpha) | PTPPP | 6971 | 6975 |
| **Q9UPN6** | SCAF8 CCAP7 KIAA1116 RBM16 | Protein SCAF8 (CDC5L complex-associated protein 7) (RNA-binding motif protein 16) (SR-related and CTD-associated factor 8) | PGTPVTP | 158 | 164 |
| **Q9UPQ7** | PDZRN3 KIAA1095 LNX3 SEMCAP3 | E3 ubiquitin-protein ligase PDZRN3 (EC 6.3.2.-) (Ligand of Numb protein X 3) (PDZ domain-containing RING finger protein 3) (Semaphorin cytoplasmic domain-associated protein 3) (Protein SEMACAP3) | PTPSEP | 912 | 917 |
| **Q9UPR5** | SLC8A2 KIAA1087 NCX2 | Sodium/calcium exchanger 2 (Na(+)/Ca(2+)-exchange protein 2) (Solute carrier family 8 member 2) | PTPSLP | 24 | 29 |
| **Q9UPS6** | SETD1B KIAA1076 KMT2G SET1B | Histone-lysine N-methyltransferase SETD1B (EC 2.1.1.43) (Lysine N-methyltransferase 2G) (SET domain-containing protein 1B) (hSET1B) | PGTPPGP | 448 | 454 |
| **Q9UPT8** | ZC3H4 C19orf7 KIAA1064 | Zinc finger CCCH domain-containing protein 4 | PGTPPPP | 5 | 11 |
| **Q9UPV0** | CEP164 KIAA1052 NPHP15 | Centrosomal protein of 164 kDa (Cep164) | PPTPCKP | 278 | 284 |
| **Q9UPV9** | TRAK1 KIAA1042 OIP106 | Trafficking kinesin-binding protein 1 (106 kDa O-GlcNAc transferase-interacting protein) | PGTPGTP | 484 | 490 |
| **Q9UPX0** | IGSF9B KIAA1030 | Protein turtle homolog B (Immunoglobulin superfamily member 9B) (IgSF9B) | PETPPP | 1317 | 1322 |
| **Q9UPX8** | SHANK2 CORTBP1 KIAA1022 PROSAP1 | SH3 and multiple ankyrin repeat domains protein 2 (Shank2) (Cortactin-binding protein 1) (CortBP1) (Proline-rich synapse-associated protein 1) | PTPAFP | 276 | 281 |
| **Q9UQ35** | SRRM2 KIAA0324 SRL300 SRM300 HSPC075 | Serine/arginine repetitive matrix protein 2 (300 kDa nuclear matrix antigen) (Serine/arginine-rich splicing factor-related nuclear matrix protein of 300 kDa) (SR-related nuclear matrix protein of 300 kDa) (Ser/Arg-related nuclear matrix protein of 300 kDa) (Splicing coactivator subunit SRm300) (Tax-responsive enhancer element-binding protein 803) (TaxREB803) | PQTPRP | 1490 | 1495 |
| **Q9UQQ1** | NAALADL1 NAALADASEL NAALADL | N-acetylated-alpha-linked acidic dipeptidase-like protein (NAALADase L) (EC 3.4.17.21) (100 kDa ileum brush border membrane protein) (I100) (Ileal dipeptidylpeptidase) | PLTPYLP | 256 | 262 |
| **Q9Y219** | JAG2 | Protein jagged-2 (Jagged2) (hJ2) | PSTPCLP | 955 | 961 |
| **Q9Y278** | HS3ST2 3OST2 UNQ2442/PRO5004 | Heparan sulfate glucosamine 3-O-sulfotransferase 2 (EC 2.8.2.29) (Heparan sulfate D-glucosaminyl 3-O-sulfotransferase 2) (3-OST-2) (Heparan sulfate 3-O-sulfotransferase 2) (h3-OST-2) | PTPSEP | 78 | 83 |
| **Q9Y2B5** | VPS9D1 ATPBL C16orf7 | VPS9 domain-containing protein 1 (Protein ATP-BL) | PPTPNP | 315 | 320 |
| **Q9Y2D9** | ZNF652 KIAA0924 | Zinc finger protein 652 | PATPVP | 512 | 517 |
| **Q9Y2E8** | SLC9A8 KIAA0939 NHE8 | Sodium/hydrogen exchanger 8 (Na(+)/H(+) exchanger 8) (NHE-8) (Solute carrier family 9 member 8) | PTPGKP | 36 | 41 |
| **Q9Y2F5** | ICE1 KIAA0947 | Little elongation complex subunit 1 (Interactor of little elongator complex ELL subunit 1) | PTPKP | 831 | 835 |
| **Q9Y2H5** | PLEKHA6 KIAA0969 PEPP3 | Pleckstrin homology domain-containing family A member 6 (PH domain-containing family A member 6) (Phosphoinositol 3-phosphate-binding protein 3) (PEPP-3) | PDTPLSP | 935 | 941 |
| **Q9Y2I8** | WDR37 KIAA0982 | WD repeat-containing protein 37 | PTPQP | 233 | 237 |
| **Q9Y2K7** | KDM2A CXXC8 FBL7 FBXL11 JHDM1A KIAA1004 | Lysine-specific demethylase 2A (EC 1.14.11.27) (CXXC-type zinc finger protein 8) (F-box and leucine-rich repeat protein 11) (F-box protein FBL7) (F-box protein Lilina) (F-box/LRR-repeat protein 11) (JmjC domain-containing histone demethylation protein 1A) ([Histone-H3]-lysine-36 demethylase 1A) | PLTPPP | 711 | 716 |
| **Q9Y2W3** | SLC45A1 DNB5 | Proton-associated sugar transporter A (PAST-A) (Deleted in neuroblastoma 5 protein) (DNb-5) (Solute carrier family 45 member 1) | PNTPCP | 64 | 69 |
| **Q9Y2X7** | GIT1 | ARF GTPase-activating protein GIT1 (ARF GAP GIT1) (Cool-associated and tyrosine-phosphorylated protein 1) (CAT-1) (CAT1) (G protein-coupled receptor kinase-interactor 1) (GRK-interacting protein 1) | PTPPLP | 479 | 484 |
| **Q9Y3L3** | SH3BP1 | SH3 domain-binding protein 1 (3BP-1) | PPTPPQP | 624 | 630 |
| **Q9Y3Q0** | NAALAD2 | N-acetylated-alpha-linked acidic dipeptidase 2 (EC 3.4.17.21) (Glutamate carboxypeptidase III) (GCPIII) (N-acetylated-alpha-linked acidic dipeptidase II) (NAALADase II) | PLTPGYP | 257 | 263 |
| **Q9Y3Q4** | HCN4 | Potassium/sodium hyperpolarization-activated cyclic nucleotide-gated channel 4 | PTPTP | 767 | 771 |
| **Q9Y3Q8** | TSC22D4 THG1 TILZ2 | TSC22 domain family protein 4 (TSC22-related-inducible leucine zipper protein 2) (Tsc-22-like protein THG-1) | PPTPQPP | 30 | 36 |
| **Q9Y3S1** | WNK2 KIAA1760 PRKWNK2 SDCCAG43 P/OKcl.13 | Serine/threonine-protein kinase WNK2 (EC 2.7.11.1) (Antigen NY-CO-43) (Protein kinase lysine-deficient 2) (Protein kinase with no lysine 2) (Serologically defined colon cancer antigen 43) | PSTPMP | 713 | 718 |
| **Q9Y3X0** | CCDC9 | Coiled-coil domain-containing protein 9 | PGTPRPP | 81 | 87 |
| **Q9Y446** | PKP3 | Plakophilin-3 | PTPPMP | 153 | 158 |
| **Q9Y466** | NR2E1 TLX | Nuclear receptor subfamily 2 group E member 1 (Nuclear receptor TLX) (Protein tailless homolog) (Tll) (hTll) | PTPKYP | 162 | 167 |
| **Q9Y467** | SALL2 KIAA0360 SAL2 ZNF795 | Sal-like protein 2 (Zinc finger protein 795) (Zinc finger protein SALL2) (Zinc finger protein Spalt-2) (Sal-2) (hSal2) | PTPAP | 298 | 302 |
| **Q9Y4A5** | TRRAP PAF400 | Transformation/transcription domain-associated protein (350/400 kDa PCAF-associated factor) (PAF350/400) (STAF40) (Tra1 homolog) | PATPVTP | 515 | 521 |
| **Q9Y4B5** | MTCL1 CCDC165 KIAA0802 SOGA2 | Microtubule cross-linking factor 1 (Coiled-coil domain-containing protein 165) (PAR-1-interacting protein) (SOGA family member 2) | PTPSP | 250 | 254 |
| **Q9Y4E8** | USP15 KIAA0529 | Ubiquitin carboxyl-terminal hydrolase 15 (EC 3.4.19.12) (Deubiquitinating enzyme 15) (Ubiquitin thioesterase 15) (Ubiquitin-specific-processing protease 15) (Unph-2) (Unph4) | PSTPKSP | 224 | 230 |
| **Q9Y4F5** | CEP170B FAM68C KIAA0284 | Centrosomal protein of 170 kDa protein B (Centrosomal protein 170B) (Cep170B) | PPTPPP | 540 | 545 |
| **Q9Y4H2** | IRS2 | Insulin receptor substrate 2 (IRS-2) | PWTPGQP | 1219 | 1225 |
| **Q9Y4R8** | TELO2 KIAA0683 | Telomere length regulation protein TEL2 homolog (Protein clk-2 homolog) (hCLK2) | PNTPCLP | 640 | 646 |
| **Q9Y520** | PRRC2C BAT2D1 BAT2L2 KIAA1096 XTP2 | Protein PRRC2C (BAT2 domain-containing protein 1) (HBV X-transactivated gene 2 protein) (HBV XAg-transactivated protein 2) (HLA-B-associated transcript 2-like 2) (Proline-rich and coiled-coil-containing protein 2C) | PTPAP | 1874 | 1878 |
| **Q9Y566** | SHANK1 | SH3 and multiple ankyrin repeat domains protein 1 (Shank1) (Somatostatin receptor-interacting protein) (SSTR-interacting protein) (SSTRIP) | PTPAFP | 692 | 697 |
| **Q9Y5C1** | ANGPTL3 ANGPT5 UNQ153/PRO179 | Angiopoietin-related protein 3 (Angiopoietin-5) (ANG-5) (Angiopoietin-like protein 3) [Cleaved into: ANGPTL3(17-221); ANGPTL3(17-224)] | PETPEHP | 153 | 159 |
| **Q9Y5E1** | PCDHB9 PCDH3H | Protocadherin beta-9 (PCDH-beta-9) (Protocadherin-3H) | PITPHLP | 769 | 775 |
| **Q9Y5G1** | PCDHGB3 | Protocadherin gamma-B3 (PCDH-gamma-B3) | PTPSDP | 679 | 684 |
| **Q9Y5P8** | PPP2R3B PPP2R3L | Serine/threonine-protein phosphatase 2A regulatory subunit B'' subunit beta (PP2A subunit B isoform PR48) (Protein phosphatase 2A 48 kDa regulatory subunit) | PGTPGP | 74 | 79 |
| **Q9Y5V0** | ZNF706 HSPC038 PNAS-113 | Zinc finger protein 706 | PKTPLPP | 63 | 69 |
| **Q9Y5Y0** | FLVCR1 FLVCR | Feline leukemia virus subgroup C receptor-related protein 1 (Feline leukemia virus subgroup C receptor) (hFLVCR) | PQTPLAP | 67 | 73 |
| **Q9Y5Z0** | BACE2 AEPLC ALP56 ASP21 CDA13 UNQ418/PRO852 | Beta-secretase 2 (EC 3.4.23.45) (Aspartic-like protease 56 kDa) (Aspartyl protease 1) (ASP1) (Asp 1) (Beta-site amyloid precursor protein cleaving enzyme 2) (Beta-site APP cleaving enzyme 2) (Down region aspartic protease) (DRAP) (Memapsin-1) (Membrane-associated aspartic protease 1) (Theta-secretase) | PTPGP | 45 | 49 |
| **Q9Y618** | NCOR2 CTG26 | Nuclear receptor corepressor 2 (N-CoR2) (CTG repeat protein 26) (SMAP270) (Silencing mediator of retinoic acid and thyroid hormone receptor) (SMRT) (T3 receptor-associating factor) (TRAC) (Thyroid-, retinoic-acid-receptor-associated corepressor) | PPTPPP | 782 | 787 |
| **Q9Y666** | SLC12A7 KCC4 | Solute carrier family 12 member 7 (Electroneutral potassium-chloride cotransporter 4) (K-Cl cotransporter 4) | PGTPEGP | 28 | 34 |
| **Q9Y679** | AUP1 | Ancient ubiquitous protein 1 | PVTPQP | 431 | 436 |
| **Q9Y6F9** | WNT6 | Protein Wnt-6 | PGTPGPP | 149 | 155 |
| **Q9Y6G9** | DYNC1LI1 DNCLI1 | Cytoplasmic dynein 1 light intermediate chain 1 (LIC1) (Dynein light chain A) (DLC-A) (Dynein light intermediate chain 1, cytosolic) | PTTPTSP | 511 | 517 |
| **Q9Y6I3** | EPN1 | Epsin-1 (EH domain-binding mitotic phosphoprotein) (EPS-15-interacting protein 1) | PTPDP | 346 | 350 |
| **Q9Y6J0** | CABIN1 KIAA0330 | Calcineurin-binding protein cabin-1 (Calcineurin inhibitor) (CAIN) | PTPLTP | 1810 | 1815 |
| **Q9Y6J3** | SMAD5-AS1 DAMS SMAD5OS | SMAD5 antisense gene protein 1 (10.3 kDa proline-rich protein DAMS) (SMAD5 antisense RNA 1) (SMAD5 opposite strand protein) | PATPPPP | 11 | 17 |
| **Q9Y6L6** | SLCO1B1 LST1 OATP1B1 OATP2 OATPC SLC21A6 | Solute carrier organic anion transporter family member 1B1 (Liver-specific organic anion transporter 1) (LST-1) (OATP-C) (Sodium-independent organic anion-transporting polypeptide 2) (OATP-2) (Solute carrier family 21 member 6) | PQTPNKP | 280 | 286 |
| **Q9Y6R7** | FCGBP | IgGFc-binding protein (Fcgamma-binding protein antigen) (FcgammaBP) | PPTPCPP | 1430 | 1436 |
| **Q9Y6V0** | PCLO ACZ KIAA0559 | Protein piccolo (Aczonin) | PTPGKP | 309 | 314 |
| **Q9Y6W5** | WASF2 WAVE2 | Wiskott-Aldrich syndrome protein family member 2 (WASP family protein member 2) (Protein WAVE-2) (Verprolin homology domain-containing protein 2) | PGTPPPP | 344 | 350 |
| **Q9Y6X0** | SETBP1 KIAA0437 | SET-binding protein (SEB) | PITPSSP | 563 | 569 |
| **Q9Y6X6** | MYO16 KIAA0865 MYO16B NYAP3 | Unconventional myosin-XVI (Neuronal tyrosine-phosphorylated phosphoinositide-3-kinase adapter 3) (Unconventional myosin-16) | PSTPPPP | 1570 | 1576 |
| **Q9Y6X9** | MORC2 KIAA0852 ZCWCC1 | MORC family CW-type zinc finger protein 2 (Zinc finger CW-type coiled-coil domain protein 1) | PTPRP | 633 | 637 |
