# Supplementary material for: Paired Immunoglobulin-like Type 2 Receptor Alpha G78R variant alters ligand binding and confers protection to Alzheimer's disease
Source: PLoS Genet. 2018 Nov 2;14(11):e1007427. doi: 10.1371/journal.pgen.1007427 (PMC6235402; doi:10.1371/journal.pgen.1007427)
Supplement: S4 Table — (DOC) [file pgen.1007427.s014.doc]

| **18hrs** |  |  |  | **LDH values** |  |  |
| --- | --- | --- | --- | --- | --- | --- |
|  | **MOI** | **10.000** | **1.000** | **0.100** | **0.010** | **lysed cells** |
| **Pair1** | **R78** | **0.269** | **0.021** | **-0.019** | **0.001** | **2.109** |
|  | **G78** | **1.059** | **0.455** | **0.063** | **0.020** | **1.866** |
| **Pair2** | **R78** | **0.241** | **0.039** | **-0.010** | **0.003** | **2.254** |
|  | **G78** | **0.734** | **0.378** | **0.089** | **0.041** | **2.599** |
| **Pair3** | **R78** | **0.046** | **0.009** | **0.005** | **-0.004** | **2.586** |
|  | **G78** | **0.132** | **0.092** | **0.144** | **0.025** | **1.649** |
| **Pair4** | **R78** | **0.155** | **0.026** | **-0.005** | **-0.025** | **2.987** |
|  | **G78** | **0.504** | **0.303** | **0.093** | **0.037** | **2.734** |
| **Pair5** | **R78** | **0.093** | **-0.035** | **-0.051** | **-0.066** | **2.934** |
|  | **G78** | **0.215** | **0.087** | **-0.010** | **-0.018** | **2.204** |
|  |  |  |  | **% cytotoxicity** |  |  |
|  | **MOI** | **10.000** | **1.000** | **0.100** | **0.010** | **lysed cells** |
| **Pair1** | **R78** | **12.745** | **0.988** | **-0.885** | **0.040** | **100.000** |
|  | **G78** | **56.736** | **24.370** | **3.386** | **1.081** | **100.000** |
| **Pair2** | **R78** | **10.671** | **1.738** | **-0.459** | **0.118** | **100.000** |
|  | **G78** | **28.251** | **14.555** | **3.437** | **1.590** | **100.000** |
| **Pair3** | **R78** | **1.779** | **0.329** | **0.193** | **-0.155** | **100.000** |
|  | **G78** | **7.977** | **5.581** | **8.705** | **1.517** | **100.000** |
| **Pair4** | **R78** | **5.189** | **0.870** | **-0.167** | **-0.837** | **100.000** |
|  | **G78** | **18.435** | **11.064** | **3.402** | **1.335** | **100.000** |
| **Pair5** | **R78** | **3.187** | **-1.193** | **-1.721** | **-2.232** | **100.000** |
|  | **G78** | **9.757** | **3.948** | **-0.431** | **-0.794** | **100.000** |
