# Supplementary material for: Paired Immunoglobulin-like Type 2 Receptor Alpha G78R variant alters ligand binding and confers protection to Alzheimer's disease
Source: PLoS Genet. 2018 Nov 2;14(11):e1007427. doi: 10.1371/journal.pgen.1007427 (PMC6235402; doi:10.1371/journal.pgen.1007427)
Supplement: S5 Table — (DOC) [file pgen.1007427.s015.doc]

| **36hrs** |  |  |  | **LDH values** |  |  |
| --- | --- | --- | --- | --- | --- | --- |
|  | **MOI** | **10** | **1** | **0.1** | **0.01** | **lysed cells** |
| **Pair1** | **R78** | **1.87** | **1.72** | **1.28** | **0.16** | **2.76** |
|  | **G78** | **1.64** | **1.54** | **1.51** | **0.36** | **1.93** |
| **Pair2** | **R78** | **1.63** | **1.89** | **1.41** | **0.16** | **2.81** |
|  | **G78** | **2.78** | **2.91** | **3.05** | **1.80** | **3.22** |
| **Pair3** | **R78** | **2.34** | **2.39** | **0.88** | **-0.18** | **2.52** |
|  | **G78** | **1.39** | **1.57** | **1.45** | **0.84** | **1.31** |
| **Pair4** | **R78** | **2.52** | **2.61** | **1.32** | **0.10** | **2.87** |
|  | **G78** | **2.74** | **2.82** | **2.27** | **0.53** | **2.90** |
| **Pair5** | **R78** | **2.93** | **2.93** | **2.64** | **0.04** | **2.86** |
|  | **G78** | **2.34** | **2.31** | **2.49** | **1.47** | **2.86** |
|  |  |  |  | **% cytotoxicity** |  |  |
|  | **MOI** | **10** | **1** | **0.1** | **0.01** | **lysed cells** |
| **Pair1** | **R78** | **67.70** | **62.46** | **46.27** | **5.65** | **100** |
|  | **G78** | **59.56** | **55.97** | **54.74** | **12.92** | **100** |
| **Pair2** | **R78** | **59.27** | **68.56** | **51.01** | **5.68** | **100** |
|  | **G78** | **100.72** | **105.65** | **110.60** | **65.23** | **100** |
| **Pair3** | **R78** | **84.83** | **86.59** | **31.99** | **-6.44** | **100** |
|  | **G78** | **50.32** | **56.79** | **52.55** | **30.48** | **100** |
| **Pair4** | **R78** | **91.37** | **94.78** | **47.73** | **3.54** | **100** |
|  | **G78** | **99.24** | **102.16** | **82.42** | **19.13** | **100** |
| **Pair5** | **R78** | **106.33** | **106.33** | **95.91** | **1.61** | **100** |
|  | **G78** | **84.88** | **83.60** | **90.14** | **53.17** | **100** |
